# Diastereoselective hydride transfer enables a synthesis of chiral 1,5-carboxamido-trifluoromethylcarbinols†

**DOI:** 10.1039/d4sc05049e

**Published:** 2024-09-09

**Authors:** Roberto Tinelli, Manuel Schupp, Immo Klose, Saad Shaaban, Boris Maryasin, Leticia González, Nuno Maulide

**Affiliations:** a Institute of Organic Chemistry, University of Vienna Währinger Straße 38 1090 Vienna Austria nuno.maulide@univie.ac.at; b Vienna Doctoral School in Chemistry, University of Vienna Währinger Straße 42 1090 Vienna Austria; c CeMM – Research Center for Molecular Medicine of the Austrian Academy of Sciences Lazarettgasse 14, AKH BT 25.3 1090 Vienna Austria; d Institute of Theoretical Chemistry, University of Vienna Währinger Straße 17 1090 Vienna Austria

## Abstract

The deployment of fluorinated functional groups has become a widespread tool in medicinal chemistry due to the impact of fluorine on lipophilicity and metabolic stability. Among these compounds, enantiopure secondary trifluoromethylcarbinols are recurrent features in bioactive compounds. Herein, we present a diastereoselective redox-neutral process allowing the stereospecific synthesis of 1,5-carboxamido-trifluoromethylcarbinols through the formal reduction of a trifluoromethylketone into a trifluoromethylcarbinol. A combined experimental and computational investigation unveiled a network of interconnected equilibria leading to a key hydride transfer event.

## Abstract 2

The synthesis of enantiopure tertiary trifluoromethylcarbinols (recurrent features in bioactive compounds) was achieved. We report herein a diastereoselective redox-neutral process allowing the synthesis of 1,5-carboxamido-trifluoromethylcarbinols.

## Graphical abstract

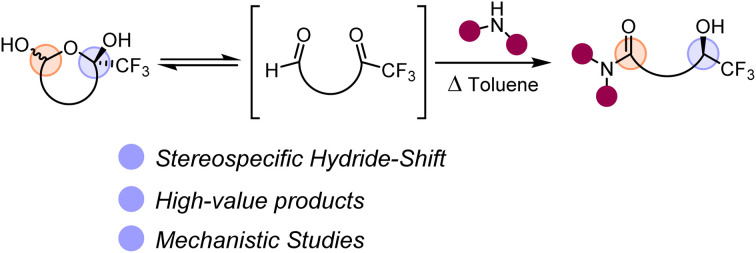

## Introduction

**Scheme 1:**
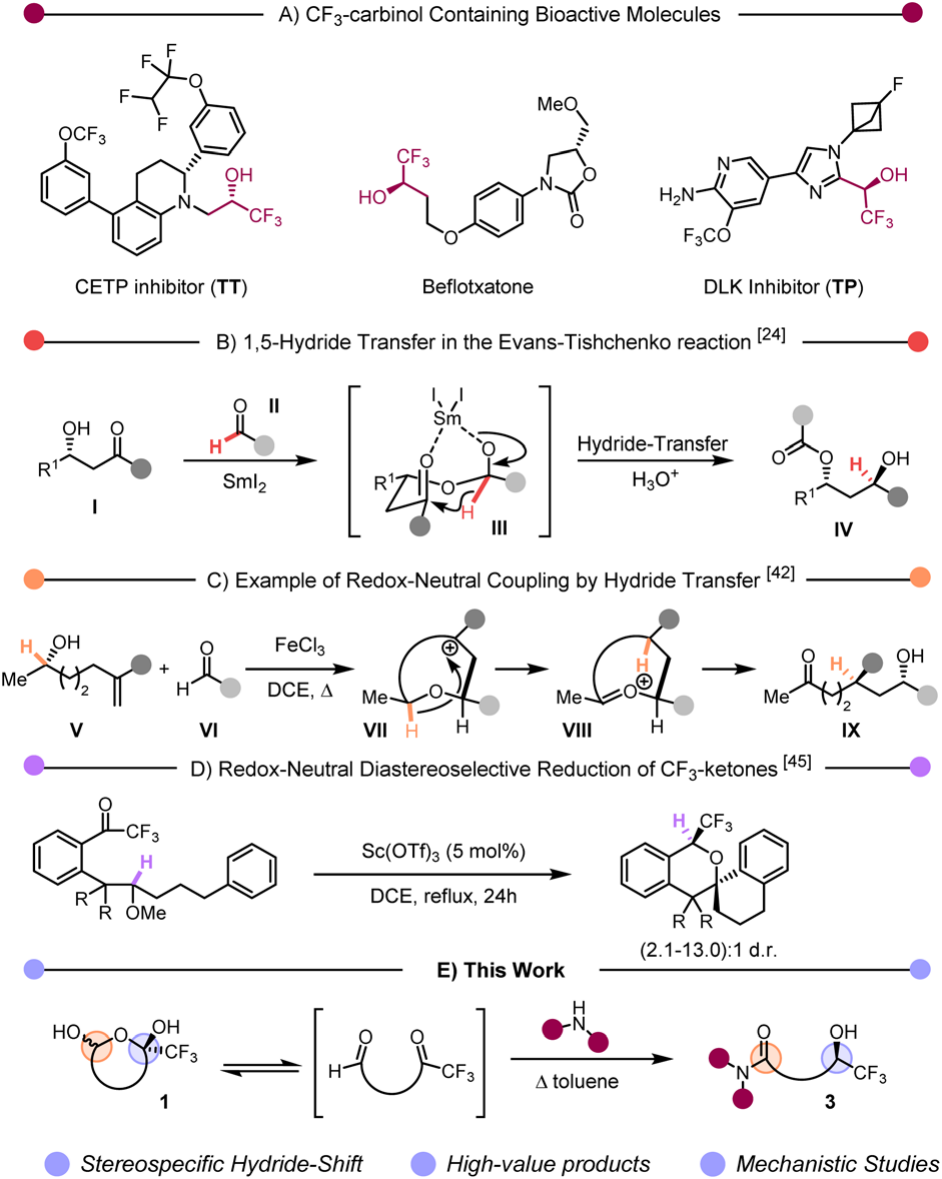
(A) Examples of bioactive CF_3_ carbinols. (B) Example of 1,5-hydride shift in the Evans–Tishchenko reaction. (C) Enantioselective redox-neutral reduction of activated aldehydes. (D) Diastereoselective reduction of CF_3_-ketones. (E) Stereospecific synthesis of 1,5-amido CF_3_ carbinols.

The introduction of fluorinated functional groups to organic compounds has become a recurrent tool in medicinal chemistry, given both the pronounced influence of fluorine on lipophilicity and metabolic stability, and the resulting boost of physicochemical and biological properties. Accordingly, a growing proportion of newly approved drugs contain at least a fluorine atom.^1–6^ Among the (by now) many fluorinated moieties commonly employed, enantiopure secondary trifluoromethylcarbinols are recurrent features in bioactive compounds, such as the cholesteryl ester transfer protein (CETP) inhibitor TT, the monoamine oxidase A inhibitor befloxatone or the dual leucine zipper kinase (DLK) inhibitor TP (Scheme 1A).^7–10^ Although not ideally suited for the synthesis of the trifluoromethylcarbinol scaffold in the latter compounds, several methods for the synthesis of such motifs have recently been developed involving direct reduction of CF_3_-ketones,^11–17^ or nucleophilic addition to trifluoroacetaldehyde (or its hemiacetal).^18–21^ In particular, the former methods require the use of stoichiometric amounts of bulky boranes,^11,13^ or, when resorting to catalytic protocols, high pressures of H_2_ to tackle the poor reactivity of CF_3_-ketones.^14–17^ In contrast, the latter methods encounter challenges regarding functional group tolerance and limited diastereomeric ratios.

Therefore, and despite these advances, alternative mild methods allowing the synthesis of densely substituted secondary trifluoromethylcarbinols with high levels of diastereo- and enantiomeric excess are still in demand.

In recent years, so-called “redox-neutral” synthetic methods have emerged^22,23^ as atom-economical processes that effect changes to molecular connectivity while simultaneously adjusting the redox state of two functional groups.^24–26^ Although recently somewhat re-profiled, the field effectively includes classical transformations such as the venerable Tishchenko (and Evans–Tishchenko) reaction (Scheme 1B).^27^ In its most commonly deployed variant, a β-ketoalcohol (I) and an aldehyde (II) associate into a hemiketal intermediate (III). In the presence of SmI_2_, III can undergo a 1,5-hydride transfer delivering IV in stereoselective fashion.^28–48^ Previously, our group has deployed hydride transfers as devices to accomplish reductive couplings that proceed by cationic mechanisms. In a notable example, stereocontrolled alkene–aldehyde coupling could be achieved with unique selectivity by engendering a cyclic carbocation reactive intermediate VIII. The subsequent hydride transfer event forms oxocarbenium VIII, which ultimately undergoes hydrolysis to the observed coupling product IX (Scheme 1C).^49–51^ Recent work by Mori demonstrated the possibility of using CF_3_-ketones as hydride acceptors to create trifluoromethyl-ethers in a diastereoselective fashion (Scheme 1D).^52–54^ In our continued efforts to further extend and harness the synthetic possibilities offered by redox neutral processes, we herein report a diastereoselective hydride transfer process allowing the stereospecific synthesis of 1,5-carboxamido-trifluoromethylcarbinols 3 from cyclic hemiacetals 1 (Scheme 1E).

## Results and discussion

During our research on inverse shuttle catalysis, we serendipitously discovered that, in the presence of a secondary amine, 1 was thermally converted to 3 in a metal-free process.^55^ Indeed, initial experiments showed that organocatalytic Hetero-Diels–Alder adduct^56^1a reacts with pyrrolidine at room temperature in toluene to deliver hemiaminal 4a in quantitative yield (Table 1, entry 1).

**Table 1:** Selected screening condition for the 1,5-H shift to from 3a

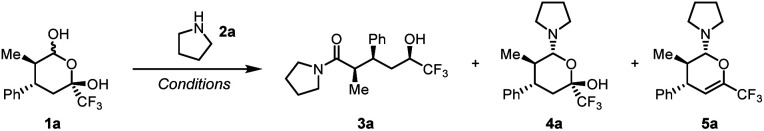							
Entry	Eq. 2a	Additive	Solvent	*T* (°C)	Yield 3a	Yield 4a	Yield 5a
1^a^	1.1	—	Toluene	25	—	100	—
2^a^	1.1	—	Toluene	70	60%	21%	—
3^a^	1.1	—	Toluene	100	82%	8%	—
4^a^	1.1	—	DMSO	100	—	—	—
5^a^	1.1	—	DCE	80	22%	28%	—
6^a^^,^^b^	1.1	BF_3_·OEt_2_	Toluene	100	—	—	17%
7^a^^,^^c^	1.1	Sc(OTf)_3_	Toluene	100	30%	—	—
8^a^^,^^d^	1.1	3 Å MS	Toluene	100	53%	27%	—
9^e^^,^^f^	2.0	—	Toluene	100	89%	—	—

a 1a (0.1 mmol, 0.04 M) 24 h.

b BF_3_·OEt_2_ 10%.

c Sc(OTf)_3_ 10%.

d MS 3 A.

e 1a (0.15 mmol, 0.04 M), 48 h.

f d.r. > 20 : 1, ee_1a_ = 94%, ee_3a_ = 90%.

Conversely, heating the mixture led to formation of the trifluoromethyl carbinol product 3a in good yield, accompanied by elimination and formation of byproduct 5a. Importantly, hemiaminal 4a was completely consumed (entry 2). Screening of different solvents showed a negative impact of increasing solvent polarity (entries 4–5), and the addition of Lewis acid catalysts (entries 6–7) or drying agents (entry 8) were found to also not have a beneficial effect on the reaction outcome. As detailed in the ESI,† the reaction revealed a good stability towards variation of concentration, reaction time and stoichiometry of pyrrolidine (see ESI, Section 3† for a full table of optimisation), and we ultimately determined that addition of 2 equivalents of amine allowed isolation of 3a in an optimised yield of 89% (entry 9). Notably, 3a is formed as a single diastereomer in 90% enantiomeric excess.

**Scheme 2:**
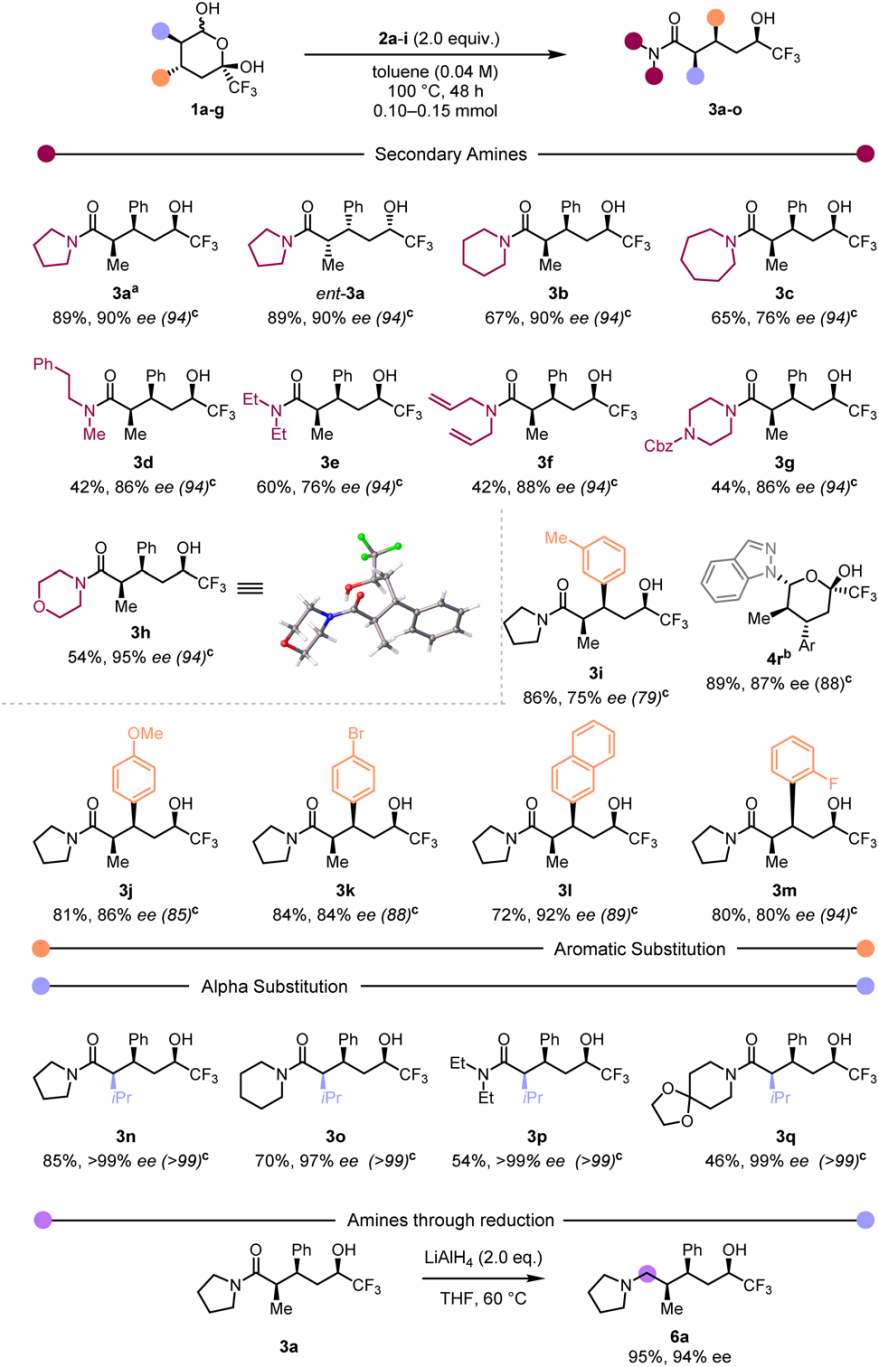
Reaction Scope.^*a*^ Scale-up of the reaction: 0.5 mmol–84% yield, 2.0 mmol–80% yield.^*b*^ Heating product 4r in the normal reaction conditions led to 3k with a 75% yield (Ar = 4-bromophenyl).^*c*^ ee of the corresponding starting material 1a–g.

With optimised conditions in hand we assessed the scope of this redox-neutral process (Scheme 2).

Employing hemiketal *ent*-1a (Scheme 2) resulted in product *ent*-3a with the same diastereo- and enantioselectivity as its antipode. Focusing on the amine component, we were pleased to observe that cyclic amines generally led to the desired products in very good yields (3b and 3c). However, a slight decrease in yield was observed when acyclic (3d–3f) or heterocyclic (3g and 3h) amines were employed. Single crystal X-ray analysis of 3h and 3k (the latter not shown in Scheme 2) provided unambiguous assignment of the absolute configuration of our products.^57^ Variation in the arene substituent revealed that both electron-rich and electron-poor aryls afforded the products in high yield (3i–3m). Replacing the α-methyl group with a more hindered *iso*-propyl group (3n–3q) led to good yields and excellent enantioselectivities. Worthy of note, employing indazole as the amine component led to the formation of a hemiaminal 4r that failed to afford the trifluoromethylcarbinol product. We believe this could be a consequence of the reduced availability of the indazole nitrogen lone pair. Representative limitations of the reported method can be found in the ESI† (primary amines, highly-hindered secondary amines, ketones, esters). In addition, reduction of the carboxamide delivered aminoalcohol 6a in excellent yield and ee value.

**Scheme 3:**
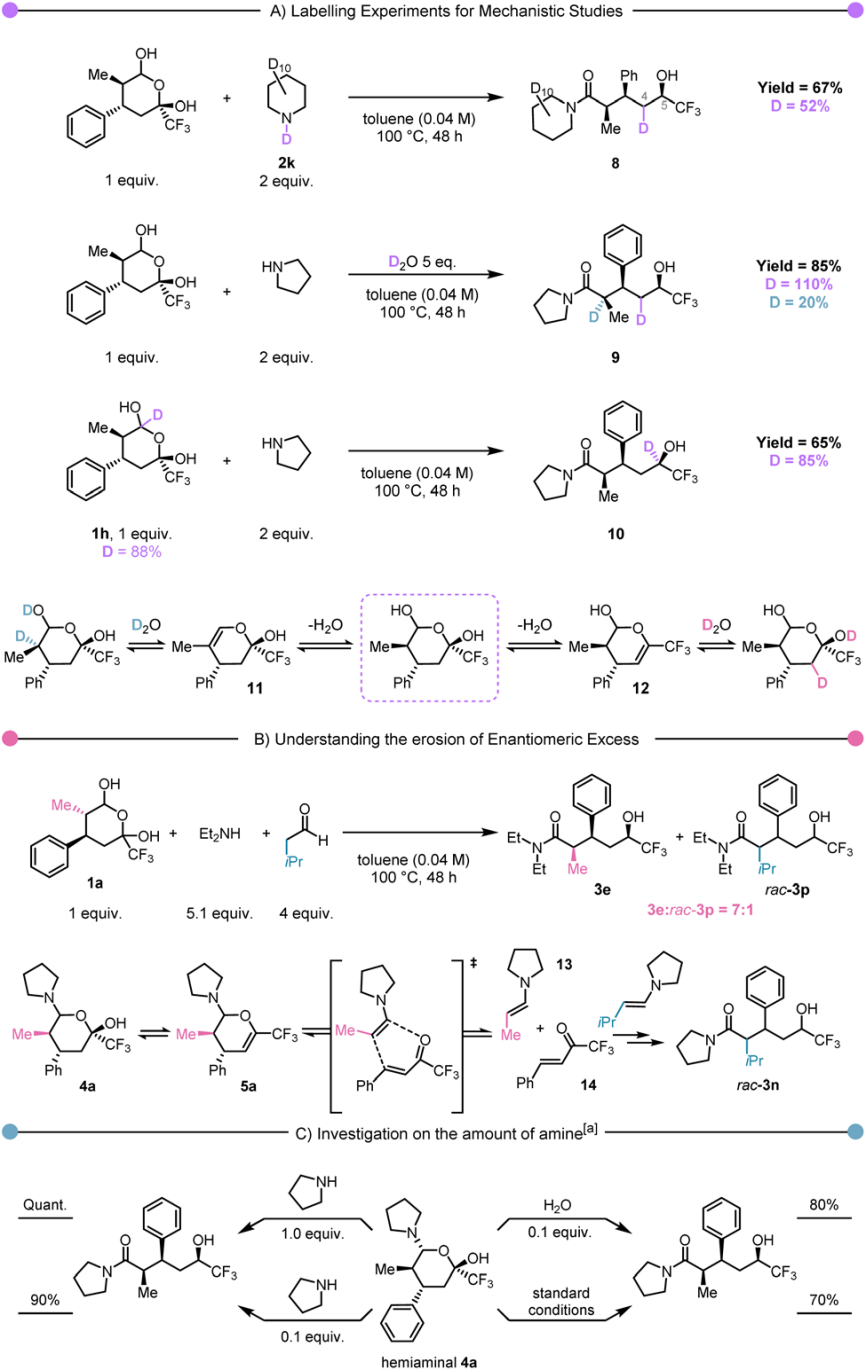
Mechanistic experiments. (A) D-labelled experiments for the hydride shift identification. (B) Demonstration of a retro Diels–Alder process (C) studies on the hemiaminal intermediate reactivity to improve the understanding on the reaction mechanism. ^*a*^Toluene (0.04 M), 100 °C, 48 h.

From the outset, we were interested in elucidating the mechanism of this atom-economical rearrangement. To explore the plausibility of a hydride originating from the amine component, we conducted the reaction in the presence of fully deuterated piperidine (Scheme 3A, first experiment). Interestingly, while this experiment demonstrated the absence of deuterium at the carbinol stereocentre (C-5) in 8, it unexpectedly revealed its presence on C-4. This intriguing observation suggested the existence of an elimination/rehydration equilibrium (*cf.*12, Scheme 3A, bottom). To validate this hypothesis, we performed the reaction in the presence of 5 equiv. of D_2_O, which resulted in deuterium labelling at C-4 and at C-2 in 9 (Scheme 3A, second experiment). In contrast to previous examples in the literature, this equilibrium did not lead to any epimerisation.^58^ Notably, the use of deuterated hemiacetal 1h led to a complete transfer of deuterium from the hemiacetal carbon to the CF_3_-alcohol carbon in 10, strongly supporting an internal hydride-transfer mechanism (Scheme 3A, third experiment).

Subsequently, our focus shifted towards unravelling the loss in enantiomeric excess reported for 3c and 3e. In particular (*cf.* equilibria shown in Scheme 3B), we speculated about the possibility of intermediate 5a undergoing retro Hetero-Diels–Alder to return to enamine 13 and trifluoromethyl enone 14. In the absence of a chiral catalyst, the equilibrium between 5a and 13–14 must lead to the racemisation of the product (Scheme 3B). To demonstrate this hypothesis, we envisioned capture by an alternative enamine in a secondary, parallel Hetero-Diels–Alder process. Therefore, we performed the rearrangement of 1a with diethylamine in the presence of isovaleraldehyde under thermal conditions. The observation of a mixture of products 3e and *rac*-3p in a 7 : 1 ratio (Scheme 3B) was a striking observation. Firstly, it unambiguously demonstrates the possibility of incorporation of an extraneous aldehyde, which supports the cycloreversion/cycloaddition hypothesis articulated earlier.

**Scheme 4:**
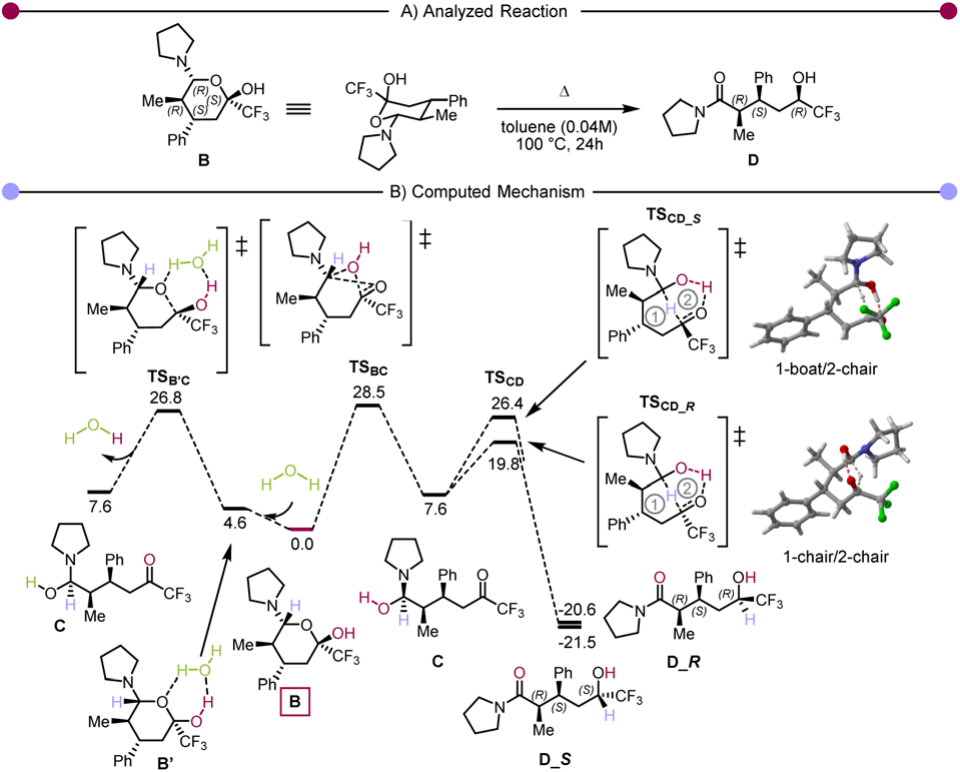
(A) The computationally analysed system. (B) Computed Gibbs free energy reaction profile (Δ*G*_373, toluene_). The hemiaminal B is used as a reference (0.0 kcal mol^−1^).

Secondly, the ratio between 3e and *rac*-3p suggests that the retro Hetero-Diels–Alder pathway, though operative, is not prevalent. Finally, we became curious about the possible role of hemiaminal 4a. Accordingly, it was isolated and exposed to different conditions (Scheme 3C). These experiments showed that once the hemiaminal intermediate is formed, the reaction proceeds quantitatively by the addition of an additional equivalent of pyrrolidine. The use of reduced (catalytic) amounts of pyrrolidine or water also allowed the formation of the product, albeit with a small drop in yield. We then performed computational studies at the density functional theory (DFT: B3LYP-D3(BJ)/def2-TZVP//B3LYP-D3(BJ)/def2-SVP,^59–65^ see ESI† for details) level to shed light on the reaction mechanism. The *in silico* computed process is shown in Scheme 4A, and Scheme 4B presents the computed Gibbs free energy profile. The hemiaminal B (4a) can intramolecularly ring-open to the acyclic form C. Notably, this step B → C is endergonic (Δ*G*(B → C) = 7.6 kcal mol^−1^) and, accordingly, has a relatively high kinetic barrier of 28.5 kcal mol^−1^. This computational result is in good agreement with the experimentally applied elevated temperature of 100 °C. The intermediate C undergoes a concerted combination of hydride and proton transfers, ultimately leading to the experimentally observed product D (3a). Interestingly, two diastereoisomeric pathways are possible for the step C→D, while only one diastereomer, depicted in Scheme 4B as D_*R*, was observed experimentally. Thermodynamically, the structures D_*S* and D_*R* are very similar (Δ*G*(C → D_*S*) = −29.1 kcal mol^−1^ and Δ*G*(C → D_*R*) = −28.2 kcal mol^−1^). However, we see a significant difference in the kinetic barriers favoring the formation of the experimentally observed product D_*R*. Indeed, Δ*G*^‡^(C → D_*S*) = 18.8 kcal mol^−1^ and Δ*G*^‡^(C → D_*R*) = 12.2 kcal mol^−1^. This barrier gap can be explained *via* analysis of the transition state structures shown on the right part of Scheme 4B. The transition state structures are bicyclic with interconnected six-membered rings. Both rings adopt a favourable chair conformation in the energetically lower transition state structure TS_CD__*_R_*. However, in the case of the energetically disfavoured transition state TS_CD__*_S_*, one of the rings adopts in a boat-like conformation, destabilising the entire structure. The described mechanistic pathway allows the formation of the product D in the absence of water. However, our experimental data shows that adding water to the system can benefit the reaction. In order to understand the influence of water, we have performed further calculations with an explicitly added singular water molecule. As shown in Scheme 4B (left), the initially formed reactant complex B′ is 4.6 kcal mol^−1^ higher as compared to the starting point B due to the entropic penalty paid for the involved water molecule. Remarkably, the subsequent transition state TS_B′C_ is better stabilised than the transition state TS_BC_: Δ*G*^‡^(B → C, *via*TS_BC_) = 28.5 kcal mol^−1^ and Δ*G*^‡^(B → C, *via*TS_B′C_) = 26.8 kcal mol^−1^. This is in complete agreement with the experimentally observed reaction acceleration in the presence of water. Both transition states, TS_BC_ and TS_B′C_, lead to the same intermediate C, but the reaction mechanism is dramatically different. Transition state TS_B′C_ is an energetically favorable six-membered cycle in which water assists the ring opening event. Instead of the hydroxy group shift within TS_BC_, the OH bond is cleaved in the transition state TS_B′C_, and the proton exchange with the water molecule allows the C–O bond to break. We also considered a four-membered ring alternative to the TS_B′C_ without water assistance, but the calculations strictly deny the possibility of this event, highlighting the crucial role of water.

## Conclusion

In conclusion, we have developed a new redox-neutral process enabling the stereospecific synthesis of multisubstituted 1,5-carboxamido trifluoromethylcarbinols from cyclic hemiketals. The mechanism of the process was studied in depth, revealing a network of interconnected equilibria that productively lead to the final product and showcasing the influence of water on the overall process. Extensive DFT calculations provided an elegant model to rationalise the observed diastereoselectivity. Notably, in this method, a redox-neutral event formally reduces a trifluoromethylketone into a trifluoromethylcarbinol, thus obviating the need for strongly nucleophilic (organometallic) reagents or high-pressured hydrogenations. We believe that this work further emphasises the growing importance of hydride transfer processes in mediating formal reductive transformations under mild conditions.

## Author contributions

The work was conceptualised by N. M. The experiments were performed by R. T., M. S. and I. K. B. M. performed the DFT calculations. The manuscript was written through contributions of all authors. S. S. and N. M. were involved in manuscript editing, finalizing and overall supervision of the project. N. M. and L. G. secured funding and supervised the entire work.

## Conflicts of interest

There are no conflicts to declare.

## Supplementary Material

SC-015-D4SC05049E-s001

## Data Availability

A data availability statement (DAS) is submitted alongside the article.
