# Supplementary material for: Diastereoselective hydride transfer enables a synthesis of chiral 1,5-carboxamido-trifluoromethylcarbinols
Source: Chem Sci. 2024 Sep 9;15(38):15751–6. doi: 10.1039/d4sc05049e (PMC11382188; doi:10.1039/d4sc05049e)
Supplement: SC-015-D4SC05049E-s001 [file SC-015-D4SC05049E-s001.pdf]

Supplementary Information (SI) for Chemical Science.
This journal is © The Royal Society of Chemistry 2024

Supplementary Information

Contents

1. General Information 3

2. Substrate preparation 4

2.1 Preparation of (*S*)-2-(diphenyl((trimethylsilyl)oxy)methyl)pyrrolidine (S1) 4

2.2 General procedure 1: Preparation of α,β-unsaturated CF_3 ketones 5

2.2.1. Characterizations of α,β-unsaturated CF_3 ketones 6

2.3 Synthesis of Nitropropane-1,1-d_2 (S2a): 8

2.4 Synthesis of propanal-1-d (S3a): 8

2.5 Preparation of the CF_3–Hemiacetals Starting Materials 9

2.5.1 General Procedure 2: CF_3–Hemiacetals 9

2.5.2 Characterizations of the prepared CF3-Hemiacetals Starting Materials 10

3. Optimization of the Hydride Transfer reaction 25

3.1 General Procedure 3: Preparation of 1,5-carboxamido-trifluoromethylcarbinols 27

3.2 Characterization of the CF_3-alcohol products 28

4. Mechanistic studies 65

4.1 Labelling Experiments 65

4.2 Hemiacetal Intermediate 68

4.3 Retro Diels-Alder Demonstration 69

5. Post Functionalization 72

6. Unsuccessful attempts 74

7. Computational details 75

7.1 Computational method 75

7.2 Cartesian coordinates (XYZ) 76

8. X-Ray Crystallographic data 83

9. NMR Spectra 90

9.1 Starting Materials 90

9.2 Hydride Transfer Products 103

9.3 Post functionalization Compounds 129

9.4 Mechanistically relevant Compounds 131

10. References 138

1. General Information

Unless otherwise stated, all glassware was flame-dried before use and all reactions were performed under an atmosphere of argon. All solvents were distilled from appropriate drying agents prior to use or, if purchased in anhydrous form, used as received from commercial suppliers. All other reagents were used as received from commercial suppliers, unless otherwise stated. Reaction progress was monitored by thin layer chromatography (TLC) performed on aluminium plates coated with silica gel F_{254} with 0.2 mm thickness. Chromatograms were visualized by fluorescence quenching with UV light at 254 nm or by staining using potassium permanganate. Flash column chromatography was performed using silica gel 60 (230-400 mesh, Merck and co.). Neat infra-red spectra were recorded using a Perkin-Elmer Spectrum 100 FT-IR spectrometer. Wavenumbers (vmax) are reported in cm^{-1}. Mass spectra were obtained using a Finnigan MAT 8200 or (70 eV) or an Agilent 5973 (70 eV) spectrometer, using electrospray ionization (ESI). All ^{1}H NMR, ^{13}C NMR and ^{19}F NMR spectra were recorded using a Bruker AV-400, AV-600 or AV-700 spectrometer at 300K. Chemical shifts are given in parts per million (ppm, δ), referenced to the solvent peak of $CDCl_3$, defined at δ = 7.26 ppm (^{1}H NMR) and δ = 77.16 (^{13}C NMR). Coupling constants are quoted in Hz (*J*). ^{1}H NMR splitting patterns were designated as singlet (s), doublet (d), triplet (t), quartet (q), pentet (p). Splitting patterns that could not be interpreted or easily visualized were designated as multiplet (m) or broad (br). Selected ^{13}C NMR spectra were recorded using the attached proton test (APT) to facilitate the confirmation and assignment of the structure. Optical rotation values were measured on a Perkin Elmer 341 polarimeter at 20 °C using a 100 mm path-length cuvette at 589 nm (c given in g/100 mL).

2. Substrate preparation

2.1 Preparation of (*S*)-2-(diphenyl((trimethylsilyl)oxy)methyl)pyrrolidine (S1)

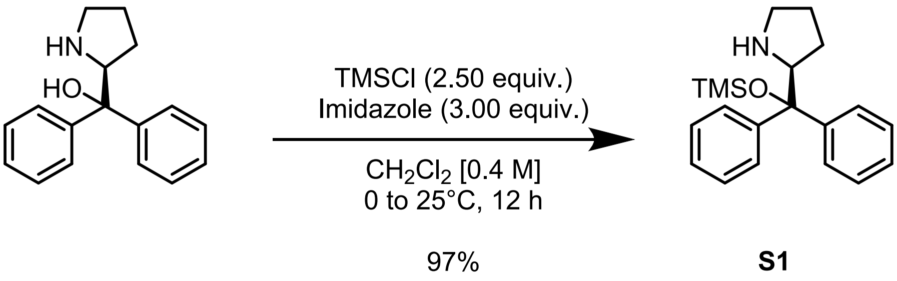

S1

To (*S*)-diphenylprolinol (4.00 g, 15.8 mmol, 1.00 equiv.) in CH_2Cl_2 (0.4 M, 40.0 mL) was added imidazole (3.22 g, 47.4 mmol, 3.00 equiv.) at 0 °C. TMSCl (5.00 mL, 39.5 mmol, 2.50 equiv.) was added dropwise and the reaction was stirred for 12h at 25 °C. After this time, MTBE (100 mL) was added to the reaction and the mixture was filtered. The organic phase was washed with H_2O (50.0 mL) and saturated aqueous NaCl (2 × 50.0 mL). Subsequently, the combined organic layers were dried over anhydrous $MgSO_4$, filtered and the solvent was removed under reduced pressure to obtain **S1** as a colorless oil (4.98 g, 15.3 mmol, 97%). All analytical data were in good accordance with those reported in the literature.[1]

2.2 General procedure 1: Preparation of α,β-unsaturated CF_3 ketones

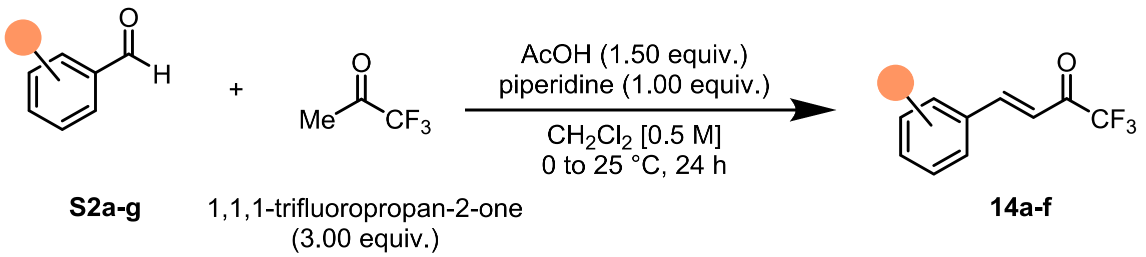

General Procedure 1: To a stirred solution of aldehydes **S2a-g** (1.00 equiv.), acetic acid (1.50 equiv.), and piperidine (1.00 equiv.) in dry CH_2Cl_2 (1.0 M) at 0 °C was added dropwise a solution of 1,1,1-trifluoropropan-2-one (3.00 equiv.) in dry CH_2Cl_2 (1.0 M). The mixture was stirred for 2h at 0 °C before being allowed to warm to 25 °C while being stirred for additional 24h. After this time, the reaction was quenched by the addition of a saturated aqueous solution of ammonium chloride. Subsequently the reaction mixture was extracted with EtOAc (3 × 15.0 mL) and washed with brine (1 × 15.0 mL). The combined organic layers were dried over anhydrous $MgSO_4$, filtered and the solvent was removed under reduced pressure. The crude product was purified by flash column chromatography on silica gel (pentane/CH_2Cl_2) to provide the α,β-unsaturated CF_3-ketones **14a-f**.

2.2.1. Characterizations of α,β-unsaturated CF_3 ketones

(*E*)-1,1,1-trifluoro-4-phenylbut-3-en-2-one (14a)

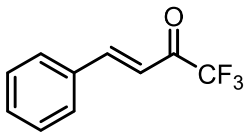

$C_{10}H_7F_3O$
MW: 200.05

Synthesized following General Procedure 1 on 40.0 mmol scale, from benzaldehyde and 1,1,1-trifluoropropan-2-one. Purification by flash column chromatography (pentane) gave the title compound in 58% yield (4.64 g, 23.2 mmol). All analytical data were in good accordance with those reported in the literature.[2]

(*E*)-1,1,1-trifluoro-4-(m-tolyl)but-3-en-2-one (14b)

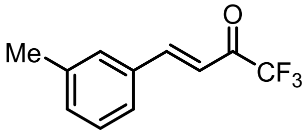

$C_{11}H_9F_3O$
MW: 214.06

Synthesized following General Procedure 1 on 8.00 mmol scale, from 3-methylbenzaldehyde and 1,1,1-trifluoropropan-2-one. Purification by flash column chromatography (pentane) gave the title compound in 69% yield (1.18 g, 5.52 mmol). All analytical data were in good accordance with those reported in the literature.[2]

(*E*)-1,1,1-trifluoro-4-(4-methoxyphenyl)but-3-en-2-one (14c)

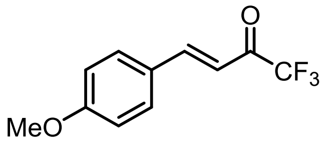

$C_{11}H_9F_3O_2$
MW: 230.05

Synthesized following General Procedure 1 on 8.00 mmol scale, from 4-methoxybenzaldehyde and 1,1,1-trifluoropropan-2-one. Purification by flash column chromatography (pentane/CH_2Cl_2) gave the title compound in 46% yield (846 mg, 3.68 mmol). All analytical data were in good accordance with those reported in the literature.[2]

(*E*)-4-(4-bromophenyl)-1,1,1-trifluorobut-3-en-2-one (14d)

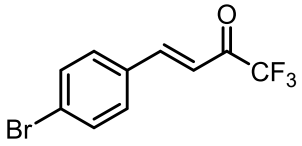

$C_9H_6F_3OBr$
MW: 277.96

Synthesized following General Procedure 1 on 8.00 mmol scale, from 4-bromobenzaldehyde and 1,1,1-trifluoropropan-2-one. Purification by flash column chromatography (pentane/CH_2Cl_2) gave the title compound in 54% yield (1.20 g, 4.32 mmol). All analytical data were in good accordance with those reported in the literature.[3]

(*E*)-1,1,1-trifluoro-4-(naphthalen-2-yl)but-3-en-2-one (14e)

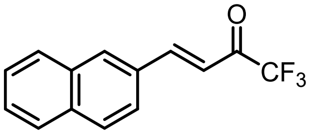

$C_{14}H_9F_4O$
MW: 250.06

Synthesized following General Procedure 1 on 10.0 mmol scale, from 2-naphthaldeyhde and 1,1,1-trifluoropropan-2-one. Purification by flash column chromatography (pentane/CH_2Cl_2) gave the title compound in 63% yield (1.58 g, 6.31 mmol). All analytical data were in good accordance with those reported in the literature.[4]

((*E*)-1,1,1-trifluoro-4-(2-fluorophenyl)but-3-en-2-one (14f)

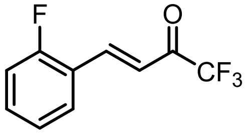

$C_{10}H_6F_4O$
MW: 218.04

Synthesized following General Procedure 1 on 8.00 mmol scale, from 2-fluorobenzaldehyde and 1,1,1-trifluoropropan-2-one. Purification by flash column chromatography (pentane/CH_2Cl_2) gave the title compound in 69% yield (1.20 g, 5.52 mmol). All analytical data were in good accordance with those reported in the literature.[2]

2.3 Synthesis of Nitropropane-1,1-d$_2$ (S2a):

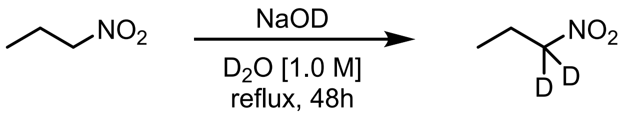

33.6 mmol

Yield 88%
D = 85%

A mixture of nitropropane (30.0 mL, 336 mmol, 1.00 equiv.) and deuterium oxide (30.0 mL, 1680 mmol, 5.00 equiv.) containing 3 drops of NaOD (40 wt% solution in D_2O) was refluxed over 48h at 100 °C. After this time, the reaction was diluted with 30 mL of CH_2Cl_2. Subsequently the reaction mixture was extracted with CH_2Cl_2 (3 × 5.0 mL). The combined organic layers were dried over anhydrous $MgSO_4$, filtered and the solvent was removed under reduced pressure. Finally, the crude product was purified by distillation to obtain the product (27.0 g, 296 mmol) with an overall yield of 88% and 85% of D incorporation. All analytical data were in good accordance with those reported in the literature.[5]

2.4 Synthesis of propanal-1-d (S3a):

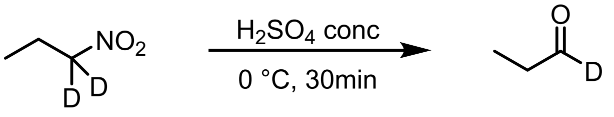

Yield 88%
D = 85%

Yield 21%
D = 96%

A solution of concentrated sulfuric acid (53.2 mL, 1.48 mol, 5.00 equiv.,) dissolved in 355 mL water was cooled in an ice-bath and stirred continuously. Another solution of nitropropane-1,1-d$_2$ (27.0 g, 0.296 mol, 1.00 equiv.) in 290 mL of ice-cold aqueous 15 % NaOH was prepared and put in a separatory funnel and added dropwise to the ice-cooled solution under vigorous stirring. At the end of the addition, the reaction mixture was stirred for 30min. After this time, the organic layers were separated by extraction and dried over anhydrous $MgSO_4$. Finally, the crude product was purified by distillation to obtain the product (3.66 g, 61.2 mmol) with an overall yield of 21% and 96% of D incorporation. All analytical data were in good accordance with those reported in the literature.[5]

2.5 Preparation of the CF_3–Hemiacetals Starting Materials

2.5.1 General Procedure 2: CF_3–Hemiacetals

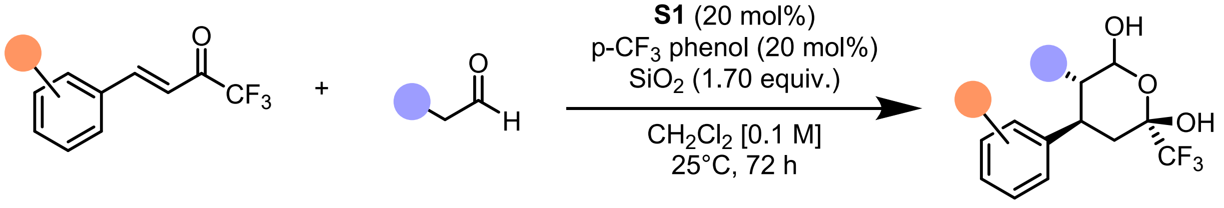

14a-f **S3** (1.20 equiv.) **1a-h**

General Procedure 2: To a stirred solution of the aldehyde **S3** (1.20 equiv.), organocatalyst **S1** (0.20 equiv.), *p*-trifluoromethylphenol (0.20 equiv.) and SiO_2 (1.70 equiv.) in CH_2Cl_2 (0.1 M) was added α,β-unsaturated trifluoromethyl ketone **14** (1.00 equiv.). Subsequently, the reaction mixture was stirred at 25 °C for 72h. After this time, the reaction mixture was filtered and the solvent was removed under reduced pressure. Finally, the reaction crude was purified by flash column chromatography on silica gel (heptane/EtOAc = 7:3) to obtain the desired products **1a-h**. [6]

Nota bene: The reaction time can have a strong influence on the final level of enantiomeric excess.

2.5.2 Characterizations of the prepared CF3-Hemiacetals Starting Materials

(2*S*,4*S*,5*R*)-5-methyl-4-phenyl-2-(trifluoromethyl)tetrahydro-2*H*-pyran-2,6-diol (1a)

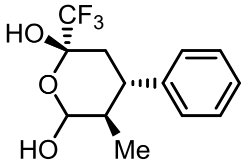

$C_{13}H_{15}F_3O_3$
MW: 276.10

Synthesized following General Procedure 2 on 5.00 mmol scale from **14a** and propionaldehyde (**S3b**). Purification by flash column chromatography (heptane/EtOAc = 7:3) gave the title compound in 43% yield (593.6 mg, 2.15 mmol) as a diastereomeric mixture in a 2.3:1.0 ratio.

^{1}H NMR (600 MHz, CDCl$_3$) δ δ 7.34 (t, J = 7.4 Hz, 2H), 7.27 –7.19 (m, 3H), 5.11 (d, J = 8.3 Hz, 0.3H), 4.65 (s, 0.7H), 4.65 (s, 0.3H), 4.21 (s, 0.3H), 3.75 (s, 0.7H), 3.58 (s, 0.7H), 3.20 (td, J = 12.4, 3.8 Hz, 0,3H), 2.92 (td, J = 12.3, 4.1 Hz, 0,7H), 2.08 – 2.01 (m, 0.7H), 1.96 (t, J = 13.2 Hz, 1H), 1.88 – 1.78 (m, 0.7H), 0.85 (d, J = 6.6 Hz, 2H), 0.80 (d, J = 6.9 Hz, 1H) ppm.

^{13}C NMR (151 MHz, CDCl$_3$) δ 141.6, 128.8, 127.6, 127.5, 127.1, 122.2 (q, *J* = 280.1 Hz), 97.2, 97.1, 95.4 (q, *J* = 32.6 Hz), 42.4, 42.1, 39.2, 36.2, 36.0, 34.8, 14.2, 13.7 ppm.

^{19}F NMR (565 MHz, CDCl$_3$) δ −87.30, −87.45 ppm.

HRMS (ESI$^+$): exact mass calculated for $[M+Na]^+$ ($C_{13}H_{15}F_3O_3Na^+$) requires *m/z* 299.0866, found 299.0868 *m/z*.

IR (neat) ν_{max}: 3261, 1140, 999, 701 cm^{-1}.

$[\alpha]_D^{20}$ = −0.07 (c = 1.0, $CHCl_3$).

Determination of enantiomeric excess: ee: 94%. Method description: Lux-Cellulose 1 250x4.6 mm, Particle size 5 μm, solvent system: *n*-heptane+0.1%IPA/IPA 95:5; flow 1 mL/min, 25 °C. Peak area 97.1% (Rt = 12.6 min), 2.9% (Rt = 16.5 min).

Enantioenriched Compound

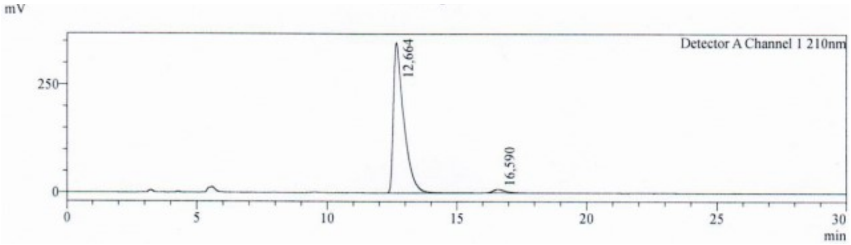

Detector A Channel 1 210nm

Peak#	Ret. Time	Area	Area%
1	12,664	9806717	97,177
2	16,590	284902	2,823
Total		10091619	100,000

Racemic Compound

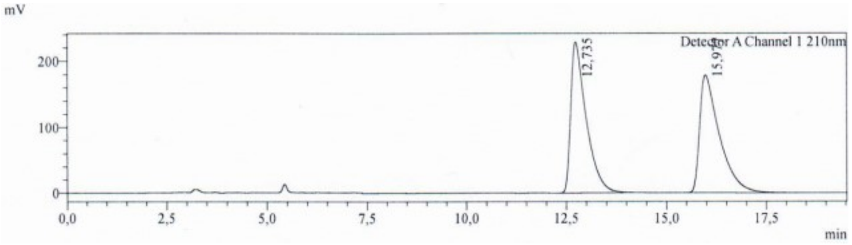

Detector A Channel 1 210nm

Peak#	Ret. Time	Area	Area%
1	12,735	6160633	49,947
2	15,977	6173691	50,053
Total		12334325	100,000

(2*S*,4*S*,5*R*)-5-methyl-4-(*m*-tolyl)-2-(trifluoromethyl)tetrahydro-2*H*-pyran-2,6-diol (1b)

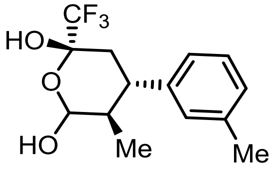

$C_{14}H_{17}F_3O_3$
MW: 290.11

Synthesized following General Procedure 2 on 5.00 mmol scale from **14b** and propionaldehyde (**S3b**). Purification by flash column chromatography (heptane/EtOAc = 7:3) gave the title compound in 14% yield (203.1 mg, 0.700 mmol) as a diastereomeric mixture in a 2.3:1.0 ratio.

^{1}H NMR (600 MHz, CDCl$_3$) δ 7.24 – 7.20 (m, 1H), 7.09 – 6.96 (m, 3H), 5.37 (s, 0.3H), 5.05 (t, *J* = 7.2 Hz, 0.7H), 4.00 (d, *J* = 15.7 Hz, 0.3H), 3.38 (s, 0.3H), 3.12 (td, *J* = 12.5, 3.8 Hz, 0.3H), 2.95 (dd, *J* = 32.7, 15.1 Hz, 0.7H), 2.86 (td, *J* = 12.3, 4.1 Hz, 0.7H), 2.77 (s, 0.7H), 2.35 (s, 0.9H), 2.35 (s, 2.1H), 2.19 – 2.08 (m, 0.6H), 2.07 – 1.97 (m, 1H), 1.97 – 1.88 (m, 0.7H), 1.81 – 1.71 (m, 0.7H), 0.85 (d, *J* = 6.6 Hz, 2.1H), 0.80 (d, *J* = 6.9 Hz, 0.9H) ppm.

^{13}C NMR (151 MHz, CDCl$_3$) δ 141.6, 141.6, 138.4, 128.7, 128.3, 128.3, 127.8, 127.8, 124.6, 124.5, 122.2 (q, *J* = 285.5 Hz), 122.2 (q, *J* = 285.5 Hz), 97.2, 97.2, 95.4 (q, *J* = 33.2 Hz), 42.3, 42.1, 39.2, 36.3, 35.9, 34.9, 21.4, 14.3, 13.8 ppm.

^{19}F NMR (565 MHz, CDCl$_3$) δ −87.27 (s), −87.43 (s) ppm.

IR (neat) ν_{max}: 3272, 1126, 1012, 728 cm^{-1}.

HRMS (ESI$^+$): exact mass calculated for [M+Na]$^+$ ($C_{14}H_{17}F_3O_3Na^+$) requires *m/z* 313.1022, found 313.1024 *m/z*.

$[\alpha]_D^{20}$ = 0.05 (c = 1.0, CHCl$_3$).

Determination of enantiomeric excess: ee: 79%. Method description: Chiralpack IH-3 150x4.6 mm, Particle size 5 μm, solvent system: *n*-heptane/IPA/EtOH 98:1:1; flow 1 mL/min, 25 °C. Peak area 10.5% (Rt = 11.4 min), 89.5% (Rt = 12.7 min).

Enantioenriched compound:

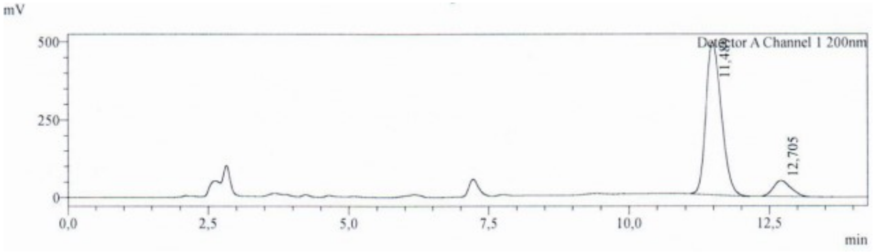

Detector A Channel 2 210nm			
Peak#	Ret. Time	Area	Area%
1	11,491	10078897	89,551
2	12,707	1175965	10,449
Total		11254862	100,000

Racemic compound:

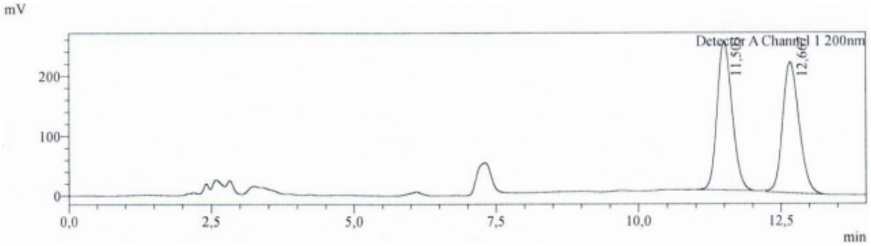

Detector A Channel 2 210nm			
Peak#	Ret. Time	Area	Area%
1	11,504	4782969	50,546
2	12,668	4679576	49,454
Total		9462545	100,000

(2*S*,4*S*,5*R*)-4-(4-methoxyphenyl)-5-methyl-2-(trifluoromethyl)tetrahydro-2*H*-pyran-2,6-diol (1c)

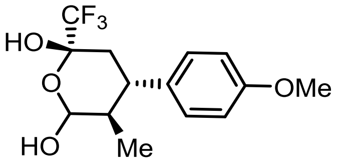

$C_{14}H_{17}F_3O_4$
MW: 306.11

Synthesized following General Procedure 2 on 5.00 mmol scale from **14c** and propionaldehyde (**S3b**). Purification by flash column chromatography (heptane/EtOAc = 7:3) gave the title compound in 18% yield (275.5 mg, 0.900 mmol) as a diastereomeric mixture in a 4.0:1.0 ratio.

^{1}H NMR (600 MHz, $CDCl_3$) δ 7.19 – 7.06 (m, 2H), 6.95 – 6.85 (m, 2H), 5.37 (dd, J = 6.2, 3.4 Hz, 0.2H), 5.04 (t, J = 8.3 Hz, 0.8H), 3.32 (s, 0.2H), 3.81 (s, 0.6H), 3.81 (s, 2.4H), 3.30 (d, J = 5.9 Hz, 0.2H), 3.11 (td, J = 12.4, 3.7 Hz, 0.2H), 2.92 (s, 0.8H), 2.87 – 2.81 (m, 0.8H), 2.71 (d, J = 8.0 Hz, 0.8H), 2.13 (dd, J = 13.4, 3.7 Hz, 0.2H), 2.07 (d, J = 25.6 Hz, 0.2H), 2.01 (dd, J = 13.6, 4.2 Hz, 1H), 1.91 (td, J = 13.2, 2.7 Hz, 0.8H), 1.76 – 1.67 (m, 0.8H), 0.84 (d, J = 2.4 Hz, 13H), 0.79 (d, J = 6.9 Hz, 0.6H).

^{13}C NMR (151 MHz, $CDCl_3$) δ 158.5, 133.8, 133.7, 128.5, 128.4, 122.2 (q, *J* = 285.4 Hz), 122.1 (q, *J* = 285.4 Hz), 114.2, 97.2, 97.2, 95.3 (q, *J* = 32.6 Hz), 55.3, 42.7, 42.6, 41.3, 39.5, 36.4, 35.0, 14.3, 13.7 ppm.

^{19}F NMR (565 MHz, $CDCl_3$) δ −87.27 (s), −87.43 (s) ppm.

IR (neat) ν_{max}: 3278, 2937, 1173, 1028, 1009 cm^{-1}.

HRMS (ESI$^+$): exact mass calculated for $[M+Na]^+$ ($C_{14}H_{17}F_3O_4Na^+$) requires *m/z* 329.0971, found 329.0972 *m/z*.

$[\alpha]_D^{20}$ = −0.12 (c = 1.0, $CHCl_3$).

Determination of enantiomeric excess: ee: 85%. Method description: Lux-Cellulose 1 250x4.6 mm, Particle size 5 µm, solvent system: *n*-heptane+0.1%IPA/IPA 98:2; flow 1 mL/min, 25 °C. Peak area 92.5% (Rt = 19.9 min), 7.5% (Rt = 14.7 min).

Enantioenriched Compound

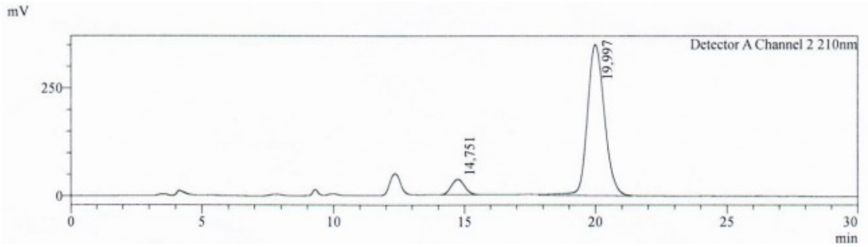

Detector A Channel 1 200nm

Peak#	Ret. Time	Area	Area%
1	14,747	1613730	7,799
2	19,994	19078837	92,201
Total		20692567	100,000

Racemic Compound

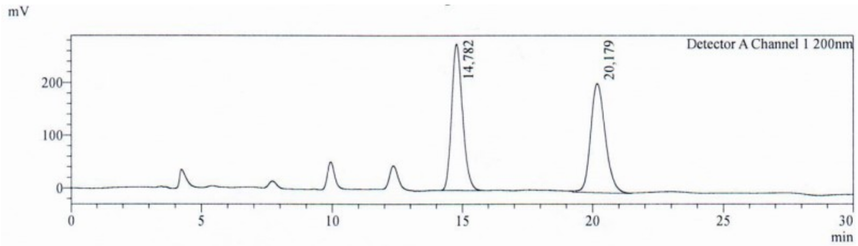

Detector A Channel 1 200nm

Peak#	Ret. Time	Area	Area%
1	14,782	8129188	50,219
2	20,179	8058299	49,781
Total		16187487	100,000

(2*S*,4*S*,5*R*)-4-(4-bromophenyl)-5-methyl-2-(trifluoromethyl)tetrahydro-2*H*-pyran-2,6-diol (1d)

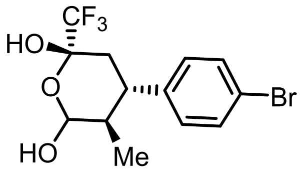

$C_{13}H_{14}F_3O_3Br$
MW: 354.01

Synthesized following General Procedure 2 on 5.00 mmol scale from **14d** and propionaldehyde (**S3b**). Purification by flash column chromatography (heptane/EtOAc = 7:3) gave the title compound in 18% yield (318.6 mg, 0.900 mmol) as a diastereomeric mixture in a 2.3:1.0 ratio.

^{1}H NMR (600 MHz, CDCl$_3$) δ 7.49 – 7.43 (m, 2H), 7.12 – 7.06 (m, 7H), 5.39 (dt, *J* = 5.1, 3.6 Hz, 0.3H), 5.08 (dd, *J* = 18.0, 10.0 Hz, 0.7H), 4.48 (s, 0.3H), 4.01 (d, *J* = 5.3 Hz, 0.3H), 3.51 (s, 0.7H), 3.32 (d, *J* = 7.5 Hz, 0.7H), 3.17 (td, *J* = 12.4, 3.7 Hz, 0.3H), 2.88 (td, *J* = 12.3, 4.1 Hz, 0.7H), 2.12 (dt, *J* = 7.5, 3.7 Hz, 0.3H), 2.10 – 2.03 (m, 0.3H), 2.03 – 1.84 (m, 1.7H), 1.79 – 1.68 (m, 0.7H), 0.83 (d, *J* = 6.6 Hz, 2.1H), 0.78 (d, *J* = 6.9 Hz, 0.9H) ppm.

^{13}C NMR (151 MHz, CDCl$_3$) δ 140.7, 140.6, 132.0, 132.0, 129.3, 129.3, 122.2 (q, *J* = 285.4 Hz), 122.1 (q, *J* = 284.7 Hz), 120.8, 97.0, 96.9, 95.3 (q, *J* = 32.8 Hz), 42.1, 41.7, 39.1, 36.0, 35.6, 34.8, 14.1, 13.6 ppm.

^{19}F NMR (565 MHz, CDCl$_3$) δ −87.07 (s), −87.30 (s) ppm.

IR (neat) ν_{max}: 3306, 2954, 1753, 1362, 1012, 704 cm^{-1}.

HRMS (ESI$^+$): exact mass calculated for $[M+Na]^+$ ($C_{13}H_{14}F_3O_3BrNa^+$) requires *m/z* 376.9971, found 376.9971 *m/z*.

$[\alpha]_D^{20}$ = −0.02 (c = 1.0, $CHCl_3$).

Determination of enantiomeric excess: ee: 88%. Method description: Chiralpack IH-3 150x4.6 mm, Particle size 5 μm, solvent system: *n*-heptane + 1%IPA + 1%EtOH; flow 1 mL/min, 25 °C. Peak area 94.0% (Rt = 14.7 min), 6.0% (Rt = 15.8 min).

Enantioenriched compound:

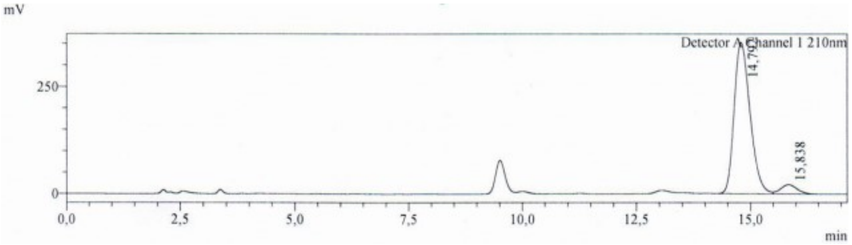

Detector A Channel 1 210nm			
Peak#	Ret. Time	Area	Area%
1	14,792	8599975	93,922
2	15,838	556542	6,078
Total		9156518	100,000

Racemic compound:

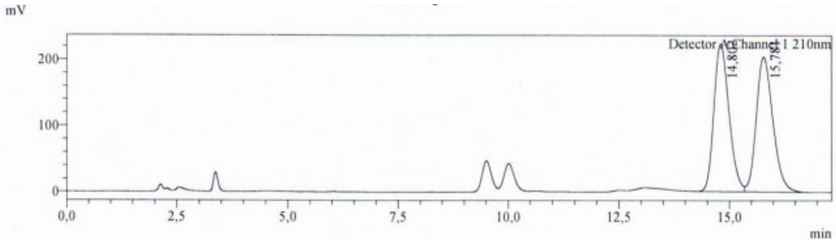

Detector A Channel 1 210nm			
Peak#	Ret. Time	Area	Area%
1	14,802	5332040	49,698
2	15,781	5396854	50,302
Total		10728894	100,000

(2*S*,4*S*,5*R*)-5-methyl-4-(naphthalen-2-yl)-2-(trifluoromethyl)tetrahydro-2*H*-pyran-2,6-diol (1e)

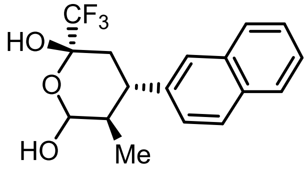

$C_{17}H_{17}F_3O_3$
MW: 326.11

Synthesized following General Procedure 2 on 5.00 mmol scale from **14e** and propionaldehyde (**S3b**). Purification by flash column chromatography (heptane/EtOAc = 7:3) gave the title compound in 28% yield (456.6 mg, 1.40 mmol) as a diastereomeric mixture in a 2.0:1.0 ratio.

^{1}H NMR (600 MHz, CDCl$_3$) δ (major diastereomer) 7.85 – 7.80 (m, 3H), 7.66 (s, 1H), 7.49 – 7.46 (m, 2H), 7.34 (dd, *J* = 8.5, 1.8 Hz, 1H), 5.12 (d, *J* = 3.2 Hz, 1H), 3.09 (td, *J* = 11.8, 4.8 Hz, 1H), 2.95 (s, 1H), 2.79 (d, *J* = 4.7 Hz, 1H), 2.13 – 2.04 (m, 2H), 1.94 – 1.87 (m, 1H), 0.88 (d, *J* = 6.6 Hz, 3H) ppm.

^{13}C NMR (151 MHz, CDCl$_3$) δ 139.2, 133.7, 132.8, 128.8, 127. 79 (d, *J* = 9.0 Hz), 126.8, 126.4, 125.9, 125.4, 122.39 (d, *J* = 285.2 Hz), 97.4, 95.58 (q, *J* = 33.0 Hz), 42.4, 35.0, 14.0 ppm.

^{19}F NMR (565 MHz, CDCl$_3$) δ −87.3, −87.4 ppm.

HRMS (ESI+): exact mass calculated for $[M+Na]^+$ ($C_{17}H_{17}O_3F_3Na$) required *m/z* 349.1027, found *m/z* 349.1011.

FT-IR (neat) ν_{max}: 3378, 3055, 2969, 2939, 1458, 1438, 1378, 1362, 1329, 1297, 1280, 1240, 1182, 1127, 1032, 1016, 970, 909, 857, 817, 734, 649, 477.

Determination of enantiomeric excess: ee: 89%. Method description: Chiralpak IC 250 x 4.6 mm, Particle size 5 μm, solvent system: *n*-heptane+0.1%IPA/IPA 98:2; flow 1 mL/min, 25 °C. Peak area 5.5% (Rt = 11.6 min), 95.5% (Rt = 15.4 min).

Enantioenriched compound:

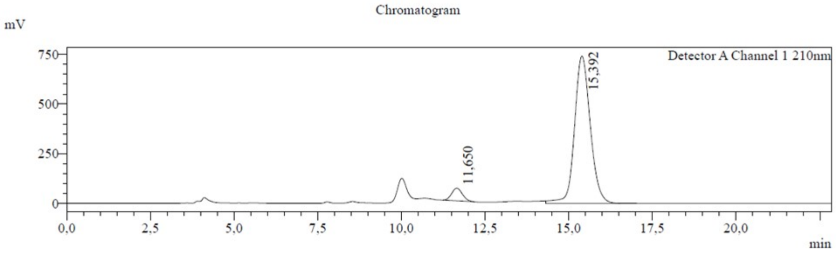

Detector A Channel 1 210nm

Peak#	Ret. Time	Area	Area%
1	11,650	1400924	5,425
2	15,392	24421470	94,575
Total		25822394	100,000

Racemic compound:

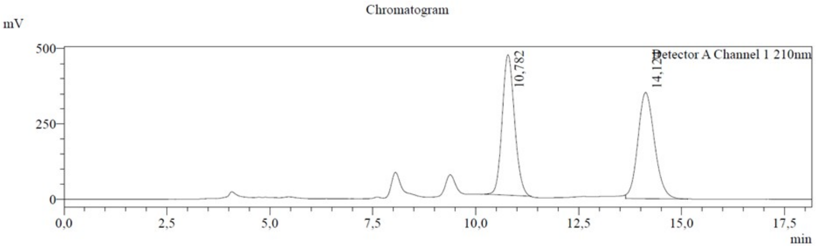

Detector A Channel 1 210nm

Peak#	Ret. Time	Area	Area%
1	10,782	9774709	49,510
2	14,124	9968276	50,490
Total		19742985	100,000

(2*S*,4*S*,5*R*)-4-(2-fluorophenyl)-5-methyl-2-(trifluoromethyl)tetrahydro-2*H*-pyran-2,6-diol (1f)

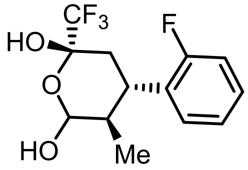

$C_{13}H_{14}F_4O_3$
MW: 294.09

Synthesized following General Procedure 2 on 3.00 mmol scale from **14f** and propionaldehyde (**S3b**). Purification by flash column chromatography (heptane/EtOAc = 7:3) gave the title compound in 16% yield (141.2 mg, 0.480 mmol) as a single diastereomer.

^{1}H NMR (600 MHz, $CDCl_3$) δ 7.23 (t, *J* = 10.2 Hz, 2H), 7.13 (t, *J* = 7.4 Hz, 1H), 7.07 – 7.03 (m, 1H), 5.10 (d, *J* = 8.8 Hz, 1H), 3.58 (s, 1H), 3.33 (td, *J* = 12.1, 4.3 Hz, 1H), 2.22 (s, 1H), 2.12 – 1.89 (m, 3H), 0.84 (d, *J* = 6.6 Hz, 3H) ppm.

^{13}C NMR (151 MHz, $CDCl_3$) δ 161.0 (d, *J* = 245.6 Hz), 128.6 (d, *J* = 4.0 Hz), 128.5, 128.4 (d, *J* = 8.2 Hz), 124.5 (d, *J* = 3.4 Hz), 122.3 (q, *J* = 285.2 Hz), 115.8 (d, *J* = 22.9 Hz), 99.0, 95.2 (q, *J* = 33.0 Hz), 39.0, 35.2, 33.3, 13.2 ppm.

^{19}F NMR (565 MHz, $CDCl_3$) δ −87.24 (s), −118.12 (s) ppm.

IR (neat) ν_{max}: 3333, 2952, 1173, 989, 768 cm^{-1}.

HRMS (ESI$^+$): exact mass calculated for $[M+Na]^+$ ($C_{13}H_{14}F_4O_3Na^+$) requires *m/z* 317.0771, found 317.0773 *m/z*.

$[\alpha]_D^{20}$ = −0.14 (c = 1.0, $CHCl_3$).

Determination of enantiomeric excess: ee: 94%. Method description: Chiralpak IC 250 x 4.6 mm, Particle size 5 μm, solvent system: *n*-heptane+0.1%IPA/IPA 98:2; flow 1 mL/min, 25 °C. Peak area 3.0% (Rt = 9.0 min), 97.0% (Rt = 11.2 min).

Enantioenriched compound:

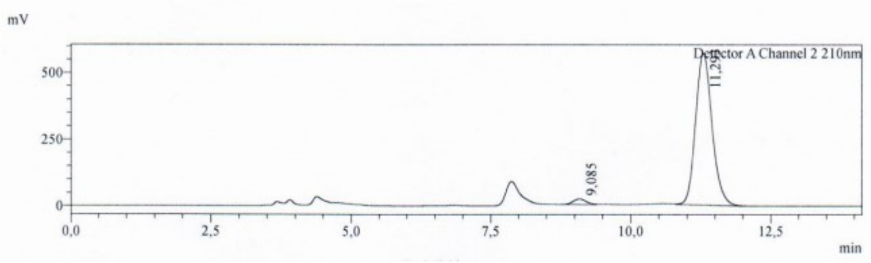

Detector A Channel 2 210nm			
Peak#	Ret. Time	Area	Area%
1	9,085	357513	2,962
2	11,293	11711505	97,038
Total		12069018	100,000

Racemic compound:

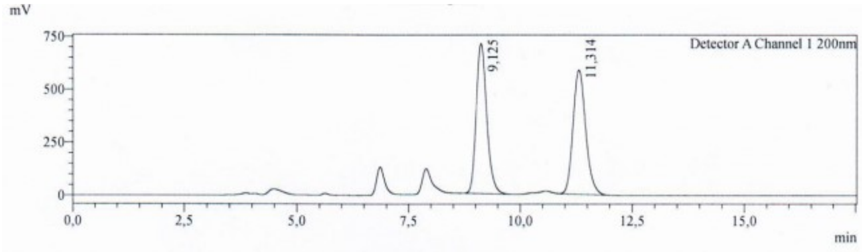

Detector A Channel 1 200nm			
Peak#	Ret. Time	Area	Area%
1	9,125	11730314	50,362
2	11,314	11561780	49,638
Total		23292095	100,000

(2*S*,4*S*,5*R*)-5-isopropyl-4-phenyl-2-(trifluoromethyl)tetrahydro-2*H*-pyran-2,6-diol (1g)

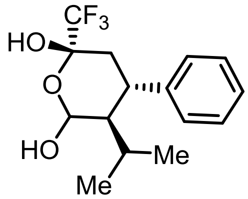

$C_{15}H_{19}F_3O_3$
MW: 304.30

Synthesized following General Procedure 2 on 10.0 mmol scale from **14a** and isovalerylaldehyde (**S3c**). Purification by flash column chromatography (heptane/EtOAc = 7:3) gave the title compound in 31% yield (943.3 mg, 3.10 mmol) as a single diastereomer.

^{1}H NMR (600 MHz, CDCl$_3$) δ 7.37 – 7.30 (m, 2H), 7.26 – 7.22 (m, 3H), 5.41 (d, *J* = 8.7 Hz, 1H), 3.17 (td, *J* = 11.5, 5.5 Hz, 1H), 3.10 (s, 1H), 1.99 (q, *J* = 8.8 Hz, 2H), 1.89 – 1.82 (m, 1H), 1.74 – 1.68 (m, 1H), 1.00 (d, *J* = 7.1 Hz, 3H), 0.81 (d, *J* = 7.2 Hz, 3H) ppm.

^{13}C NMR (151 MHz, CDCl$_3$) δ 142.9 (s), 128.8 (s), 127.7 (s), 126.9 (s), 122.4 (q, *J* = 285.5 Hz), 94.9 (s), 94.7 (q, *J* = 32.7 Hz), 48.7 (s), 38.8 (s), 36.0 (s), 26.7 (s), 22.3 (s), 16.0 (s) ppm.

^{19}F NMR (565 MHz, CDCl$_3$) δ –87.21 (s) ppm.

IR (neat) ν_{max}: 3258, 1151, 1007, 717 cm^{-1}.

HRMS (ESI$^+$): exact mass calculated for [M+Na]$^+$ ($C_{15}H_{19}F_3O_3Na^+$) requires *m/z* 327.1179, found 327.1177 *m/z*.

$[\alpha]_D^{20}$ = –0.855 (c = 1.0, CHCl$_3$).

Determination of enantiomeric excess: ee > 99.9%. Method description: Lux-Cellulose 1 250x4.6 mm, Particle size 5 μm, solvent system: *n*-heptane+0.1%IPA/IPA 95:5; flow 1 mL/min, 25 °C. Peak area 0.0% (Rt = 7.7 min), 100.0% (Rt = 16.6 min).

Enantiopure compound:

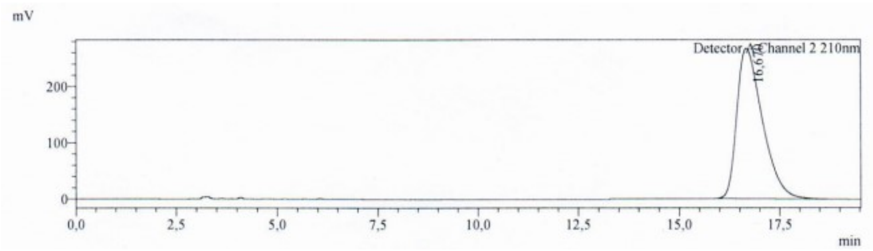

Detector A Channel 1 200nm

Peak#	Ret. Time	Area	Area%
1	16,667	10857384	100,000
Total		10857384	100,000

Racemic compound:

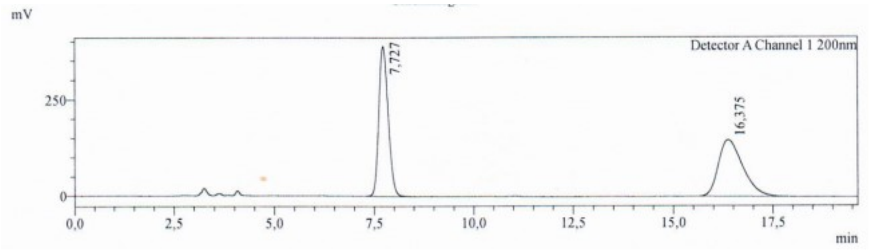

Detector A Channel 1 200nm

Peak#	Ret. Time	Area	Area%
1	7,727	6390316	50,298
2	16,375	6314720	49,702
Total		12705036	100,000

(2*S*,4*S*,5*R*,6*R*)-5-methyl-4-phenyl-2-(trifluoromethyl)tetrahydro-2*H*-pyran-6-*d*-2,6-diol (1h)

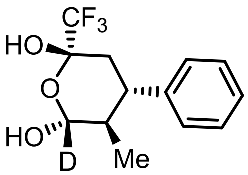

$C_{13}H_{14}DF_4O_3$
MW: 277.10

D = 88%

Synthesized following General Procedure 2 on 8.00 mmol scale from **14a** and 1d-propionaldehyde (**S3a**). Purification by flash column chromatography (heptane/EtOAc = 7:3) gave the title compound in 14% yield (310 mg, 1.12 mmol) as a diastereomeric mixture in a 2.3:1.0 ratio.

^{1}H NMR (600 MHz, $CDCl_3$) δ 7.37 – 7.31 (m, 2H), 7.28 – 7.24 (m, 1H), 7.25 – 7.18 (m, 2H), 5.39 (dd, *J* = 5.8, 3.4 Hz, 0.04H), 5.07 (t, *J* = 8.2 Hz, 0.08H), 4.16 (s, 0.3H), 3.59 (s, 0.3H), 3.17 (td, *J* = 12.4, 3.7 Hz, 0.3H), 3.11 (d, *J* = 2.2 Hz, 0.7H), 2.95 – 2.87 (m, 1.4H), 2.18 – 2.09 (m, 0.6H), 2.07 – 2.00 (m, 1H), 1.95 (td, *J* = 13.2, 2.3 Hz, 0.7H), 1.79 (dq, *J* = 12.9, 6.5 Hz, 0.7H), 0.85 (d, *J* = 6.6 Hz, 2.1H), 0.79 (d, *J* = 6.9 Hz, 0.9H) ppm.
^{13}C NMR (151 MHz, $CDCl_3$) δ 141.7, 141.6, 128.8, 128.8, 127.6, 127.5, 127.10, 127.01, 122.2 (q, *J* = 285.0 Hz), 122.1 (q, *J* = 285.0 Hz), 97.2, 97.1, 96.85 (t, *J* = 20.0 Hz), 95.34 (q, *J* = 31.7 Hz), 42.2, 42.2, 39.08, 36.2, 36.0, 34.9, 14.2, 13.7 ppm.
^{19}F NMR (565 MHz, $CDCl_3$) δ −87.22 (s), −87.40 (s) ppm.
IR (neat) ν_{max}: 3261, 1140, 999, 701 cm^{-1}.
HRMS (ESI$^+$): exact mass calculated for $[M+Na]^+$ ($C_{13}H_{14}DF_3O_3Na^+$) requires *m/z* 300.0928, found 300.0928 *m/z*.

3. Optimization of the Hydride Transfer reaction

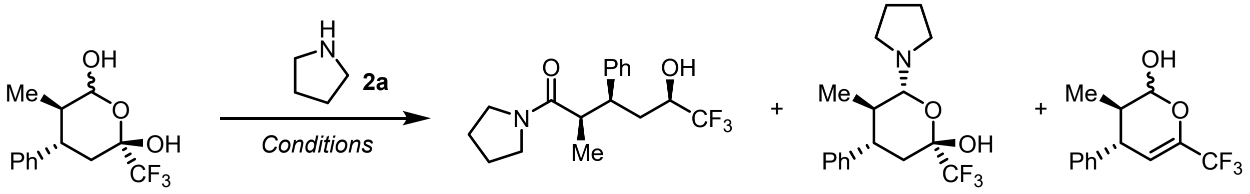

Procedure: A borosilicate glass vial containing a magnetic stirring bar was charged with hemiacetal **1a** (27.6 mg, 0.100 mmol, 1.00 equiv.), the given amount of solvent (according to the concentration stated in *Table S1*) and pyrrolidine (equiv. **2a**). The clear solution was stirred for 30min at 25 °C and was subsequently heated in an oil bath at the reported temperature (**T**). After the indicated reaction time, the reaction mixture was allowed to cool to 25 °C, and the volatiles were removed under reduced pressure. Finally, 1,3,5-Trimethoxybenzene was added as an internal standard and the NMR yields of the different components were determined.

(*Nota bene: the starting material **1a** was submitted as a diastereomeric mixture*).

Entry	eq. 2a	Additive	Solvent	Conc. [M]	Time (h)	T (°C)	Yield 3a	Yield 4a	Yield 5a
**1**	1.1	–	Toluene	0.04	24	25	–	100%	–
**2**	1.1	–	Toluene	0.04	24	70	60%	21%	–
**3**	1.1	–	Toluene	0.04	24	**100**	**82%**	8%	–
**4**	1.1	–	Toluene	**0.10**	24	100	**41%**	14%	13%
**5**	1.1	–	Toluene	**0.02**	24	100	**81%**	5%	–
**6**	1.1	–	Toluene	**0.01**	24	100	**81%**	6%	–
**7**	1.1	–	**DMSO**	0.04	24	100	**0%**	–	–
**8**	1.1	–	**DCE**	0.04	24	80	**22%**	28%	–
**9**	1.1	–	**Trifluoroethanol**	0.04	24	100	**0%**	–	–
**10**	1.1	**BF_3•OEt_2 10%**	Toluene	0.04	24	100	**0%**	–	17%
**11**	1.1	**$Sc(OTf)_3$ 10%**	Toluene	0.04	24	**25**	**0%**	90%	–
**12**	1.1	**$Sc(OTf)_3$ 10%**	Toluene	0.04	24	100	**30%**	–	–
**13**	1.1	**$Yb(OTf)_3$ 10%**	Toluene	0.04	24	100	**0%**	–	–
**14**	1.1	**3 Å MS**	Toluene	0.04	24	100	**53%**	27%	–
**15**	1.2	–	Toluene	0.04	24	100	**83%**	–	–

**16**	**2.0**	–	Toluene	0.04	24	100	**86%**	–	–
**17**	**3.0**	–	Toluene	0.04	24	100	**85%**	–	–
**18**	2.0	–	Toluene	0.04	**48**	100	**89%**	–	–

Table S1: Optimization of the reaction.

Preparation of Hydride Transfer CF_3-alcohol products

3.1 General Procedure 3: Preparation of 1,5-carboxamido-trifluoromethylcarbinols

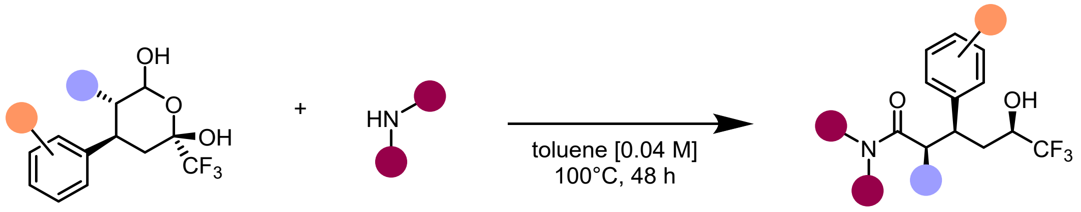

1a-g **2a-g** **3a-o**

General procedure 3: In a borosilicate glass vial containing a magnetic stirring bar, the hemiacetal **1a-g** (1.00 equiv.) was dissolved in toluene (0.04 M). To this stirring solution, amine **2a-g** was added (2.00 equiv.) at 25 °C and the mixture was stirred for 30min. Subsequently, the vial was transferred to an oil bath and was heated to 100 °C for 48h. After this time, the reaction mixture was allowed to cool to 25 °C. The volatiles were removed under reduced pressure and the resulting crude product was purified by flash column chromatography on silica gel (heptane/EtOAc = 7:3) to obtain the desired product.

(*Nota bene: when presented as a mixture, the starting material **1a-g** was submitted as a diastereomeric mixture*).

3.2 Characterization of the CF_3-alcohol products

(2*R*,3*S*,5*S*)-6,6,6-trifluoro-5-hydroxy-2-methyl-3-phenyl-1-(pyrrolidin-1-yl)hexan-1-one (3a)

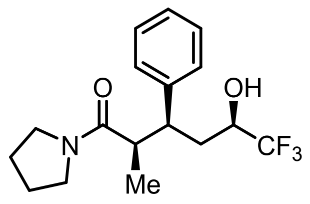

$C_{17}H_{22}F_3NO_2$
MW: 329.16

Synthesized following General Procedure 3 on 0.150 mmol scale from **1a** and pyrrolidine **2a**. Purification by flash column chromatography (heptane/EtOAc = 7:3) gave the title compound in 89% yield (43.8 mg, 0.134 mmol).

^{1}H NMR (600 MHz, CDCl$_3$) δ 7.33 (dd, *J* = 10.5, 4.6 Hz, 2H), 7.25 – 7.22 (m, 1H), 7.22 – 7.19 (m, 2H), 3.82 (s, 1H), 3.59 – 3.44 (m, 5H), 3.25 (dt, *J* = 10.3, 6.8 Hz, 1H), 2.75 (dq, *J* = 10.4, 6.9 Hz, 1H), 2.07 – 1.87 (m, 6H), 0.86 (d, *J* = 6.9 Hz, 3H) ppm.

^{13}C NMR (151 MHz, CDCl$_3$) 175.1, 143.0, 128.8, 128.1, 126.9, 125.1 (q, *J* = 281.9 Hz), 68.8 (q, *J* = 30.7 Hz), 46.8, 46.2, 44.0, 43.9, 36.5, 26.1, 24.3, 17.1 ppm.

^{19}F NMR (565 MHz, CDCl$_3$) δ −79.11 (d, *J* = 7.6 Hz) ppm.

IR (neat) ν_{max}: 3309, 2939, 1613, 1439, 1142, 1107, 703 cm^{-1}.

HRMS (ESI$^+$): exact mass calculated for $[M+Na]^+$ ($C_{17}H_{22}F_3NO_2Na^+$) requires *m/z* 352.1495, found 352.1494 *m/z*.

$[\alpha]_D^{20}$ = −0.74 (c = 1.0, $CHCl_3$).

Determination of enantiomeric excess: ee: 90%. Method description: Method description: Chiralpak IC 250 x 4.6 mm, particle size 5 μm, solvent system: *n*-heptane+0.1%IPA/IPA 92:8; flow 1 mL/min, 25 °C. Peak area 5.0% (Rt = 8.6 min), 95.0% (Rt = 9.8 min).

Enantioenriched compound:

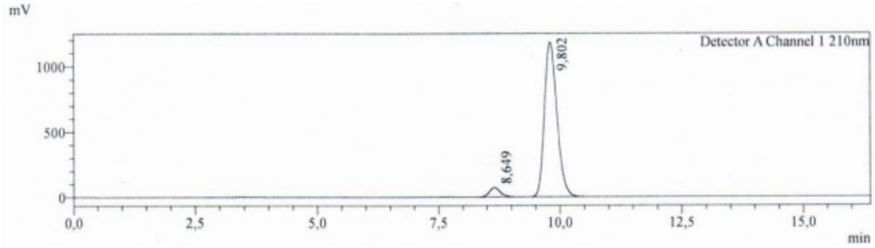

Detector A Channel 1 210nm			
Peak#	Ret. Time	Area	Area%
1	8,649	1126180	5,011
2	9,802	21345863	94,989
Total		22472043	100,000

Racemic compound:

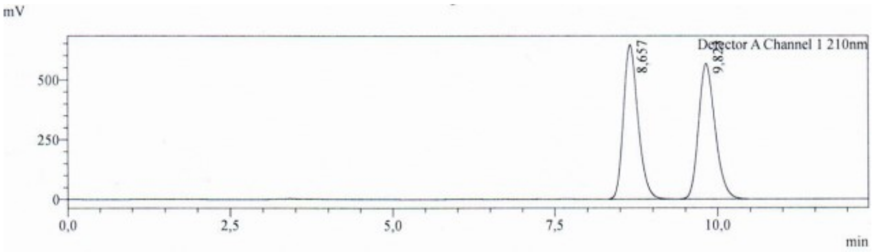

Detector A Channel 1 210nm			
Peak#	Ret. Time	Area	Area%
1	8,657	10136946	49,981
2	9,823	10144588	50,019
Total		20281534	100,000

Scale-up of the synthesis of 3a

In a round-bottom flask containing a magnetic stirring bar, the hemiacetal **1a** (1.00 equiv.) was dissolved in toluene (0.04 M). To this stirring solution, amine **2a** was added (2.00 equiv.) at 25 °C and the mixture was stirred for 30min. Subsequently, the vial was transferred to an oil bath and was heated to 100 °C for 48h. After this time, the reaction mixture was allowed to cool to 25 °C. The volatiles were removed under reduced pressure and the resulting crude product was purified by flash column chromatography on silica gel (heptane/EtOAc = 7:3) to obtain the desired product.

Scale 0.5 mmol: Synthesized following the previous procedure on 0.500 mmol scale from **1a** and pyrrolidine **2a**. Purification by flash column chromatography (heptane/EtOAc = 7:3) gave the title compound in 84% yield (138.4 mg, 0.420 mmol).

Scale 2.0 mmol: Synthesized following the previous procedure on 0.500 mmol scale from **1a** and pyrrolidine **2a**. Purification by flash column chromatography (heptane/EtOAc = 7:3) gave the title compound in 80% yield (526.7 mg, 1.60 mmol).

(2*R*,3*S*,5*S*)-6,6,6-trifluoro-5-hydroxy-2-methyl-3-phenyl-1-(pyrrolidin-1-yl)hexan-1-one (*ent*-3a)

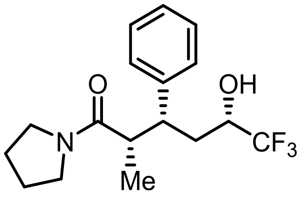

$C_{17}H_{22}F_3NO_2$
MW: 329.16

Synthesized following General Procedure 3 on 0.150 mmol scale from ***ent*-1a** and pyrrolidine **2a**. Purification by flash column chromatography (heptane/EtOAc = 7:3) gave the title compound in 89% yield (43.9 mg, 0.134 mmol).

IR (neat) ν_{max}: 3309, 2939, 1613, 1439, 1142, 1107, 703 cm^{-1}.

$[\alpha]_D^{20}$ = 0.74 (c = 1.0, $CHCl_3$).

Determination of enantiomeric excess: ee: 90%. Method description: Chiralpak IC 250 x 4.6 mm, particle size 5 μm, solvent system: *n*-heptane+0.1%IPA/IPA 92:8; flow 1 mL/min, 25 °C. Peak area 95.0% (Rt = 8.6 min), 5.0% (Rt = 9.8 min).

Enantioenriched compound:

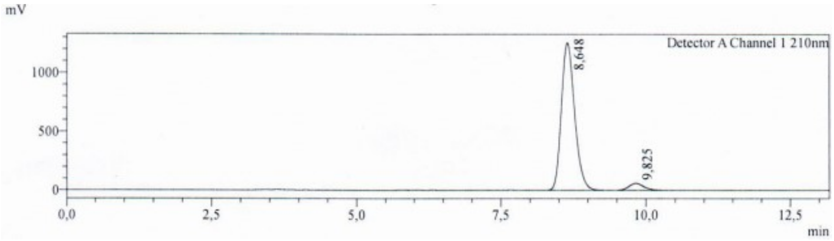

Detector A Channel 1 210nm

Peak#	Ret. Time	Area	Area%
1	8,648	19962041	94,920
2	9,825	1068304	5,080
Total		21030345	100,000

Racemic compound:

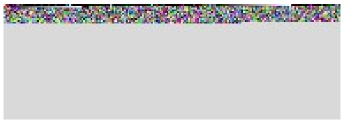

(2*R*,3*S*,5*S*)-6,6,6-trifluoro-5-hydroxy-2-methyl-3-phenyl-1-(piperidin-1-yl)hexan-1-one (3b)

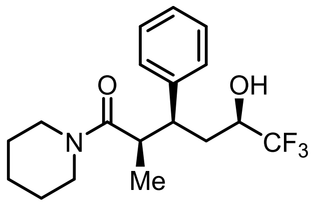

$C_{18}H_{24}F_3NO_2$
MW: 343.18

Synthesized following General Procedure 3 on 0.150 mmol scale from **1a** and piperidine **2b**. Purification by flash column chromatography (heptane/EtOAc = 7:3) gave the title compound in 67% yield (34.5 mg, 0.101 mmol).

^{1}H NMR (600 MHz, $CDCl_3$) δ 7.34 – 7.31 (m, 2H), 7.25 – 7.19 (m, 3H), 3.89 – 3.81 (m, 1H), 3.76 – 3.70 (m, 1H), 3.68 (s, 1H), 3.61 – 3.52 (m, 2H), 3.50 – 3.44 (m, 1H), 3.31 (dt, *J* = 10.2, 6.7 Hz, 1H), 2.93 (dq, *J* = 10.2, 7.0 Hz, 1H), 1.96 (t, *J* = 6.6 Hz, 2H), 1.76 – 1.51 (m, 6H), 0.82 (d, *J* = 7.0 Hz, 3H) ppm.
^{13}C NMR (151 MHz, $CDCl_3$) δ 174.9, 143.3, 128.9, 128.4, 127.0, 125.3 (q, *J* = 282.4 Hz), 68.97 (q, *J* = 30.7 Hz), 47.0, 43.9, 43.5, 41.5, 37.0, 27.0, 25.8, 24.7, 17.7 ppm.

^{19}F NMR (565 MHz, $CDCl_3$) δ −79.04 (d, *J* = 6.2 Hz) ppm.
IR (neat) ν_{max}: 3311, 2932, 16139 1428, 1151, 1100, 718 cm^{-1}.

HRMS (ESI$^+$): exact mass calculated for $[M+Na]^+$ ($C_{18}H_{24}F_3NO_2Na^+$) requires *m/z* 366.1651, found 366.1673 *m/z*.

$[\alpha]_D^{20}$ = −0.46 (c = 1.0, $CHCl_3$).

Determination of enantiomeric excess: ee: 95%. Method description: Chiralpak IC 250 x 4.6 mm, particle size 5 μm, solvent system: *n*-heptane+0.1%IPA/IPA 92:8; flow 1 mL/min, 25 °C. Peak area 2.7% (Rt = 10.9 min), 97.3% (Rt = 12.1 min).

Enantioenriched compound:

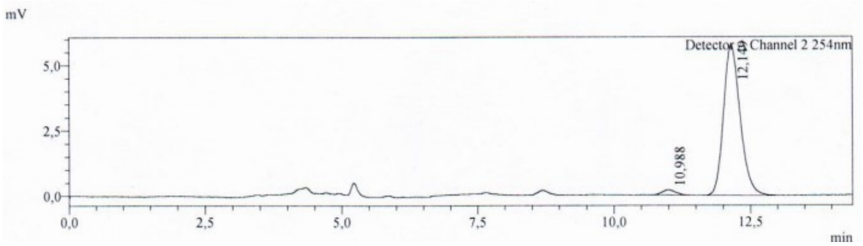

Detector A Channel 2 254nm

Peak#	Ret. Time	Area	Area%
1	10,988	3470	2,741
2	12,149	123096	97,259
Total		126566	100,000

Racemic compound:

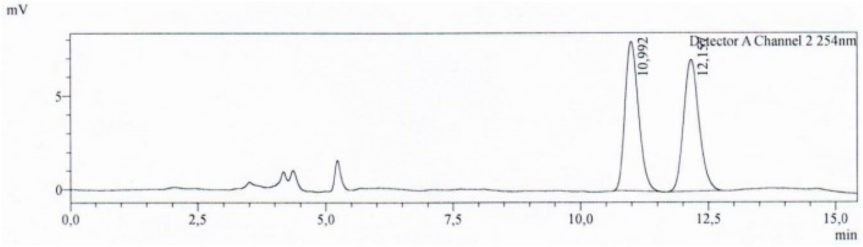

Detector A Channel 2 254nm

Peak#	Ret. Time	Area	Area%
1	10,992	146886	49,616
2	12,157	149159	50,384
Total		296045	100,000

(2*R*,3*S*,5*S*)-1-(azepan-1-yl)-6,6,6-trifluoro-5-hydroxy-2-methyl-3-phenylhexan-1-one (3c)

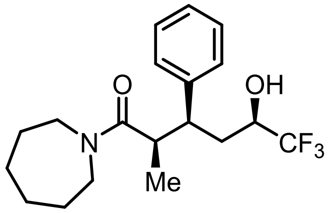

$C_{19}H_{26}F_3NO_2$
MW: 357.19

Synthesized following General Procedure 3 on 0.150 mmol scale from **1a** and azepane **2c**. Purification by flash column chromatography (heptane/EtOAc = 7:3) gave the title compound in 65% yield (34.8 mg, 0.098 mmol).

^{1}H NMR (700 MHz, $CDCl_3$) δ 7.32 (t, *J* = 7.6 Hz, 2H), 7.25 – 7.22 (m, 1H), 7.22 – 7.19 (m, 2H), 3.92 – 3.83 (m, 1H), 3.82 – 3.78 (m, 1H), 3.68 (s, 1H), 3.62 (dt, *J* = 14.5, 5.5 Hz, 1H), 3.43 (ddd, *J* = 22.6, 10.7, 6.4 Hz, 1H), 3.37 (ddd, *J* = 13.6, 7.3, 4.7 Hz, 1H), 3.29 (dt, *J* = 10.3, 6.7 Hz, 1H), 2.87 (dq, *J* = 10.4, 6.9 Hz, 1H), 1.99 – 1.91 (m, 2H), 1.89 – 1.80 (m, 1H), 1.78 – 1.56 (m, 6H), 1.56 – 1.48 (m, 1H), 0.85 (d, *J* = 6.9 Hz, 3H) ppm.
^{13}C NMR (176 MHz, $CDCl_3$) δ 176.3, 143.2, 128.8, 128.2, 126.9, 125.1 (q, *J* = 281.5 Hz), 68.8 (q, *J* = 30.7 Hz), 47.9, 46.5, 44.0, 41.9, 36.8, 29.4, 27.6, 26.8 (2C), 17.8 ppm.

^{19}F NMR (659 MHz, $CDCl_3$) δ −79.13 (s) ppm.
IR (neat) ν_{max}: 3321, 2918, 1632, 1148, 1101, 716 cm^{-1}.

HRMS (ESI$^+$): exact mass calculated for $[M+Na]^+$ ($C_{19}H_{26}F_3NO_2Na^+$) requires *m/z* 380.1808, found 380.1808 *m/z*.

$[\alpha]_D^{20}$ = −0.54 (c = 1.0, $CHCl_3$).

Determination of enantiomeric excess: ee: 76%. Method description: Chiralpak IC 250 x 4.6 mm, particle size 5 μm, solvent system: *n*-heptane+0.1%IPA/IPA 92:8; flow 1 mL/min, 25 °C. Peak area 12.0% (Rt = 10.3 min), 88.0% (Rt = 11.8 min).

Enantioenriched compound:

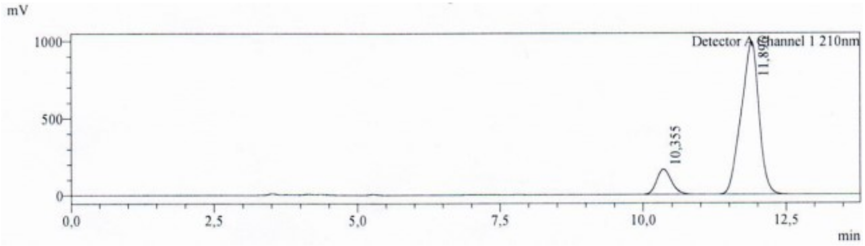

Detector A Channel 1 210nm			
Peak#	Ret. Time	Area	Area%
1	10,355	2977281	12,137
2	11,896	21553871	87,863
Total		24531152	100,000

Racemic compound:

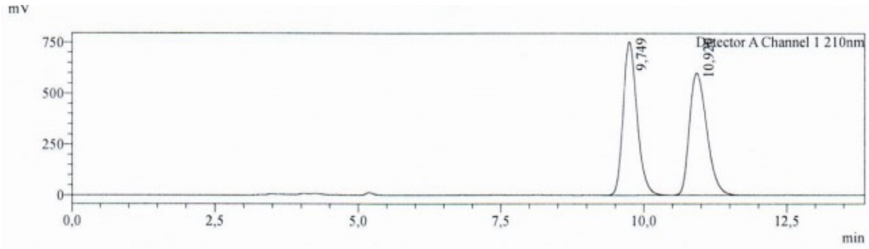

Detector A Channel 1 210nm			
Peak#	Ret. Time	Area	Area%
1	9,749	12790965	50,020
2	10,920	12780779	49,980
Total		25571744	100,000

(2*R*,3*S*,5*S*)-6,6,6-trifluoro-5-hydroxy-*N*,2-dimethyl-N-phenethyl-3-phenylhexanamide (3d)

$C_{22}H_{26}F_3NO_2$
MW: 393.19

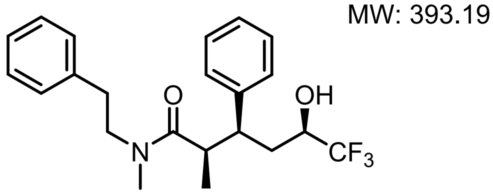

Synthesized following General Procedure 3 on 0.150 mmol scale from **1a** and *N*-methyl-2-phenylethan-1-amine **2d**. Purification by flash column chromatography (heptane/EtOAc = 7:3) to afford the title compound in 42% yield (24.8 mg, 0.063 mmol). The reported NMR data account for the observation of a rotameric mixture.

^{1}H NMR (600 MHz, $CDCl_3$) δ 7.38 (t, *J* = 7.5 Hz, 1H), 7.35 – 7.27 (m, 3.5H), 7.25 – 7.18 (m, 4.5H), 7.08 (d, *J* = 7.2 Hz, 1H), 3.85 – 3.77 (m, 0.55H), 3.74 – 3.64 (m, 2H), 3.51 (dt, *J* = 14.6, 6.3 Hz, 0.45H), 3.42 (d, *J* = 4.8 Hz, 0.55H), 3.29 – 3.24 (m, 1H), 3.11 (dt, *J* = 10.4, 6.6 Hz, 0.45H), 3.01 (s, 1.35H), 2.97 (s, 1.65H), 2.94 – 2.83 (m, 2.6H), 2.61 (dq, *J* = 10.4, 6.9 Hz, 0.45H), 1.96 – 1.88 (m, 1H), 1.64 – 1.56 (m, 2H), 0.78 (d, *J* = 6.9 Hz, 1.65H), 0.73 (d, *J* = 6.9 Hz, 1.35H).

^{13}C NMR (151 MHz, $CDCl_3$) δ 176.4, 176.4, 143.1, 142.8, 138.7, 137.9, 129.0, 128.8 (2x), 128.8, 128.8, 128.6, 128.2, 128.1, 127.1, 126.9, 126.9, 126.5, 125.1 (q, *J* = 281. Hz), 125.1 (q, *J* = 281.7 Hz), 69.1 (q, *J* = 61.4), 68.9 (q, *J* = 61.3 Hz), 51.4, 50.2, 44.2, 44.0, 41.9, 41.9, 36.6, 36.2, 35.9, 35.0, 33.9, 33.6, 17.5, 17.1.

^{19}F NMR (565 MHz, $CDCl_3$) δ –78.92 (d, *J* = 6.6 Hz), –78.98 (d, *J* = 6.2 Hz).

IR (neat) ν: 3311, 2941, 1618, 1427, 1141, 1103, 707 cm^{-1}.

HRMS (ESI$^+$): exact mass calculated for $[M+Na]^+$ ($C_{22}H_{26}F_3NO_2Na^+$) requires m/z 416.1808, found 416.1806 m/z.

$[\alpha]_D^{20}$ = –0.299 (c = 1.0, $CHCl_3$).

Determination of enantiomeric excess: ee: 86%. Method description: Lux-Cellulose 1 250x4.6 mm, Particle size 5 μm, solvent system: *n*-heptane/EtOH 97:3; flow 1 mL/min, 25 °C. Peak area 7.0% (Rt = 16.6 min), 93.0% (Rt = 17.9 min).

Enantioenriched compound:

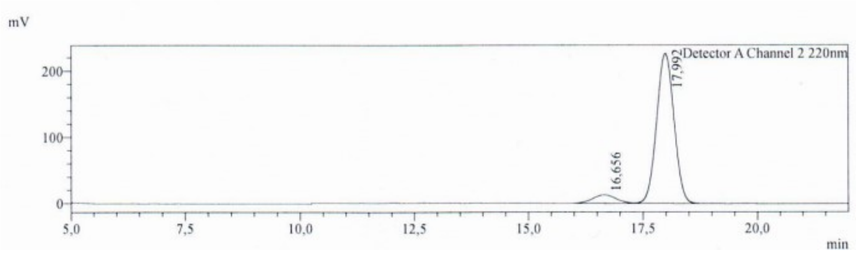

Detector A Channel 2 220nm

Peak#	Ret. Time	Area	Area%
1	16,656	452160	7,034
2	17,992	5975829	92,966
Total		6427989	100,000

Racemic compound:

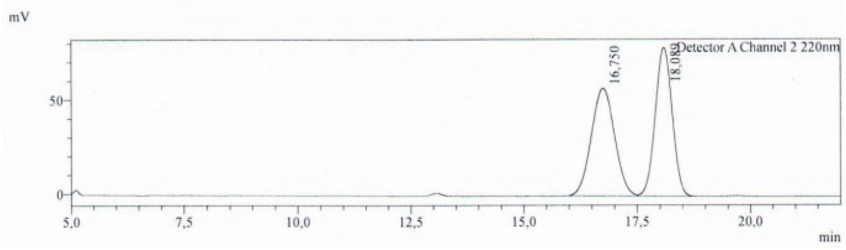

Detector A Channel 2 220nm

Peak#	Ret. Time	Area	Area%
1	16,750	2049792	49,894
2	18,089	2058479	50,106
Total		4108271	100,000

(2*R*,3*S*,5*S*)-*N*,*N*-diethyl-6,6,6-trifluoro-5-hydroxy-2-methyl-3-phenylhexanamide (3e)

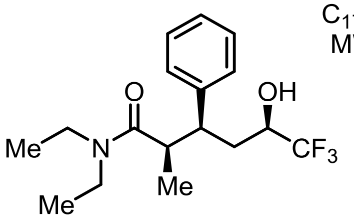

Synthesized following General Procedure 3 on 0.150 mmol scale from **1a** and *N*,*N*-diethylamine **2e**. Purification by flash column chromatography (heptane/EtOAc = 7:3) to afford the title compound in 60% yield (29.8 mg, 0.090 mmol).

^{1}H NMR (600 MHz, $CDCl_3$) δ 7.33 (t, *J* = 7.5 Hz, 2H), 7.24 (t, *J* = 7.4 Hz, 1H), 7.21 (d, *J* = 7.3 Hz, 2H), 3.87 – 3.79 (m, 1H), 3.61 (s, 1H), 3.47 – 3.40 (m, 3H), 3.35 (dq, *J* = 14.5, 7.1 Hz, 1H), 3.28 (dt, *J* = 10.2, 6.9 Hz, 1H), 2.81 (dq, *J* = 10.4, 6.9 Hz, 1H), 1.95 (t, *J* = 6.9 Hz, 2H), 1.27 (t, *J* = 7.2 Hz, 3H), 1.15 (t, *J* = 7.1 Hz, 3H), 0.85 (d, *J* = 6.8 Hz, 3H) ppm.

^{13}C NMR (151 MHz, $CDCl_3$) δ 176.32, 143.4, 129.1, 128.4, 127.2, 126.2 (q, *J* = 256.7 Hz), 69.0 (q, *J* = 30.7 Hz), 44.3, 42.6, 42.0, 41.4, 37.0, 18.2, 15.2, 13.0 ppm.

^{19}F NMR (565 MHz, $CDCl_3$) δ −79.17 (d, *J* = 6.3 Hz) ppm.

IR (neat) ν_{max}: 3312, 2926, 1615, 1432, 1148, 1106, 708 cm^{-1}.

HRMS (ESI$^+$): exact mass calculated for $[M+Na]^+$ ($C_{17}H_{24}F_3NO_2Na^+$) requires *m*/*z* 354.1651, found 354.1645 *m*/*z*.

$[\alpha]_D^{20}$ = −0.41 (c = 1.0, $CHCl_3$).

Determination of enantiomeric excess: ee: 77%. Method description: Chiralpak IC 250 x 4.6 mm, particle size 5 μm, solvent system: (*n*-heptane+0.1%IPA)/MTBE/IPA 88:10:2; flow 1 mL/min, 25 °C. Peak area 11.5% (Rt = 9.9 min), 88.5% (Rt = 10.8 min).

Enantioenriched compound:

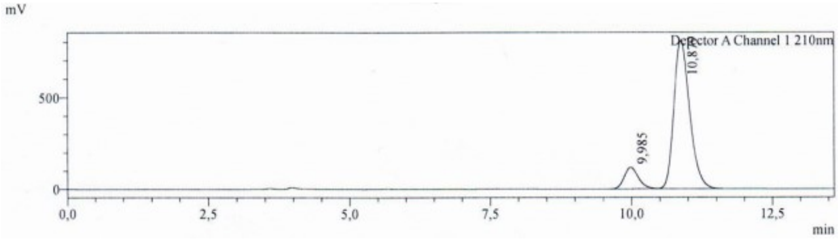

Detector A Channel 1 210nm			
Peak#	Ret. Time	Area	Area%
1	9,985	2104221	11,685
2	10,879	15903254	88,315
Total		18007475	100,000

Racemic compound:

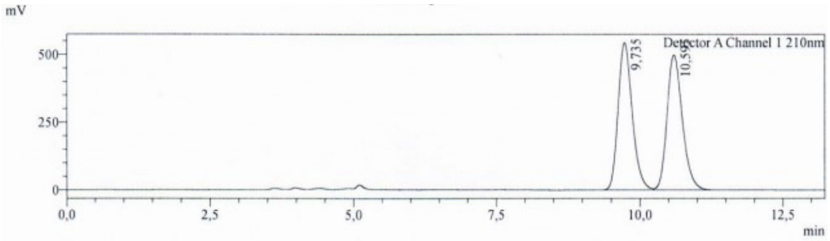

Detector A Channel 1 210nm			
Peak#	Ret. Time	Area	Area%
1	9,735	9309230	49,877
2	10,595	9354983	50,123
Total		18664213	100,000

(2*R*,3*S*,5*R*)-*N*,*N*-diallyl-6,6,6-trifluoro-5-hydroxy-2-methyl-3-phenylhexanamide (3f)

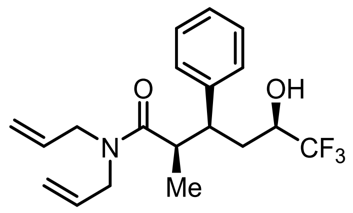

$C_{19}H_{24}F_3NO_2$
MW: 355.16

Synthesized following General Procedure 3 on 0.150 mmol scale from **1a** and *N*,*N*-diallylamine **2f**. Purification by flash column chromatography (heptane/EtOAc = 7:3) to afford the title compound in 42% yield (22.4 mg, 0.063 mmol).

^{1}H NMR (700 MHz, CDCl$_3$) δ 7.33 (t, *J* = 7.6 Hz, 2H), 7.24 (t, *J* = 7.4 Hz, 1H), 7.18 (d, *J* = 7.1 Hz, 2H), 5.85 (ddt, *J* = 17.0, 10.2, 5.1 Hz, 1H), 5.78 (ddt, *J* = 16.6, 10.2, 6.1 Hz, 1H), 5.31 (dd, *J* = 10.3, 1.0 Hz, 1H), 5.24 (dd, *J* = 17.2, 0.9 Hz, 1H), 5.20 (dd, *J* = 10.2, 1.1 Hz, 1H), 5.17 (dd, *J* = 17.1, 1.4 Hz, 1H), 4.16 (dd, *J* = 15.0, 5.7 Hz, 1H), 4.00 (dd, *J* = 17.5, 5.1 Hz, 1H), 3.97 – 3.89 (m, 2H), 3.84 (m, 1H), 3.25 (dt, *J* = 10.3, 6.8 Hz, 1H), 3.14 (d, *J* = 4.0 Hz, 1H), 2.84 (dq, *J* = 10.3, 6.9 Hz, 1H), 1.98 (ddd, *J* = 14.4, 7.1, 3.9 Hz, 1H), 1.94 – 1.88 (m, 1H), 0.86 (d, *J* = 6.9 Hz, 3H) ppm.

^{13}C NMR (176 MHz, CDCl$_3$) δ 176.9, 143.0, 133.2, 132.7, 129.0, 128.3, 127.2, 125.2 (q, *J* = 281.4 Hz), 118.1, 117.5, 69.2 (q, *J* = 30.8 Hz), 49.7, 48.8, 44.6, 42.0, 36.5, 18.1 ppm.

^{19}F NMR (659 MHz, CDCl$_3$) δ −79.25 (s) ppm.

IR (neat) ν_{max}: 3345, 2962, 2929, 2875, 2856, 1618, 1495, 1454, 1442, 1416, 1376, 1342, 1315, 1273, 1245, 1164, 1128, 1075, 1058, 1033, 1018, 991, 950, 925 cm^{-1}.

HRMS (ESI$^+$): exact mass calculated for $[M+Na]^+$ ($C_{19}H_{24}O_2F_3NNa$) required *m/z* 378.1657, found *m/z* 378.1656.

$[\alpha]_D^{20}$ = −0.39 (c = 1.0, CHCl$_3$).

Determination of enantiomeric excess: ee: 88%. Method description: Chiralpak IC 250 x 4.6 mm, particle size 5 μm, solvent system: *n*-heptane+0.1%IPA/MTBE 80:20; flow 1 mL/min, 25 °C. Peak area 5.8% (Rt = 25.0 min), 94.2% (Rt = 28.4 min).

Enantioenriched compound:

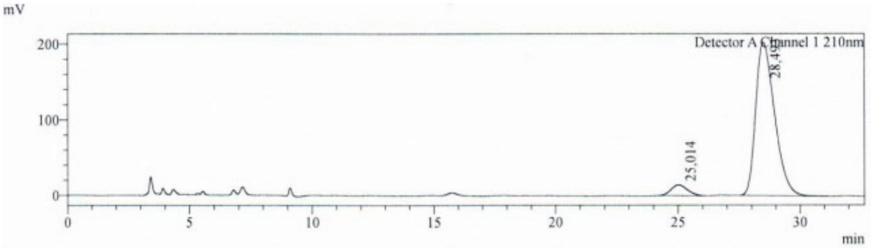

Detector A Channel 1 210nm

Peak#	Ret. Time	Area	Area%
1	25,014	678223	5,804
2	28,493	11006548	94,196
Total		11684771	100,000

Racemic compound:

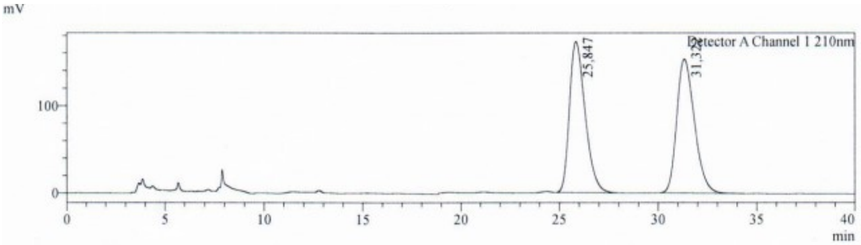

Detector A Channel 1 210nm

Peak#	Ret. Time	Area	Area%
1	25,847	9551485	49,885
2	31,322	9595619	50,115
Total		19147104	100,000

benzyl 4-((2*R*,3*S*,5*R*)-6,6,6-trifluoro-5-hydroxy-2-methyl-3-phenylhexanoyl)piperazine-1-carboxylate (3g)

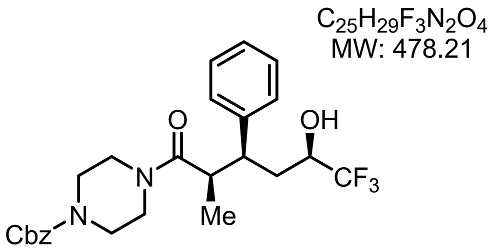

Synthesized following General Procedure 3 on 0.150 mmol scale from **1a** and 1-Cbz-piperazine **2g**. Purification by flash column chromatography (heptane/EtOAc = 7:3) to afford the title compound in 44% yield (31.6 mg, 0.066 mmol).

^{1}H NMR (700 MHz, $CDCl_3$) δ 7.41 – 7.31 (m, 7H), 7.26 – 7.24 (m, 1H), 7.20 (d, *J* = 7.4 Hz, 2H), 5.16 (s, 2H), 3.85 – 3.80 (m, 1H), 3.68 (bd, *J* = 14.3 Hz, 2H), 3.61 – 3.46 (m, 6H), 3.27 (dt, *J* = 9.8, 6.6 Hz, 1H), 2.95 (b, 2H), 2.03 – 1.92 (m, 2H), 0.85 (d, *J* = 6.9 Hz, 3H) ppm.

^{13}C NMR (176 MHz, $CDCl_3$) δ 175.03, 155.3,142.4, 136.2, 129.2, 129.0, 128.7, 128.6, 128.4, 128.3, 128.24, 128.23, 128.21, 127.3, 125.2 (q, *J* = 281.8 Hz, 69.3 (q, *J* = 30.8 Hz), 67.7, 45.6, 44.6, 44.0, 41.9, 41.5, 35.9, 17.5 ppm.

^{19}F NMR (659 MHz, $CDCl_3$) δ −79.2 (s) ppm.

IR (neat) ν_{max}: 3375, 2930, 2867, 1701, 1621, 1454, 1229, 1122, 700 cm^{-1}.

HMRS (ESI+): exact mass calculated for $[M+Na]^+$ ($C_{25}H_{29}O_4F_3N_2Na$) required *m/z* 501.1977, found *m/z* 501.1975.

$[\alpha]_D^{20}$ = −0.48 (c = 1.0, $CHCl_3$).

Determination of enantiomeric excess: ee: 86%. Method description: Chiralpak IC 250 x 4.6 mm, particle size 5 μm, solvent system: *n*-heptane+0.1%IPA/MTBE 80:20; flow 1 mL/min, 25 °C. Peak area 92.9% (Rt = 14.3 min), 7.1% (Rt = 37.4 min).

Enantioenriched compound:

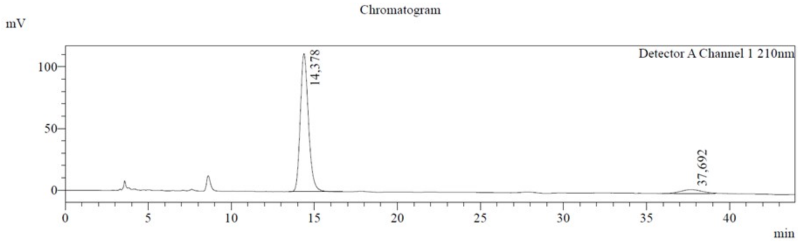

Detector A Channel 1 210nm

Peak#	Ret. Time	Area	Area%
1	14,378	3812538	92,849
2	37,692	293635	7,151
Total		4106173	100,000

Racemic compound:

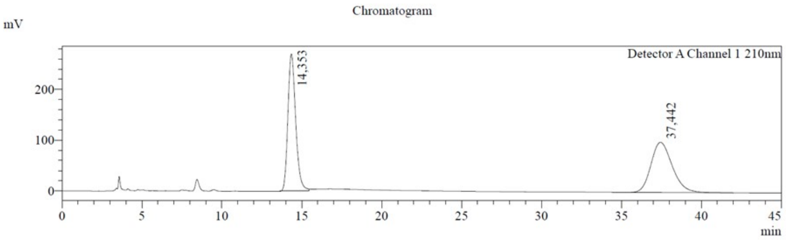

Detector A Channel 1 210nm

Peak#	Ret. Time	Area	Area%
1	14,353	9132498	49,860
2	37,442	9183678	50,140
Total		18316175	100,000

(2*R*,3*S*,5*S*)-6,6,6-trifluoro-5-hydroxy-2-methyl-1-morpholino-3-phenylhexan-1-one (3h)

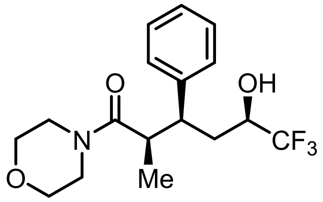

$C_{17}H_{22}F_3NO_3$
MW: 344.16

Synthesized following General Procedure 3 on 0.150 mmol scale from **1a** and morpholine **2h**. Purification by flash column chromatography (heptane/EtOAc = 7:3) to afford the title compound in 54% yield (27.9 mg, 0.081 mmol).

^{1}H NMR (700 MHz, $CDCl_3$) δ 7.34 – 7.31 (m, 2H), 7.27 – 7.23 (m, 1H), 7.22 – 7.19 (m, 2H), 3.82 (t, *J* = 17.2 Hz, 1H), 3.70 – 3.67 (m, 6H), 3.62 – 3.56 (m, 1H), 3.53 (ddd, *J* = 13.3, 5.9, 3.9 Hz, 1H), 3.27 (dt, *J* = 10.0, 6.6 Hz, 1H), 3.17 (s, 1H), 2.92 (dq, *J* = 10.1, 6.9 Hz, 1H), 2.04 – 1.93 (m, 2H), 0.85 (d, *J* = 6.9 Hz, 3H) ppm.
^{13}C NMR (176 MHz, $CDCl_3$) δ 174.8, 142.4, 128.9, 128.2, 127.1, 125.1 (q, *J* = 281.5 Hz), 69.1 (q, *J* = 30.7 Hz), 66.9, 66.7, 46.2, 44.3, 42.4, 41.1, 36.0, 17.3 ppm.

^{19}F NMR (659 MHz, $CDCl_3$) δ −79.15 (s) ppm.
IR (neat) ν_{max}: 3299, 2918, 1618, 1435, 1171, 1113 cm^{-1}.

HRMS (ESI$^+$): exact mass calculated for $[M+Na]^+$ ($C_{17}H_{22}F_3NO_3Na^+$) requires *m/z* 368.1444, found 368.1442 *m/z*.

$[\alpha]_D^{20}$ = −0.48 (c = 1.0, $CHCl_3$).

Determination of enantiomeric excess: ee: 95%. Method description: Chiralpak IC 250 x 4.6 mm, particle size 5 μm, solvent system: *n*-heptane+0.1%IPA/IPA 92:8; flow 1 mL/min, 25 °C. Peak area 97.4% (Rt = 13.3 min), 2.6% (Rt = 25.3 min).

Enantioenriched compound:

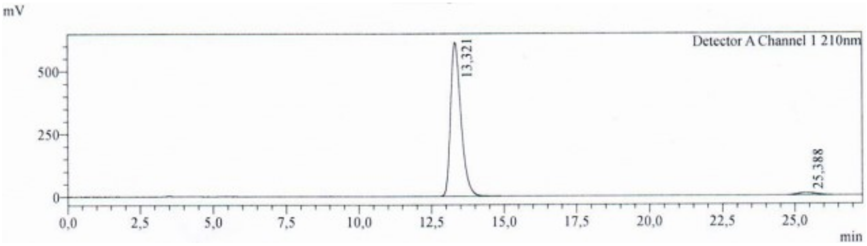

Detector A Channel 1 210nm

Peak#	Ret. Time	Area	Area%
1	13,321	15342994	97,395
2	25,388	410444	2,605
Total		15753438	100,000

Racemic compound:

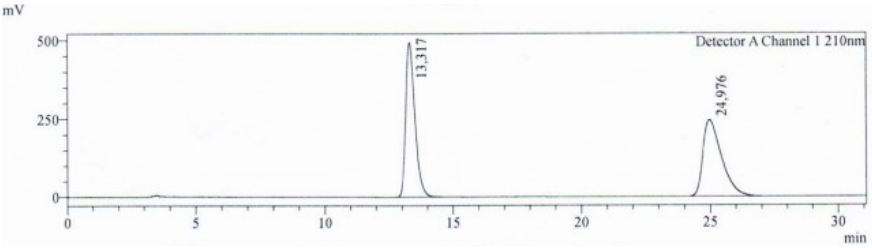

Detector A Channel 1 210nm

Peak#	Ret. Time	Area	Area%
1	13,317	12372350	49,882
2	24,976	12430920	50,118
Total		24803270	100,000

(2*R*,3*S*,5*S*)-6,6,6-trifluoro-5-hydroxy-2-methyl-1-(pyrrolidin-1-yl)-3-(m-tolyl)hexan-1-one (3i)

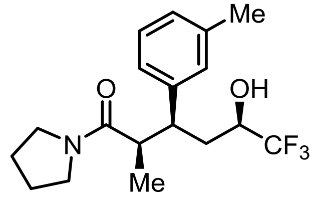

$C_{18}H_{24}F_3NO_2$
MW: 343.18

Synthesized following General Procedure 3 on 0.100 mmol scale from **1b** and pyrrolidine **2a**. Purification by flash column chromatography (heptane/EtOAc = 7:3) to afford the title compound in 86% yield (29.5 mg, 0.086 mmol).

^{1}H NMR (700 MHz, CDCl$_3$) δ 7.21 (t, *J* = 7.5 Hz, 1H), 7.05 (d, *J* = 7.5 Hz, 1H), 7.02 – 6.97 (m, 2H), 3.87 – 3.78 (m, 1H), 3.62 (s, 1H), 3.58 – 3.45 (m, 4H), 3.19 (dt, *J* = 10.4, 6.8 Hz, 1H), 2.74 (dq, *J* = 10.4, 6.9 Hz, 1H), 2.34 (s, 3H), 2.05 – 1.87 (m, 6H), 0.86 (d, *J* = 6.9 Hz, 3H) ppm.

^{13}C NMR (176 MHz, CDCl$_3$) δ 175.1, 143.0, 138.4, 128.8, 128.6, 127.6, 125.2 (q, *J* = 281.3 Hz), 125.1, 68.9 (q, *J* = 30.5 Hz), 46.8, 46.2, 44.0 (2C), 36.4, 26.1, 24.3, 21.5, 17.1 ppm.

^{19}F NMR (659 MHz, CDCl$_3$) δ −79.14 (s) ppm.

IR (neat) ν_{max}: 3312, 2934, 1621, 1438, 1135, 1102, 708 cm^{-1}.

HRMS (ESI$^+$): exact mass calculated for [M+Na]$^+$ ($C_{18}H_{24}F_3NO_2Na^+$) requires *m/z* 366.1651, found 366.1654 *m/z*.

$[\alpha]_D^{20}$ = −0.42 (c = 1.0, $CHCl_3$).

Determination of enantiomeric excess: ee: 72%. Method description: Chiralpak IC 250 x 4.6 mm, particle size 5 μm, solvent system: 77% (*n*-heptane+0.1%IPA):20% MTBE:3% IPA; flow 1 mL/min, 25 °C. Peak area 14.0% (Rt = 13.5 min), 86.0% (Rt = 15.5 min).

Enantioenriched compound:

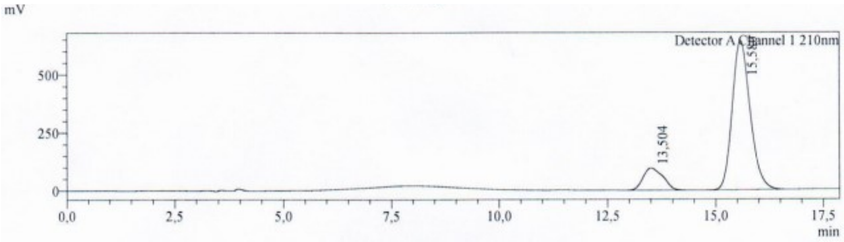

Detector A Channel 1 210nm

Peak#	Ret. Time	Area	Area%
1	13,504	3058032	14,064
2	15,580	18685912	85,936
Total		21743943	100,000

Racemic compound:

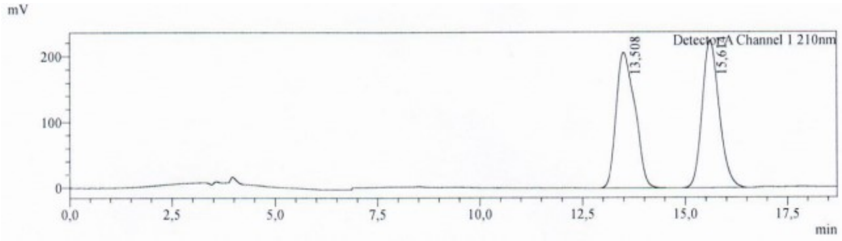

Detector A Channel 1 210nm

Peak#	Ret. Time	Area	Area%
1	13,508	6573145	50,188
2	15,614	6523777	49,812
Total		13096922	100,000

(2*R*,3*S*,5*S*)-6,6,6-trifluoro-5-hydroxy-3-(4-methoxyphenyl)-2-methyl-1-(pyrrolidin-1-yl)hexan-1-one (3j)

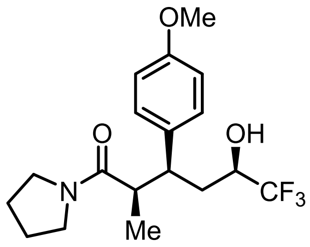

$C_{18}H_{24}F_3NO_3$
MW: 359.17

Synthesized following General Procedure 3 on 0.150 mmol scale from **1c** and pyrrolidine **2a**. Purification by flash column chromatography (heptane/EtOAc = 7:3) to afford the title compound in 81% yield (43.7 mg, 0.122 mmol).

^{1}H NMR (700 MHz, $CDCl_3$) δ 7.14 – 7.09 (m, 2H), 6.89 – 6.83 (m, 2H), 3.84 – 3.80 (m, 1H), 3.79 (s, 3H), 3.64 – 3.61 (m, 1H), 3.56 – 3.44 (m, 4H), 3.19 (dt, *J* = 10.3, 6.8 Hz, 1H), 2.70 (dq, *J* = 10.3, 6.9 Hz, 1H), 2.05 – 1.85 (m, 6H), 0.85 (d, *J* = 6.9 Hz, 3H) ppm.

^{13}C NMR (176 MHz, $CDCl_3$) δ 175.1, 158.4, 134.9, 129.0, 125.2 (q, *J* = 281.7 Hz), 114.1, 68.9 (q, *J* = 30.6 Hz), 55.2, 46.8, 46.1, 44.2, 43.2, 36.5, 26.1, 24.3, 17.0 ppm.

^{19}F NMR (659 MHz, $CDCl_3$) δ −79.11 (s) ppm.

IR (neat) ν_{max}: 3307, 2954, 1601, 1157, 1120 cm^{-1}.

HRMS (ESI$^+$): exact mass calculated for $[M+Na]^+$ ($C_{18}H_{24}F_3NO_3Na^+$) requires *m/z* 382.1600, found 382.1603 *m/z*.

$[\alpha]_D^{20}$ = −0.56 (c = 1.0, $CHCl_3$).

Determination of enantiomeric excess: ee: 86%. Method description: Lux Cellulose 1 (Chiralcel OD-H) 250 x 4.6 mm, particle size 5 μm, solvent system: *n*-heptane+0.1%IPA/IPA 85:15; flow 1 mL/min, 25 °C. Peak area 7.0% (Rt = 11.0 min), 93.0% (Rt = 13.4 min).

Enantioenriched compound:

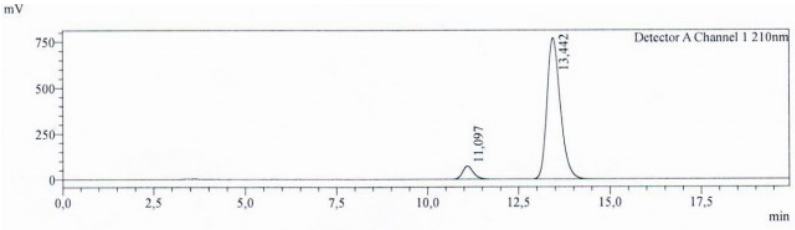

Detector A Channel 1 210nm

Peak#	Ret. Time	Area	Area%
1	11,097	1470766	6,994
2	13,442	19557538	93,006
Total		21028304	100,000

Racemic compound:

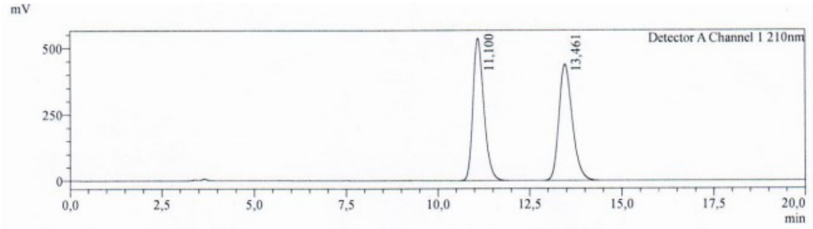

Detector A Channel 1 210nm

Peak#	Ret. Time	Area	Area%
1	11,100	11068566	49,935
2	13,461	11097253	50,065
Total		22165820	100,000

(2*R*,3*S*,5*S*)-6,6,6-trifluoro-5-hydroxy-3-(4-methoxyphenyl)-2-methyl-1-(pyrrolidin-1-yl)hexan-1-one (3k)

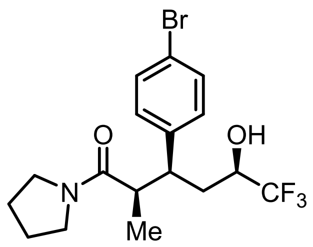

$C_{17}H_{21}F_3NO_2Br$
MW: 407.07

Synthesized following General Procedure 3 on 0.100 mmol scale from **1d** and pyrrolidine **2a**. Purification by flash column chromatography (heptane/EtOAc = 7:3) to afford the title compound in 84% yield (34.2 mg, 0.084 mmol).

^{1}H NMR (600 MHz, $CDCl_3$) δ 7.44 (d, *J* = 8.4 Hz, 2H), 7.08 (d, *J* = 8.4 Hz, 2H), 3.97 (d, *J* = 5.0 Hz, 1H), 3.88 – 3.76 (m, 1H), 3.59 – 3.42 (m, 4H), 3.23 (dt, *J* = 10.2, 6.6 Hz, 1H), 2.71 (dq, *J* = 10.4, 6.9 Hz, 1H), 2.06 – 1.85 (m, 6H), 0.85 (d, *J* = 6.9 Hz, 3H) ppm.

^{13}C NMR (151 MHz, $CDCl_3$) δ 174.7, 142.2, 131.8, 129.9, 125.1 (q, *J* = 281.8 Hz), 120.5, 68.6 (q, *J* = 30.8 Hz), 46.8, 46.2, 44.0, 43.3, 36.3, 26.1, 24.2, 17.0 ppm.

^{19}F NMR (565 MHz, $CDCl_3$) δ −79.10 (d, *J* = 6.2 Hz) ppm.

IR (neat) ν_{max}: 3358, 2961, 1741, 1608, 1108, 869, 550 cm^{-1}.

HRMS (ESI$^+$): exact mass calculated for $[M+Na]^+$ ($C_{17}H_{21}F_3NO_2BrNa^+$) requires *m/z* 430.0600, found 430.0601 *m/z*.

$[\alpha]_D^{20}$ = −0.57 (c = 1.0, $CHCl_3$).

Determination of enantiomeric excess: ee: 84%. Method description: Chiralpak IC 250 x 4.6 mm, particle size 5 μm, solvent system: *n*-heptane+0.1%IPA/IPA 92:8; flow 1 mL/min, 25 °C. Peak area 7.8% (Rt = 11.9 min), 92.2% (Rt = 14.4 min).

Enantioenriched compound:

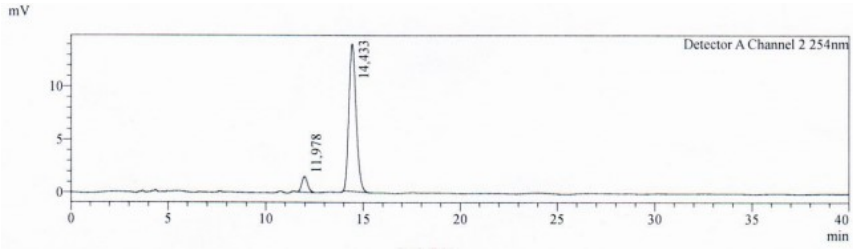

Detector A Channel 2 254nm

Peak#	Ret. Time	Area	Area%
1	11,978	30903	7,878
2	14,433	361341	92,122
Total		392243	100,000

Racemic compound:

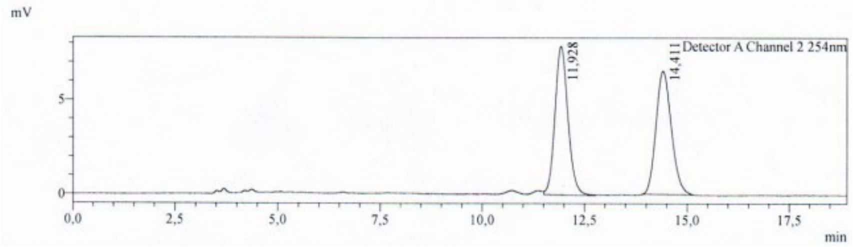

Detector A Channel 2 254nm

Peak#	Ret. Time	Area	Area%
1	11,928	174057	50,414
2	14,411	171200	49,586
Total		345257	100,000

(2*R*,3*S*,5*S*)-6,6,6-trifluoro-5-hydroxy-2-methyl-3-(naphthalen-2-yl)-1-(pyrrolidin-1-yl)hexan-1-one (3l)

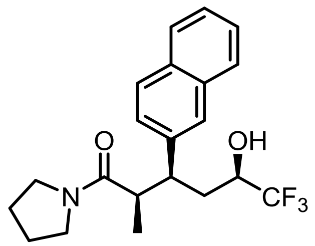

$C_{21}H_{24}F_3NO_2$
MW: 379.18

Synthesized following General Procedure 3 on 0.150 mmol scale from **1e** and pyrrolidine **2a**. Purification by flash column chromatography (heptane/EtOAc = 7:3) to afford the title compound in 72% yield (41.0 mg, 0.108 mmol).

^{1}H NMR (700 MHz, $CDCl_3$) δ 7.85 – 7.78 (m, 3H), 7.68 (s, 1H), 7.48 (dtd, *J* = 14.6, 6.9, 1.2 Hz, 2H), 7.33 (dd, *J* = 8.4, 1.6 Hz, 1H), 3.90 – 3.83 (m, 1H), 3.62 – 3.48 (m, 5H), 3.44 (dt, *J* = 10.3, 6.8 Hz, 1H), 2.87 (dq, *J* = 10.4, 6.9 Hz, 1H); 2.11 – 1.96 (m, 4H), 1.94 – 1.89 (m, 2H), 0.88 (d, *J* = 6.9 Hz, 3H) ppm.

^{13}C NMR (176 MHz, $CDCl_3$) δ 175.2, 140.6, 133.7, 132.6, 128.8, 127.82, 127.80, 127.3, 126.45, 126.0, 125.91, 125.3 (d, *J* = 281.3 Hz), 69.0 (q, *J* = 30.9 Hz), 47.0, 46.4, 44.2, 44.1, 36.7, 26.3, 24.4, 17.4 ppm.

^{19}F NMR (659 MHz, $CDCl_3$) δ −79.1 (s) ppm.

IR (neat) ν_{max}: 3312, 2957, 2929, 2876, 1608, 1508, 1458, 1372, 1227, 1198, 1061, 749 cm^{-1}.

HRMS (ESI^+): exact mass calculated for $[M+Na]^+$ ($C_{21}H_{24}O_2F_3NNa$) required *m/z* 402.1657, found *m/z* 402.1649.

$[\alpha]_D^{20}$ = -0.51 (c = 1.0, $CHCl_3$).

Determination of enantiomeric excess: ee: 88%. Method description: Lux-Cellulose 1 250x4.6 mm, particle size 5 μm, solvent system: *n*-heptane+0.1%IPA/IPA 95:5; flow 1 mL/min, 25 °C. Peak area 6.2% (Rt = 19.1 min), 93.8% (Rt = 22.6 min).

Enantioenriched compound:

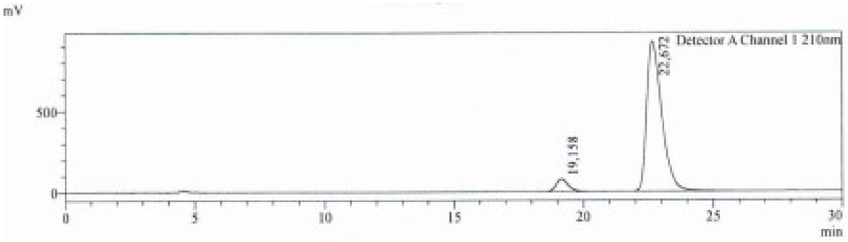

Detector A Channel 1 210nm

Peak#	Ret. Time	Area	Area%
1	19,158	2485472	6,201
2	22,672	37594452	93,799
Total		40079924	100,000

Racemic compound:

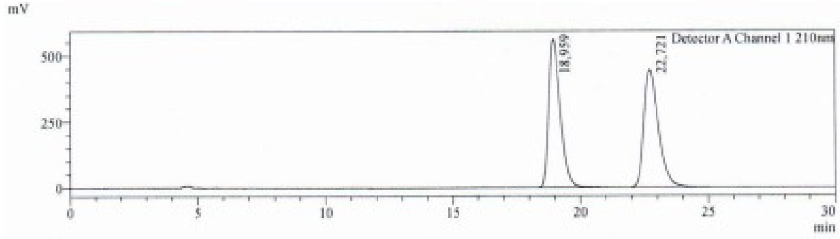

Detector A Channel 1 210nm

Peak#	Ret. Time	Area	Area%
1	18,959	17751142	49,991
2	22,721	17757632	50,009
Total		35508774	100,000

(2*R*,3*S*,5*S*)-6,6,6-trifluoro-3-(2-fluorophenyl)-5-hydroxy-2-methyl-1-(pyrrolidin-1-yl)hexan-1-one (3m)

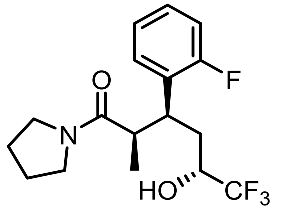

$C_{17}H_{21}F_4NO_2$
MW: 347.15

Synthesized following General Procedure 3 on 0.150 mmol scale from **1f** and pyrrolidine **2a**. Purification by flash column chromatography (heptane/EtOA = 7:3) to afford the title compound in 80% yield (41.7 mg, 0.120 mmol).

^{1}H NMR (700 MHz, CDCl$_3$) δ 7.24 – 7.20 (m, 2H), 7.09 (td, *J* = 7.6, 1.1 Hz, 1H), 7.03 (ddd, *J* = 8.8, 6.8, 1.0 Hz, 1H), 4.00 (s, 1H), 3.94 – 3.78 (m, 1H), 3.59 (dt, *J* = 9.8, 7.0 Hz, 1H), 3.55 – 3.46 (m, 4H), 2.98 (dq, *J* = 13.8, 6.8 Hz, 1H), 2.10 – 1.94 (m, 4H), 1.92 – 1.88 (m, 2H), 0.89 (d, *J* = 6.9 Hz, 3H) ppm.

^{13}C NMR (176 MHz, CDCl$_3$) δ 175.0, 161.2 (d, *J* = 245.3 Hz), 130.8, 130.0 (d, *J* = 13.9 Hz), 128.5 (d, *J* = 8.5 Hz), 125.1 (q, *J* = 281.6 Hz), 124.3 (d, *J* = 3.2 Hz), 115.9 (d, *J* = 22.5 Hz), 68.7 (q, *J* = 30.7 Hz), 46.8, 46.2, 42.2 (2C), 34.9, 26.1, 24.3, 17.0 ppm.

^{19}F NMR (659 MHz, CDCl$_3$) δ −79.19 (d, *J* = 25.6 Hz), −115.97 (s) ppm.

IR (neat) ν_{max}: 3291, 2927, 1608, 1157, 1111, 706 cm^{-1}.

HRMS (ESI$^+$): exact mass calculated for [M+Na]$^+$ ($C_{17}H_{21}F_4NO_2Na^+$) requires *m/z* 370.1401, found 370.1402 *m/z*.

$[\alpha]_D^{20}$ = 0.02 (c = 1.0, $CHCl_3$).

Determination of enantiomeric excess: ee: 80%. Method description: Lux-Cellulose 1 250x4.6 mm, particle size 5 μm, solvent system: *n*-heptane+0.1%IPA/IPA 97:3; flow 1 mL/min, 25 °C. Peak area 10.1% (Rt = 12.3 min), 89.9% (Rt = 13.3 min).

Enantioenriched compound:

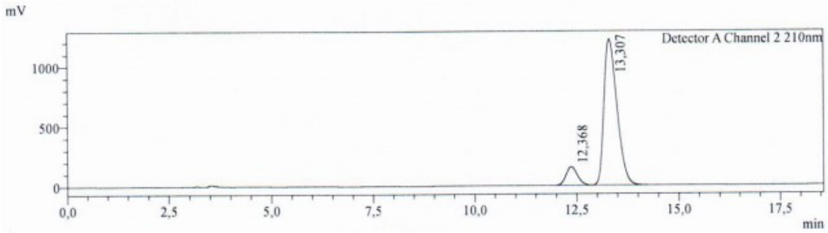

Detector A Channel 2 210nm

Peak#	Ret. Time	Area	Area%
1	12,368	3030862	10,090
2	13,307	27006540	89,910
Total		30037402	100,000

Racemic compound:

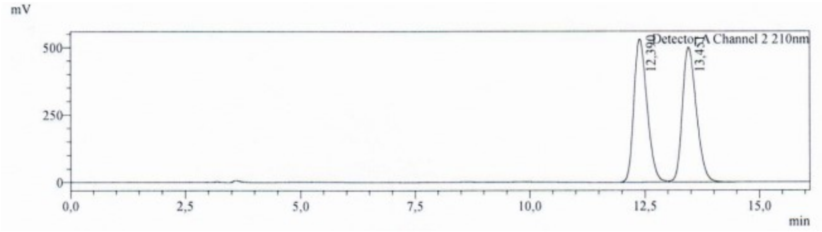

Detector A Channel 2 210nm

Peak#	Ret. Time	Area	Area%
1	12,390	10589700	49,943
2	13,451	10614047	50,057
Total		21203747	100,000

(2*R*,3*S*,5*S*)-6,6,6-trifluoro-5-hydroxy-2-isopropyl-3-phenyl-1-(pyrrolidin-1-yl)hexan-1-one (3n)

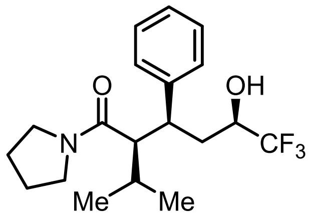

$C_{19}H_{26}F_3NO_2$
MW: 357.19

Synthesized following General Procedure 3 on 0.150 mmol scale from **1g** and pyrrolidine **2a**. Purification by flash column chromatography (heptane/EtOAc = 7:3) to afford the title compound in 86% yield (46.1 mg, 0.129 mmol).

^{1}H NMR (700 MHz, $CDCl_3$) δ 7.32 – 7.27 (m, 2H), 7.25 – 7.21 (m, 3H), 3.83 – 3.74 (m, 1H), 3.45 – 3.40 (m, 1H), 3.39 – 3.33 (m, 2H), 3.31 – 3.26 (m, 1H), 3.19 (dd, *J* = 15.6, 7.2 Hz, 1H), 2.58 (s, 1H), 2.52 (t, *J* = 7.5 Hz, 1H), 2.37 (dd, *J* = 18.5, 11.8 Hz, 1H), 2.28 – 2.22 (m, 1H), 2.01 – 1.93 (m, 1H), 1.77 – 1.67 (m, 3H), 1.60 – 1.52 (m, 1H), 0.97 (d, *J* = 6.8 Hz, 3H), 0.88 (d, *J* = 6.6 Hz, 3H) ppm.

^{13}C NMR (176 MHz, $CDCl_3$) δ 172.3, 142.8, 128.7, 128.0, 127.0, 126.5 (q, *J* = 281.6 Hz), 69.3 (q, *J* = 30.5 Hz), 56.1, 46.9, 45.6, 42.1, 32.4, 28.7, 25.9, 24.2, 20.3, 20.0 ppm.

^{19}F NMR (659 MHz, $CDCl_3$) δ −78.87 (s) ppm.

IR (neat) ν_{max}: 3309, 2939, 1608, 1144, 1108, 704 cm^{-1}.

HRMS (ESI$^+$): exact mass calculated for $[M+Na]^+$ ($C_{19}H_{26}F_3NO_2Na^+$) requires *m*/*z* 380.1808, found 380.1810 *m*/*z*.

$[\alpha]_D^{20}$ = −0.53 (c = 1.0, $CHCl_3$).

Determination of enantiomeric excess: ee > 99.9%. Method description: Chiralpak IC 250 x 4.6 mm, particle size 5 μm, solvent system: 77% (*n*-heptane+0.1%IPA):20% MTBE:3% IPA; flow 1 mL/min, 25 °C. Peak area 0.0% (Rt = 9.7 min), 100.0% (Rt = 15.3 min).

Enantiopure compound:

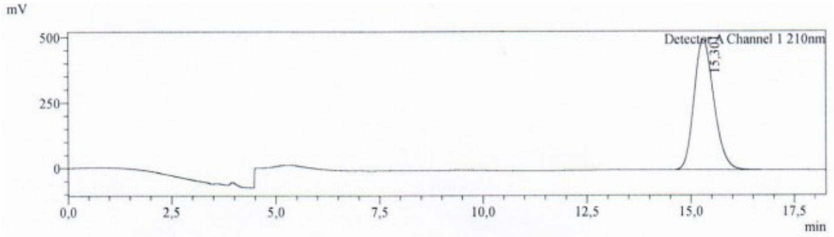

Detector A Channel 1 210nm

Peak#	Ret. Time	Area	Area%
1	15,301	16644340	100,000
Total		16644340	100,000

Racemic compound:

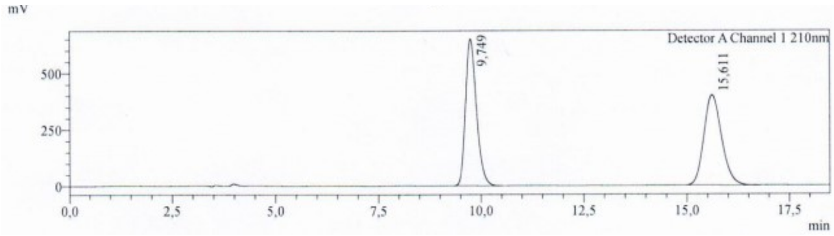

Detector A Channel 1 210nm

Peak#	Ret. Time	Area	Area%
1	9,749	11998589	50,034
2	15,611	11982217	49,966
Total		23980806	100,000

(2*R*,3*S*,5*S*)-6,6,6-trifluoro-5-hydroxy-2-isopropyl-3-phenyl-1-(piperidin-1-yl)hexan-1-one (3o)

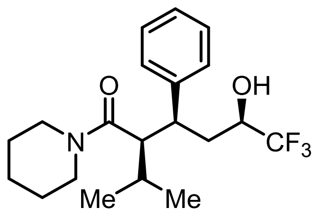

$C_{20}H_{28}F_3NO_2$
MW: 371.21

Synthesized following General Procedure 3 on 0.150 mmol scale from **1g** and piperidine **2b**. Purification by flash column chromatography (heptane/EtOAc = 7:3) to afford the title compound in 70% yield (39.0 mg, 0.105 mmol).

^{1}H NMR (700 MHz, $CDCl_3$) δ 7.31 (t, *J* = 7.6 Hz, 2H), 7.25 – 7.21 (m, 3H), 3.88 – 3.79 (m, 1H), 3.62 – 3.57 (m, 1H), 3.54 – 3.48 (m, 1H), 3.32 (dd, *J* = 13.9, 8.2 Hz, 1H), 3.24 – 3.18 (m, 1H), 3.15 – 3.10 (m, 1H), 3.00 (s, 1H), 2.85 (t, *J* = 7.4 Hz, 1H), 2.22 (dt, *J* = 16.0, 8.2 Hz, 1H), 2.15 (dt, *J* = 14.8, 5.5 Hz, 1H), 1.86 (dp, *J* = 13.5, 6.7 Hz, 1H), 1.59 – 1.38 (m, 5H), 1.21 – 1.12 (m, 1H), 0.92 (d, *J* = 6.8 Hz, 3H), 0.85 (d, *J* = 6.7 Hz, 3H) ppm.
^{13}C NMR (176 MHz, $CDCl_3$) δ 171.94, 142.88, 128.78, 128.13, 126.90, 125.30 (q, *J* = 281.9 Hz), 69.41 (q, *J* = 30.5 Hz), 51.80, 47.19, 42.67, 42.14, 33.49, 28.91, 26.23, 25.59, 24.41, 20.72, 19.37 ppm.
^{19}F NMR (659 MHz, $CDCl_3$) δ −79.01 (d, *J* = 4.8 Hz) ppm.
IR (neat) ν_{max}: 3304, 2941, 1606, 1424, 1141, 1115, 714 cm^{-1}.
HRMS (ESI$^+$): exact mass calculated for $[M+Na]^+$ ($C_{20}H_{28}F_3NO_2Na^+$) requires *m/z* 394.1964, found 394.1968 *m/z*.

$[\alpha]_D^{20}$ = −0.20 (c = 1.0, $CHCl_3$).

Determination of enantiomeric excess: ee: 97%. Method description: Chiralpak IC 250 x 4.6 mm, particle size 5 μm, solvent system: 77% (*n*-heptane+0.1%IPA):20% MTBE:3% IPA; flow 1 mL/min, 25 °C. Peak area 1.4% (Rt = 7.2 min), 98.6% (Rt = 9.5 min).

Enantioenriched compound:

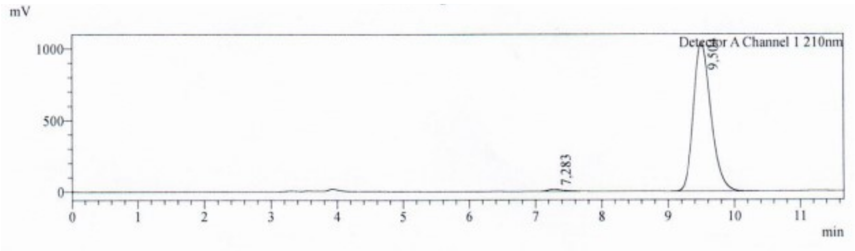

Detector A Channel 1 210nm

Peak#	Ret. Time	Area	Area%
1	7,283	257781	1,329
2	9,504	19145269	98,671
Total		19403051	100,000

Racemic compound:

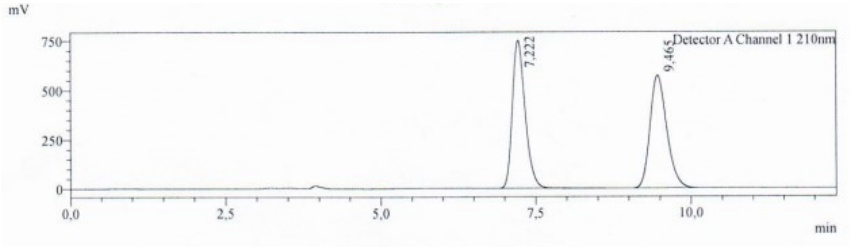

Detector A Channel 1 210nm

Peak#	Ret. Time	Area	Area%
1	7,222	10833968	50,249
2	9,465	10726710	49,751
Total		21560678	100,000

(2*R*,3*S*,5*S*)-*N*,*N*-diethyl-6,6,6-trifluoro-5-hydroxy-2-isopropyl-3-phenylhexanamide (3p)

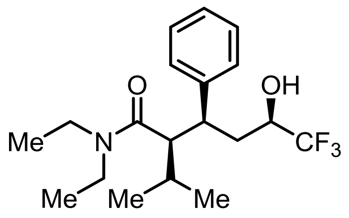

$C_{19}H_{28}F_3NO_2$
MW: 359.21

Synthesized following General Procedure 3 on 0.150 mmol scale from **1g** and *N*,*N*-diethylamine **2e**. Purification by flash column chromatography (heptane/EtOAc = 7:3) to afford the title compound in 54% yield (29.1 mg, 0.081 mmol).

^{1}H NMR (600 MHz, CDCl$_3$) δ 7.33 – 7.28 (m, 2H), 7.25 – 7.21 (m, 3H), 3.86 – 3.77 (m, 1H), 3.40 (dq, *J* = 14.1, 7.1 Hz, 1H), 3.36 – 3.25 (m, 2H), 3.13 (dq, *J* = 14.5, 7.2 Hz, 1H), 2.87 (dq, *J* = 14.6, 7.2 Hz, 1H), 2.82 (s, 1H), 2.68 (t, *J* = 7.3 Hz, 1H), 2.19 (t, *J* = 6.9 Hz, 2H), 1.87 (dq, *J* = 13.6, 6.8 Hz, 1H), 1.12 (t, *J* = 7.1 Hz, 3H), 0.99 (t, *J* = 7.2 Hz, 3H), 0.94 (d, *J* = 6.8 Hz, 3H), 0.86 (d, *J* = 6.7 Hz, 3H) ppm.

^{13}C NMR (151 MHz, CDCl$_3$) δ 173.1, 143.1, 129.0, 128.3, 127.2, 125.4 (q, *J* = 282.1 Hz), 69.6 (q, *J* = 30.5 Hz), 52.5, 42.5, 42.1, 40.8, 33.8, 29.3, 20.8, 19.8, 14.3, 12.9 ppm.

^{19}F NMR (565 MHz, CDCl$_3$) δ −79.07 (d, *J* = 6.3 Hz) ppm.

IR (neat) ν_{max}: 3301, 2947, 1605, 1424, 1141, 1111, 712 cm^{-1}.

HRMS (ESI$^+$): exact mass calculated for $[M+Na]^+$ ($C_{19}H_{28}F_3NO_2Na^+$) requires *m/z* 382.1964, found 382.1965 *m/z*.

$[\alpha]_D^{20}$ = −0.22 (c = 1.0, $CHCl_3$).

Determination of enantiomeric excess: ee > 99%. Method description: Chiralpak IC 250 x 4.6 mm, solvent system: 77% (*n*-heptane+0.1%IPA):20% MTBE:3% IPA; flow 1 mL/min, 25 °C. Peak area 0.2% (Rt = 6.2 min), 99.8% (Rt = 8.3 min).

Enantiopure compound:

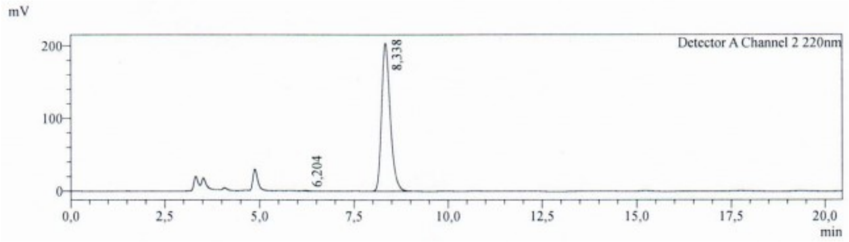

Detector A Channel 2 220nm

Peak#	Ret. Time	Area	Area%
1	6,204	6273	0,192
2	8,338	3263226	99,808
Total		3269498	100,000

Racemic compound:

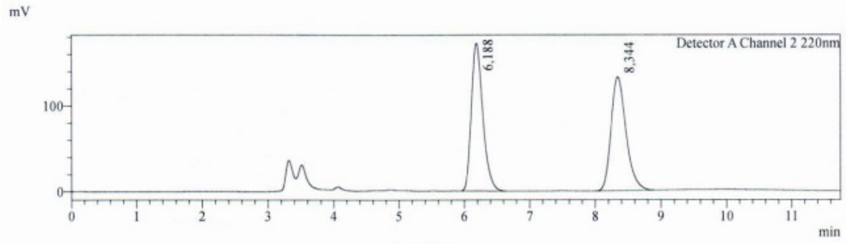

Detector A Channel 2 220nm

Peak#	Ret. Time	Area	Area%
1	6,188	2115818	49,952
2	8,344	2119916	50,048
Total		4235734	100,000

(2R,3S,5R)-6,6,6-trifluoro-5-hydroxy-2-isopropyl-3-phenyl-1-(1,4-dioxa-8-azaspiro[4.5]decan-8-yl)hexan-1-one (3q)

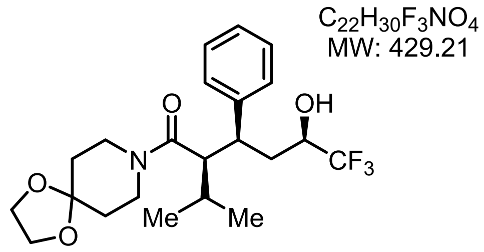

Synthesized following General Procedure 3 on 0.15 mmol scale from **1g** and 1,4-dioxa-8-azaspiro[4.5]decane **2i**. Purification by flash column chromatography (heptane/EtOAc = 7:3) to afford the title compound in 46% yield (29.6 mg, 0.069 mmol).

^{1}H NMR (700 MHz, $CDCl_3$) δ 7.31 (t, *J* = 7.5 Hz, 2H), 7.25 – 7.22 (m, 3H), 3.95 – 3.90 (m, 4H), 3.80 (dt, *J* = 12.8, 6.4 Hz, 1H), 3.74 – 3.67 (m, 1H), 3.67 – 3.61 (m, 1H), 3.33 – 3.23 (m, 2H), 3.18 – 3.09 (m, 1H), 2.84 (d, *J* = 5.8 Hz, 1H), 2.80 (t, *J* = 7.4 Hz, 1H), 2.30 (ddd, *J* = 16.5, 9.6, 7.2 Hz, 1H), 2.24 – 2.18 (m, 1H), 1.94 (dq, *J* = 13.7, 6.8 Hz, 1H), 1.61 – 1.53 (m, 2H), 1.50 – 1.44 (m, 1H), 1.19 – 1.13 (m, 1H), 0.96 (d, *J* = 6.8 Hz, 3H), 0.84 (d, *J* = 6.6 Hz, 3H) ppm.

^{13}C NMR (176 MHz, $CDCl_3$) δ 171.9, 142.5, 128.9, 128.1, 127.1, 125.3 (q, *J* = 282.2 Hz), 106.7, 69.6 (q, *J* = 30.4 Hz), 64.4, 64.35, 52.3, 43.9, 42.3, 39.5, 35.2, 34.7, 32.5, 28.9, 20.5, 19.8.

^{19}F NMR (659 MHz, $CDCl_3$) δ –78.99 (d, *J* = 6.8 Hz).

IR (neat) ν_{max}: 3294, 2951, 1611, 1419, 1183, 1138, 1115, 717 cm^{-1}.

HRMS (ESI$^+$): exact mass calculated for $[M+Na]^+$ ($C_{22}H_{30}F_3NO_4Na^+$) requires *m/z* 452.2019, found 452.2017 *m/z*.

$[\alpha]_D^{20}$ = −0.43 (c = 1.0, $CHCl_3$).

Determination of enantiomeric excess: ee = 99%. Method description: Chiralpak IC 250 x 4.6 mm, solvent system: (*n*-heptane+0.1%IPA)/IPA = 85:15; flow 1 mL/min, 25 °C. Peak area 0.5% (Rt = 6.1 min), 99.5% (Rt = 7.3 min).

Enantiopure compound:

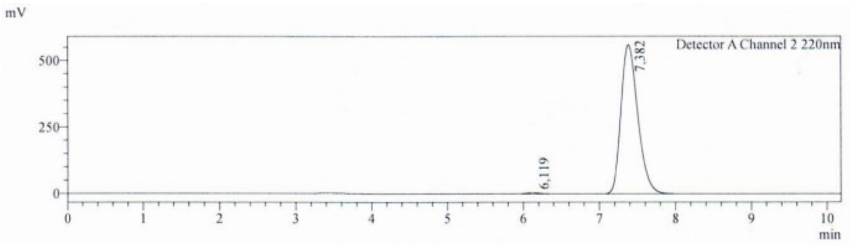

Detector A Channel 2 220nm

Peak#	Ret. Time	Area	Area%
1	6,119	42197	0,489
2	7,382	8594706	99,511
Total		8636904	100,000

Racemic compound:

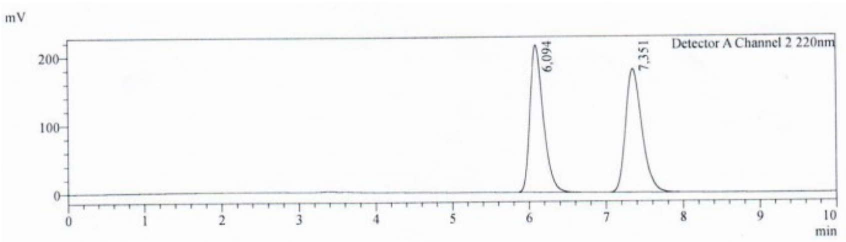

Detector A Channel 2 220nm

Peak#	Ret. Time	Area	Area%
1	6,094	2619720	49,728
2	7,351	2648344	50,272
Total		5268065	100,000

(2*S*,4*S*,5*R*,6*R*)-4-(4-bromophenyl)-6-(1*H*-indazol-1-yl)-5-methyl-2-(trifluoromethyl)tetrahydro-2*H*-pyran-2-ol (4r)

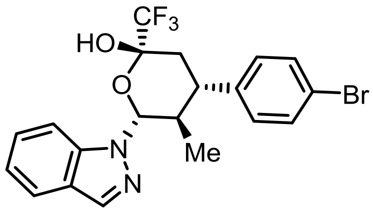

$C_{20}H_{18}F_3N_2O_2Br$
MW: 454.05

Synthesized following General Procedure 3 on 0.150 mmol scale from **1d** and indazole **2j**. Purification by flash column chromatography (heptane/EtOAc = 9:1) to afford the title compound in 89% yield (60.6 mg, 0.134 mmol).

^{1}H NMR (600 MHz, $CDCl_3$) δ 8.03 (s, 1H), 7.74 (d, *J* = 8.1 Hz, 1H), 7.62 (d, *J* = 8.5 Hz, 1H), 7.49 (d, *J* = 8.1 Hz, 2H), 7.43 (t, *J* = 7.7 Hz, 1H), 7.24 – 7.15 (m, 3H), 6.14 (d, *J* = 10.0 Hz, 1H), 4.81 (s, 1H) 3.17 (td, *J* = 11.9, 4.3 Hz, 1H), 2.88 – 2.75 (m, 1H), 2.24 – 2.12 (m, 2H), 0.52 (d, *J* = 6.5 Hz, 3H) ppm.

^{13}C NMR (151 MHz, $CDCl_3$) δ 140.6, 139.1, 134.6, 132.0, 129.3, 127.2, 125.0, 122.3 (q, *J* = 286.2 Hz), 121.7, 121.5, 121.0, 110.2, 95.7 (q, *J* = 32.8 Hz), 87.2, 42.7, 39.0, 35.0, 13.5 ppm.

^{19}F NMR (565 MHz, $CDCl_3$) δ −86.51 (s), −86.69 (s) ppm.

IR (neat) ν_{max}: 3361, 2989, 1657, 1201, 1013, 841 cm^{-1}.

HRMS (ESI$^+$): exact mass calculated for $[M+Na]^+$ ($C_{20}H_{18}F_3N_2O_2BrNa^+$) requires *m/z* 477.0396, found 477.0398 *m/z*.

$[\alpha]_D^{20}$ = −0.12 (c = 1.0, $CHCl_3$).

Determination of enantiomeric excess: ee = 87%. Method description: Lux-Cellulose-1 (OD-H) 250 x 4.6 mm, solvent system: *n*-heptane+0.1%IPA/IPA = 98:2; flow 1 mL/min, 25 °C. Peak area 93.5% (Rt = 18.0 min), 6.512% (Rt = 23.8 min).

Enantioenriched compound:

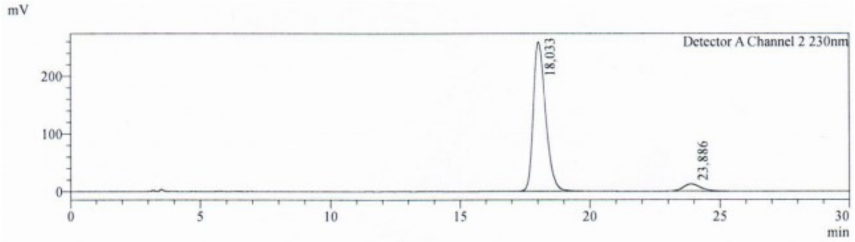

Detector A Channel 2 230nm

Peak#	Ret. Time	Area	Area%
1	18,033	8546055	93,488
2	23,886	595280	6,512
Total		9141335	100,000

Racemic compound:

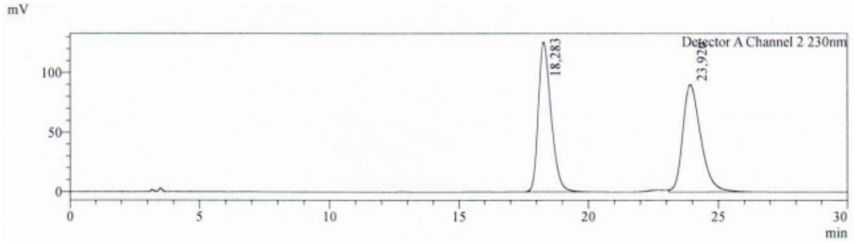

Detector A Channel 2 230nm

Peak#	Ret. Time	Area	Area%
1	18,283	4352024	49,776
2	23,920	4391158	50,224
Total		8743183	100,000

4. Mechanistic studies

4.1 Labelling Experiments

(2*R*,3*S*,5*S*)-6,6,6-trifluoro-5-hydroxy-2-methyl-3-phenyl-1-(piperidin-1-yl-d10)hexan-1-one-4-d (8)

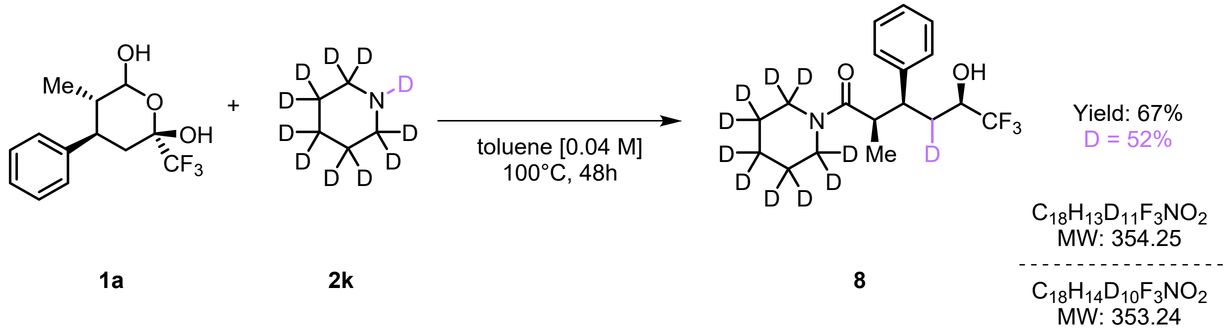

In a borosilicate glass vial containing a magnetic stirring bar, hemiacetal **1a** (49.9 mg, 0.180 mmol, 1.00 equiv.) was dissolved in toluene (0.04 M). To this stirring solution, piperidine-d-11 **2k** was added (31.5 µL, 0.360 mmol, 2.00 equiv.) at 25 °C and the mixture was stirred for 30min. Subsequently, the vial was transferred to an oil bath and was heated to 100 °C for 48h. After this time, the reaction mixture was allowed to cool to 25 °C. The volatiles were removed under reduced pressure and the resulting crude product was purified by flash column chromatography on silica gel (heptane/EtOAc = 7:3) to afford the desired product in 67% yield (42.8 mg, 0.121 mmol) with a deuterium incorporation at C-4 of 52%.

^{1}H NMR (700 MHz, $CDCl_3$) δ 7.32 (t, *J* = 7.6 Hz, 2H), 7.24 (t, *J* = 7.4 Hz, 1H), 7.21 (d, *J* = 7.3 Hz, 2H), 3.89 – 3.80 (m, 1H), 3.73 – 3.65 (m, 1H), 3.36 – 3.27 (m, 1H), 2.92 (dq, *J* = 10.3, 7.0 Hz, 1H), 1.99 – 1.90 (m, 1.48H), 0.81 (d, *J* = 7.0 Hz, 3H) ppm.

^{13}C NMR (176 MHz, $CDCl_3$) δ 174.8, 143.1 (d, *J* = 3.2 Hz), 128.8, 128.2, 126.9, 125.2 (q, *J* = 281.5 Hz), 69.1 – 68.5 (m), 43.7 (dd, *J* = 18.5, 7.9 Hz), 42.2 – 40.2 (m), 37.1 – 36.2 (m), 19.3 – 16.2 (m) ppm.

^{19}F NMR (659 MHz, $CDCl_3$) δ −78.83 – −79.22 (m) ppm.

IR (neat) ν_{max}: 3311, 2932, 16139 1428, 1151, 1100, 718 cm^{-1}.

HRMS (ESI^{+}): exact mass calculated for $[M-H+Na]^+$ ($C_{18}H_{14}D_{10}F_3NO_2Na^+$) requires *m/z* 376.2279, found 376.2277 *m/z*. exact mass calculated for $[M-D+Na]^+$ ($C_{18}H_{13}D_{11}F_3NO_2Na^+$) requires *m/z* 377.2342, found 377.2332 *m/z*.

(2*R*,3*S*,5*S*)-6,6,6-trifluoro-5-hydroxy-2-methyl-3-phenyl-1-(pyrrolidin-1-yl)hexan-1-one-5-d (9)

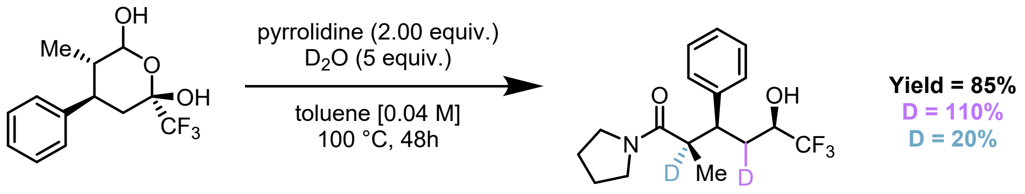

1a **9**

In a borosilicate glass vial containing a magnetic stirring bar, the hemiacetal **1a** (49.9 mg, 0.180 mmol, 1.00 equiv.) was dissolved in toluene (0.04 M). To this stirring solution, pyrrolidine was added (29.6 µL, 0.360 mmol, 2.00 equiv.), followed by D_2O (16.2 µL, 0.900 mmol, 5.00 equiv.) and the resulting reaction mixture was stirred for 30min at 25 °C. Subsequently, the vial was transferred to an oil bath and was heated to 100 °C for 48h. After this time, the reaction mixture was allowed to cool to 25 °C. The volatiles were removed under reduced pressure and the resulting crude product was purified by flash column chromatography on silica gel (heptane/EtOAc = 7:3) to afford the desired product in 85% yield (50.6 mg, 0.153 mmol) with a deuterium incorporation at C-2 of 20% and at C-4 of 110%.

^{1}H NMR (700 MHz, $CDCl_3$) δ 7.32 (t, *J* = 7.6 Hz, 2H), 7.24 (t, *J* = 7.4 Hz, 1H), 7.22 – 7.19 (m, 2H), 3.83 (ddd, *J* = 9.2, 8.4, 4.2 Hz, 1H), 3.62 – 3.44 (m, 5H), 3.25 (dt, *J* = 10.3, 6.8 Hz, 1H), 2.75 (dq, *J* = 10.4, 6.9 Hz, 0.8H), 2.07 – 1.94 (m, 2.9H), 1.94 – 1.87 (m, 2H), 0.86 (d, *J* = 6.9 Hz, 3H) ppm.

(2*R*,3*S*,5*S*)-6,6,6-trifluoro-5-hydroxy-2-methyl-3-phenyl-1-(pyrrolidin-1-yl)hexan-1-one-5-d (10)

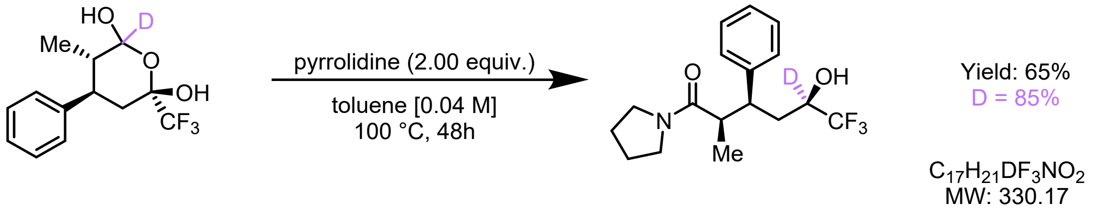

1h
D = 88%

10
D = 85%

In a borosilicate glass vial containing a magnetic stirring bar, the deuterated hemiacetal **1h** (50.0 mg, 0.180 mmol, 1.00 equiv., D = 88%) was dissolved in toluene (0.04 M). To this stirring solution, pyrrolidine was added (29.6 µL, 0.360 mmol, 2.00 equiv.) at 25 °C and the mixture was stirred for 30min. Subsequently, the vial was transferred to an oil bath and was heated to 100 °C for 48h. After this time, the reaction mixture was allowed to cool to 25 °C. The volatiles were removed under reduced pressure and the resulting crude product was purified by flash column chromatography on silica gel (heptane/EtOAc = 7:3) to afford the desired product in 65% yield (38.6 mg, 0.117 mmol) with a deuterium incorporation at C-5 of 85%.

^{1}H NMR (700 MHz, $CDCl_3$) δ 7.32 (t, *J* = 7.6 Hz, 2H), 7.24 (t, *J* = 7.4 Hz, 1H), 7.22 – 7.19 (m, 2H), 3.83 (ddd, *J* = 9.2, 8.4, 4.2 Hz, 0.15H), 3.62 – 3.44 (m, 5H), 3.25 (dt, *J* = 10.3, 6.8 Hz, 1H), 2.75 (dq, *J* = 10.4, 6.9 Hz, 1H), 2.07 – 1.94 (m, 4H), 1.94 – 1.87 (m, 2H), 0.86 (d, *J* = 6.9 Hz, 3H) ppm.

^{13}C NMR (176 MHz, $CDCl_3$) δ 175.0, 143.1, 128.8, 128.1, 126.9, 125.1 (q, *J* = 281.7 Hz), 68.8 (d, *J* = 30.7 Hz), 46.8, 46.2, 44.0, 43.9, 36.4, 26.1, 24.3, 17.1 ppm.

^{19}F NMR (659 MHz, $CDCl_3$) δ −79.11 (s), −79.12 (s), −79.1 (s), −79.14 (s) ppm.

IR (neat) ν_{max}: 3309, 2939, 1613, 1439, 1142, 1107, 703 cm^{-1}.

HRMS (ESI$^+$): exact mass calculated for $[M+Na]^+$ ($C_{17}H_{21}DF_3NO_2Na^+$) requires *m/z* 353.1558, found 353.1563 *m/z*.

4.2 Hemiacetal Intermediate

(2*S*,4*R*,5*S*,6*R*)-5-methyl-4-phenyl-6-(pyrrolidin-1-yl)-2-(trifluoromethyl)tetrahydro-2*H*-pyran-2-ol (4a)

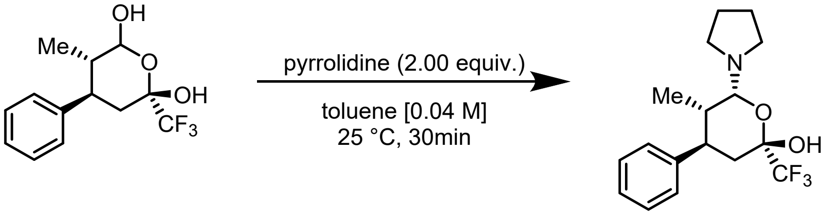

1a **4a**

Yield: 45%

$C_{17}H_{22}F_3NO_2$
MW: 329.16

In a borosilicate glass vial containing a magnetic stirring bar, the hemiacetal **1a** (55.3 mg, 0.200 mmol, 1.00 equiv.) was dissolved in toluene (0.04 M). To the stirring solution, pyrrolidine (32.9 µL, 0.400 mmol, 2.00 equiv.) was added and the resulting mixture was stirred for 30min at 25 °C. After this time, the volatiles were removed under reduced pressure and the resulting crude product was purified by flash column chromatography on silica gel (heptane/EtOAc = 7:3) to obtain the desired product **4a** in 45% yield (29.6 mg, 0.090 mmol) (*Nota bene: the observed low isolated yield is due to decomposition of the product during column chromatography*).

^{1}H NMR (600 MHz, $CDCl_3$) δ 7.37 – 7.32 (m, 2H), 7.29 – 7.26 (m, 0.6H), 7.26 – 7.22 (m, 1H), 7.22 – 7.18 (m, 1.4H), 5.41 – 5.36 (m, 0.3H), 5.07 (t, *J* = 7.5 Hz, 0.7H), 4.17 – 4.09 (m, 0.3H), 3.60 – 3.50 (m, 0.3H), 3.24 – 3.11 (m, 1H), 2.97 – 2.86 (m, 1.4H), 2.17 – 2.10 (m, 0.7H), 2.07 – 2.00 (m, 1H), 1.98 – 1.91 (m, 0.7H), 1.83 – 1.74 (m, 0.7H), 0.85 (d, *J* = 6.6 Hz, 2.1H), 0.79 (d, *J* = 6.9 Hz, 0.9H) ppm.

^{13}C NMR (151 MHz, $CDCl_3$) δ 141.7, 141.6, 128.8, 127.6, 127.5, 127.1, 127.1, 122.2 (q, *J* = 285.5 Hz), 122.1 (q, *J* = 285.5 Hz), 97.2, 97.1, 95.5 (q, *J* = 32.4 Hz), 95.4 (q, *J* = 32.4 Hz), 42.4, 42.3, 42.2, 39.2, 36.2, 36.0, 34.9, 14.2, 13.7 ppm.

^{19}F NMR (565 MHz, $CDCl_3$) δ −86.39 (s),

4.3 Retro Diels-Alder Demonstration

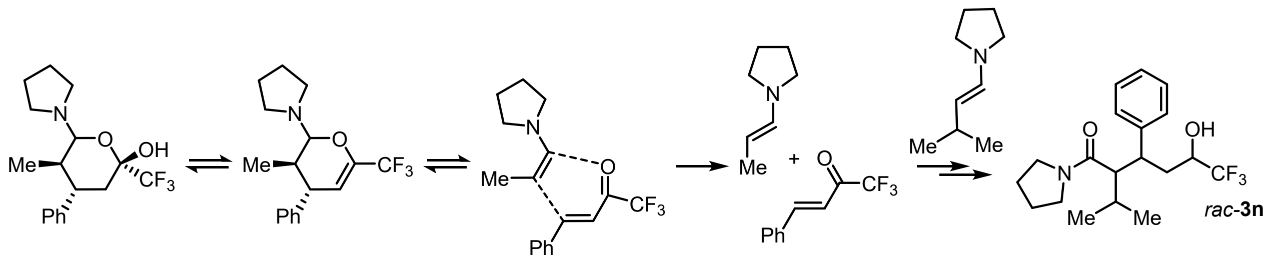

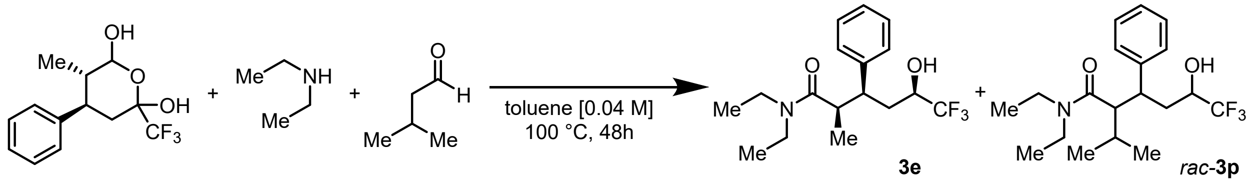

1a (1.00 equiv.) **2e** (5.10 equiv.) **S3c** (4.00 equiv.)

Yield = 23%
3e:*rac*-**3p = 7:1**

In a borosilicate glass vial containing a magnetic stirring bar, the hemiacetal **1a** (221 mg, 0.800 mmol, 1.00 equiv.) was dissolved in toluene (0.04 M). To the stirring solution, diethylamine (0.422 mL, 4.08 mmol, 5.10 equiv.) and 3-methylbutanal (0.343 mL, 3.20 mmol, 4.00 equiv.) were added and the resulting reaction mixture was stirred for 30min at 25 °C. Subsequently, the vial was transferred to an oil bath and was heated to 100 °C for 48h. After this time, the reaction mixture was allowed to cool to 25 °C. The volatiles were removed under reduced pressure and the resulting mixture of products was purified by flash column chromatography on silica gel (heptane/EtOAc = 7:3). Due to an extremely low difference in polarity, the products could not be obtained pure; nevertheless, the were obtained in a 7:1 mixture **3e**:*rac*-**3p** with an overall 23% yield.

HRMS:

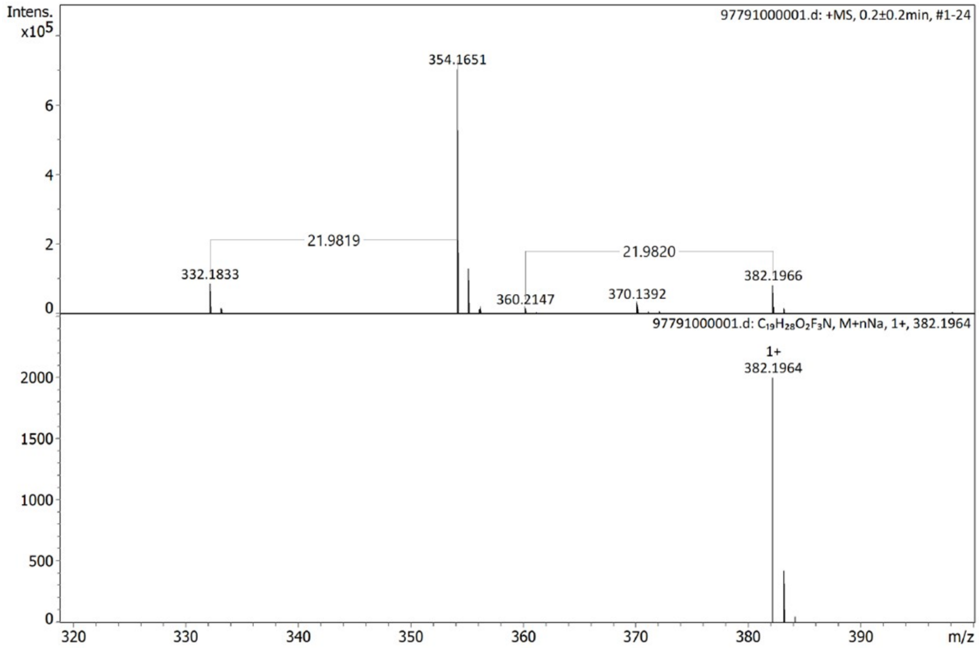

Intens.
x10^5
97791000001.d: +MS, 0.2±0.2min, #1-24
354.1651
21.9819
21.9820
332.1833
360.2147
370.1392
382.1966
97791000001.d: C19H28O2F3N, M+nNa, 1+, 382.1964
1+
382.1964
m/z

^{19}F – NMR:

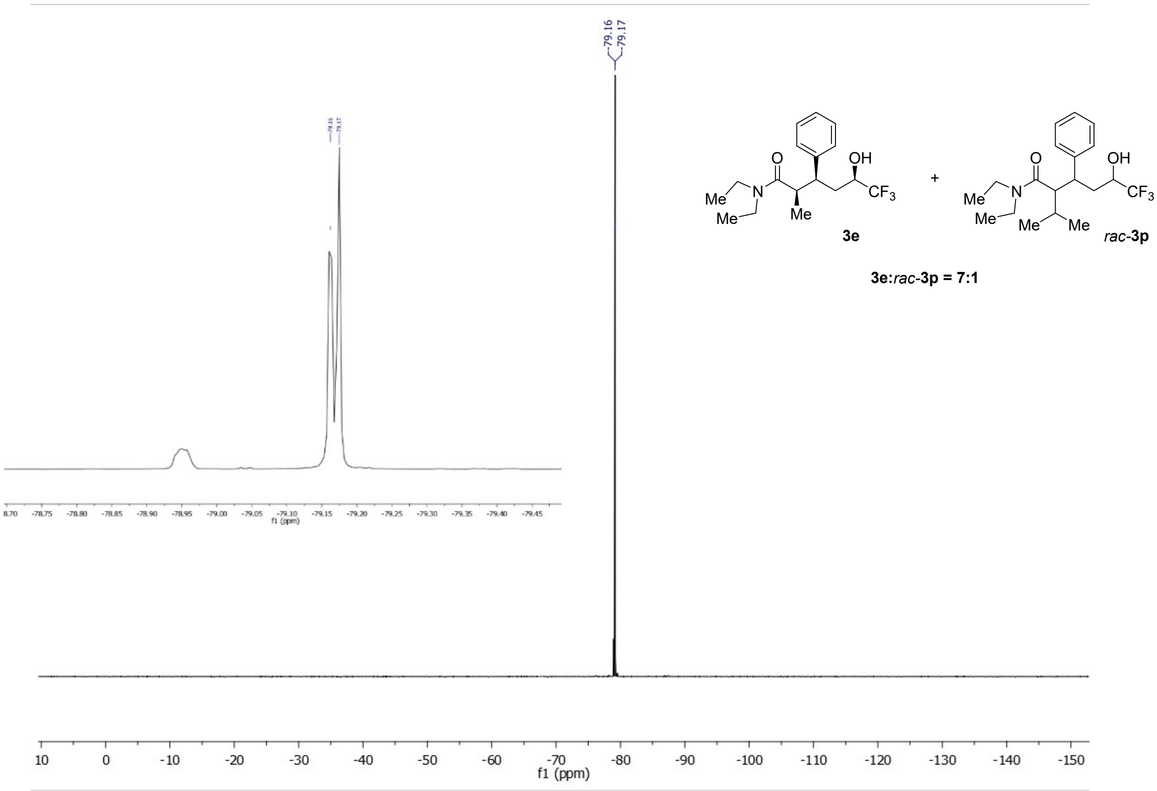

-79.16
-79.17
f1 (ppm)
3e
rac-3p
3e:rac-3p = 7:1

^{1}H – NMR:

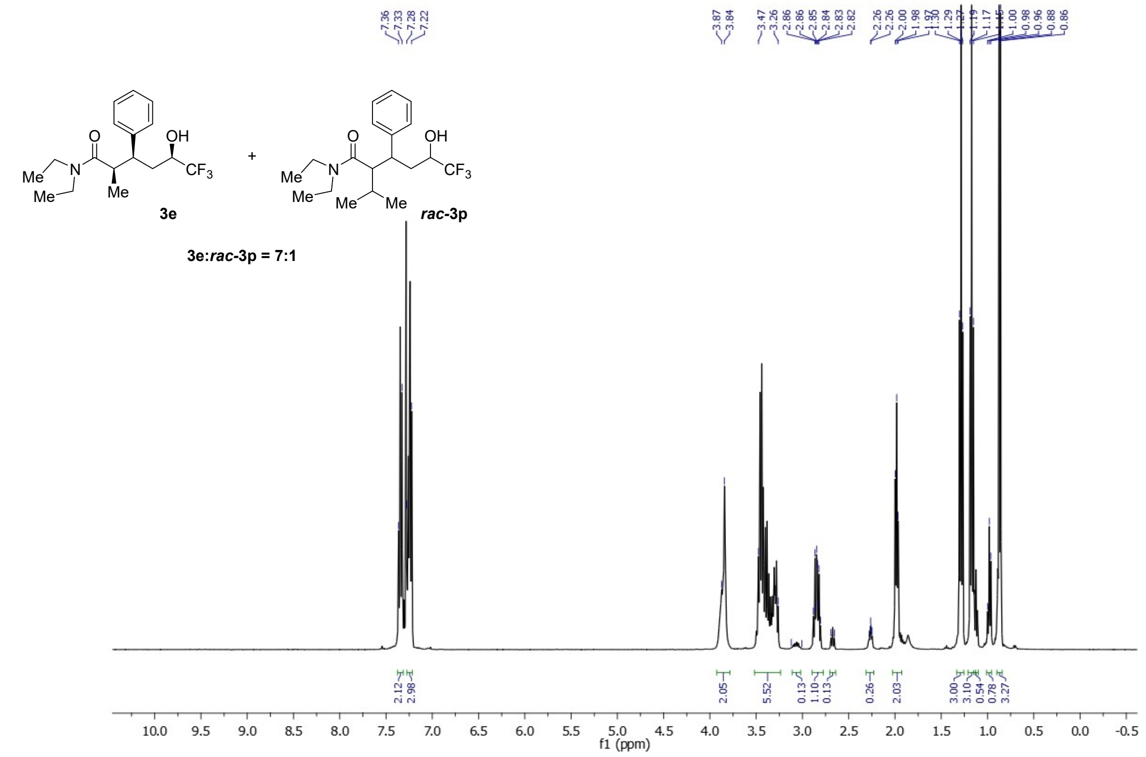

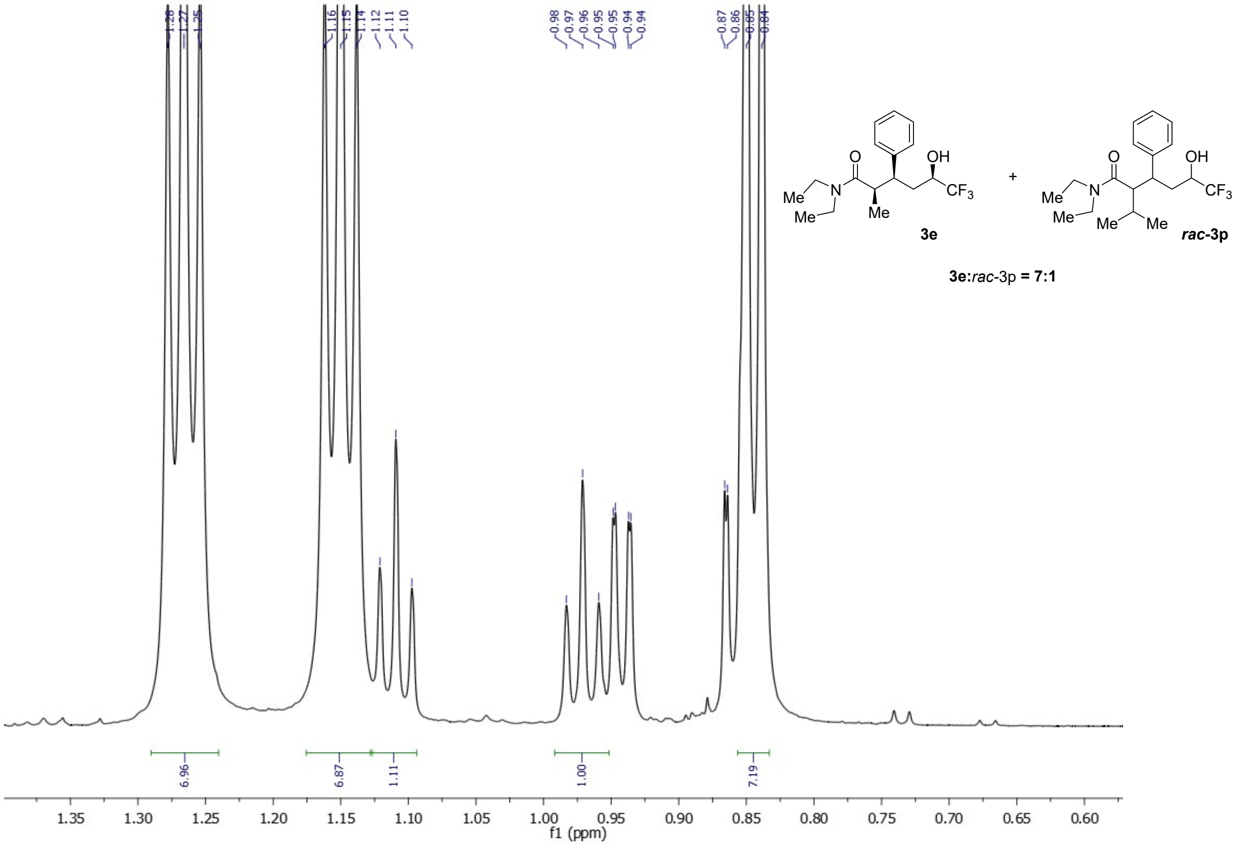

5. Post Functionalization

(2*R*,4*S*,5*R*)-1,1,1-trifluoro-5-methyl-4-phenyl-6-(pyrrolidin-1-yl)hexan-2-ol (6a)

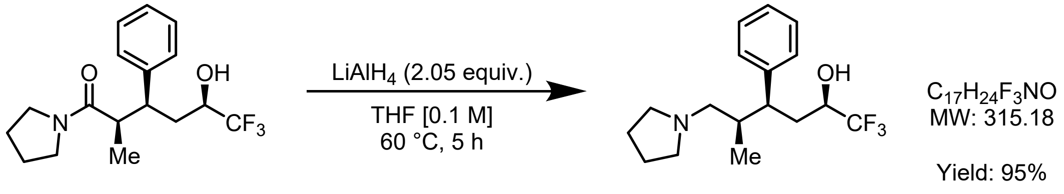

3a **6a**

A solution of $LiAlH_4$ 1M in THF (0.307 ml, 0.307 mmol, 2.05 equiv.) was suspended in THF (0.5 mL) and transferred via syringe to another vial containing a stirring solution of **3a** (49.4 mg, 0.150 mmol, 1.00 equiv.) in 1.0 mL of THF at 25 °C. The resulting suspension was heated to 60 °C for 5h before being allowed to cool to 25 °C and diluted with THF (0.5 mL). Subsequently, a saturated aq. solution of Na_2SO_4 (0.5 mL) was added dropwise while stirring vigorously. The reaction mixture was stirred for 10min, and then diluted with Et_2O (3.0 mL). The organic layers were extracted and dried over anhydrous $MgSO_4$. Finally, the solvent was removed under reduced pressure to afford the amine **6a** in a 95% yield (44.9 mg, 0.143 mmol, ee = 94%) as a colorless oil.

^{1}H NMR (600 MHz, $CDCl_3$) δ 7.34 – 7.27 (m, 4H), 7.23 (t, *J* = 7.1 Hz, 1H), 3.73 (s, 1H), 2.88 – 2.78 (m, 3H), 2.69 (s, 1H), 2.61 (s, 2H), 2.51 – 2.40 (m, 1H), 2.27 (d, *J* = 13.2 Hz, 1H), 2.21 (ddd, *J* = 14.6, 11.9, 2.9 Hz, 1H), 1.89 – 1.81 (m, 6H), 0.73 (d, *J* = 6.9 Hz, 3H) ppm.

^{13}C NMR (151 MHz, $CDCl_3$) δ 142.4, 128.6, 128.5, 126.6, 126.0 (q, *J* = 282.5 Hz), 67.1 (q, *J* = 29.6 Hz), 62.0, 54.1, 33.9, 32.0, 23.3 ppm.

^{19}F NMR (565 MHz, $CDCl_3$) δ −80.48 ppm.

IR (neat) ν_{max}: 3281, 2954, 1424, 1131, 1117, 712 cm^{-1}.

HRMS (ESI$^+$): exact mass calculated for $[M+H]^+$ ($C_{17}H_{25}F_3NO^+$) requires *m/z* 316.1883, found 316.1883 *m/z*.

$[\alpha]_D^{20}$ = 0.28 (c = 1.0, $CHCl_3$).

Determination of enantiomeric excess: ee: 94%. Method description: Chiralpak IC 250 x 4.6 mm, solvent system: *n*-heptane+0.1%IPA/IPA =98:2; flow 1 mL/min, 25 °C. Peak area 2.9% (Rt = 9.1 min), 97.1% (Rt = 11.3 min).

Enantioenriched compound:

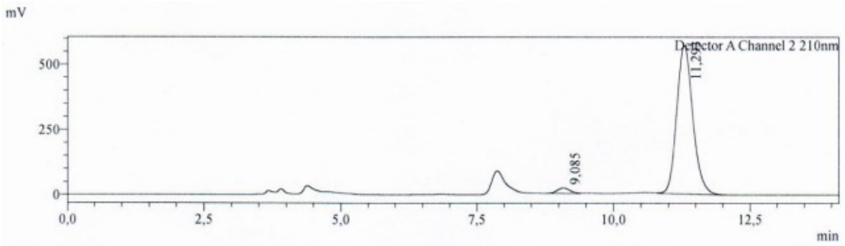

Detector A Channel 2 210nm			
Peak#	Ret. Time	Area	Area%
1	9,085	357513	2,962
2	11,293	11711505	97,038
Total		12069018	100,000

Racemic compound:

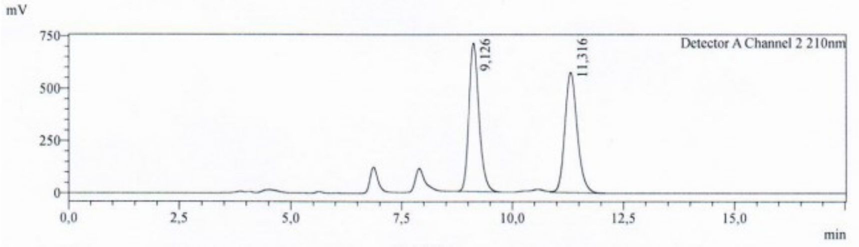

Detector A Channel 2 210nm			
Peak#	Ret. Time	Area	Area%
1	9,126	11340474	50,509
2	11,316	11111891	49,491
Total		22452365	100,000

6. Unsuccessful attempts

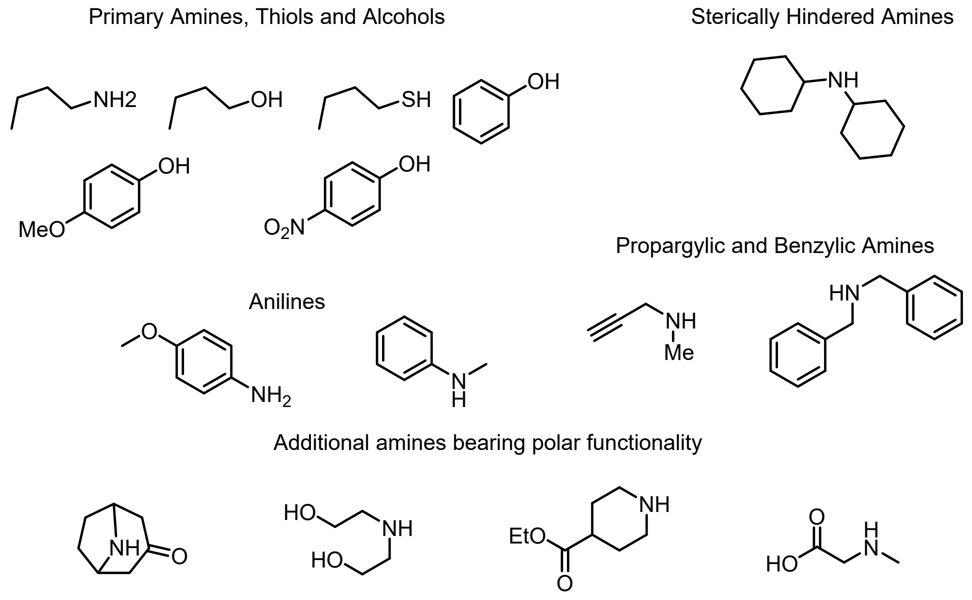

7. Computational details

7.1 Computational method

The conformational space of all molecules has been searched using meta-dynamics simulations based on tight-binding quantum chemical calculations as implemented in the software package Conformer-Rotamer Ensemble Sampling Tool (CREST). [8,9]

The structures located with the CREST have then been subjected to B3LYP-D3(BJ)/def2-SVP geometry optimization.[10–16] The nature of all stationary points (minima and transition states) was verified through the computation of the vibrational frequencies at the level applied for the geometry optimization. The thermal corrections to the Gibbs free energy were combined with the single point energies calculated at the B3LYP-D3(BJ)/def2-TZVP level of theory to yield Gibbs free energies ("G_{373}") at 373.15 K. The polarizable continuum model (PCM) with SMD parameters was applied to consider solvent (toluene) effects in both the geometries and energies.[17,18]

All energies are reported in kcal mol^{-1}. The energy profiles were constructed using each intermediate and transition state's most stable conformation (the global minimum). Free energies in solution have been corrected to a reference state of 1 mol/L at 373.15 K. The quantum chemical calculations have been performed with the Gaussian 16 program package.[19]

7.2 Cartesian coordinates (XYZ)

Cartesian coordinates for the most stabilized conformations (ΔG_{373}, toluene) as computed at the B3LYP-D3(BJ)/def2-TZVP,SMD(toluene)//B3LYP-D3(BJ)/def2 SVP,SMD(toluene) level of theory.

B

C	-1.03947	-0.83369	-0.65712
C	0.38980	-1.17404	-0.21533
C	1.34351	-0.01192	-0.58128
C	0.82100	1.31008	-0.00151
C	-0.62275	1.54852	-0.42965
H	0.38087	-1.27055	0.88365
H	-1.05899	-0.75254	-1.75759
H	0.84793	1.27663	1.09742
H	1.44339	2.15299	-0.33092
H	1.33609	0.08441	-1.67897
O	-1.44487	0.46711	-0.12334
N	-2.00329	-1.79010	-0.26655
O	-0.63125	1.85032	-1.79879
H	-1.55830	1.94221	-2.07078
C	-1.25254	2.73237	0.33687
F	-2.48282	2.99878	-0.14125
F	-0.51727	3.84499	0.19626
F	-1.36738	2.48294	1.64762
C	0.83352	-2.49934	-0.83044
H	1.82692	-2.79544	-0.46372
H	0.11735	-3.29871	-0.59360
H	0.89317	-2.41950	-1.92919
C	2.77448	-0.27093	-0.15313
C	3.10608	-0.43883	1.20106
C	3.80049	-0.35557	-1.10413
C	4.42373	-0.68539	1.59143
H	2.32444	-0.37912	1.96285
C	5.12174	-0.60107	-0.71847
H	3.55751	-0.22859	-2.16262
C	5.43798	-0.76746	0.63184
H	4.66073	-0.81414	2.65069
H	5.90617	-0.66318	-1.47711
H	6.46961	-0.96012	0.93656
C	-2.19648	-2.03697	1.17464
H	-1.61947	-2.92331	1.49464
H	-1.85607	-1.18106	1.77980
C	-3.29800	-1.74561	-0.94746
H	-3.71174	-0.71685	-0.98198
H	-3.20430	-2.10568	-1.98576
C	-3.71063	-2.26134	1.33534
H	-3.94918	-3.02123	2.09415
H	-4.19584	-1.32059	1.64185
C	-4.17647	-2.63896	-0.07662
H	-5.25175	-2.47485	-0.24100
H	-3.95543	-3.69904	-0.28541

water

O	0.00000	0.00000	0.12074
H	-0.00000	0.75636	-0.48295
H	-0.00000	-0.75636	-0.48295

B'

C	0.83039	-0.96120	-0.56486
C	-0.63466	-1.20269	-0.17816
C	-1.49161	0.02953	-0.54756

C	-0.91740	1.28256	0.12191
C	0.54992	1.46814	-0.25119
H	-0.67295	-1.32325	0.91780
H	0.89268	-0.84777	-1.65925
H	-0.99153	1.19304	1.21500
H	-1.47423	2.17844	-0.18446
H	-1.41383	0.17234	-1.63724
O	1.31199	0.31176	0.02133
N	1.70042	-1.99293	-0.16328
O	0.63566	1.84652	-1.58418
H	1.57386	1.73912	-1.86591
C	1.19093	2.55610	0.64670
F	1.16007	2.21945	1.94478
F	2.48346	2.77017	0.32192
F	0.54990	3.72406	0.51281
C	-1.14296	-2.48171	-0.84000
H	-0.49936	-3.33554	-0.58590
H	-1.14448	-2.37952	-1.93843
H	-2.17139	-2.70950	-0.52520
C	-2.95827	-0.15257	-0.21039
C	-3.92810	-0.14989	-1.22219
C	-3.38048	-0.33765	1.11615
C	-5.28212	-0.32754	-0.92288
H	-3.61479	-0.00841	-2.26010
C	-4.73140	-0.51692	1.42028
H	-2.64464	-0.34438	1.92453
C	-5.68865	-0.51260	0.40058
H	-6.02134	-0.32193	-1.72803
H	-5.03923	-0.66048	2.45923
H	-6.74617	-0.65261	0.63786
C	2.98934	-2.16515	-0.85430
H	3.21004	-1.31850	-1.52021
H	2.97388	-3.07921	-1.47810
C	1.83950	-2.27209	1.27235
H	1.01652	-2.90432	1.64627
H	1.83449	-1.33398	1.85751
C	4.01565	-2.30361	0.27395
H	4.34839	-1.30427	0.60245
H	4.90725	-2.87611	-0.02253
C	3.19799	-2.97106	1.38486
H	3.08882	-4.04859	1.17649
H	3.64258	-2.86571	2.38552
O	3.26559	1.10656	-1.81623
H	2.96229	0.74192	-0.96158
H	3.88519	1.80809	-1.56696

TS_B'C

C	0.90809	-0.94613	-0.54616
C	-0.53549	-1.18354	-0.09819
C	-1.44235	-0.00234	-0.51293
C	-0.94840	1.29390	0.13360
C	0.44043	1.67369	-0.36255
H	-0.54081	-1.24480	1.00333
H	0.94135	-0.87917	-1.64453
H	-0.94723	1.19406	1.22839
H	-1.61340	2.12938	-0.12799
H	-1.35363	0.12364	-1.60389
O	1.37919	0.40477	-0.04858
N	1.81788	-1.91652	-0.11595
O	0.54813	1.98852	-1.61916
H	1.83212	1.68308	-1.94164
C	1.09841	2.70217	0.60371
F	0.40748	3.85069	0.57121
F	1.13432	2.28945	1.88152

F	2.36840	2.98870	0.24585
C	-1.01907	-2.51565	-0.67342
H	-1.06975	-2.46963	-1.77426
H	-2.02392	-2.76274	-0.30394
H	-0.33481	-3.33199	-0.40352
C	-2.90289	-0.25043	-0.19074
C	-3.33443	-0.42296	1.13448
C	-3.85692	-0.31812	-1.21500
C	-4.67953	-0.65904	1.42528
H	-2.61057	-0.37473	1.95215
C	-5.20518	-0.55257	-0.92892
H	-3.53566	-0.18545	-2.25162
C	-5.62127	-0.72504	0.39327
H	-4.99519	-0.79148	2.46337
H	-5.93244	-0.60083	-1.74347
H	-6.67431	-0.90914	0.62011
C	2.00419	-2.16018	1.32272
H	1.34918	-2.98070	1.66905
H	1.75932	-1.26398	1.91516
C	3.05645	-2.15503	-0.86201
H	3.62416	-1.22241	-1.04630
H	2.83832	-2.60344	-1.84525
C	3.48751	-2.53789	1.45077
H	3.67519	-3.25102	2.26678
H	4.08591	-1.63392	1.65128
C	3.83723	-3.08742	0.06223
H	4.91657	-3.08947	-0.14919
H	3.46639	-4.12047	-0.04464
H	2.24589	0.67433	-0.77405
O	2.80106	1.15804	-1.81665
H	3.43271	1.83567	-1.53261

C

C	0.57258	-1.92334	-0.49357
C	-0.85630	-1.55140	-0.03561
C	-1.31715	-0.14679	-0.50163
C	-0.38360	0.98233	-0.04077
C	0.90537	1.08827	-0.80866
H	-0.86387	-1.55774	1.06639
H	0.73643	-2.98272	-0.19721
H	-0.17964	0.93244	1.03694
H	-0.88191	1.95610	-0.20708
H	-1.31792	-0.14456	-1.60253
O	1.05177	0.80847	-1.98420
N	1.62415	-1.11424	0.15089
O	0.70848	-1.90502	-1.88339
H	0.68988	-0.97671	-2.17990
C	1.98539	1.98526	-0.15595
F	3.16932	1.85014	-0.75127
F	1.59986	3.27039	-0.28835
F	2.14200	1.75484	1.15772
C	-1.81823	-2.63733	-0.52419
H	-1.50105	-3.62912	-0.16469
H	-1.83196	-2.67188	-1.62436
H	-2.84337	-2.46210	-0.16998
C	-2.73743	0.14744	-0.05084
C	-3.05156	0.26643	1.31248
C	-3.76745	0.30395	-0.98792
C	-4.35865	0.53103	1.72629
H	-2.26576	0.14756	2.06311
C	-5.07775	0.56895	-0.57856
H	-3.53798	0.21120	-2.05289
C	-5.37809	0.68283	0.78090
H	-4.58332	0.61897	2.79238

H	-5.86663	0.68547	-1.32604
H	-6.40156	0.88901	1.10359
C	1.68373	-1.30564	1.60893
H	1.40470	-2.34905	1.86132
H	0.96529	-0.64947	2.12134
C	2.96377	-1.43864	-0.36779
H	3.11635	-0.97488	-1.35172
H	3.06150	-2.53560	-0.50209
C	3.14167	-1.02737	2.02583
H	3.51294	-1.83940	2.66791
H	3.22162	-0.09572	2.60195
C	3.93299	-0.93414	0.70281
H	4.22354	0.10368	0.49716
H	4.85523	-1.53277	0.71742

TS_BC

C	0.93128	-1.21191	-0.79849
C	-0.36544	-1.21206	-0.02852
C	-1.27345	-0.02811	-0.46179
C	-0.82548	1.30970	0.16058
C	0.70272	1.47725	0.31663
H	-0.14153	-1.10423	1.04013
H	0.83303	-1.29813	-1.88392
H	-1.22150	1.39942	1.18164
H	-1.26514	2.12386	-0.43323
H	-1.17657	0.05897	-1.55530
O	1.26853	0.99350	1.33506
N	2.02902	-1.74427	-0.29939
O	1.23053	0.74120	-1.07601
H	2.19342	0.78714	-0.95724
C	1.15760	2.92684	-0.02264
F	0.64619	3.42535	-1.16702
F	0.78954	3.76733	0.96249
F	2.50067	2.99980	-0.13086
C	-0.99860	-2.59286	-0.28290
H	-1.89240	-2.72981	0.33962
H	-0.29037	-3.40156	-0.04485
H	-1.30402	-2.70529	-1.33574
C	-2.74083	-0.27763	-0.16514
C	-3.19893	-0.42878	1.15453
C	-3.67507	-0.36630	-1.20532
C	-4.54751	-0.66776	1.42277
H	-2.48998	-0.36218	1.98407
C	-5.02885	-0.59997	-0.94212
H	-3.33578	-0.24881	-2.23829
C	-5.46967	-0.75350	0.37376
H	-4.88259	-0.78515	2.45657
H	-5.74018	-0.66135	-1.76979
H	-6.52642	-0.93663	0.58350
C	2.34343	-1.83362	1.14422
H	1.83712	-2.71469	1.57306
H	1.98644	-0.91441	1.63232
C	3.20606	-2.05758	-1.13193
H	3.63125	-1.12352	-1.54042
H	2.91702	-2.69947	-1.97704
C	3.86512	-1.99030	1.16511
H	4.21738	-2.53003	2.05529
H	4.33669	-0.99393	1.16618
C	4.17274	-2.71753	-0.14987
H	5.21975	-2.62407	-0.47116
H	3.94039	-3.79128	-0.05685

TS_CD_S

C	0.96063	-0.71387	1.07627

C	-0.58671	-0.82847	1.02497
C	-1.16971	0.10023	-0.07350
C	-0.74741	1.56435	0.19911
C	0.74150	1.71700	0.54390
H	-0.89393	-0.40653	1.99406
H	1.07170	0.38453	0.40572
H	-1.28997	1.95080	1.07288
H	-1.01456	2.18926	-0.66686
H	-0.73298	-0.20331	-1.03768
O	1.11113	2.04854	1.71116
N	1.75407	-1.63185	0.40115
O	1.45408	-0.35883	2.27682
H	1.33001	0.65432	2.34648
C	1.64853	2.18561	-0.61233
F	1.54468	3.50864	-0.80224
F	2.93673	1.90380	-0.36928
F	1.32172	1.58745	-1.78153
C	-1.09773	-2.26934	0.97659
H	-0.50382	-2.91382	1.64200
H	-1.06401	-2.69702	-0.03533
H	-2.14543	-2.30941	1.30600
C	-2.67327	-0.02565	-0.20159
C	-3.52146	0.30630	0.86734
C	-3.25060	-0.49402	-1.38938
C	-4.90582	0.17117	0.75104
H	-3.09525	0.67064	1.80557
C	-4.63700	-0.62913	-1.51147
H	-2.60297	-0.75753	-2.23018
C	-5.46955	-0.29730	-0.44053
H	-5.54900	0.43284	1.59507
H	-5.06651	-0.99548	-2.44733
H	-6.55334	-0.40252	-0.53214
C	1.49671	-1.93887	-1.01125
H	1.37667	-1.00523	-1.59485
H	0.58587	-2.53685	-1.14036
C	3.21012	-1.62401	0.66747
H	3.45250	-2.39400	1.41915
H	3.52333	-0.65559	1.08094
C	2.75927	-2.67697	-1.44961
H	2.89021	-2.66744	-2.54134
H	2.71355	-3.72801	-1.11964
C	3.85393	-1.92129	-0.69181
H	4.79410	-2.48353	-0.59790
H	4.07695	-0.97442	-1.21006

TS_CD_R

C	0.90210	-0.86430	-0.71213
C	-0.53766	-1.10264	-0.22896
C	-1.41586	0.13379	-0.54894
C	-0.78372	1.42238	0.02290
C	0.60673	1.62525	-0.56835
H	-0.51328	-1.22334	0.86477
H	1.06054	0.34491	-0.25224
H	-0.73575	1.37334	1.12074
H	-1.40404	2.29018	-0.25200
H	-1.43614	0.24528	-1.64393
O	0.75556	1.76565	-1.82046
N	1.90301	-1.62859	-0.13905
O	1.04845	-0.68295	-2.03606
H	0.90347	0.31618	-2.22450
C	1.62423	2.35781	0.32770
F	1.65955	1.84306	1.57925
F	2.86259	2.27461	-0.17062
F	1.30899	3.65756	0.44419

C	-1.07230	-2.38941	-0.86069
H	-0.40288	-3.23409	-0.63709
H	-1.13044	-2.28527	-1.95450
H	-2.07528	-2.62906	-0.47988
C	-2.84406	-0.04378	-0.07753
C	-3.89721	-0.08576	-1.00135
C	-3.14720	-0.17956	1.28670
C	-5.21851	-0.25798	-0.57832
H	-3.67459	0.01649	-2.06684
C	-4.46512	-0.35271	1.71412
H	-2.34404	-0.15023	2.02763
C	-5.50690	-0.39251	0.78180
H	-6.02491	-0.28752	-1.31550
H	-4.68079	-0.45620	2.78064
H	-6.53871	-0.52734	1.11551
C	1.97554	-1.79499	1.32081
H	1.47889	-2.73112	1.62964
H	1.47207	-0.96315	1.83930
C	3.24317	-1.60900	-0.73940
H	3.57981	-0.56648	-0.90075
H	3.23873	-2.10965	-1.71731
C	3.48084	-1.81847	1.62445
H	3.72545	-2.44701	2.49276
H	3.82755	-0.79480	1.84029
C	4.10197	-2.30771	0.31205
H	5.16883	-2.06048	0.21434
H	3.99594	-3.40141	0.22001

D_S

C	1.675080	0.510850	0.704720
C	0.457270	1.345840	0.303270
C	-0.823860	0.486610	0.413640
C	-0.706760	-0.818820	-0.411180
C	-1.132760	-2.045020	0.403490
H	0.573730	1.671220	-0.742400
H	-2.225940	-2.011270	0.572020
H	0.330670	-0.979060	-0.742450
H	-1.317450	-0.766020	-1.322720
H	-0.900900	0.201340	1.471430
O	1.592730	-0.394290	1.546670
N	2.859410	0.811000	0.134730
O	-0.492710	-2.149890	1.640130
H	0.317980	-1.591960	1.633910
C	-0.891190	-3.324550	-0.396460
F	0.413690	-3.498490	-0.685980
F	-1.299360	-4.413490	0.270140
F	-1.558850	-3.303190	-1.571350
C	0.393110	2.586970	1.205900
H	1.318180	3.180760	1.147700
H	0.252400	2.286320	2.256170
H	-0.446920	3.235900	0.919710
C	-2.070570	1.277200	0.068780
C	-2.279830	1.779340	-1.226430
C	-3.042040	1.534400	1.045760
C	-3.423250	2.518860	-1.533500
H	-1.539450	1.587860	-2.008150
C	-4.191210	2.271600	0.742600
H	-2.893040	1.150150	2.058390
C	-4.385010	2.767680	-0.548330
H	-3.566800	2.901560	-2.547260
H	-4.937270	2.457350	1.519450
H	-5.281800	3.344140	-0.788460
C	4.067730	0.042790	0.469510
H	3.833850	-1.030150	0.507440

H	4.433940	0.333830	1.469650
C	3.119200	1.832330	-0.890170
H	2.687730	2.804220	-0.613350
H	2.679500	1.529010	-1.856880
C	5.053190	0.431100	-0.634130
H	4.906970	-0.216640	-1.514390
H	6.100860	0.334500	-0.315610
C	4.648930	1.871630	-0.968140
H	5.043330	2.561580	-0.203980
H	5.007010	2.215320	-1.949150

D_R

C	1.12185	-1.15696	-0.81068
C	-0.24357	-1.31174	-0.14200
C	-1.16484	-0.10423	-0.45647
C	-0.71818	1.20908	0.21703
C	0.62848	1.76327	-0.26771
H	-0.11524	-1.37180	0.94896
H	1.45347	1.12547	0.10550
H	-0.68112	1.08048	1.31018
H	-1.48414	1.97148	0.00745
H	-1.12555	0.05136	-1.54409
O	0.68826	1.92805	-1.65262
N	2.20560	-1.60822	-0.14745
O	1.22990	-0.67778	-1.94899
H	0.88243	1.03865	-2.01910
C	0.91015	3.12472	0.35783
F	0.89898	3.04108	1.70715
F	2.11735	3.59010	0.00260
F	0.00305	4.05054	0.00960
C	-0.82536	-2.64546	-0.64322
H	-0.12060	-3.47363	-0.46762
H	-1.01950	-2.59432	-1.72567
H	-1.76886	-2.88551	-0.13467
C	-2.60848	-0.39916	-0.09073
C	-3.59071	-0.48446	-1.08650
C	-2.99392	-0.60292	1.24419
C	-4.92126	-0.76711	-0.76328
H	-3.30511	-0.32776	-2.13010
C	-4.32093	-0.88797	1.57226
H	-2.24759	-0.54018	2.04049
C	-5.29097	-0.97154	0.56808
H	-5.67113	-0.82789	-1.55619
H	-4.60043	-1.04427	2.61740
H	-6.33007	-1.19321	0.82372
C	2.23747	-2.22328	1.18761
H	1.47768	-3.01152	1.28591
H	2.04397	-1.46497	1.96699
C	3.54719	-1.49333	-0.73781
H	3.68084	-0.49655	-1.18106
H	3.66591	-2.23127	-1.55031
C	3.66589	-2.76865	1.28920
H	3.71199	-3.77689	0.84564
H	4.01027	-2.84280	2.33060
C	4.47843	-1.78411	0.44027
H	4.66561	-0.85827	1.00918
H	5.45083	-2.18360	0.11918

8. X-Ray Crystallographic data

Single crystal X-ray diffraction data were collected with a Stadivari Diffractometer (STOE & Cie GmbH, Germany) equipped with an EIGER2 R500 detector (Dectris Ltd, Switzerland). Data were processed and scaled with the STOE software suite X-Area (STOE & Cie GmbH). Structures were solved with SHELXT [20] and refined with SHELXL[21] or Olex2[22]. Model building was done with Olex2 or ShelXle [23]. The structures were validated with CHECKCIF (https://checkcif.iucr.org/). See the respective CIF file for exact versions and more details.

**Sample**	**Machine**	**Source**	**Temp.**	**Detector Distance**	**Time/ Frame**	**#Frames**	**Frame width**	**CCDC**
	Bruker		[K]	[mm]	[s]		[°]	
**3h**	D8	Mo	100	40	10	623	0.360	2335899
**3k**	D8	Mo	100	40	10	623	0.360	2335900
**4r**	D8	Mo	100	40	10	623	0.360	2351935

Supplementary Table S2: Crystal data and structure refinement for **3h** (CCDC = 2335899).

Identification code	**3h**	
Empirical formula	C17 H22 F3 N O3	
Formula weight	345.36	
Temperature	100 K	
Wavelength	1.54186 Å	
Crystal system	Orthorhombic	
Space group	$P2_12_12_1$	
Unit cell dimensions	a = 5.9815(40) Å	α= 90.0011(165)°.
	b = 12.8172(74) Å	β= 90.0028(154)°.
	c = 21.4494(60) Å	γ = 90.0211(235)°.
Volume	1644.4(15) Å^3	
Z	4	
Density (calculated)	1.395 Mg/m^3	
Absorption coefficient	1.003 mm^{-1}	
F(000)	728	
Crystal size	0.200 x 0.030 x 0.010 mm^3	
Theta range for data collection	5.377 to 58.928°	
Index ranges	-6<=h<=6, -14<=k<=14, -23<=l<=22	
Reflections collected	20796	
Independent reflections	2354 [R(int) = 0.0471]	
Completeness to theta = 58.928°	99.3 %	
Absorption correction	Empirical	
Max. and min. transmission	0.9900 and 0.8187	
Refinement method	Full-matrix least-squares on F^2	
Data / restraints / parameters	2354 / 0 / 220	
Goodness-of-fit on F^2	1.106	
Final R indices [I>2sigma(I)]	R1 = 0.0549, wR2 = 0.1361	
R indices (all data)	R1 = 0.0577, wR2 = 0.1398	

Absolute structure parameter	-0.02(7)	
Extinction coefficient	0.022(3)	
Largest diff. peak and hole	0.423 and -0.345 e.Å^{-3}	

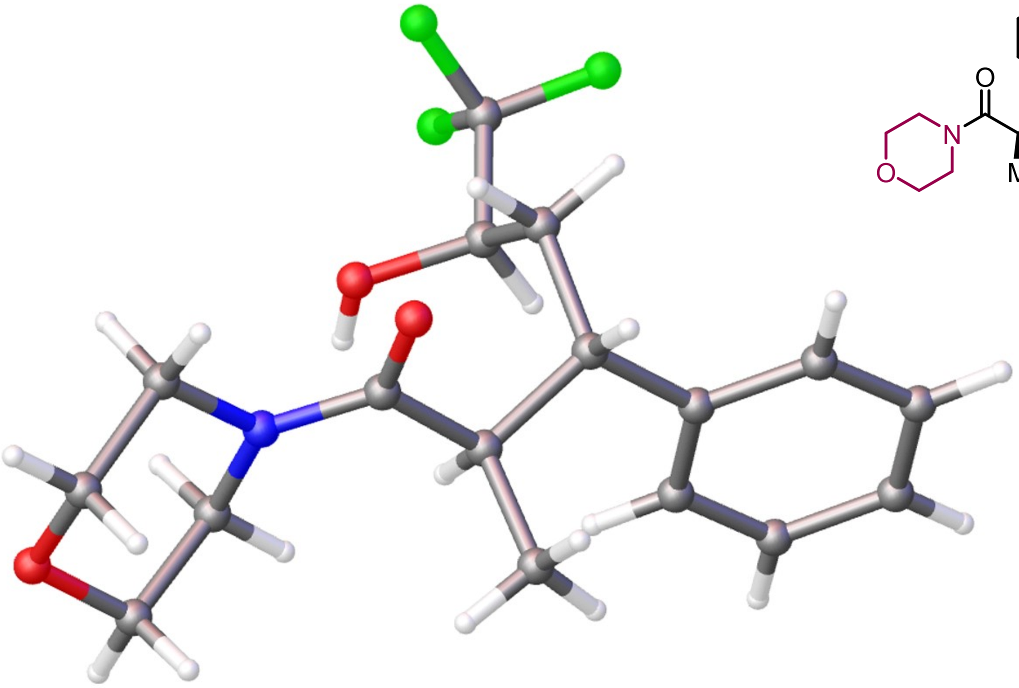

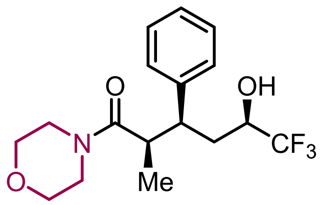

Supplementary Table S3: Crystal data and structure refinement for **3k** (CCDC = 2335900).

Identification code	**3k**	
Empirical formula	C17 H21 Br F3 N O2	
Formula weight	408.25	
Temperature	100 K	
Wavelength	0.71073 Å	
Crystal system	Monoclinic	
Space group	$P2_1/c$	
Unit cell dimensions	a = 5.8629(7) Å	α= 90°.
	b = 16.8270(11) Å	β= 95.767(8)°.
	c = 17.5656(16) Å	γ = 90°.
Volume	1724.2(3) $Å^3$	
Z	4	
Density (calculated)	1.573 Mg/m^3	
Absorption coefficient	2.424 mm^{-1}	
F(000)	832	
Crystal size	0.090 x 0.060 x 0.010 mm^3	
Theta range for data collection	3.361 to 23.256°	
Index ranges	-6<=h<=6, -18<=k<=18, -19<=l<=19	
Reflections collected	34707	
Independent reflections	2477 [R(int) = 0.1642]	
Completeness to theta = 58.928°	99.7 %	
Absorption correction	Semi-empirical from equivalents	
Max. and min. transmission	0.9189 and 0.5190	
Refinement method	Full-matrix least-squares on F^2	
Data / restraints / parameters	2477 / 0 / 219	
Goodness-of-fit on F^2	0.901	
Final R indices [I>2sigma(I)]	R1 = 0.0543, wR2 = 0.1134	
R indices (all data)	R1 = 0.1242, wR2 = 0.1264	

Absolute structure parameter	n/a	
Largest diff. peak and hole	0.898 and -0.837 e.Å^{-3}	

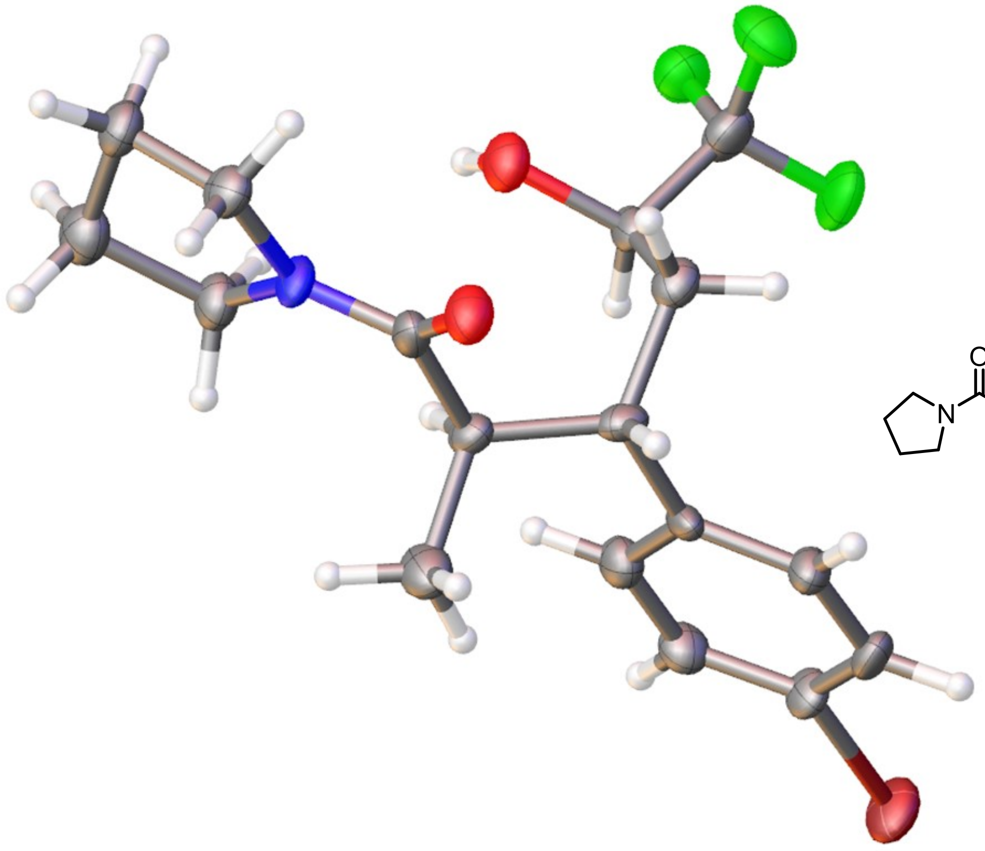

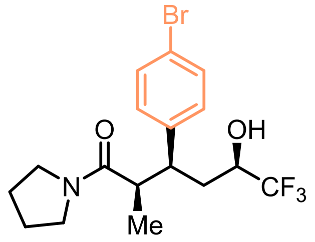

Supplementary Table S4: Crystal data and structure refinement for **4r** (CCDC = 2351935).

Identification code	**4r**	
Empirical formula	C20 H18 Br F3 N2 O2	
Formula weight	455.27	
Temperature	100 K	
Wavelength	0.71073 Å	
Crystal system	Monoclinic	
Space group	P 1 21 1	
Unit cell dimensions	a = 6.2014(5) Å	α= 90°.
	b = 7.6636(6) Å	β= 93.802(7)°.
	c = 20.8000(18) Å	γ = 90°.
Volume	986.35(14) $Å^3$	
Z	2	
Density (calculated)	1.533 Mg/m^3	
Absorption coefficient	2.129 mm^{-1}	
F(000)	460	
Crystal size	0.12 x 0.067 x 0.01 mm^3	
Theta range for data collection	1.962 to 30.688°.	
Index ranges	-8<=h<=6, -10<=k<=11, -29<=l<=27	
Reflections collected	9937	
Independent reflections	5214 [R(int) = 0.0438]	
Completeness to theta = 58.928°	99.9 %	
Absorption correction	Semi-empirical from equivalents	
Max. and min. transmission	0.9371 and 0.6469	
Refinement method	Full-matrix least-squares on F^2	
Data / restraints / parameters	5214 / 1 / 255	
Goodness-of-fit on F^2	1.059	
Final R indices [I>2sigma(I)]	R1 = 0.0455, wR2 = 0.0993	
R indices (all data)	R1 = 0.1236, wR2 = 0.1466	

Absolute structure parameter	-0.004(12)	
Extinction coefficient	n/a	
Largest diff. peak and hole	1.230 and -2.349 e.Å^{-3}	

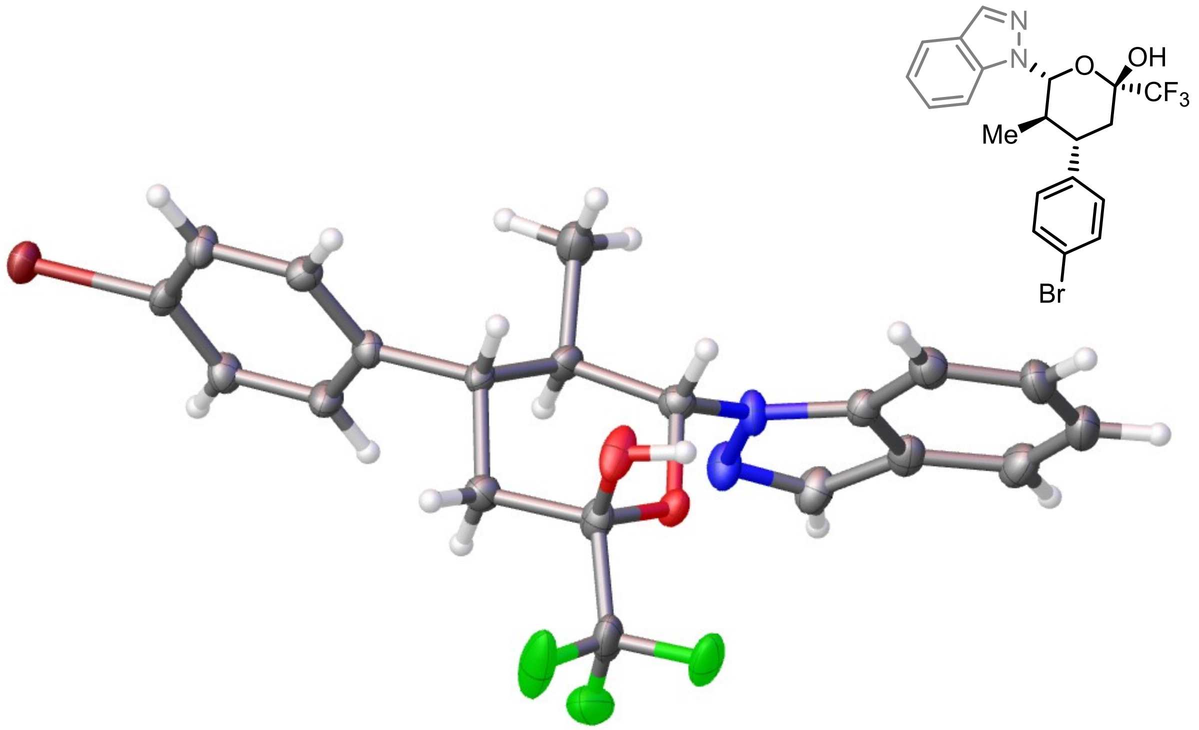

9. NMR Spectra

9.1 Starting Materials

Nitropropane-1,1-d$_2$ (S2a)

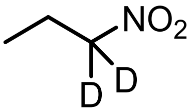

D = 85%

^{1}H NMR - 600 MHz

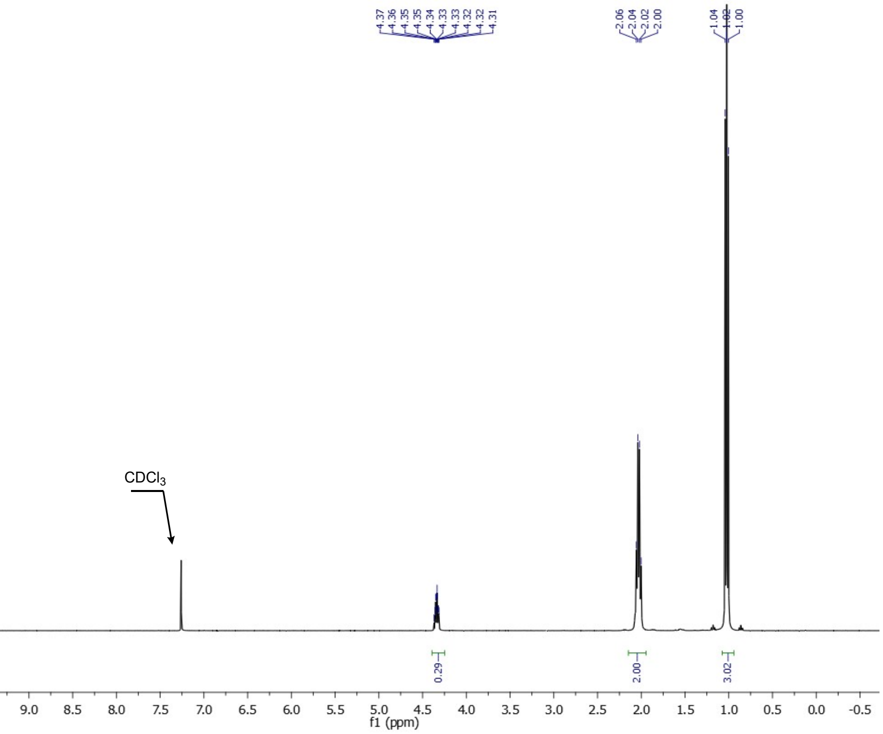

1d-propionaldehyde (S3a) ^{1}H NMR (600 MHz, CDCl$_3$) δ

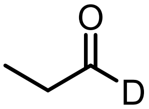

D = 96%

^{1}H NMR - 600 MHz

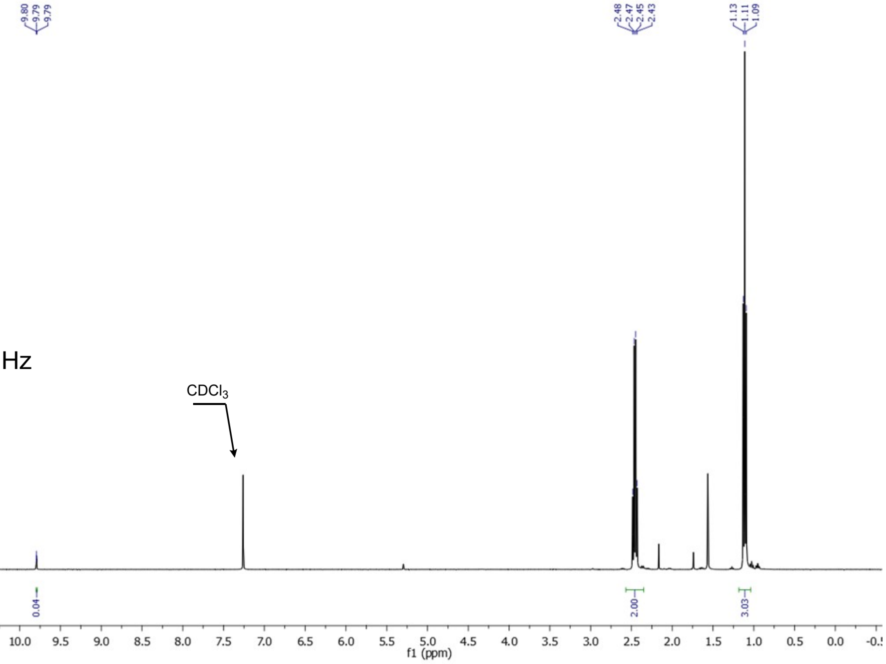

(2*S*,4*S*,5*R*)-5-methyl-4-phenyl-2-(trifluoromethyl)tetrahydro-2*H*-pyran-2,6-diol (1a)

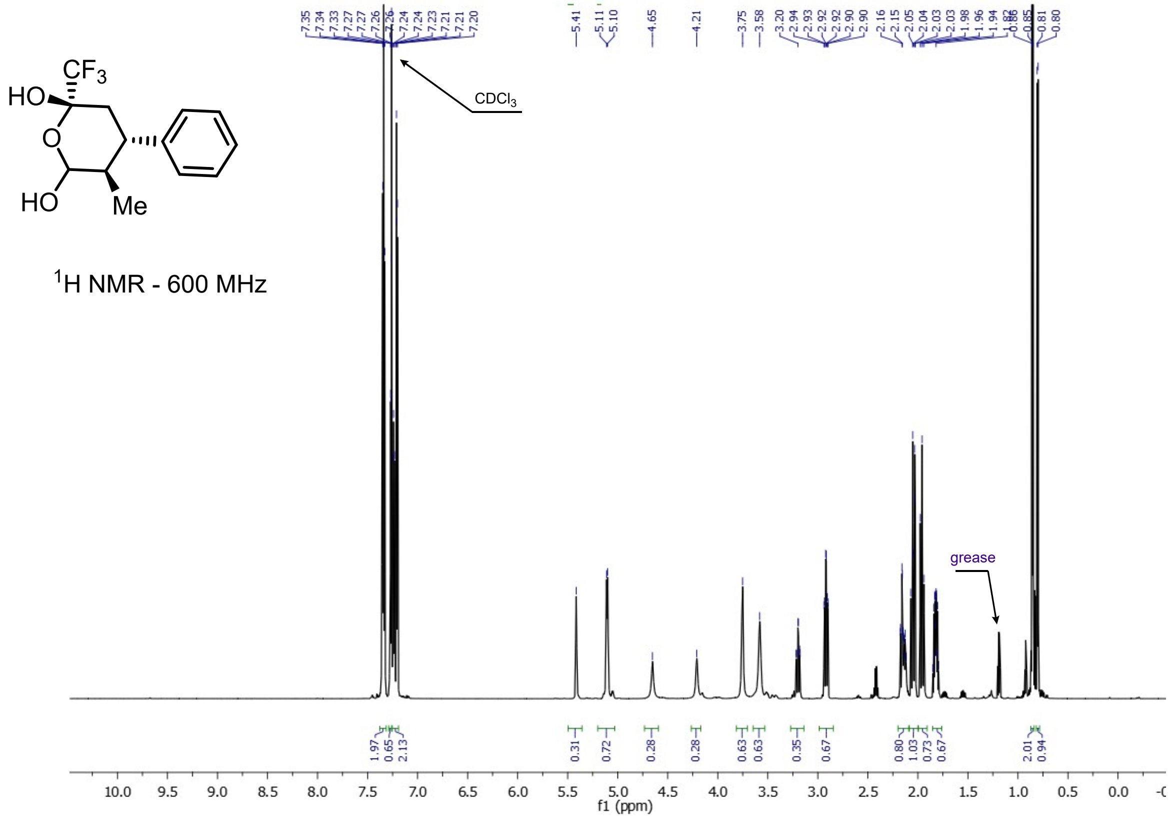

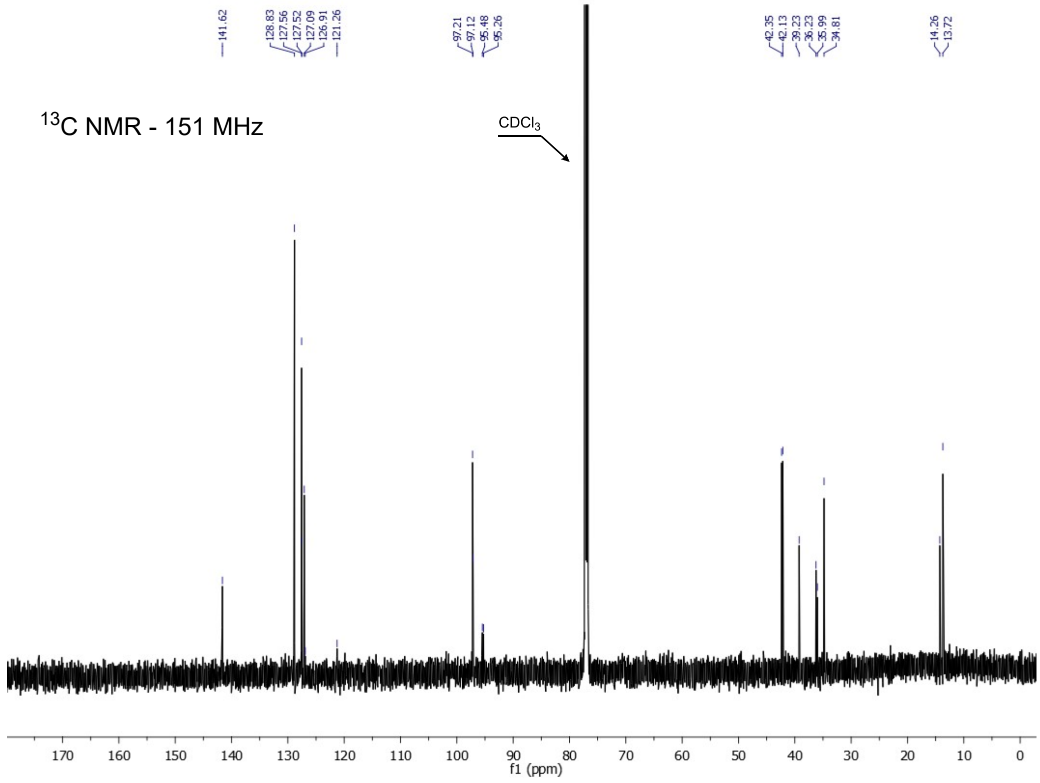

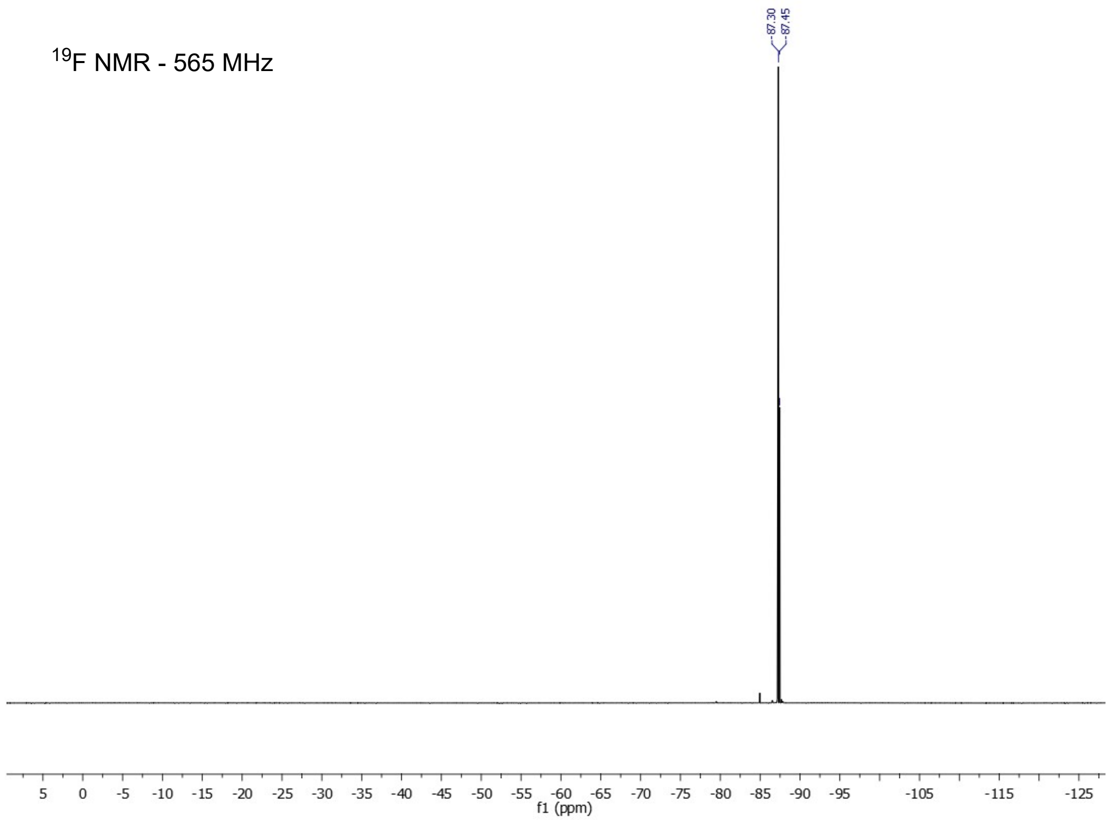

(2*S*,4*S*,5*R*)-5-methyl-4-(m-tolyl)-2-(trifluoromethyl)tetrahydro-2H-pyran-2,6-diol (1b)

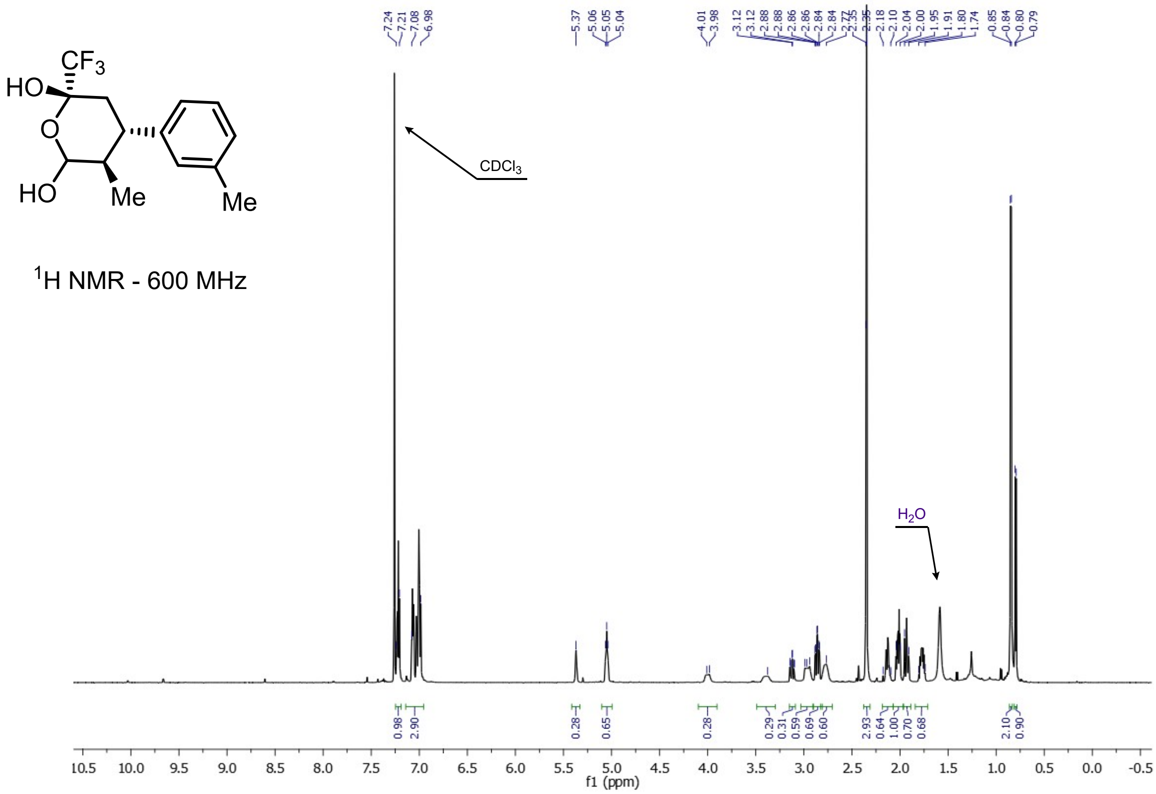

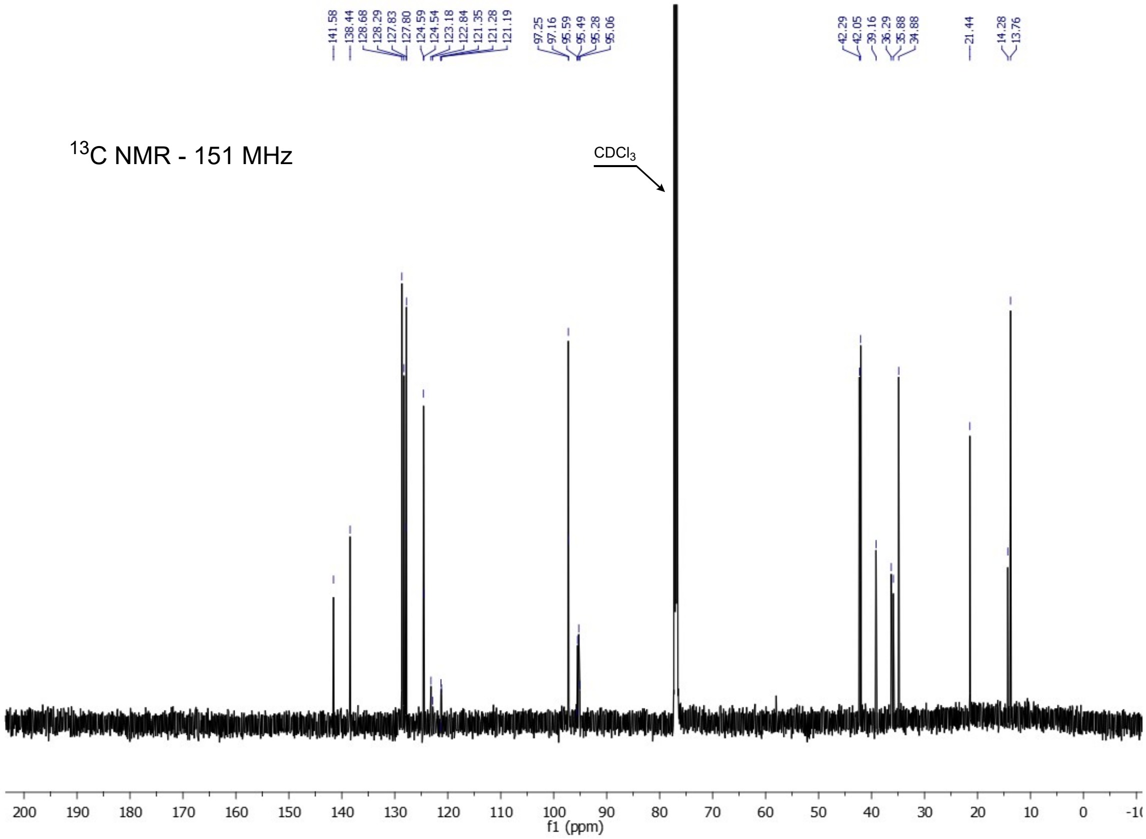
141.58
138.44
128.68
128.29
127.83
127.80
124.59
124.54
123.18
122.84
121.35
121.28
121.19
97.25
97.16
95.59
95.49
95.28
95.06
42.29
42.05
39.16
36.29
35.88
34.88
21.44
14.28
13.76
^{13}C NMR - 151 MHz
$CDCl_3$
f1 (ppm)

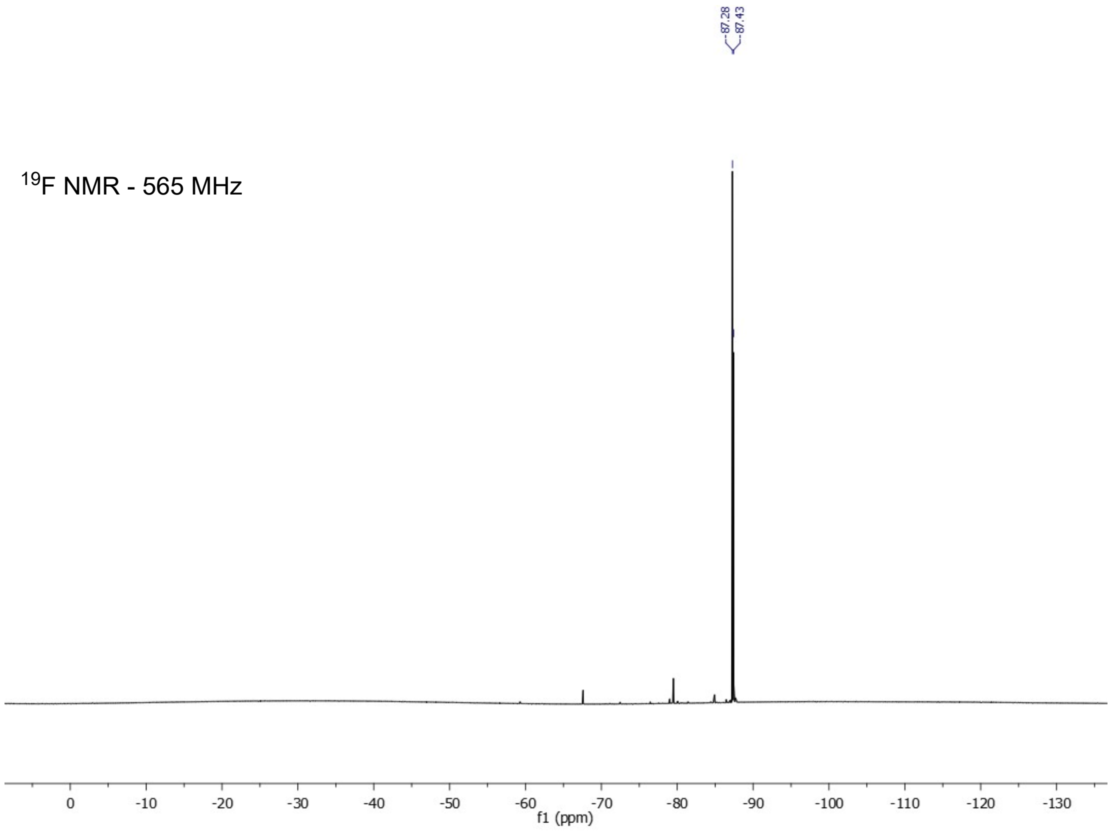
-87.28
-87.43
^{19}F NMR - 565 MHz
f1 (ppm)

(2*S*,4*S*,5*R*)-4-(4-methoxyphenyl)-5-methyl-2-(trifluoromethyl)tetrahydro-2H-pyran-2,6-diol (1c)

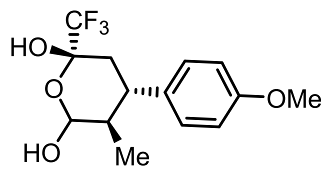

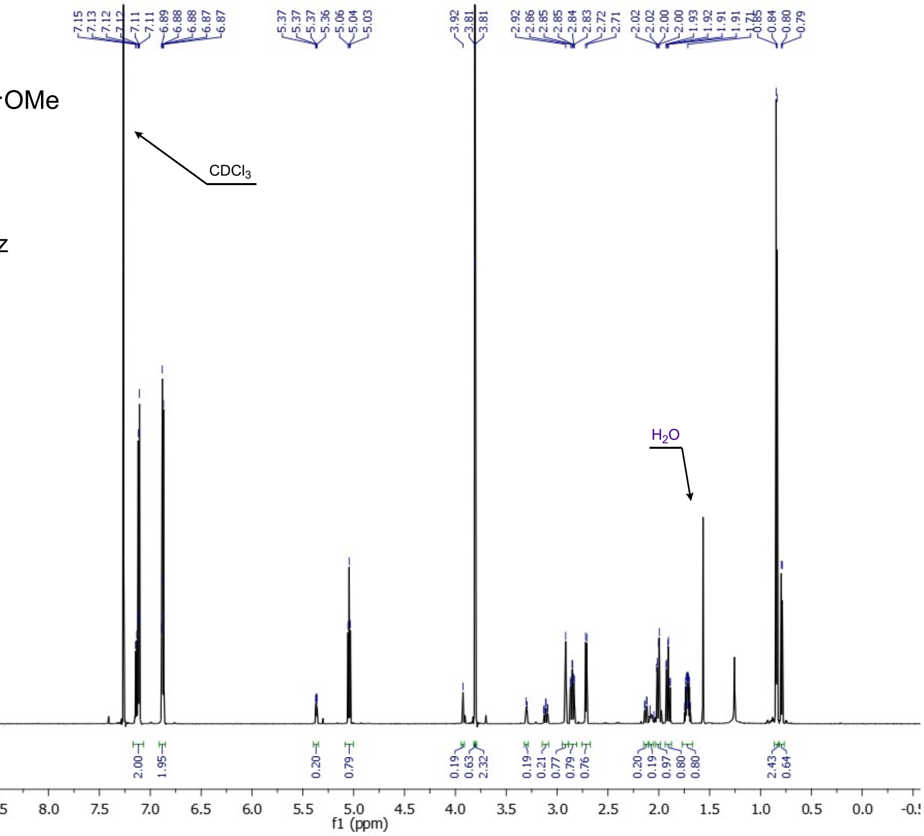

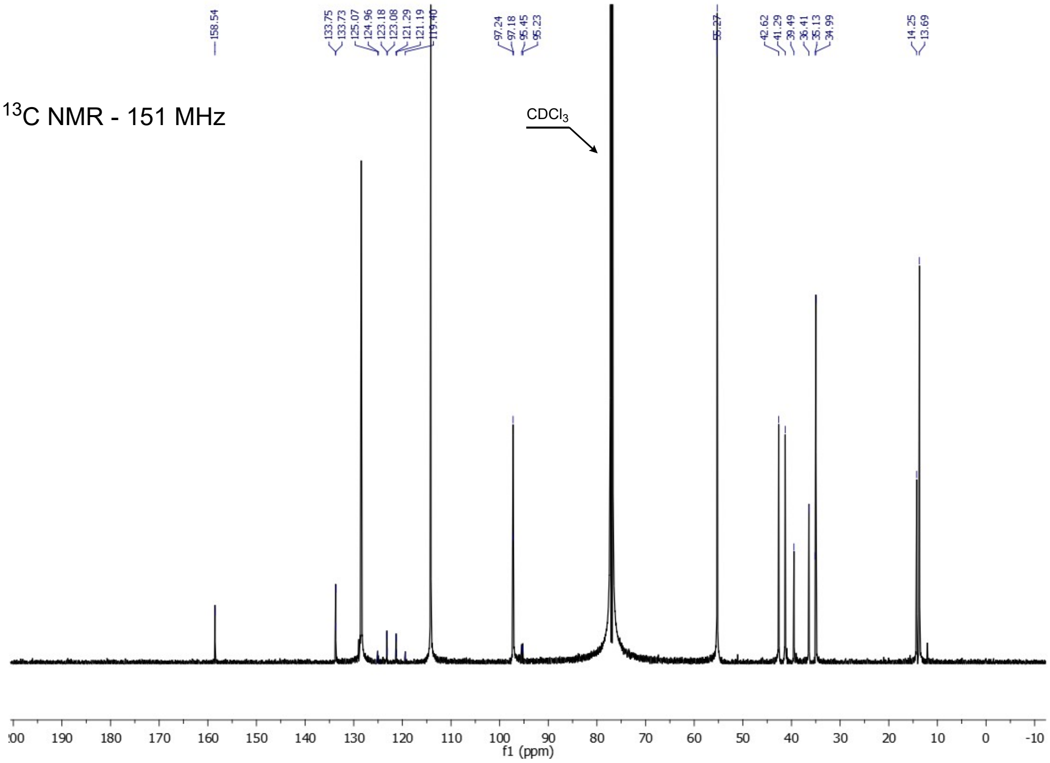

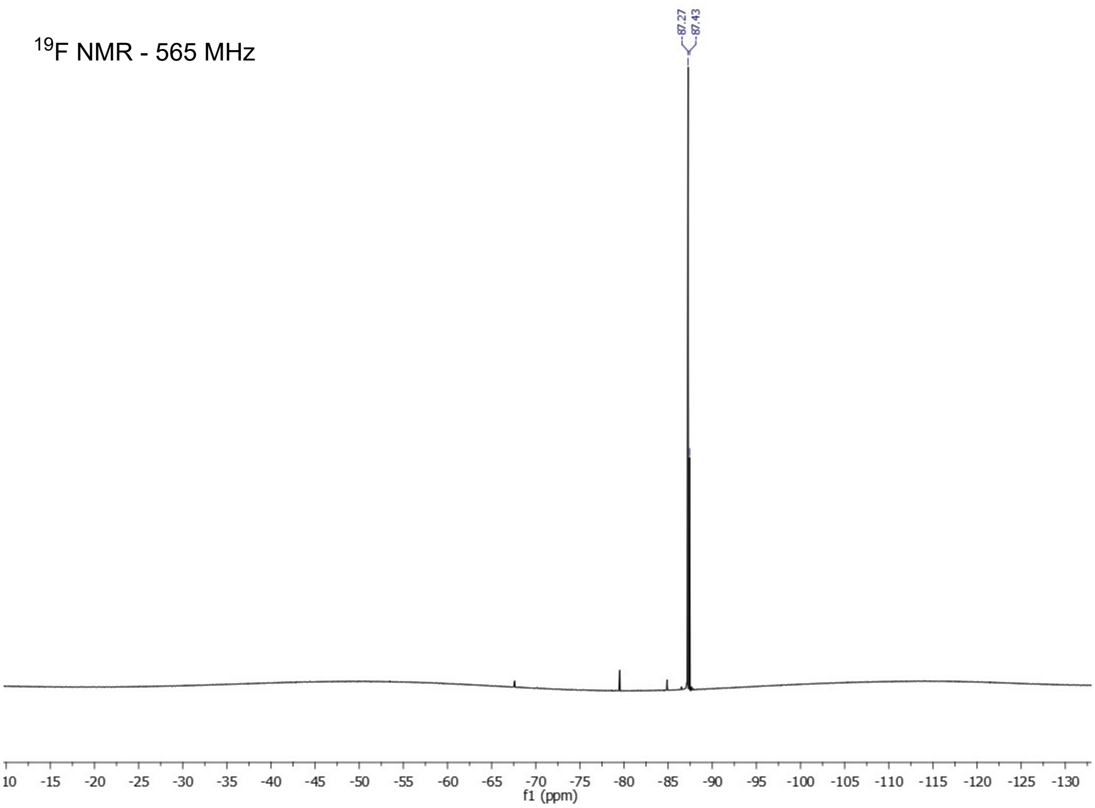

(2*S*,4*S*,5*R*)-4-(4-bromophenyl)-5-methyl-2-(trifluoromethyl)tetrahydro-2H-pyran-2,6-diol (1d)

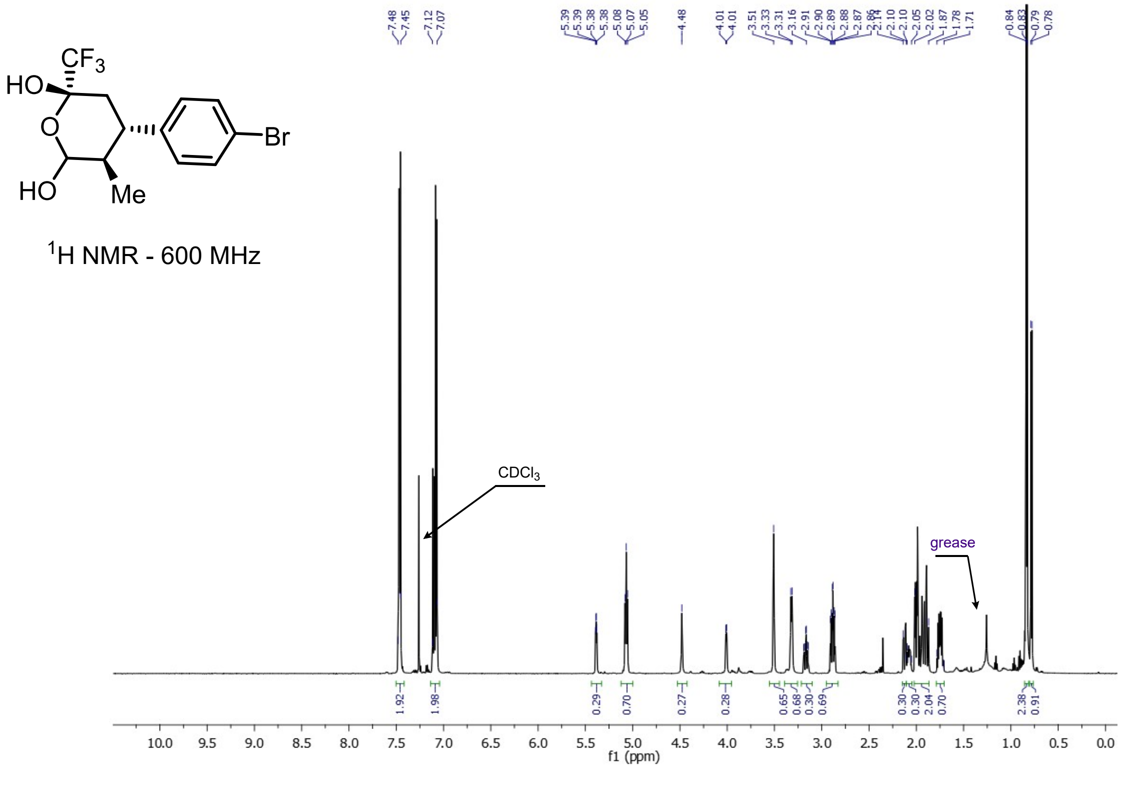

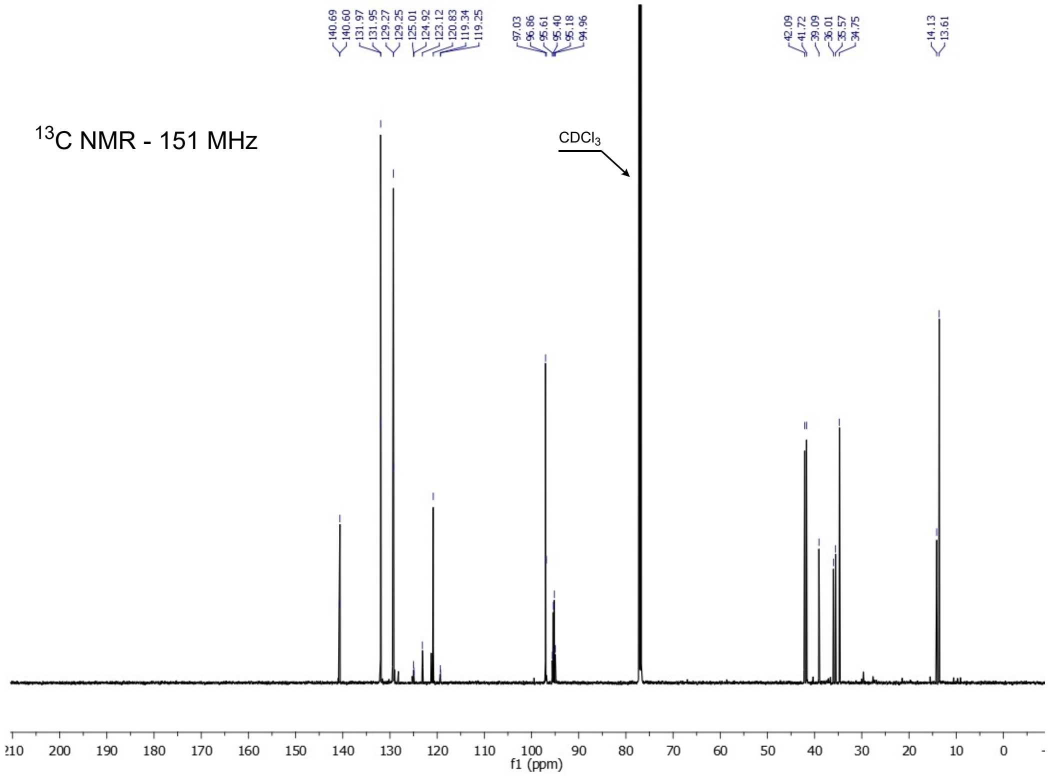

140.69
140.60
131.97
131.95
129.27
129.25
125.01
124.92
123.12
120.83
119.34
119.25
97.03
96.86
95.61
95.40
95.18
94.96
42.09
41.72
39.09
36.01
35.57
34.75
14.13
13.61
^{13}C NMR - 151 MHz
$CDCl_3$
f1 (ppm)

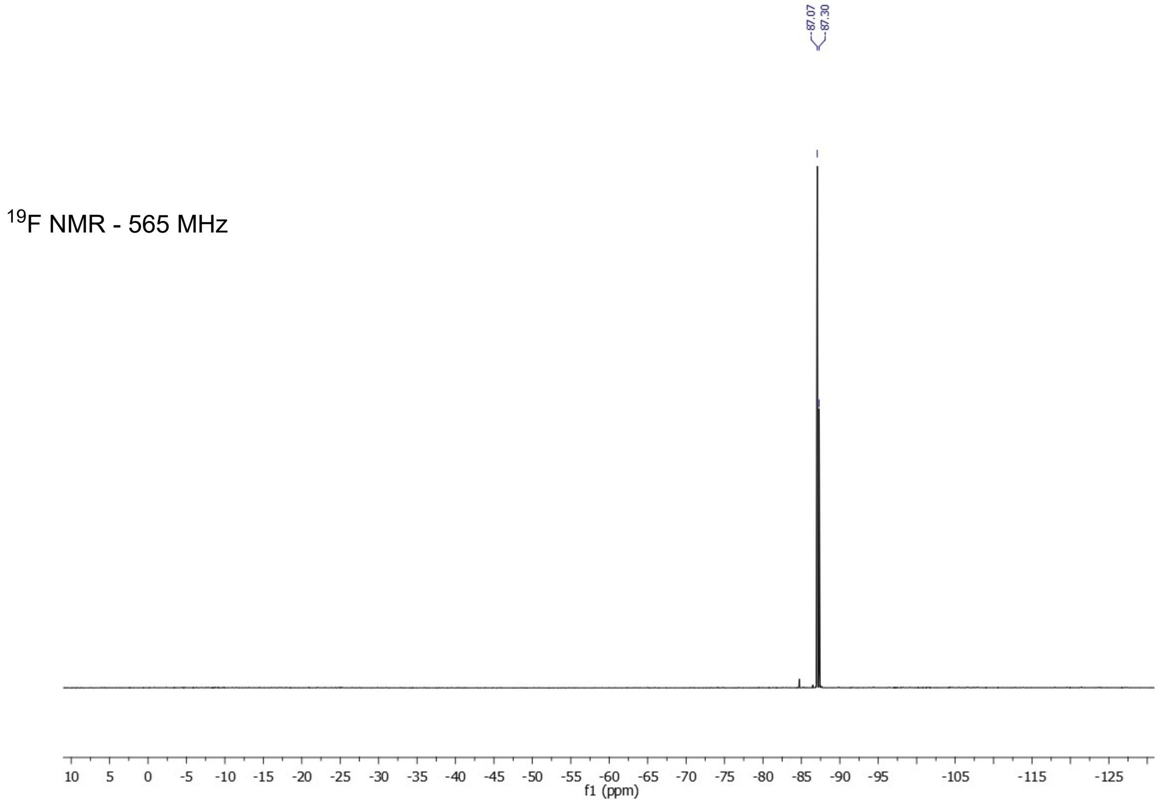

-87.07
-87.30
^{19}F NMR - 565 MHz
f1 (ppm)

(2*S*,4*S*,5*R*)-5-methyl-4-(naphthalen-2-yl)-2-(trifluoromethyl)tetrahydro-2*H*-pyran-2,6-diol (1e)

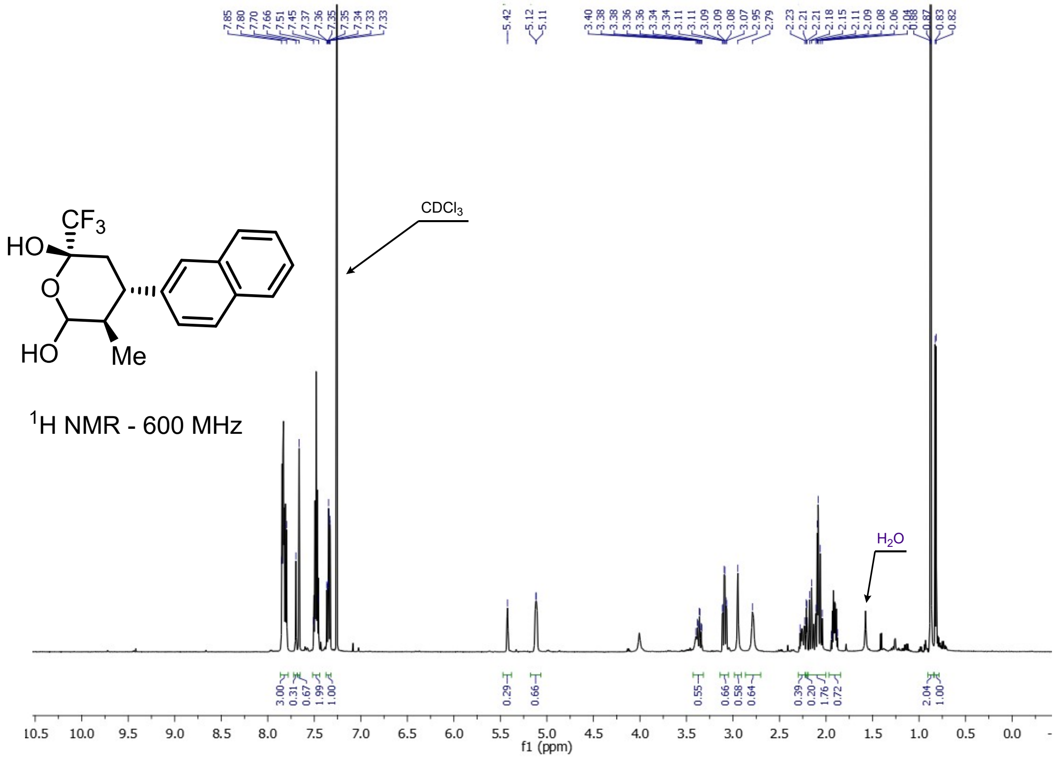

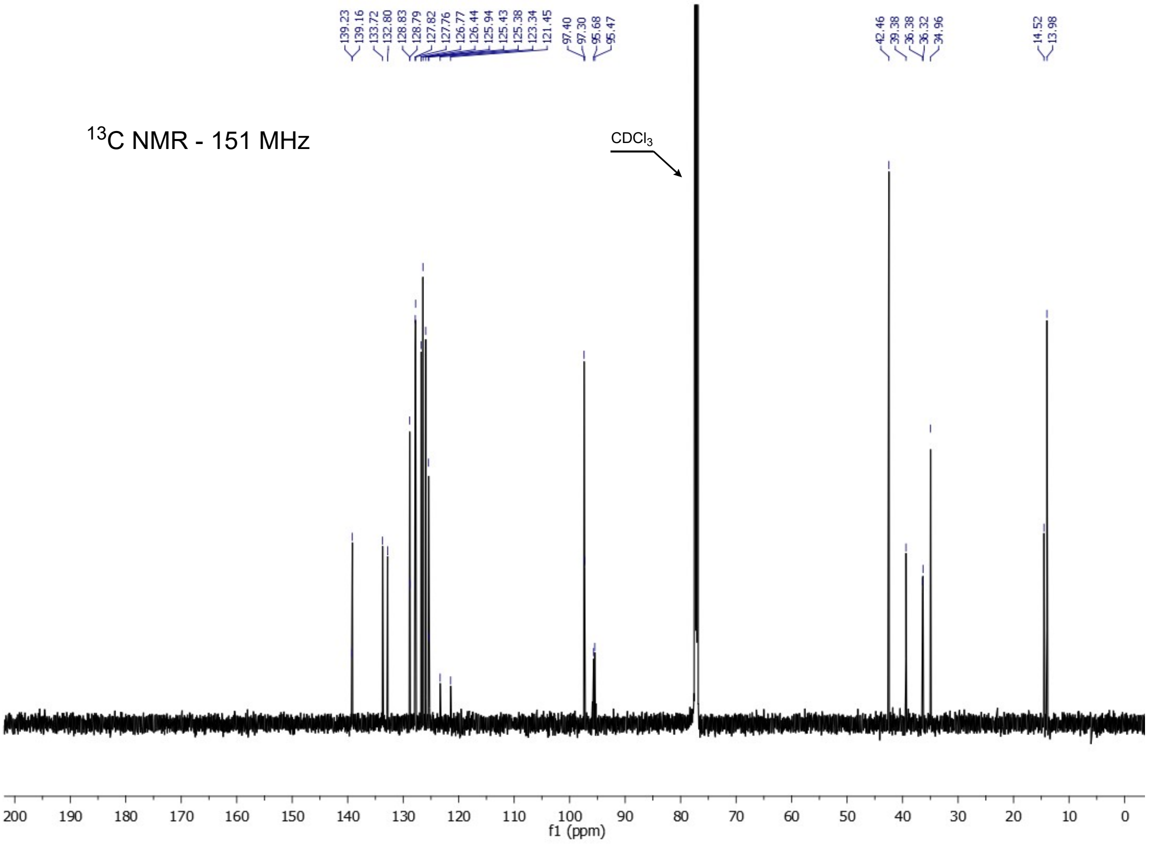

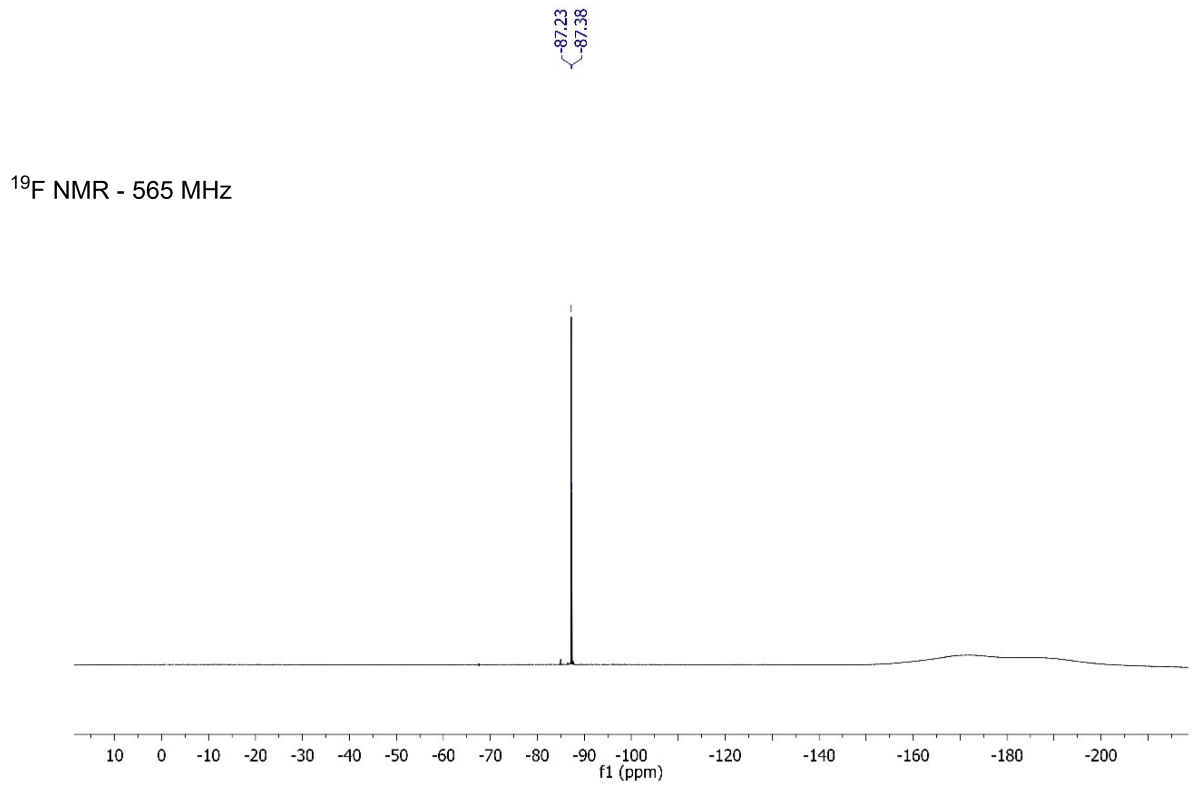

(2*S*,4*S*,5*R*)-4-(2-fluorophenyl)-5-methyl-2-(trifluoromethyl)tetrahydro-2H-pyran-2,6-diol (1f)

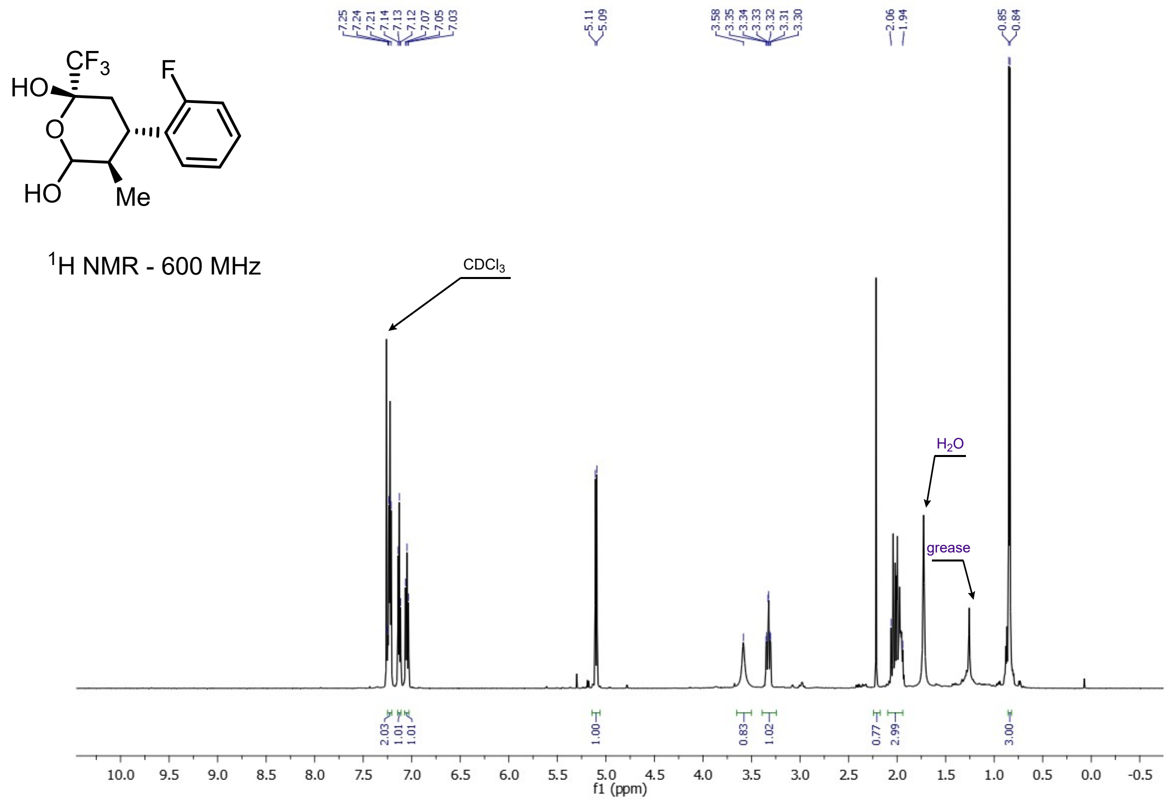

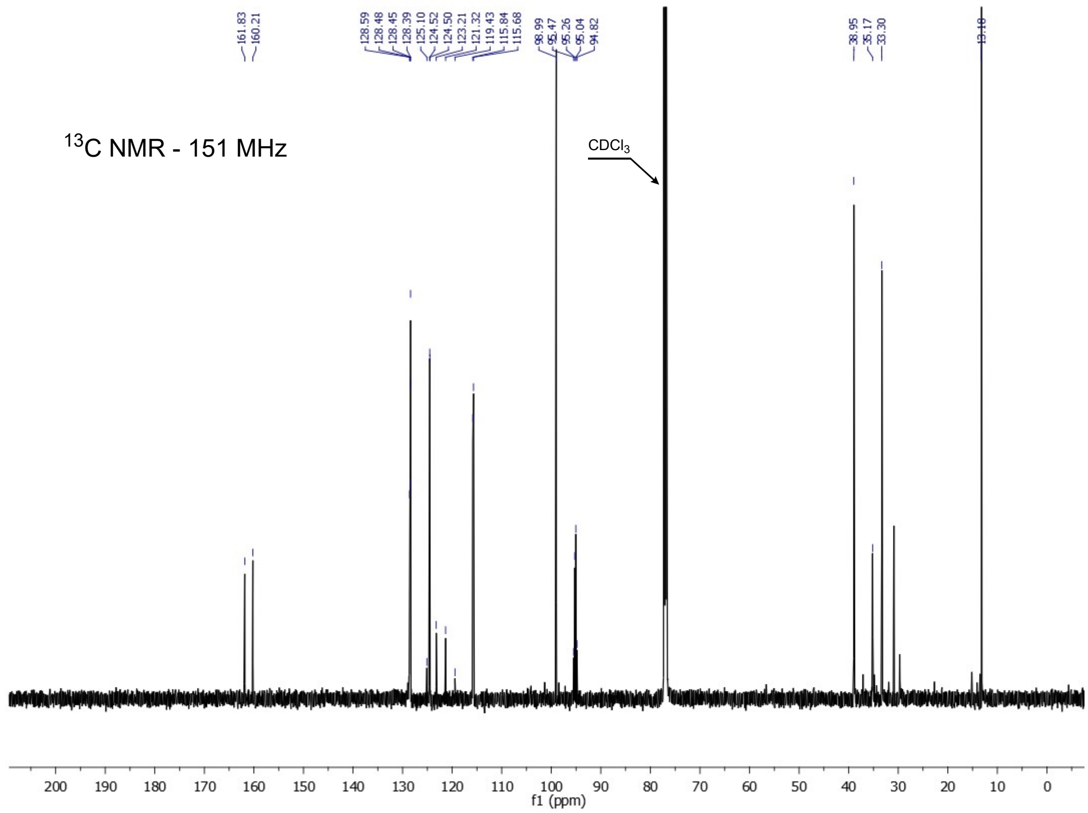

161.83
160.21
128.59
128.48
128.45
128.39
125.10
124.52
124.50
123.21
121.32
119.43
115.84
115.68
98.99
95.47
95.26
95.04
94.82
38.95
35.17
33.30
13.10
^{13}C NMR - 151 MHz
$CDCl_3$
f1 (ppm)

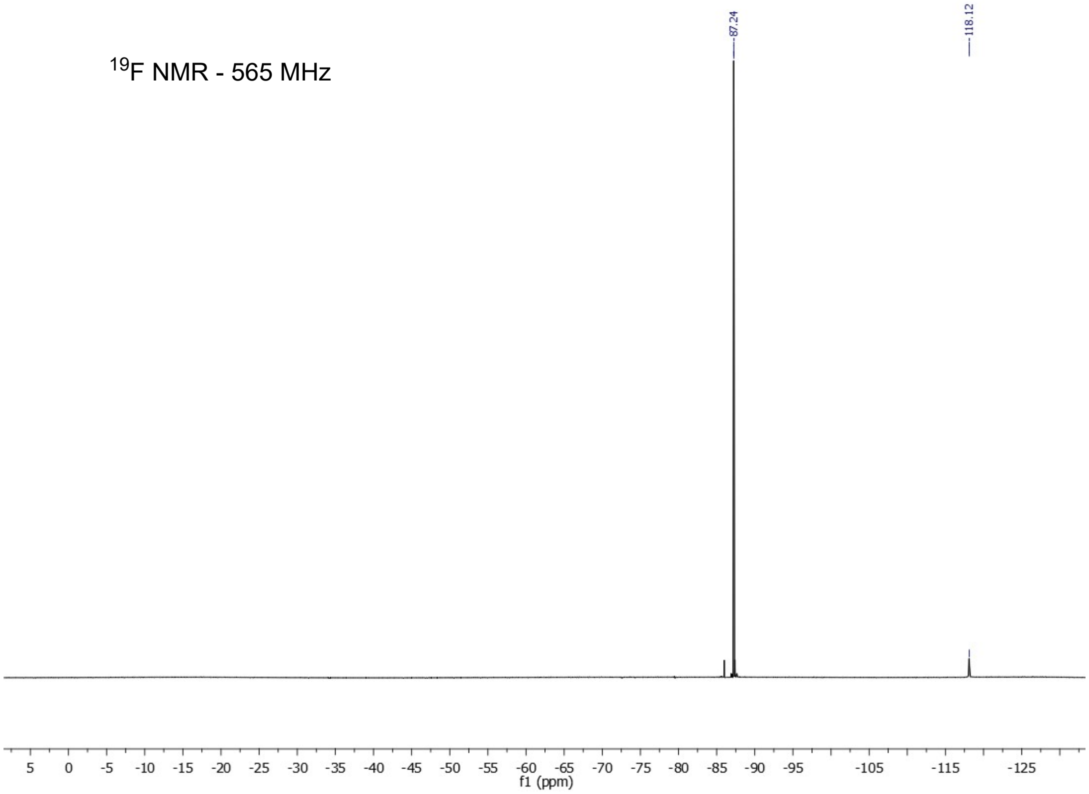

-87.24
-118.12
^{19}F NMR - 565 MHz
f1 (ppm)

(2*S*,4*S*,5*R*)-5-isopropyl-4-phenyl-2-(trifluoromethyl)tetrahydro-2H-pyran-2,6-diol (1g)

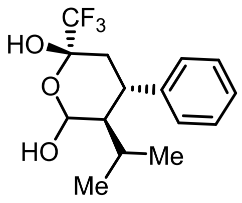

^{1}H NMR - 600 MHz

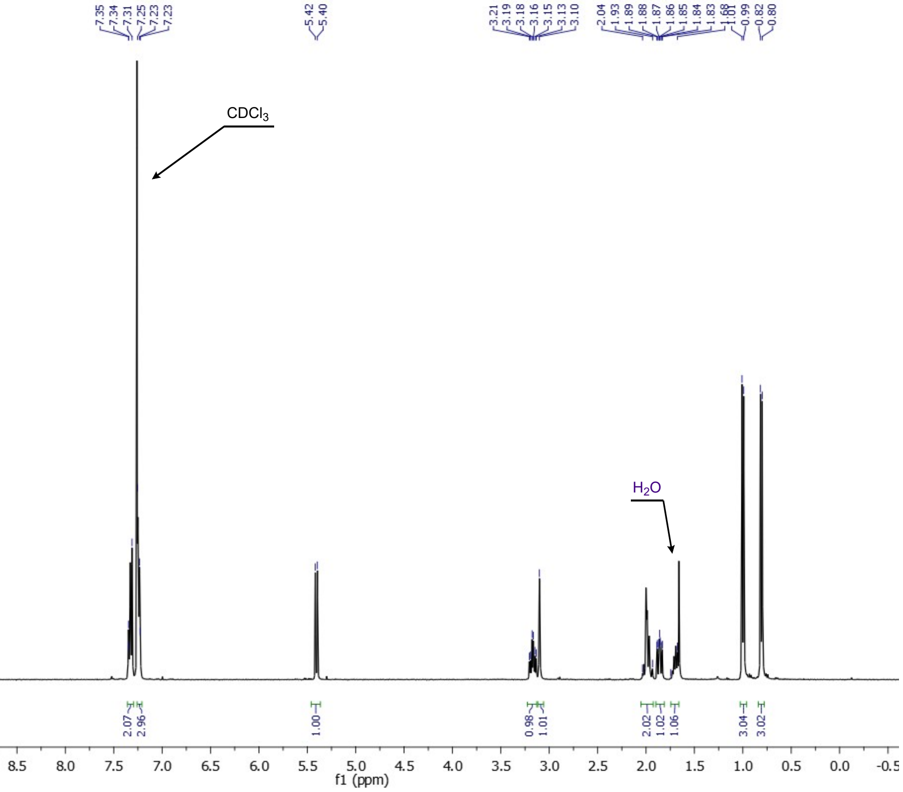

^{13}C NMR - 151 MHz

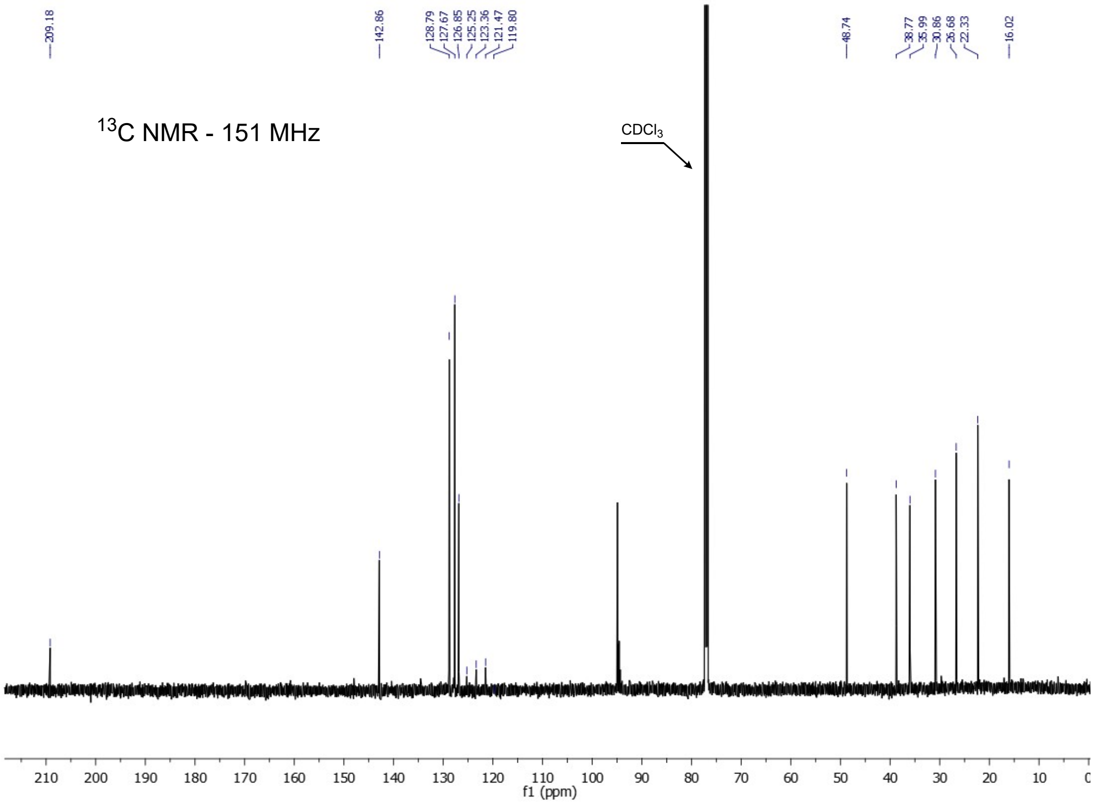

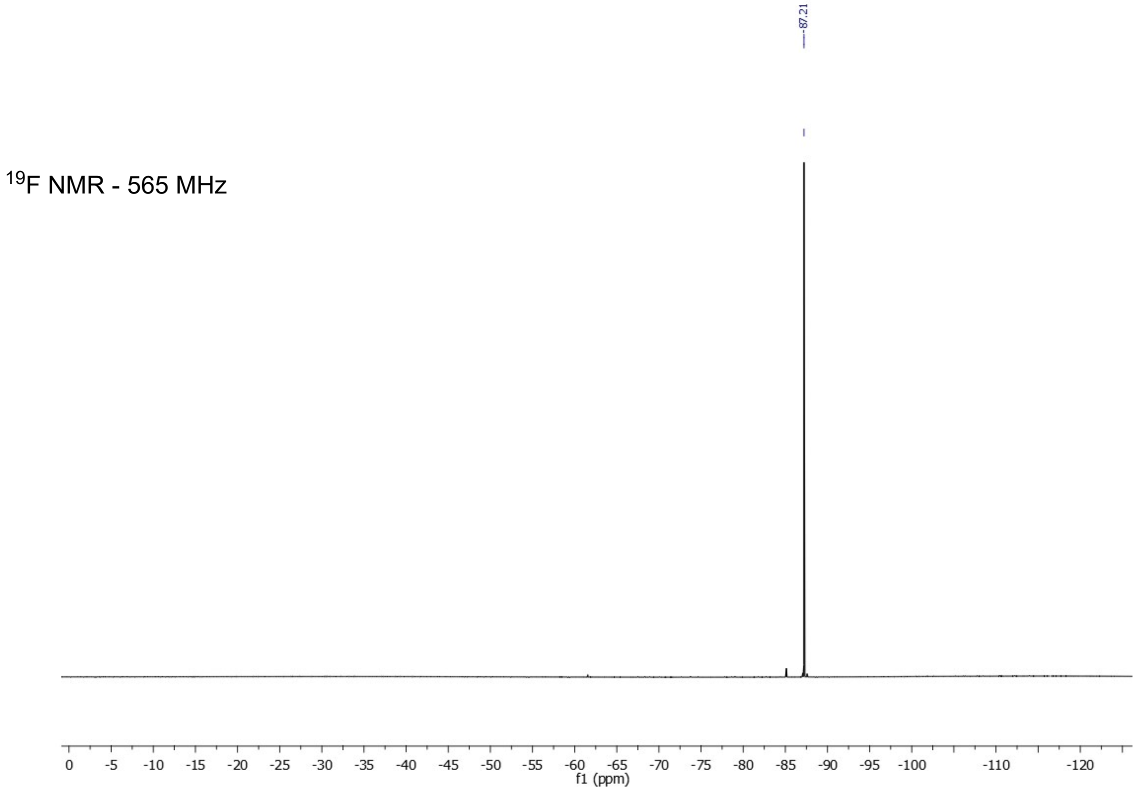

(2*S*,4*S*,5*R*)-5-methyl-4-phenyl-2-(trifluoromethyl)tetrahydro-2H-pyran-6-d-2,6-diol (1h)

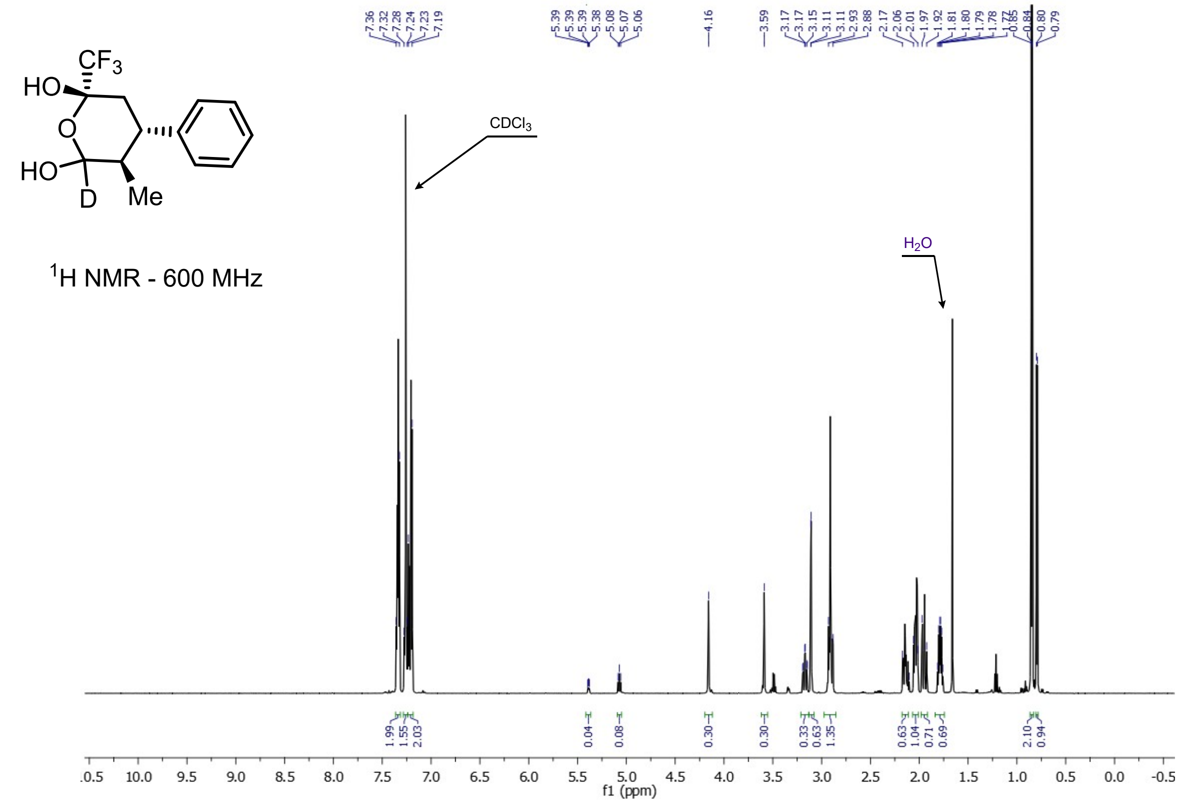

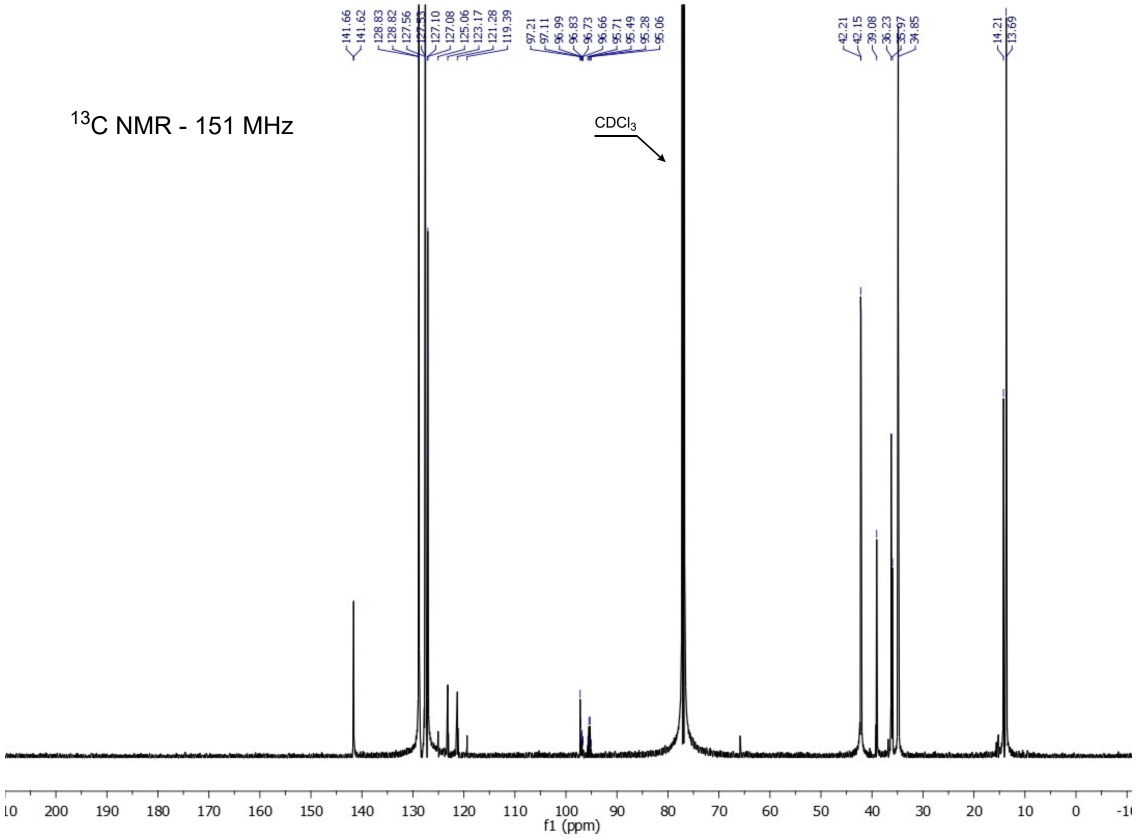

13C NMR - 151 MHz
CDCl3
141.66
141.62
128.83
128.82
127.56
127.53
127.10
127.08
125.06
123.17
121.28
119.39
97.21
97.11
96.99
96.83
96.73
96.66
95.71
95.49
95.28
95.06
42.21
42.15
39.08
36.23
35.97
34.85
14.21
13.69
f1 (ppm)

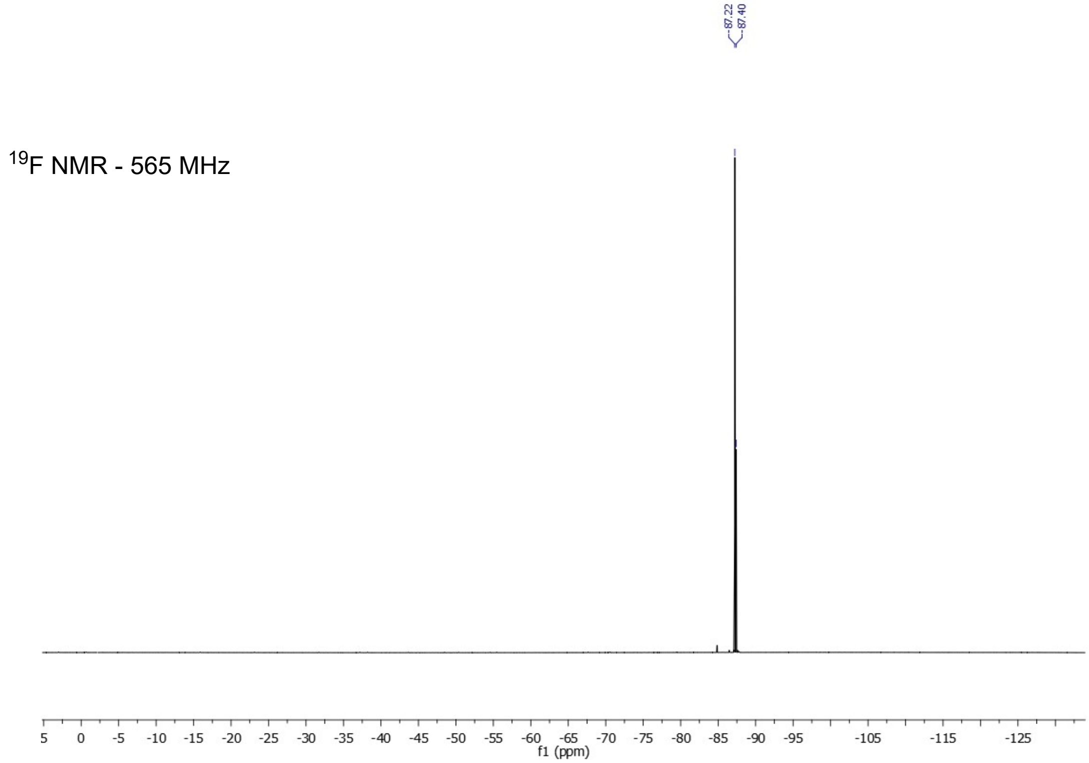

19F NMR - 565 MHz
-87.22
-87.40
f1 (ppm)

9.2 Hydride Transfer Products

(2*R*,3*S*,5*R*)-6,6,6-trifluoro-5-hydroxy-2-methyl-3-phenyl-1-(pyrrolidin-1-yl)hexan-1-one (3a)

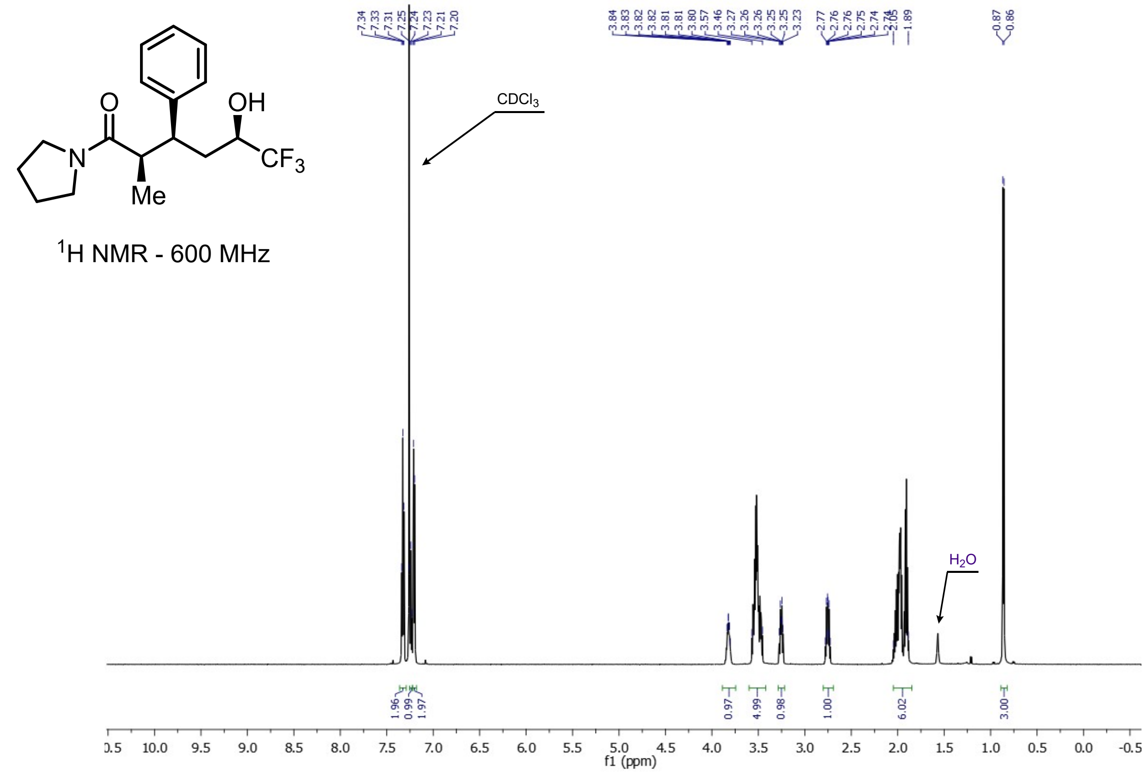

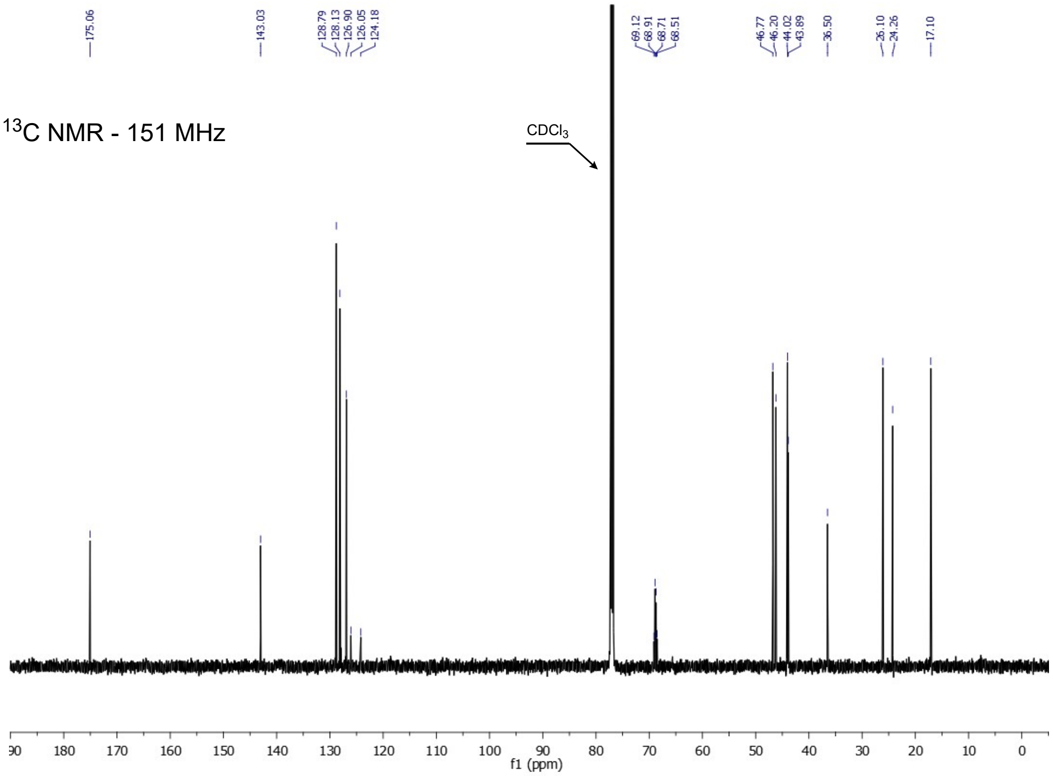

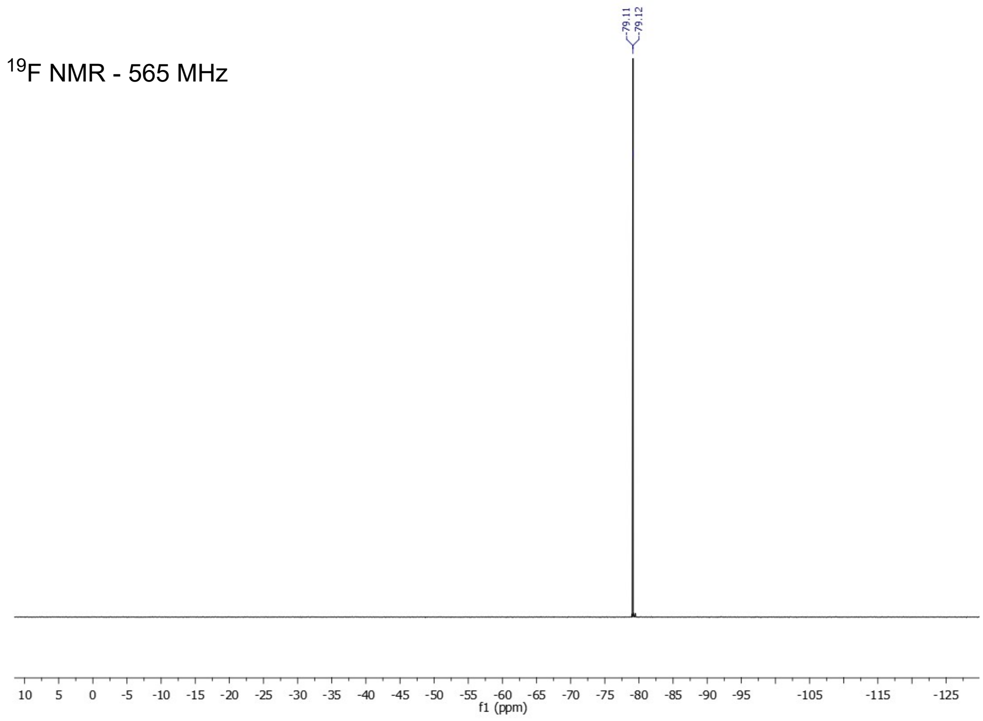

(2*R*,3*S*,5*R*)-6,6,6-trifluoro-5-hydroxy-2-methyl-3-phenyl-1-(piperidin-1-yl)hexan-1-one (3b)

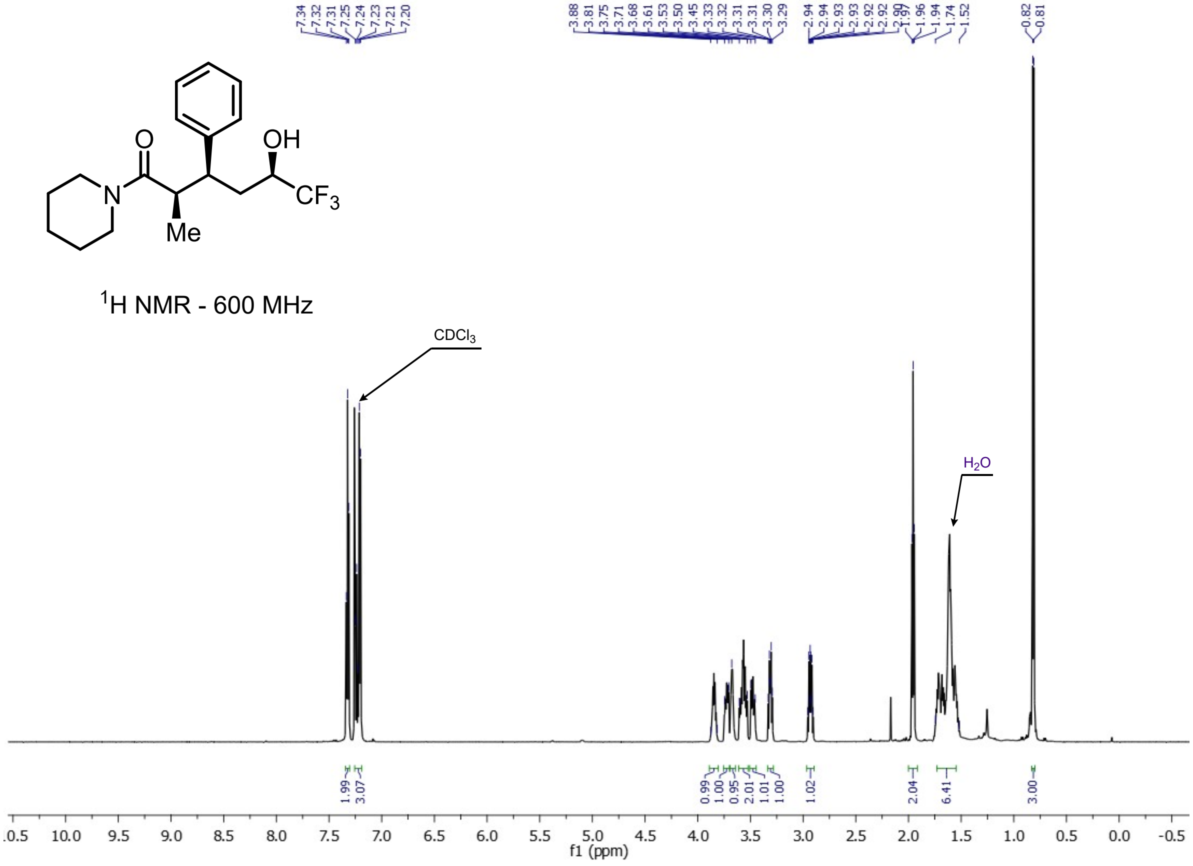

13C NMR - 151 MHz

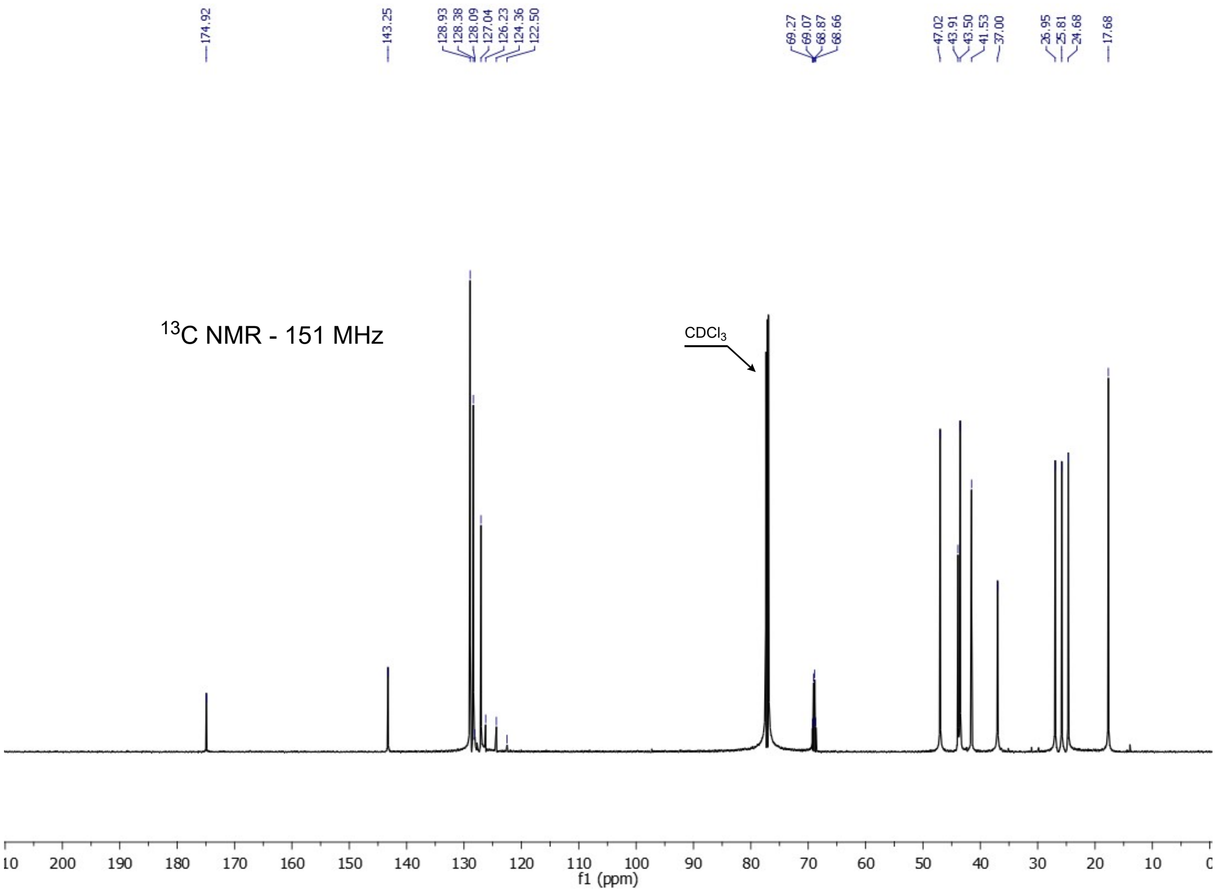

19F NMR - 565 MHz

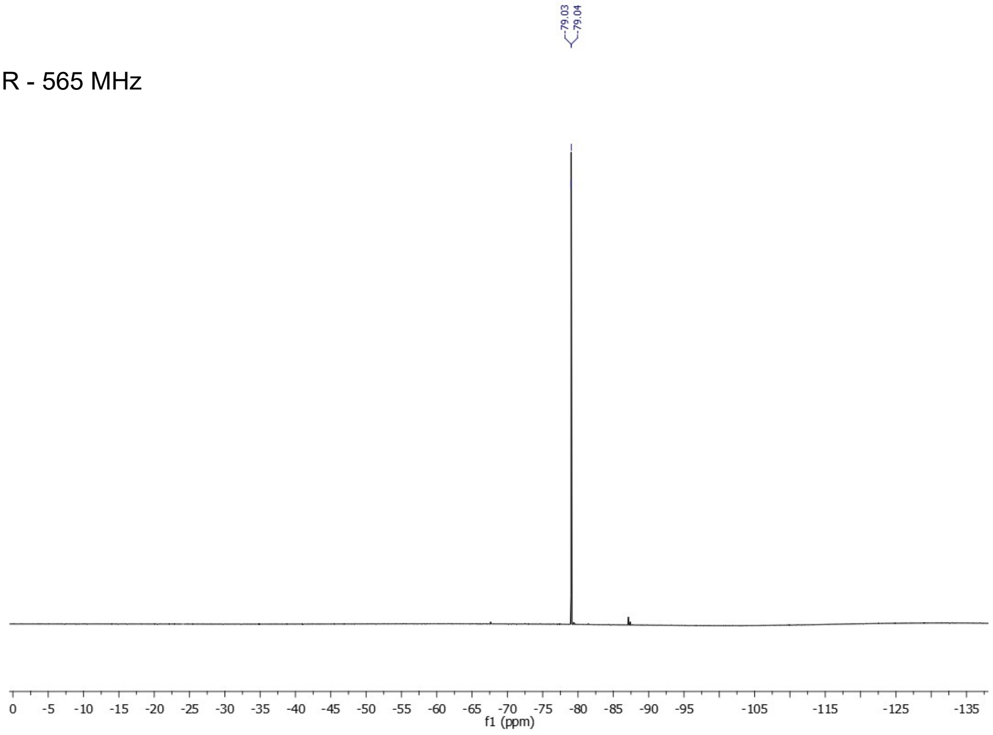

(2*R*,3*S*,5*R*)-1-(azepan-1-yl)-6,6,6-trifluoro-5-hydroxy-2-methyl-3-phenylhexan-1-one (3c)

^{1}H NMR - 700 MHz

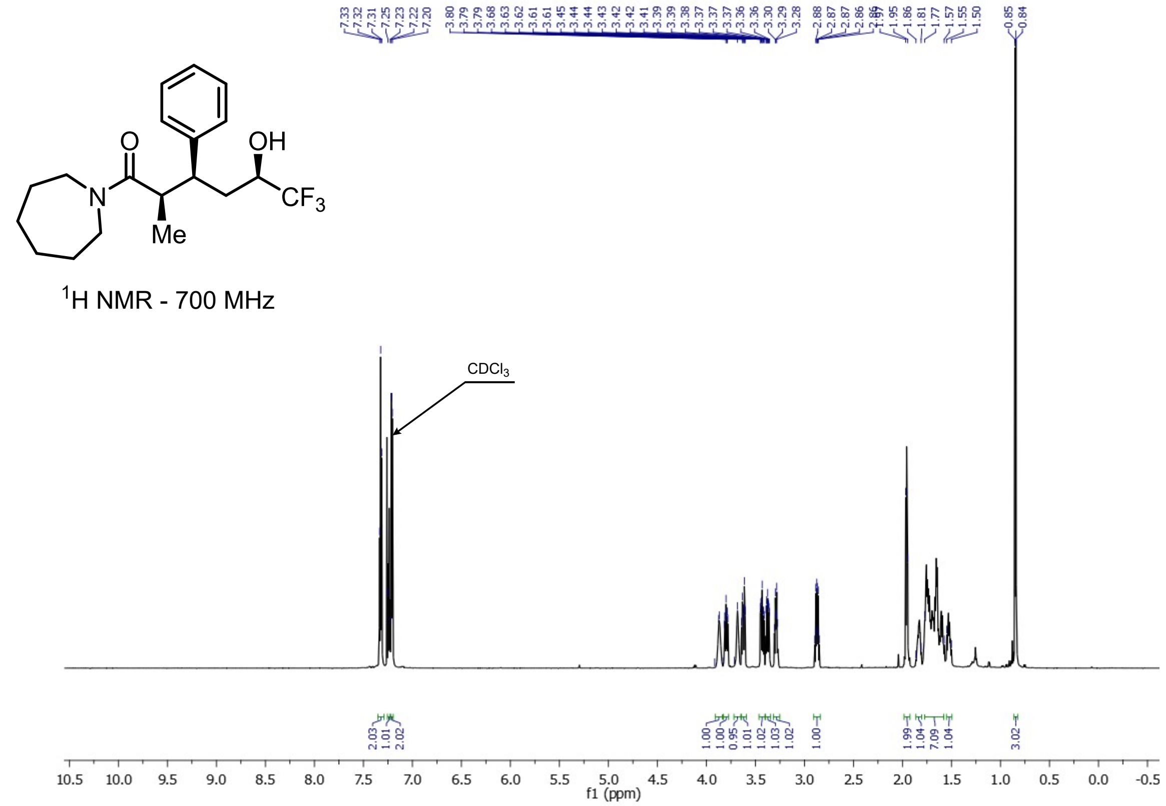

^{13}C NMR - 176 MHz

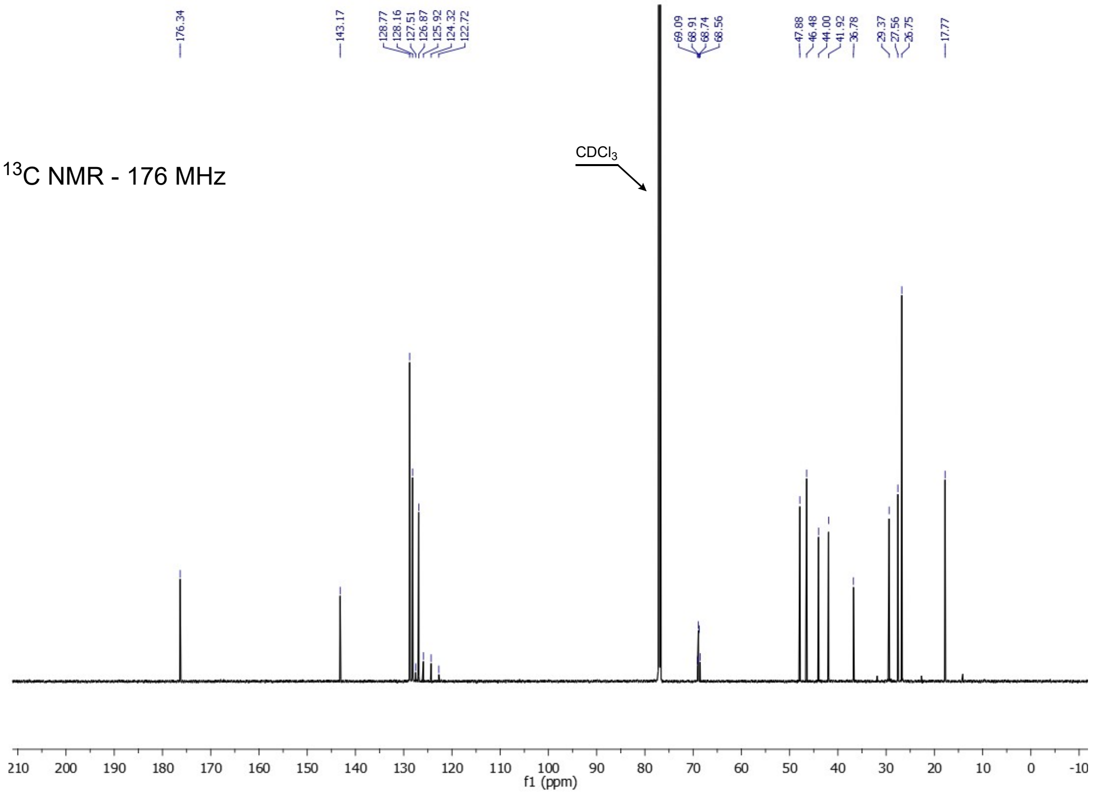

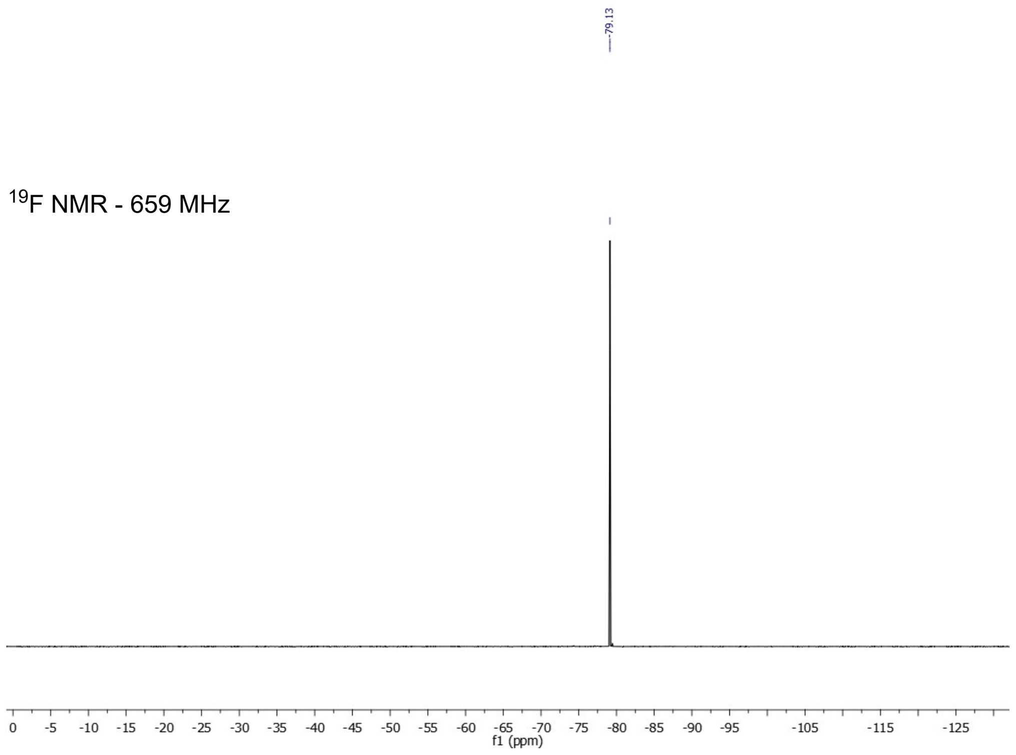

(2R,3S,5R)-6,6,6-trifluoro-5-hydroxy-N,2-dimethyl-N-phenethyl-3-phenylhexanamide (3d)

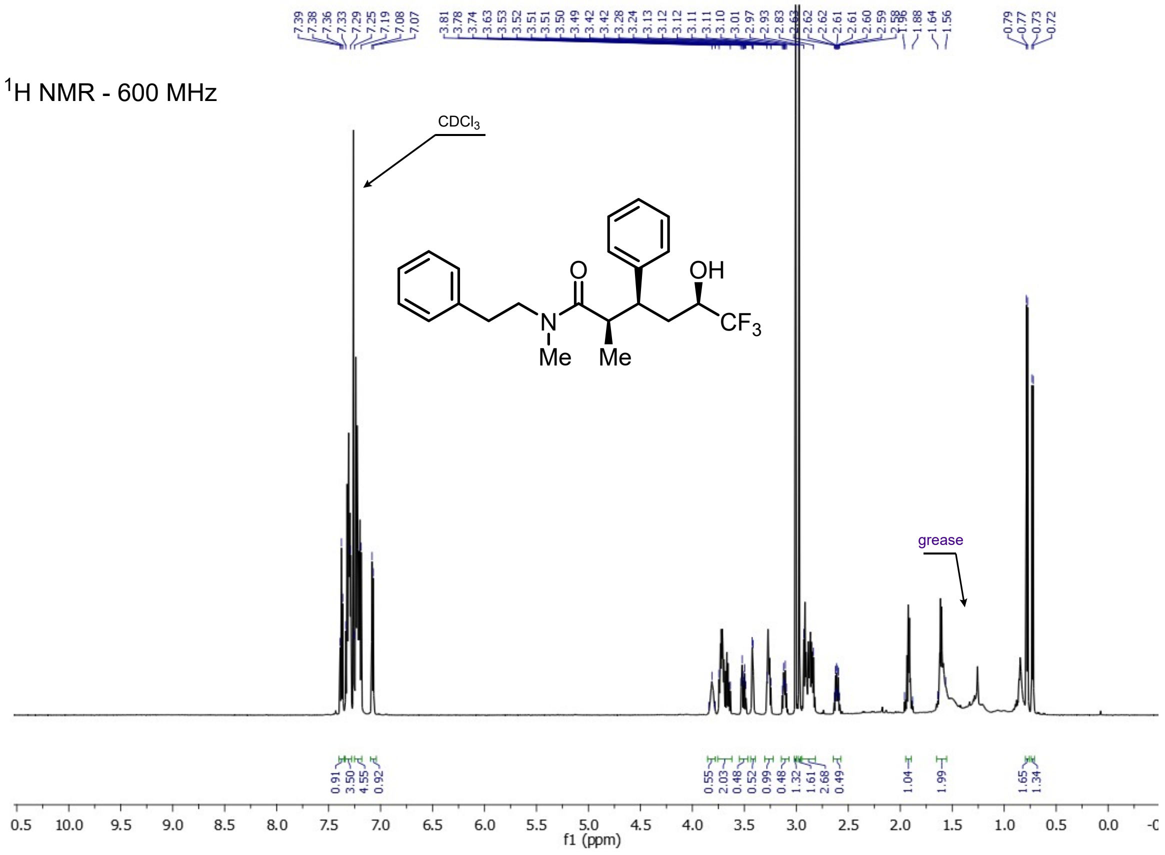

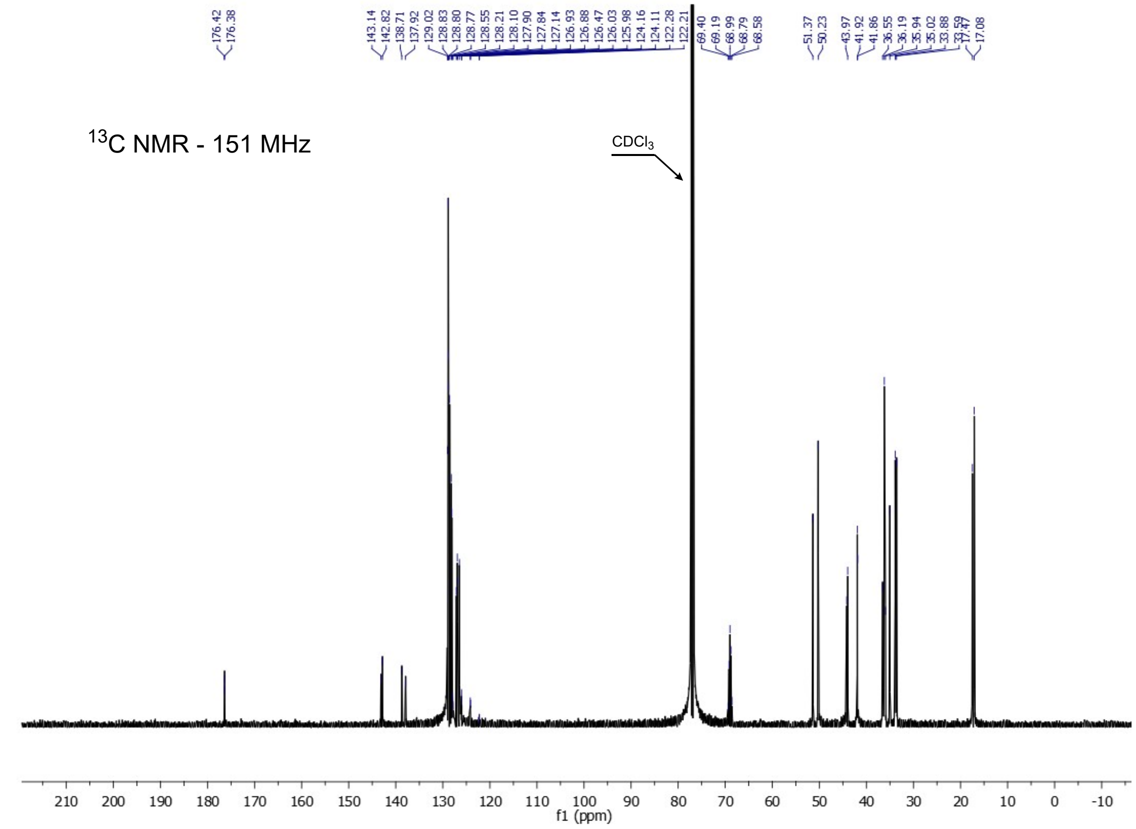
13C NMR - 151 MHz
CDCl3
176.42
176.38
143.14
142.82
138.71
137.92
129.02
128.83
128.80
128.77
128.55
128.21
128.10
127.90
127.84
127.14
126.93
126.88
126.47
126.03
125.98
124.16
124.11
122.28
122.21
69.40
69.19
68.99
68.79
68.58
51.37
50.23
43.97
41.92
41.86
36.55
36.19
35.94
35.02
33.88
33.59
17.47
17.08
210 200 190 180 170 160 150 140 130 120 110 100 90 80 70 60 50 40 30 20 10 0 -10
f1 (ppm)

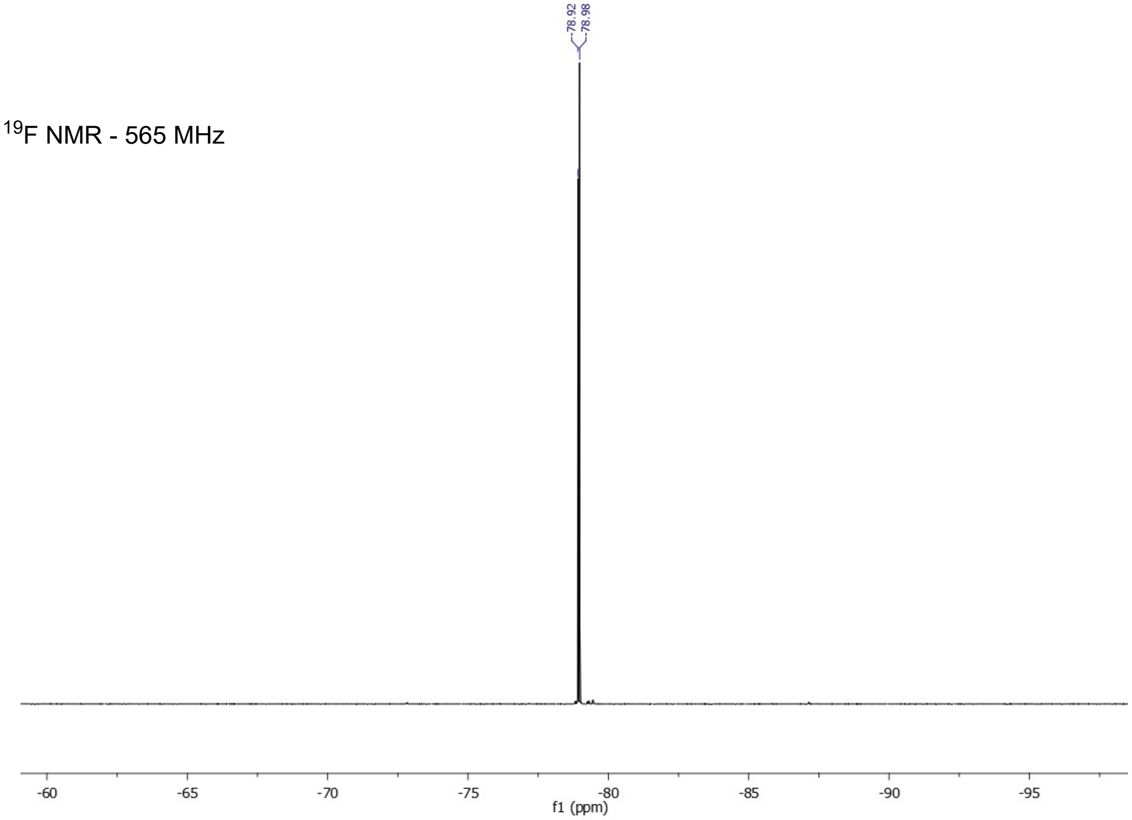
19F NMR - 565 MHz
-78.92
-78.98
-60 -65 -70 -75 -80 -85 -90 -95
f1 (ppm)

(2*R*,3*S*,5*R*)-N,N-diethyl-6,6,6-trifluoro-5-hydroxy-2-methyl-3-phenylhexanamide (3e)

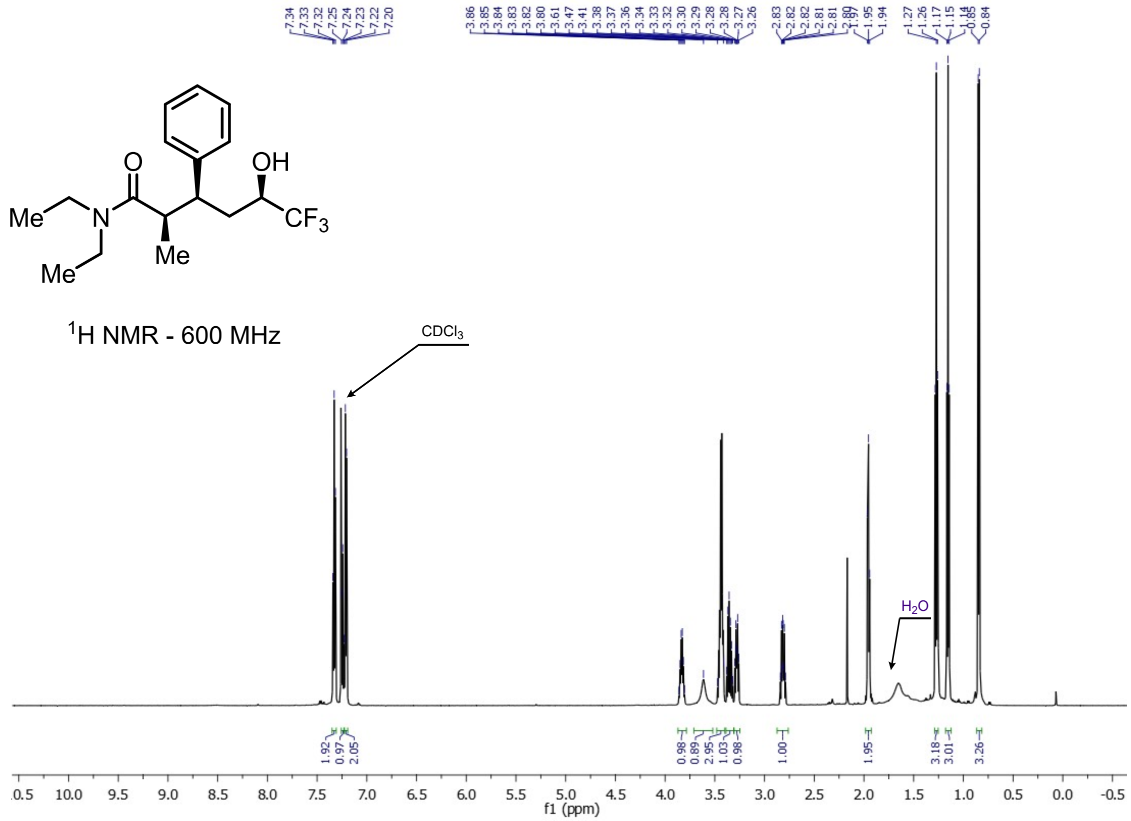

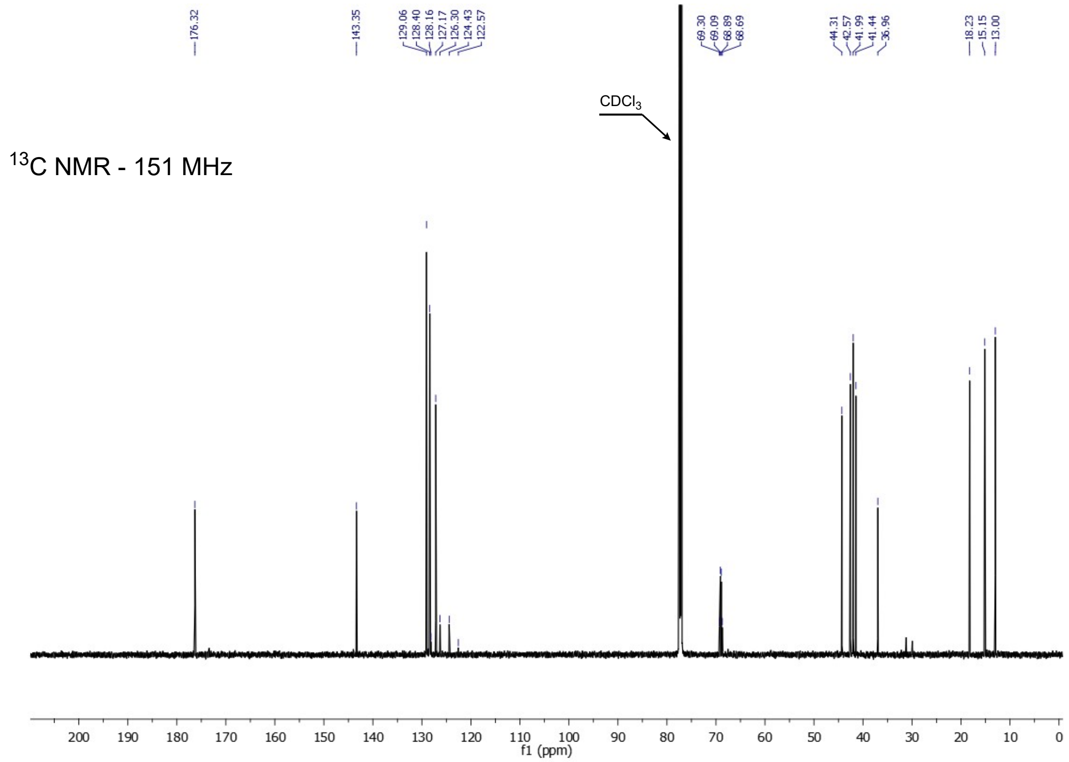

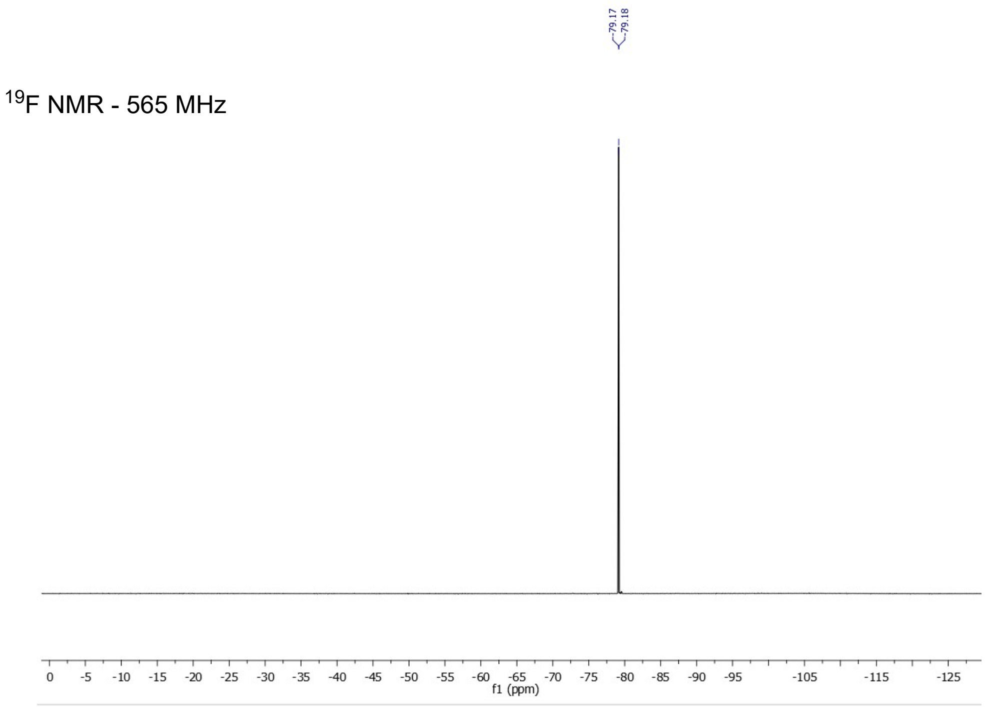

(2*R*,3*S*,5*R*)-*N*,*N*-diallyl-6,6,6-trifluoro-5-hydroxy-2-methyl-3-phenylhexanamide (3f)

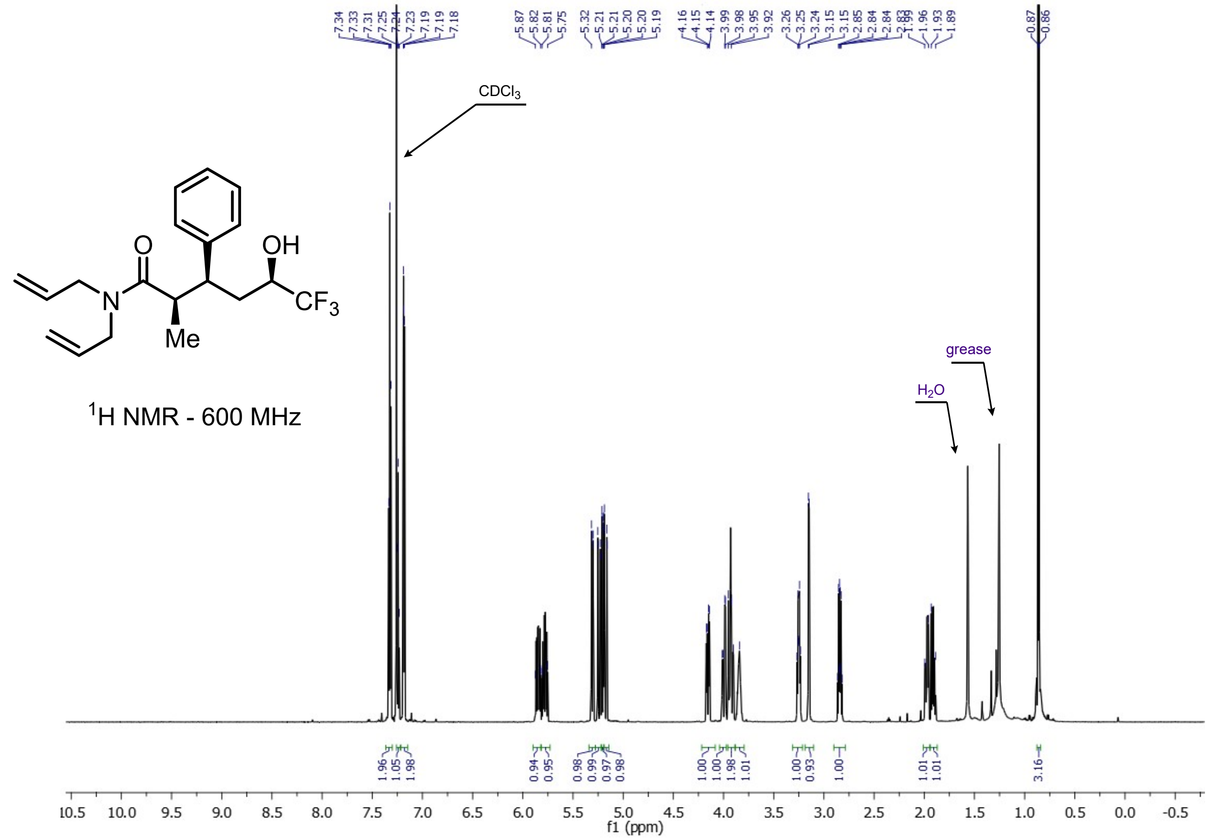

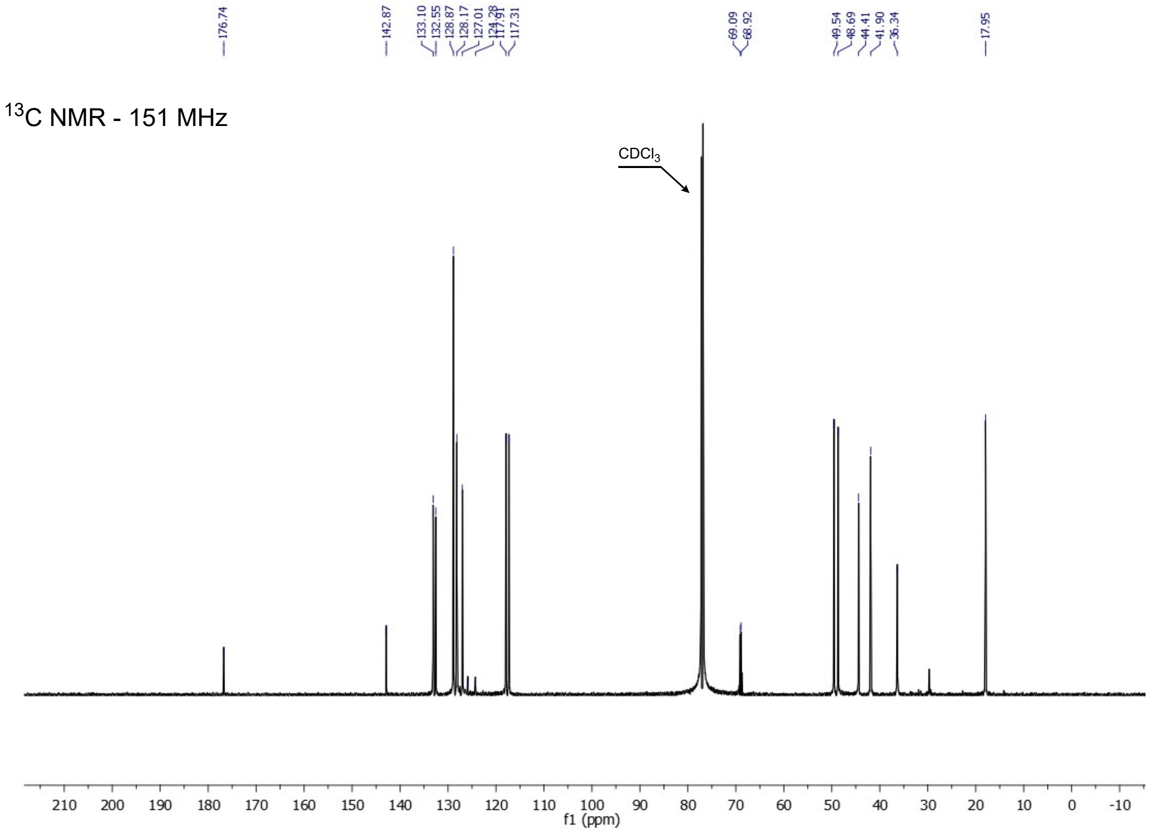
176.74
142.87
133.10
132.55
128.87
128.17
127.01
117.91
117.31
69.09
68.92
49.54
48.69
44.41
41.90
36.34
17.95
13C NMR - 151 MHz
CDCl3
f1 (ppm)

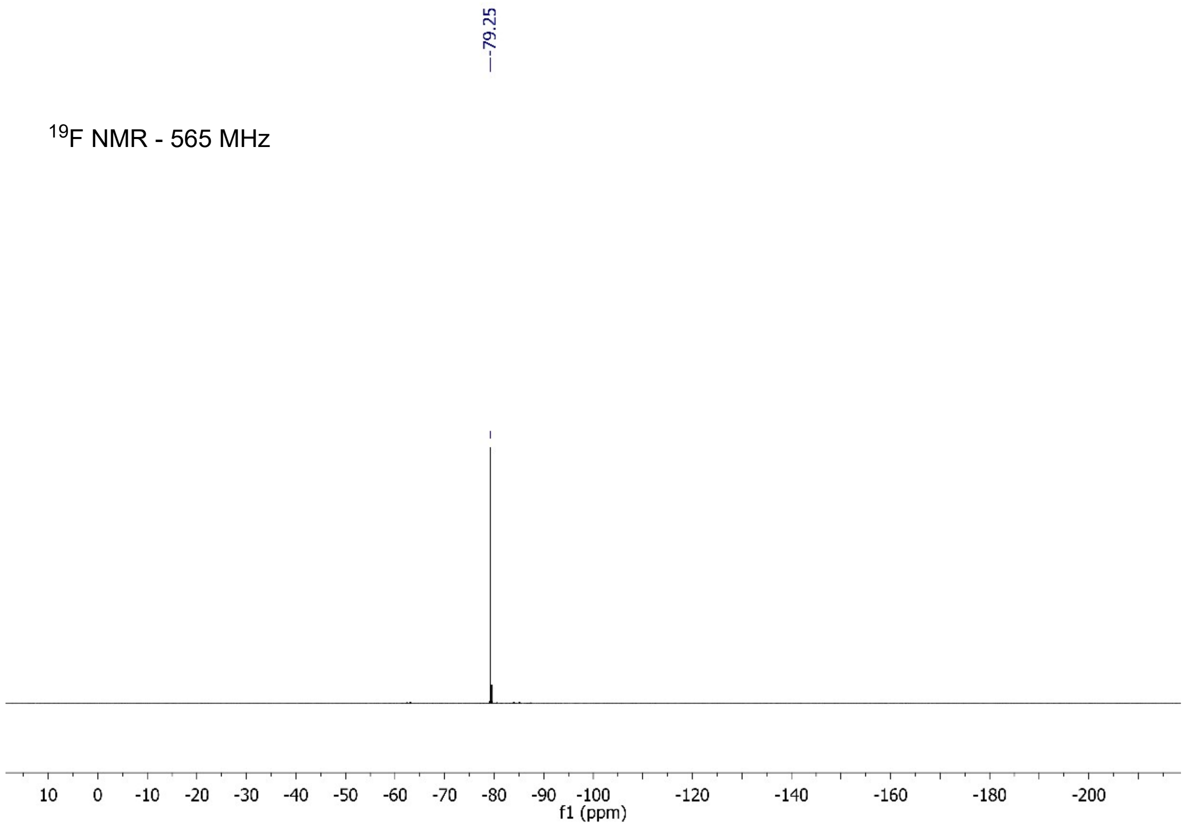
-79.25
19F NMR - 565 MHz
f1 (ppm)

benzyl 4-((2*R*,3*S*,5*R*)-6,6,6-trifluoro-5-hydroxy-2-methyl-3-phenylhexanoyl)piperazine-1-carboxylate (3g)

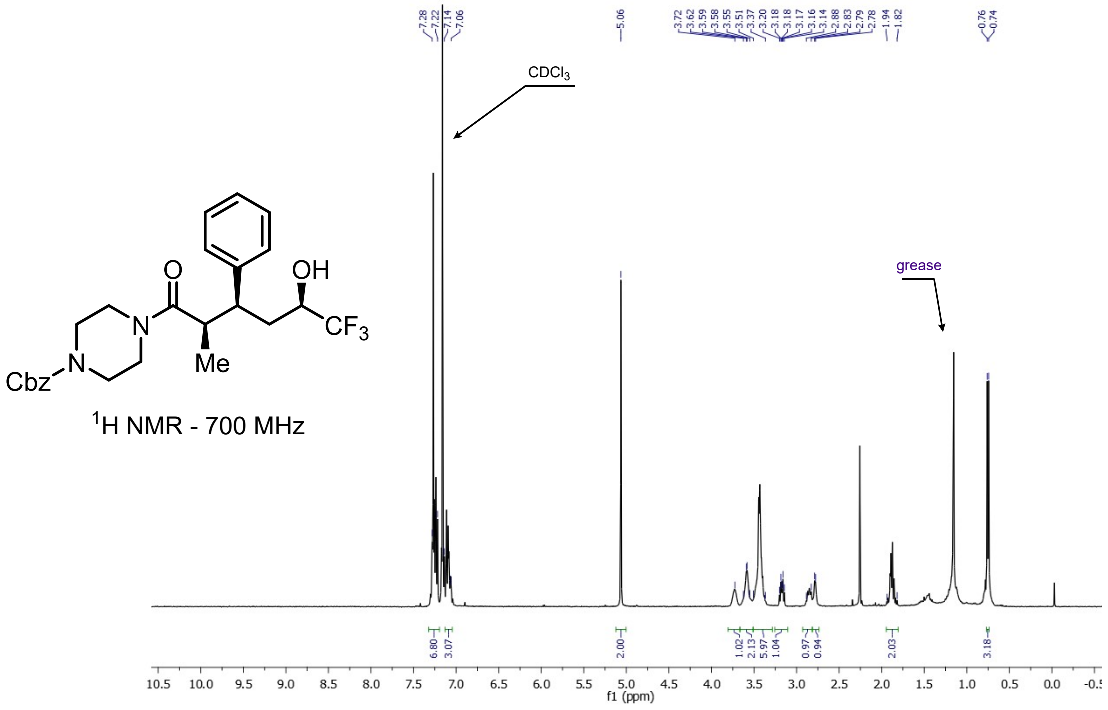

^{13}C NMR - 176 MHz

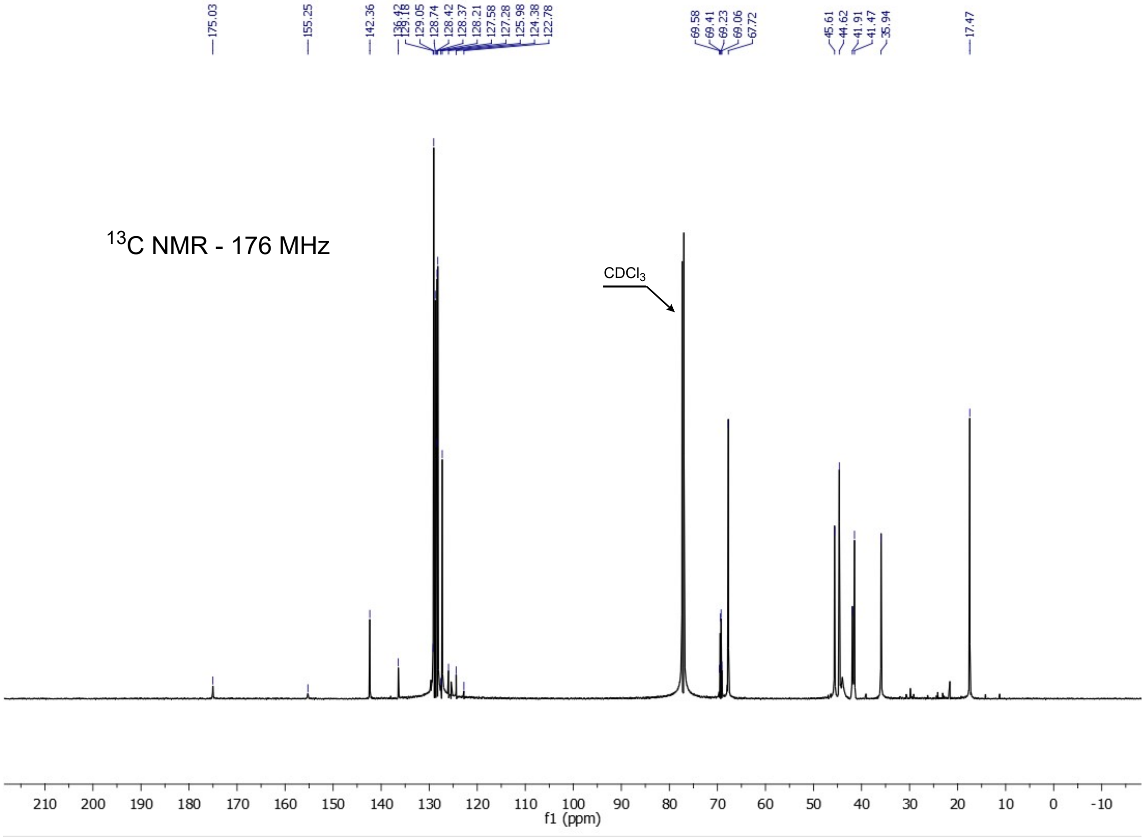

^{19}F NMR - 659 MHz

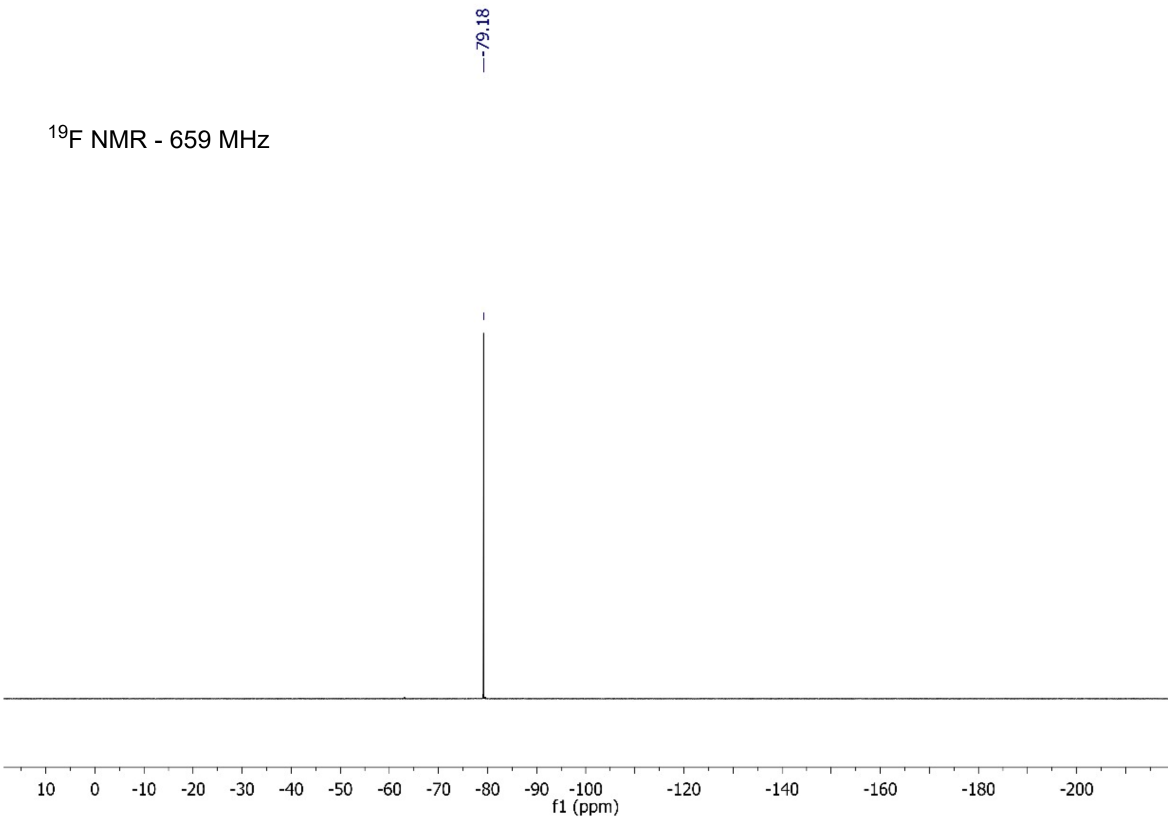

(2*R*,3*S*,5*R*)-6,6,6-trifluoro-5-hydroxy-2-methyl-1-morpholino-3-phenylhexan-1-one (3h)

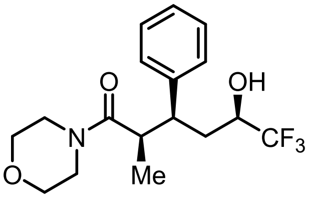

^{1}H NMR - 700 MHz

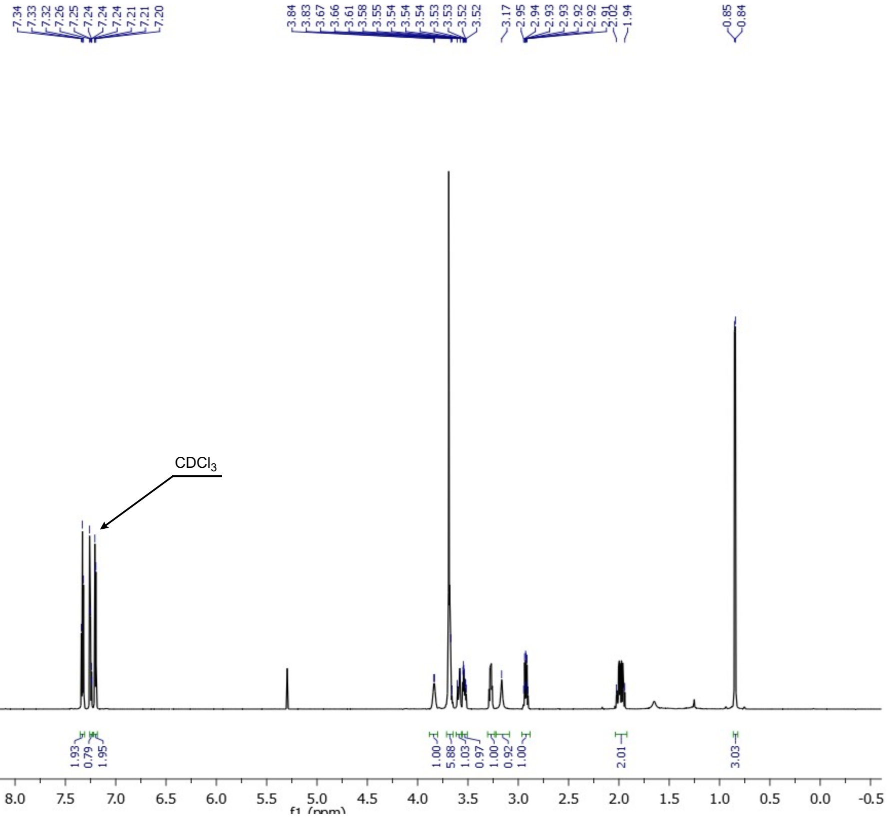

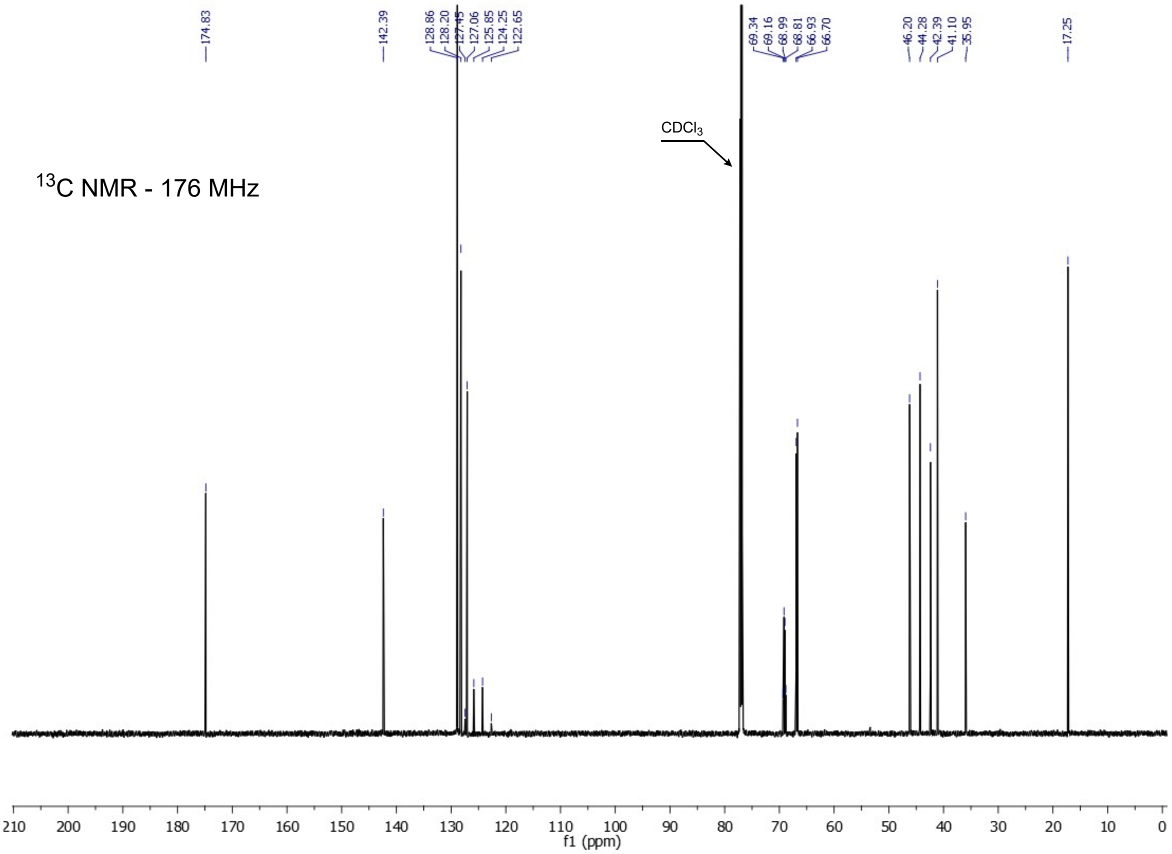
174.83
142.39
128.86
128.20
127.45
127.06
125.85
124.25
122.65
69.34
69.16
68.99
68.81
66.93
66.70
46.20
44.28
42.39
41.10
35.95
17.25
CDCl3
13C NMR - 176 MHz
f1 (ppm)

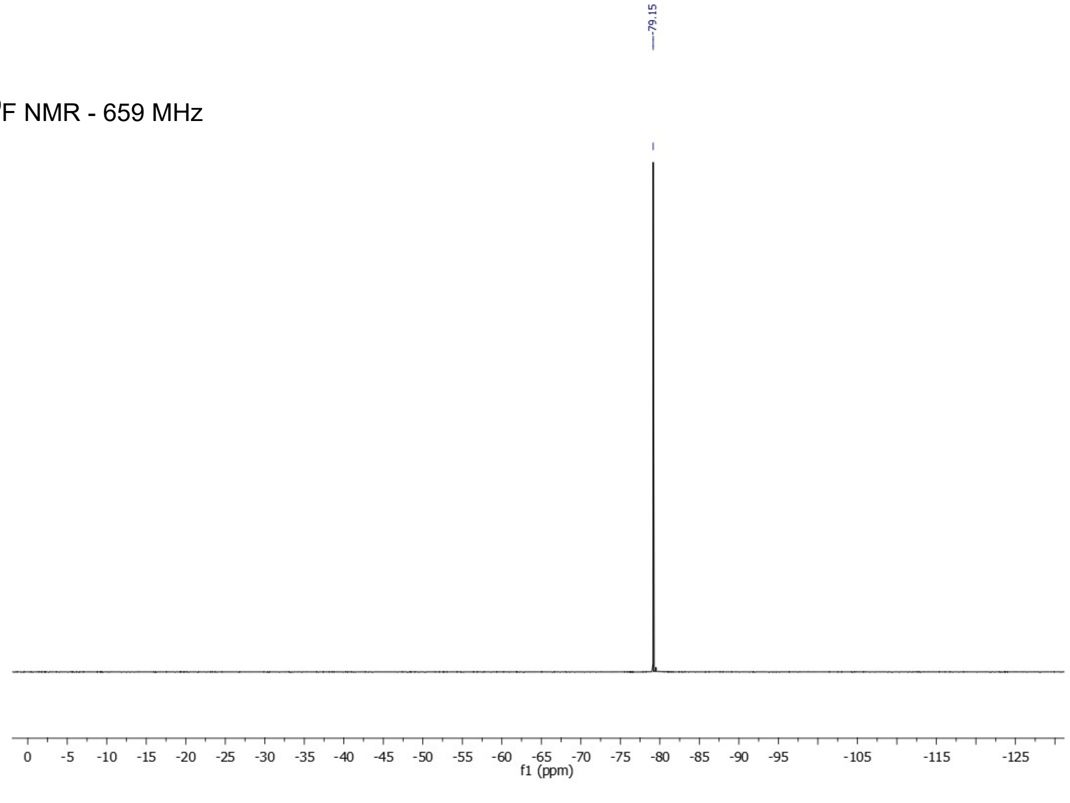
-79.15
19F NMR - 659 MHz
f1 (ppm)

(2*R*,3*S*,5*R*)-6,6,6-trifluoro-5-hydroxy-2-methyl-1-(pyrrolidin-1-yl)-3-(m-tolyl)hexan-1-one (3i)

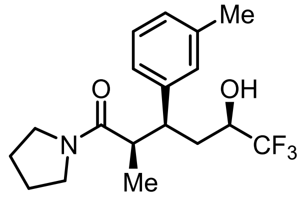

^{1}H NMR - 700 MHz

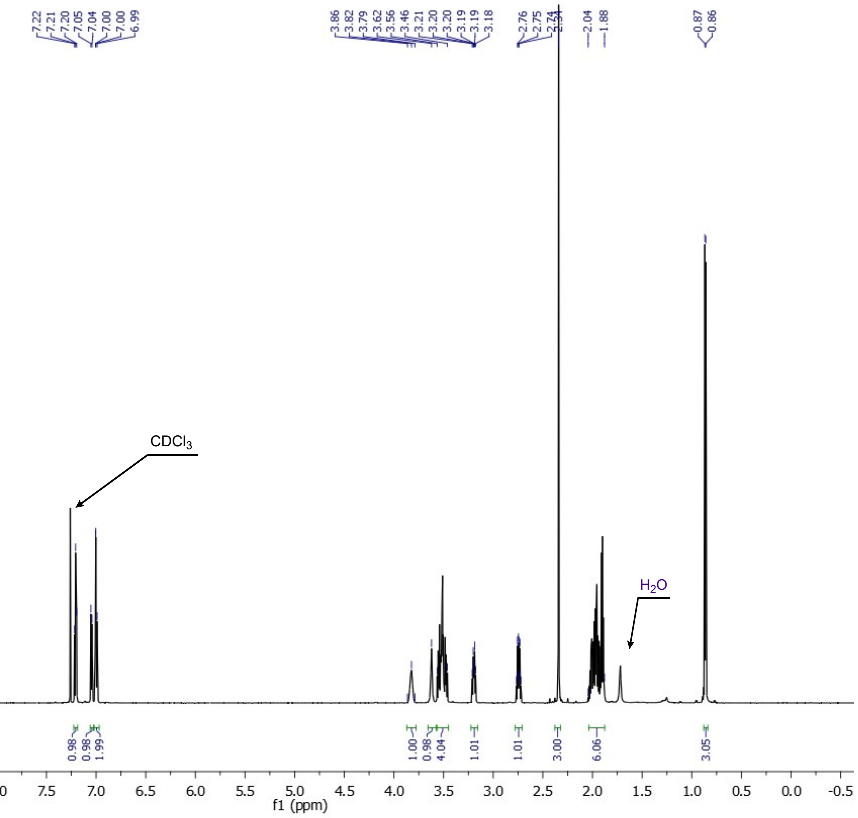

^{13}C NMR - 176 MHz

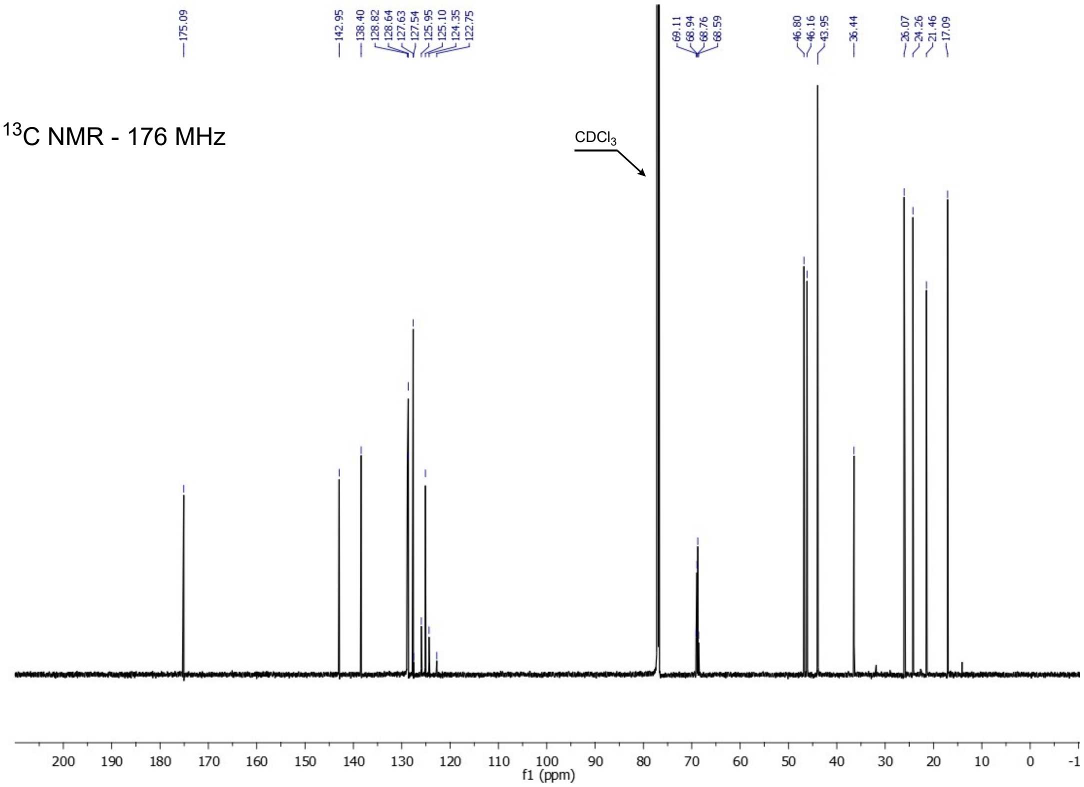

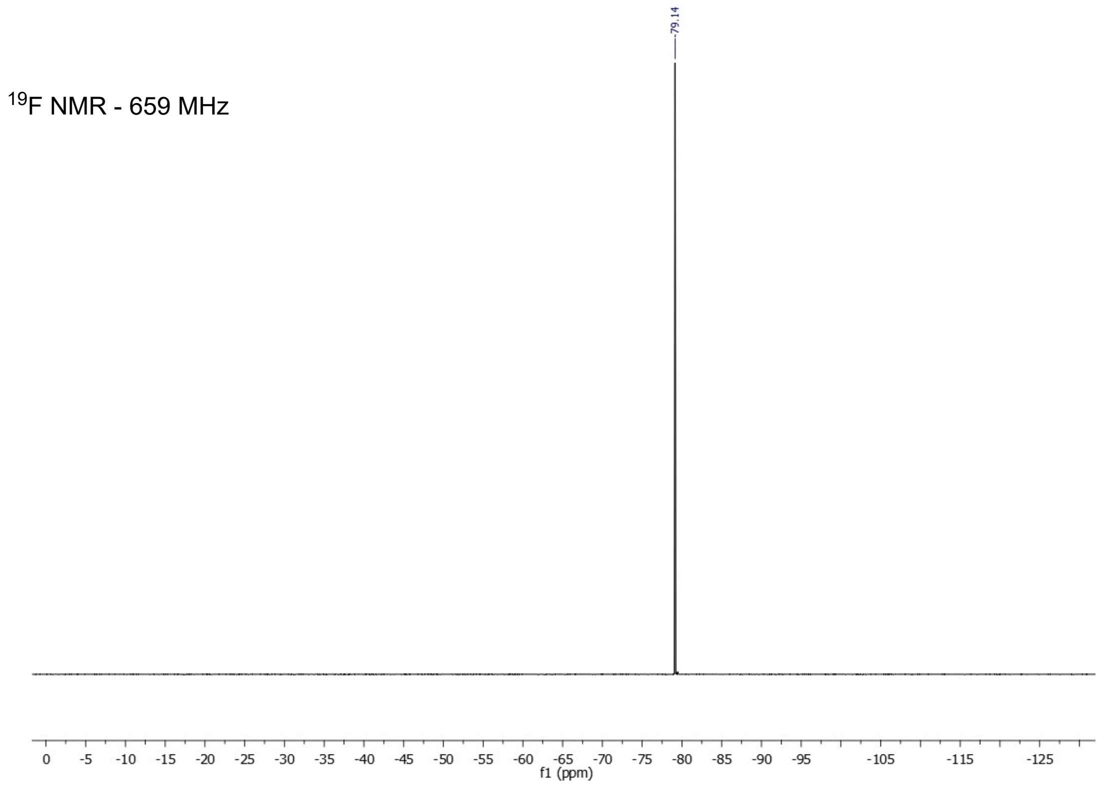

(2*R*,3*S*,5*R*)-6,6,6-trifluoro-5-hydroxy-3-(4-methoxyphenyl)-2-methyl-1-(pyrrolidin-1-yl)hexan-1-one (3j)

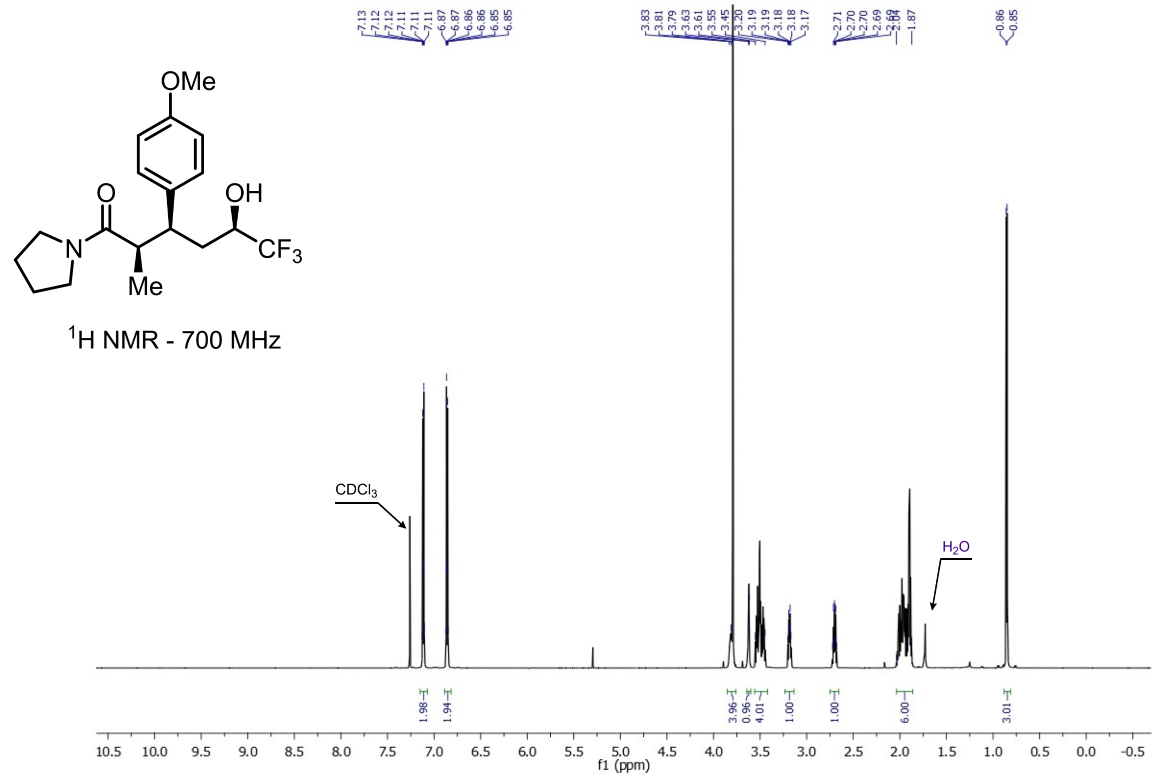

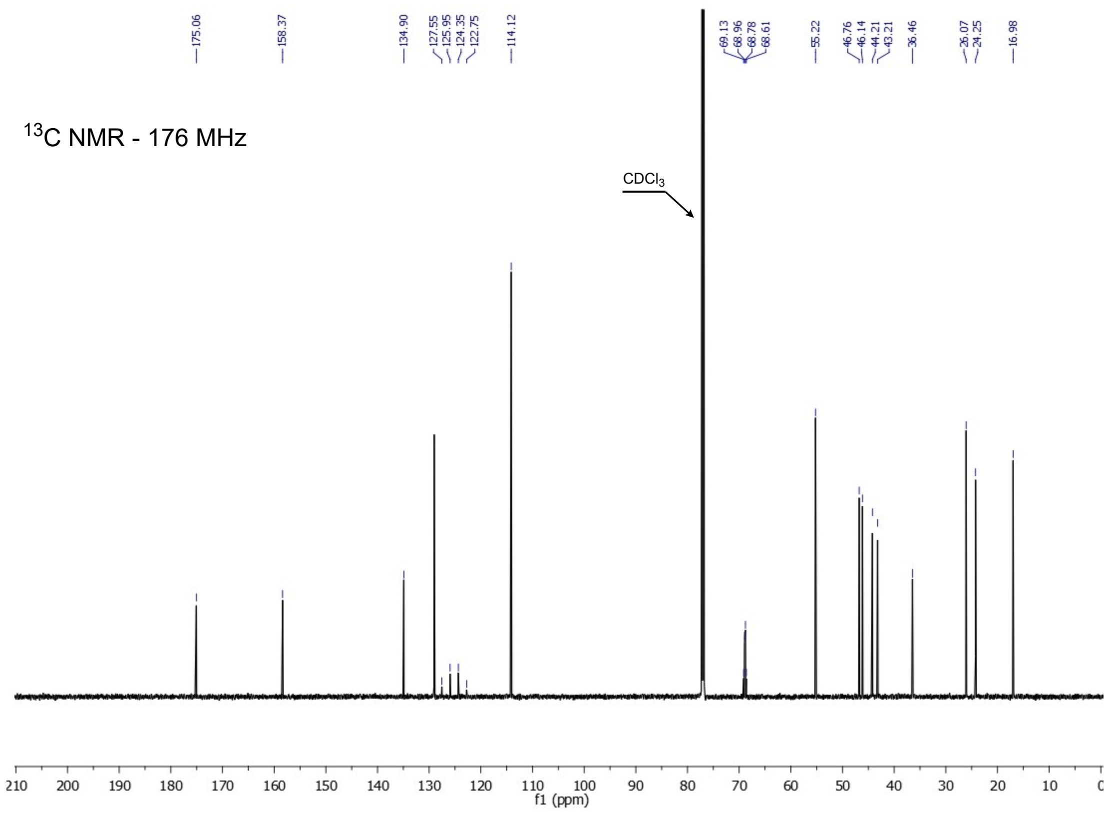
175.06
158.37
134.90
127.55
125.95
124.35
122.75
114.12
69.13
68.96
68.78
68.61
55.22
46.76
46.14
44.21
43.21
36.46
26.07
24.25
16.98
^{13}C NMR - 176 MHz
CDCl$_3$
210
200
190
180
170
160
150
140
130
120
110
100
90
80
70
60
50
40
30
20
10
f1 (ppm)

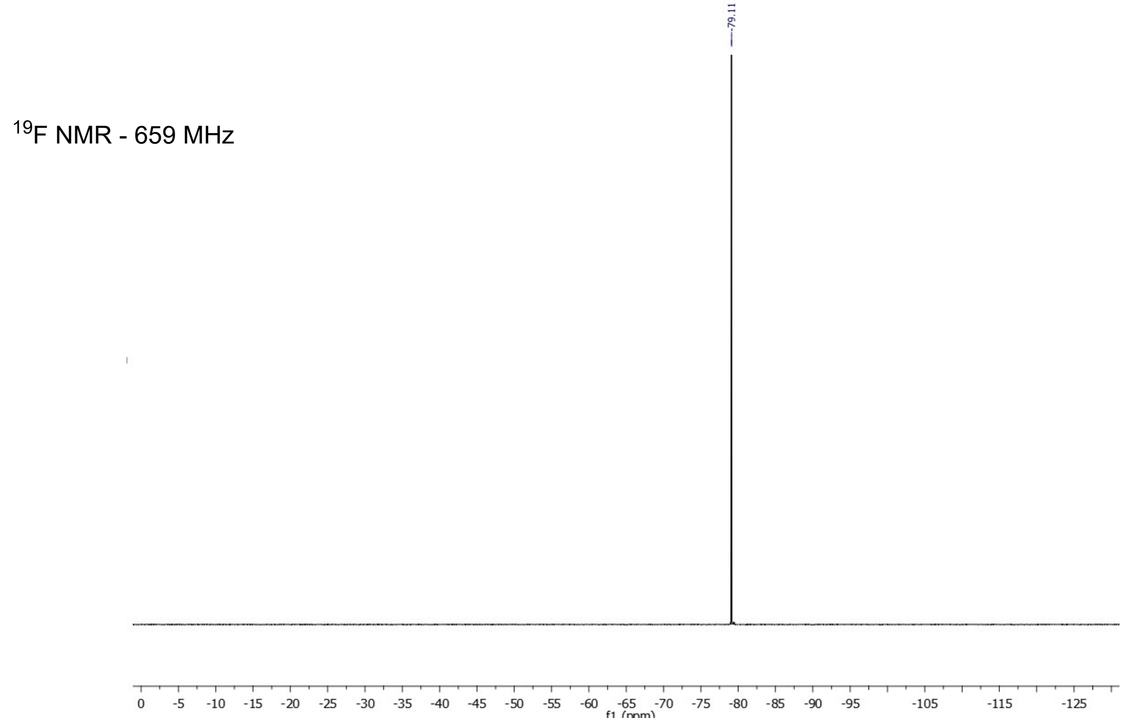
-79.11
^{19}F NMR - 659 MHz
0
-5
-10
-15
-20
-25
-30
-35
-40
-45
-50
-55
-60
-65
-70
-75
-80
-85
-90
-95
-105
-115
-125

(2*R*,3*S*,5*R*)-3-(4-bromophenyl)-6,6,6-trifluoro-5-hydroxy-2-methyl-1-(pyrrolidin-1-yl)hexan-1-one (3k)

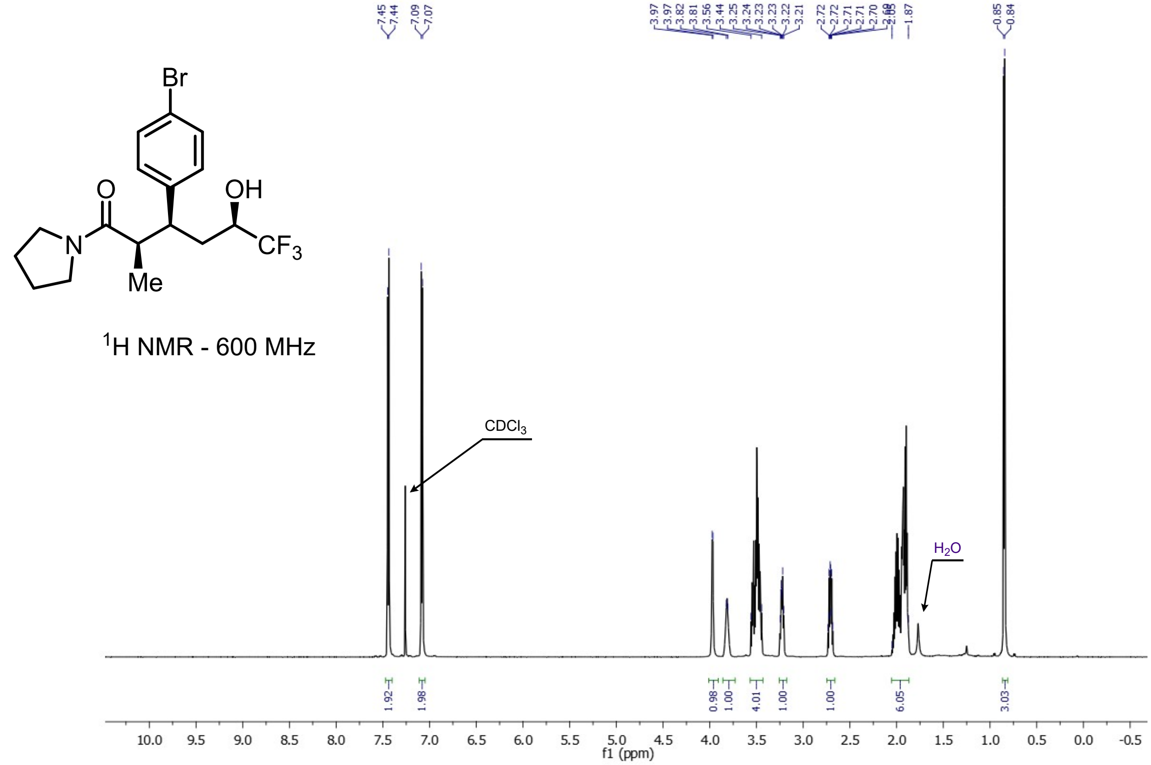

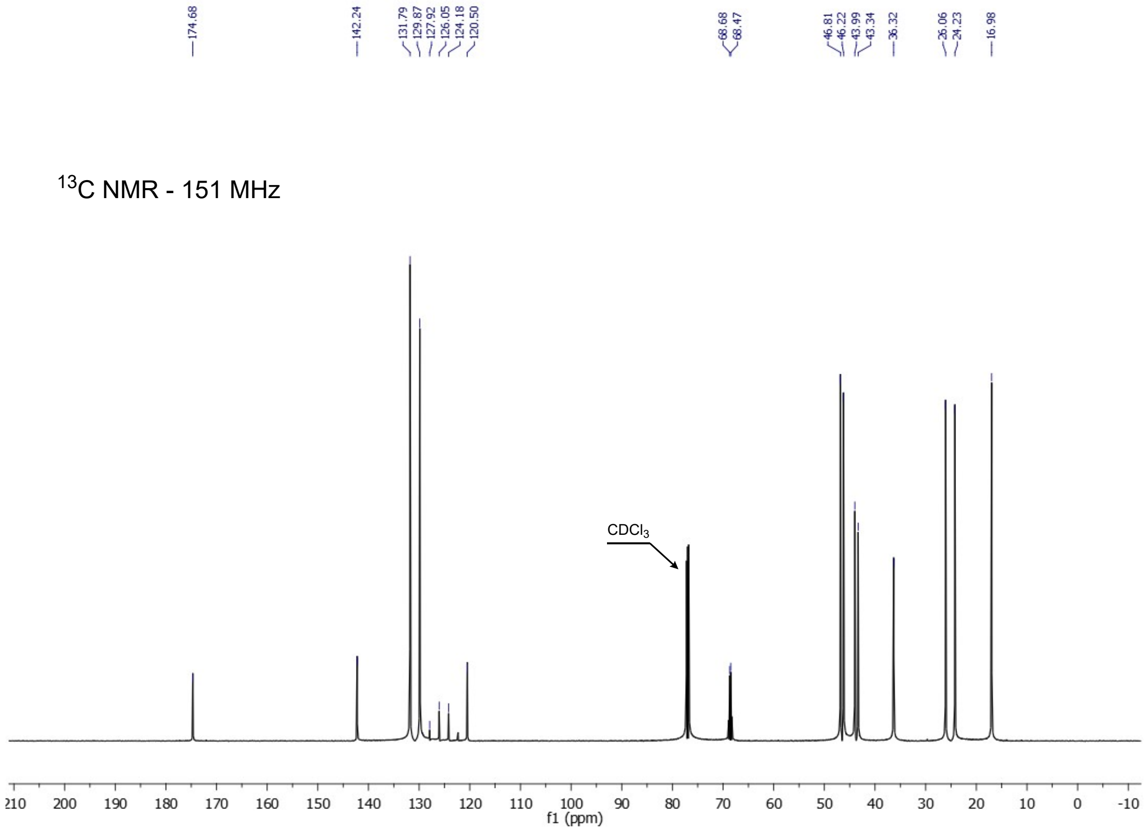

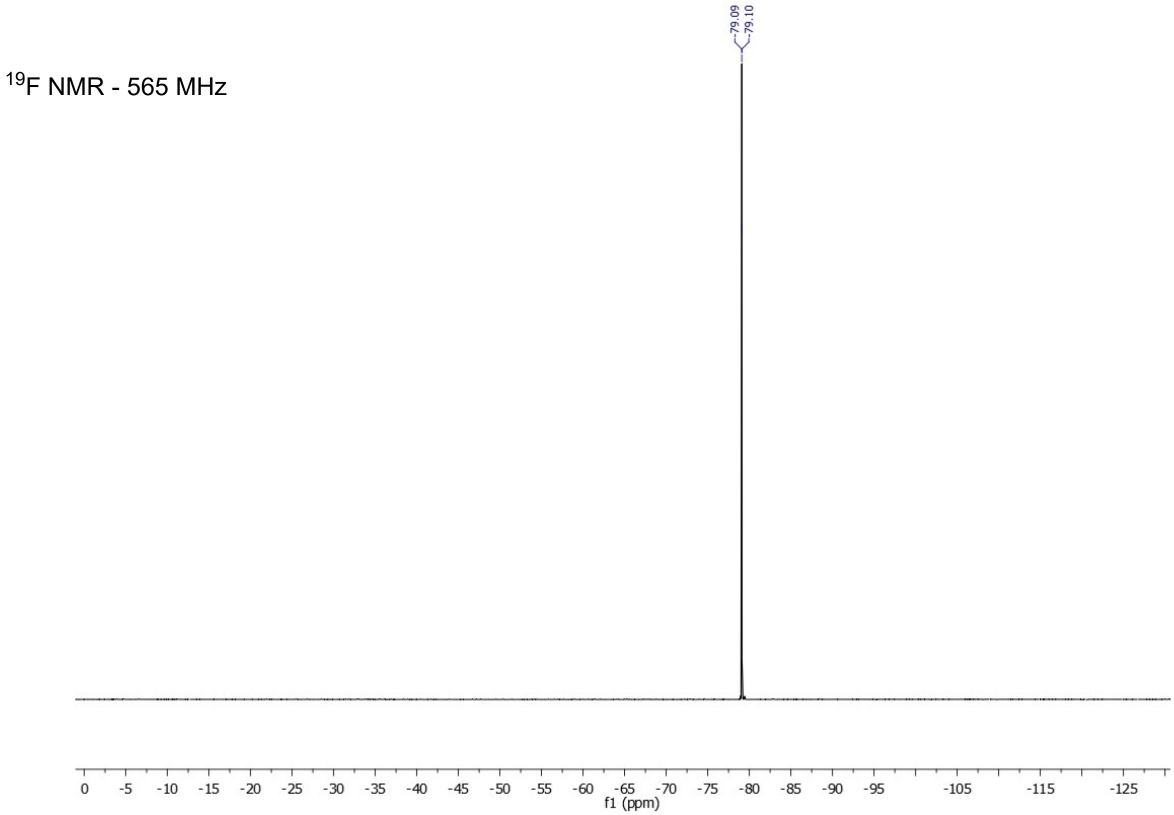

(2*R*,3*S*,5*S*)-6,6,6-trifluoro-5-hydroxy-2-methyl-3-(naphthalen-2-yl)-1-(pyrrolidin-1-yl)hexan-1-one (3l)

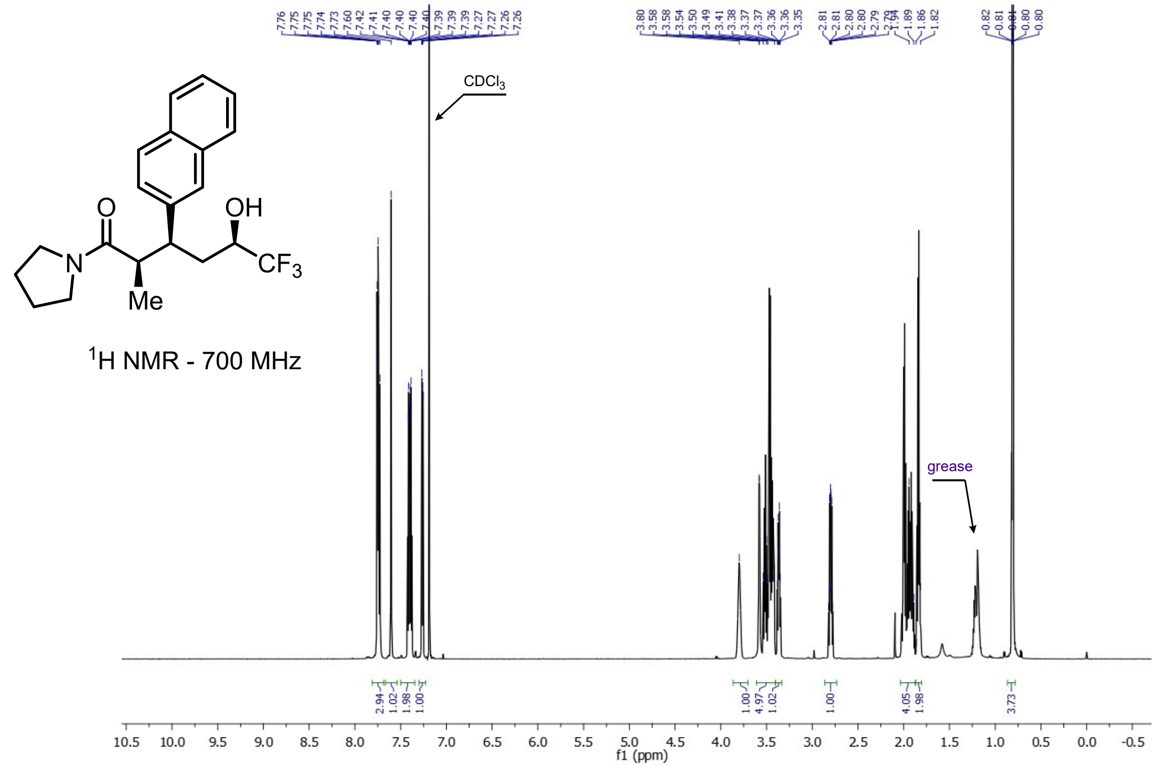

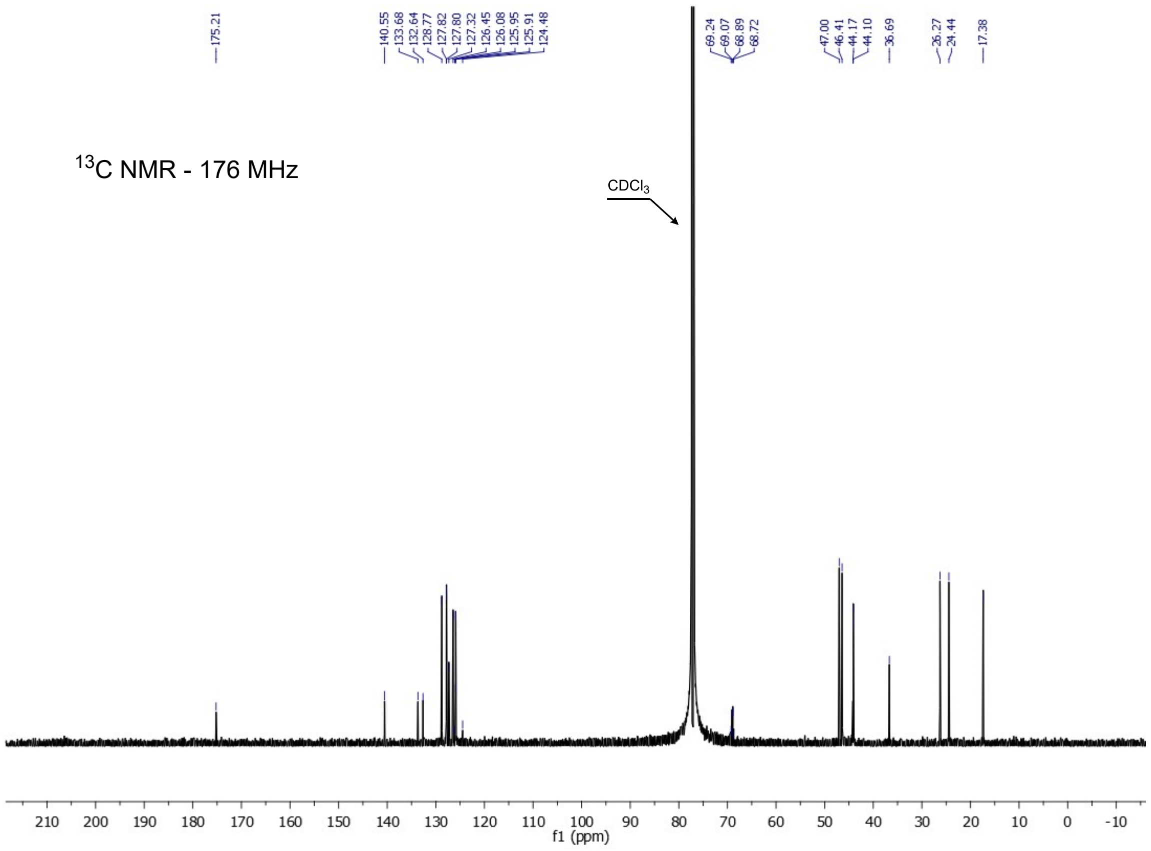
175.21
140.55
133.68
132.64
128.77
127.82
127.80
127.32
126.45
126.08
125.95
125.91
124.48
69.24
69.07
68.89
68.72
47.00
46.41
44.17
44.10
36.69
26.27
24.44
17.38
^{13}C NMR - 176 MHz
$CDCl_3$
f1 (ppm)

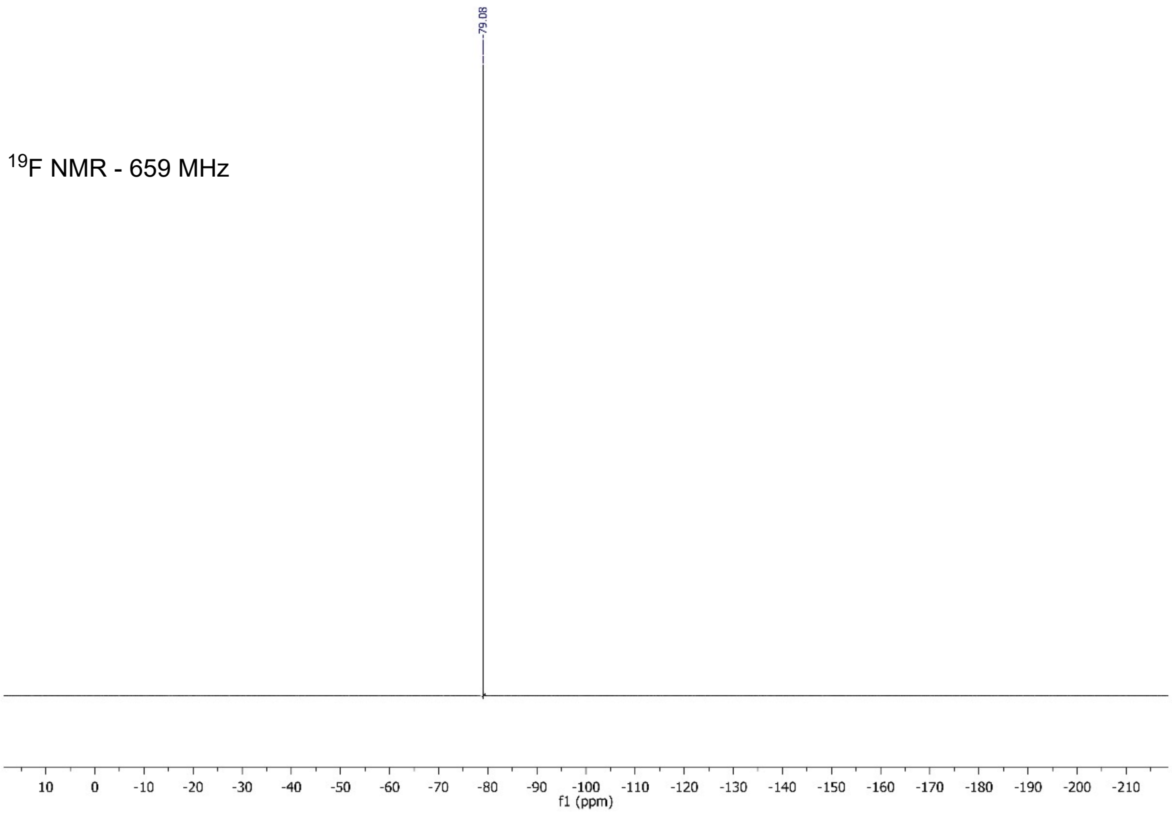
-79.08
^{19}F NMR - 659 MHz
f1 (ppm)

(2*R*,3*S*,5*R*)-6,6,6-trifluoro-3-(2-fluorophenyl)-5-hydroxy-2-methyl-1-(pyrrolidin-1-yl)hexan-1-one (3m)

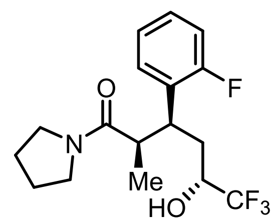

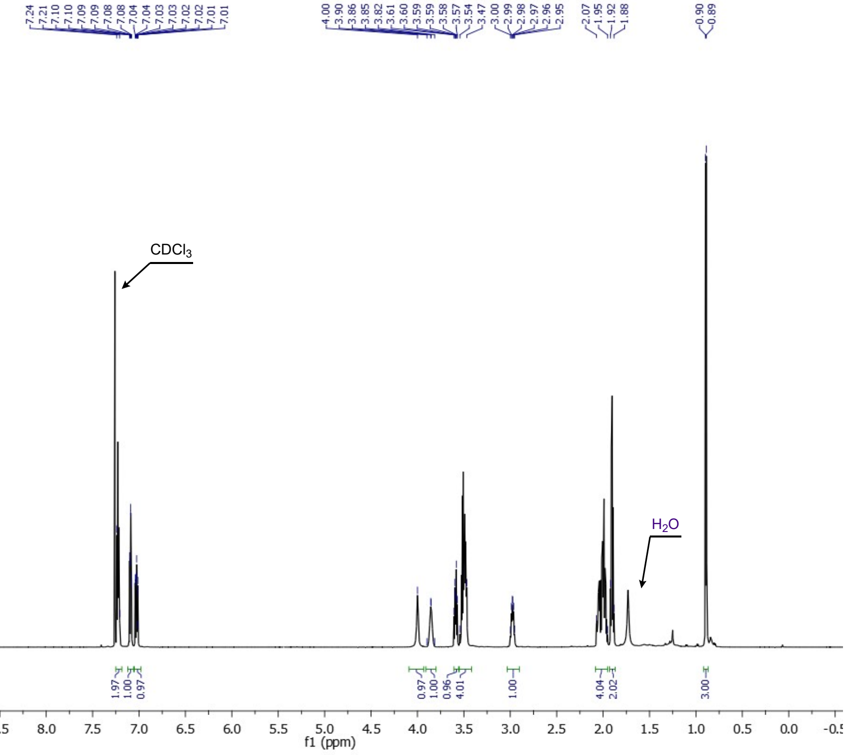

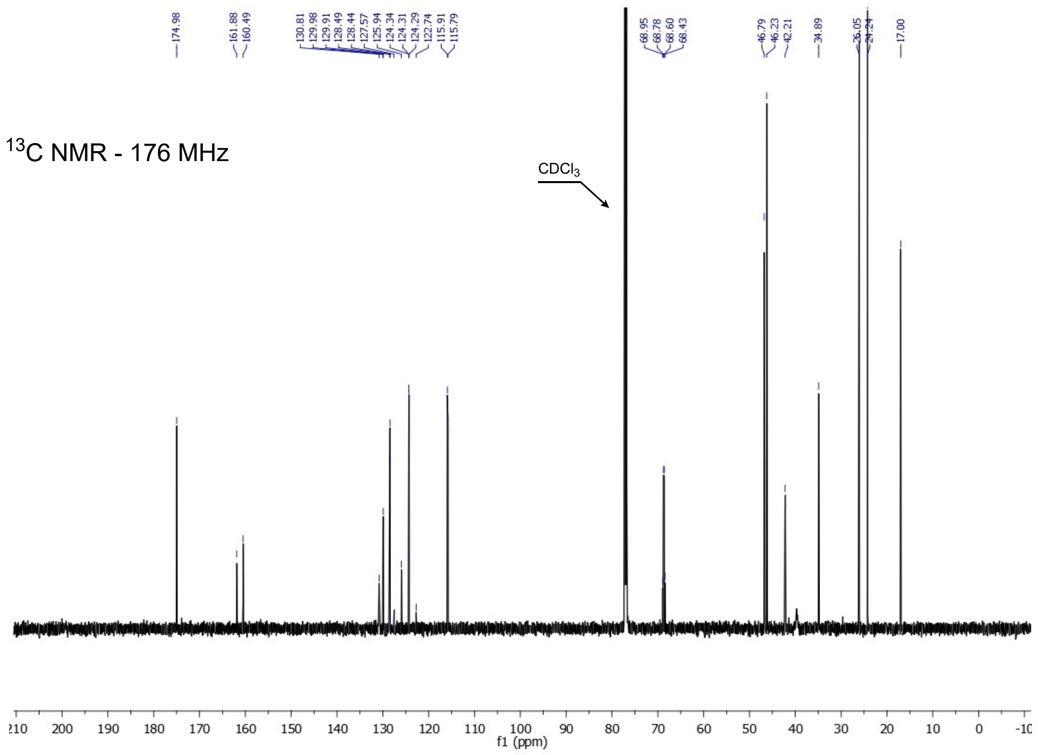

^{19}F NMR - 659 MHz

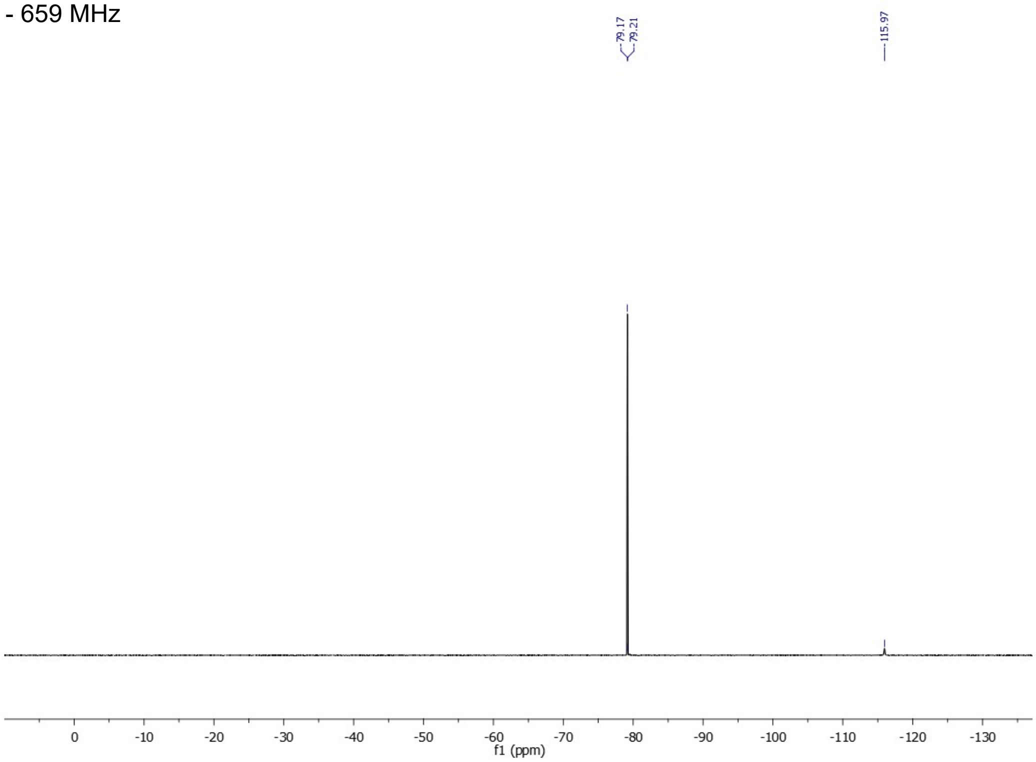

(2*R*,3*S*,5*R*)-6,6,6-trifluoro-5-hydroxy-2-isopropyl-3-phenyl-1-(pyrrolidin-1-yl)hexan-1-one (3n)

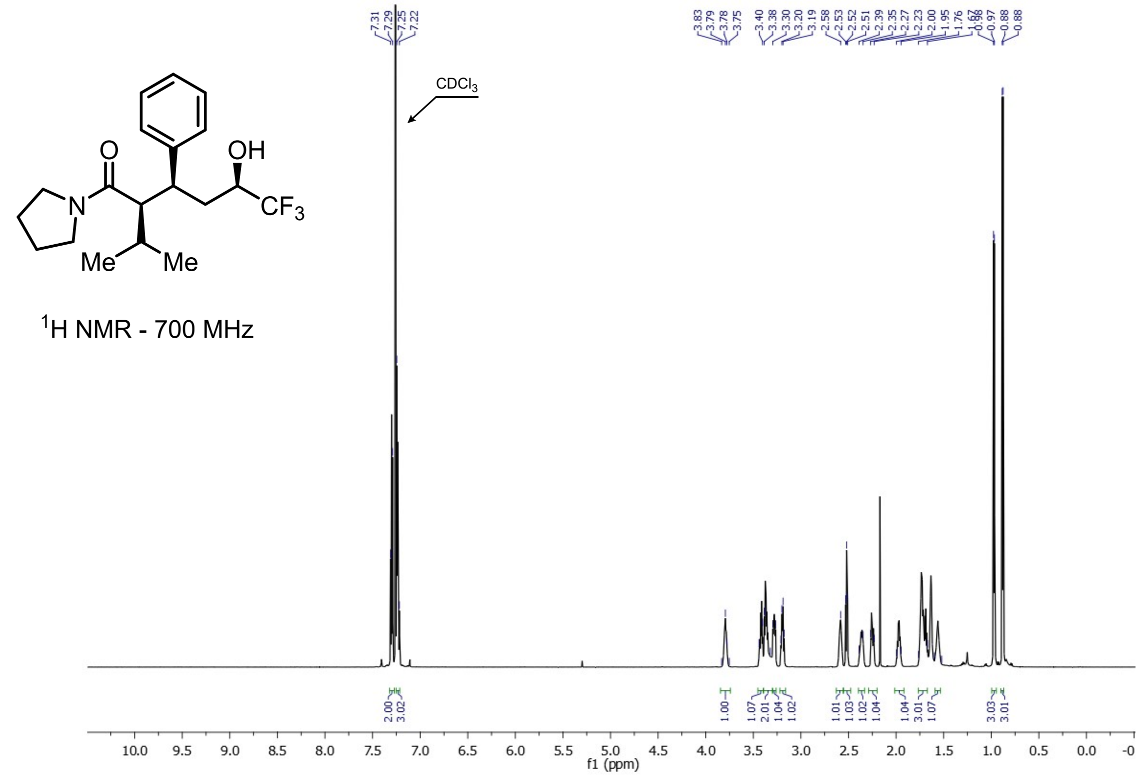

^{1}H NMR - 700 MHz

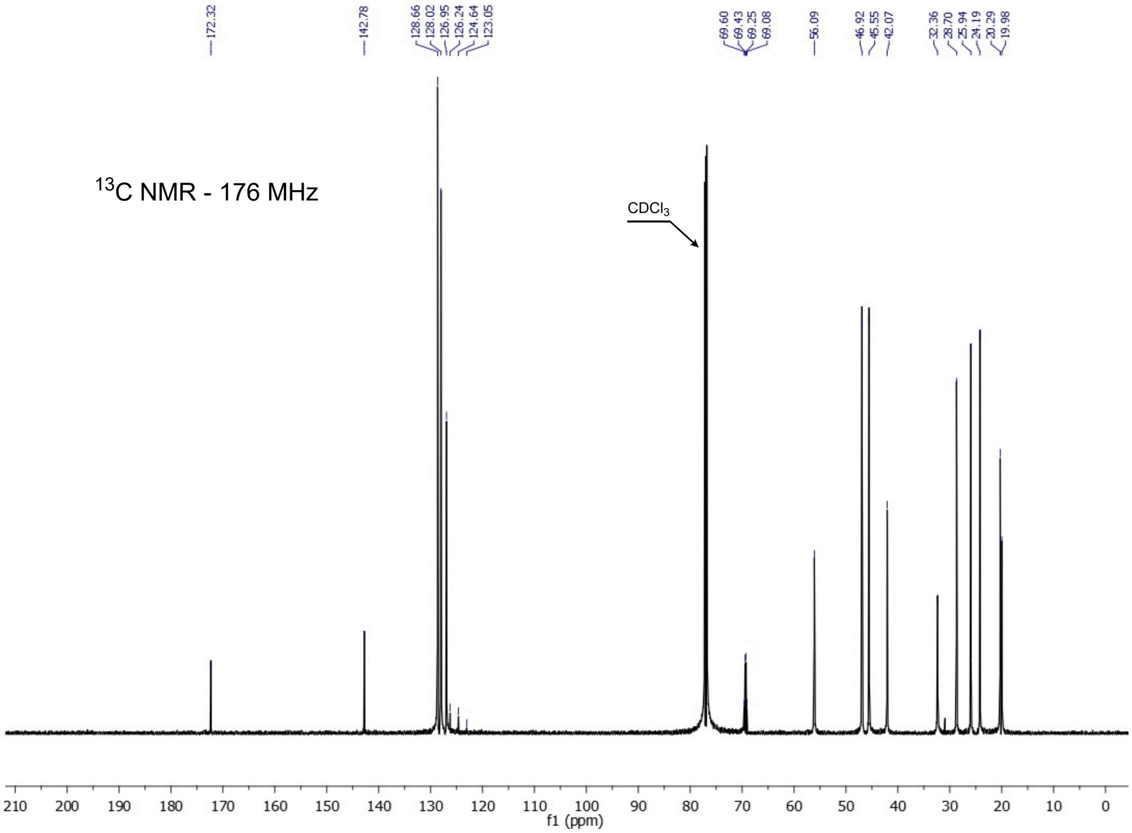
172.32
142.78
128.66
128.02
126.95
126.24
124.64
123.05
69.60
69.43
69.25
69.08
56.09
46.92
45.55
42.07
32.36
28.70
25.94
24.19
20.29
19.98
^{13}C NMR - 176 MHz
$CDCl_3$
f1 (ppm)

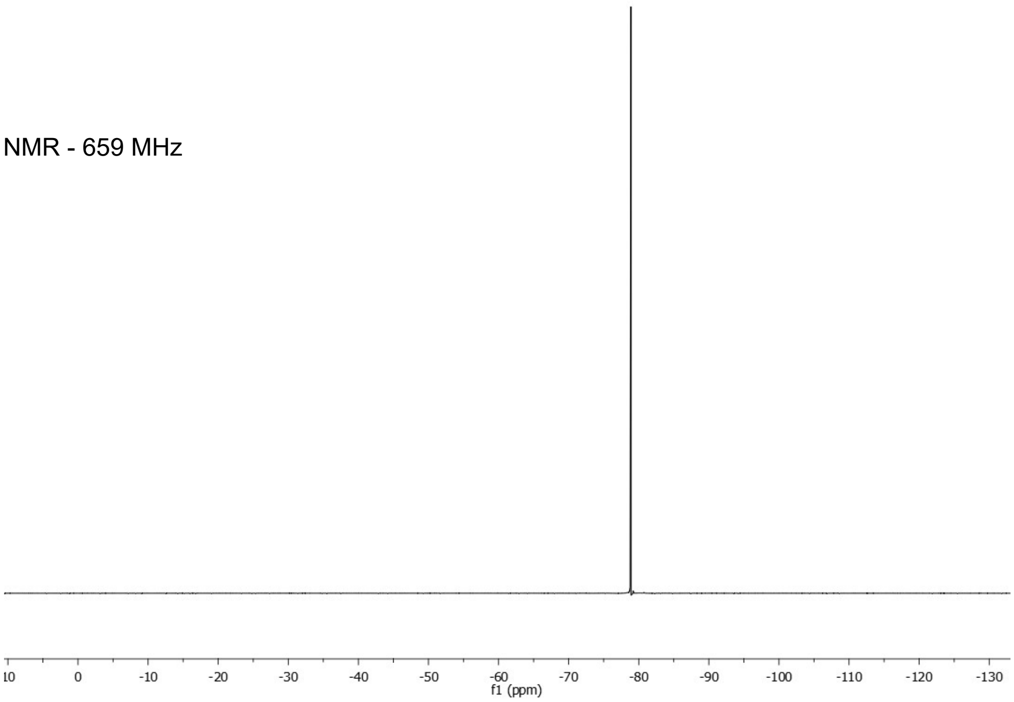
^{19}F NMR - 659 MHz
f1 (ppm)

(2*R*,3*S*,5*R*)-6,6,6-trifluoro-5-hydroxy-2-isopropyl-3-phenyl-1-(piperidin-1-yl)hexan-1-one (3o)

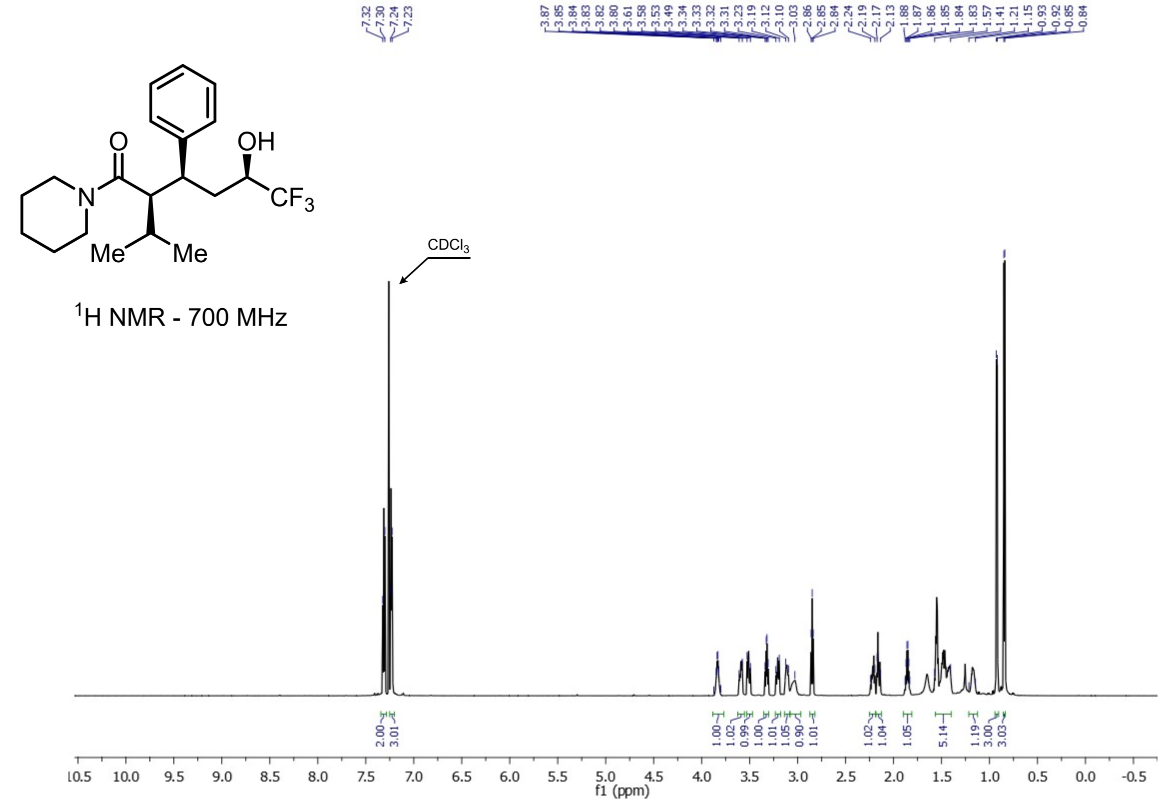

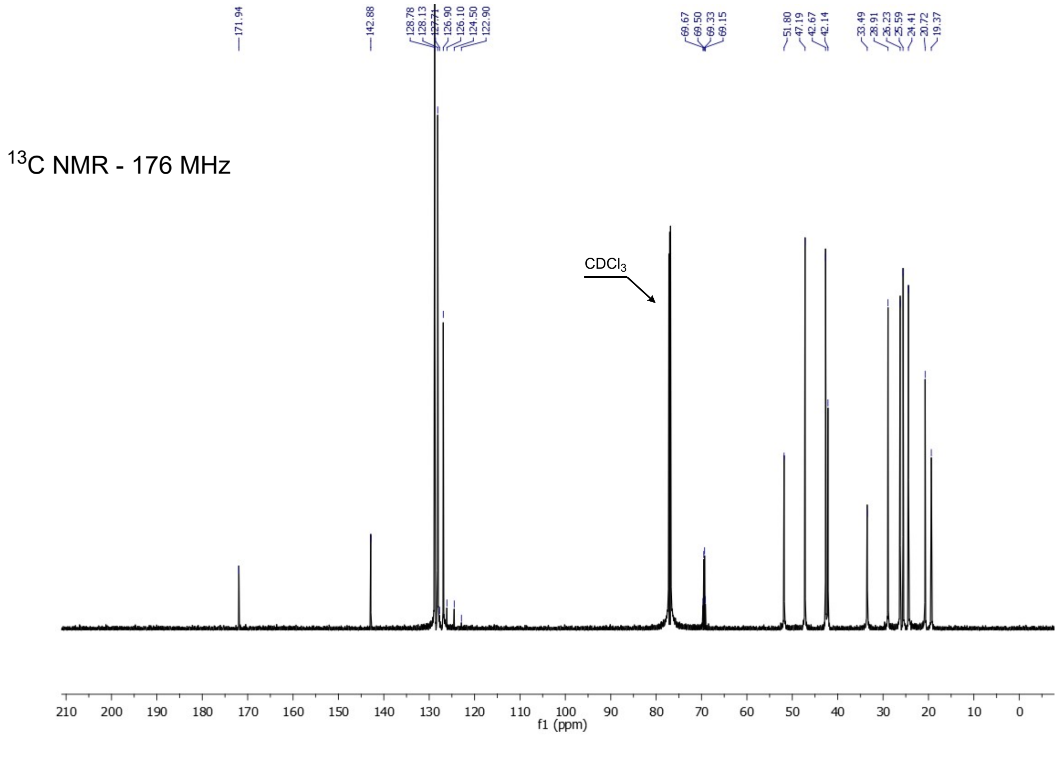

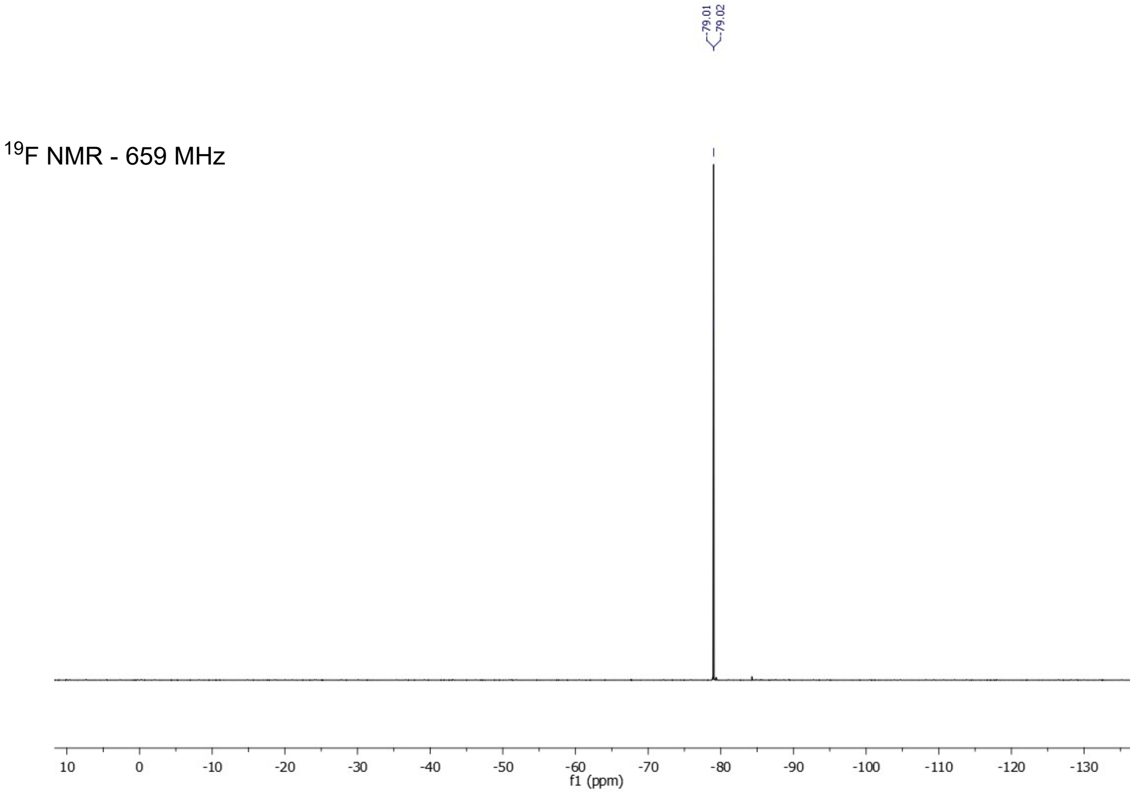

(2*R*,3*S*,5*R*)-*N*,*N*-diethyl-6,6,6-trifluoro-5-hydroxy-2-isopropyl-3-phenylhexanamide (3p)

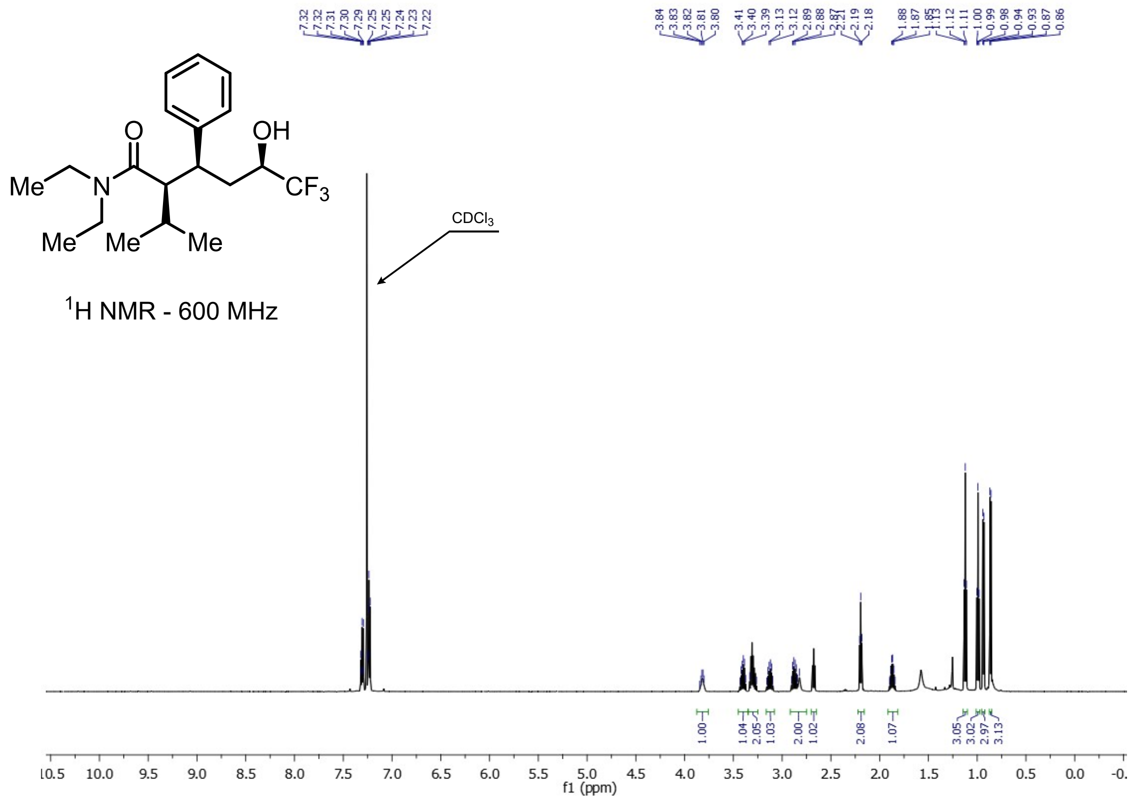

^{13}C NMR - 151 MHz

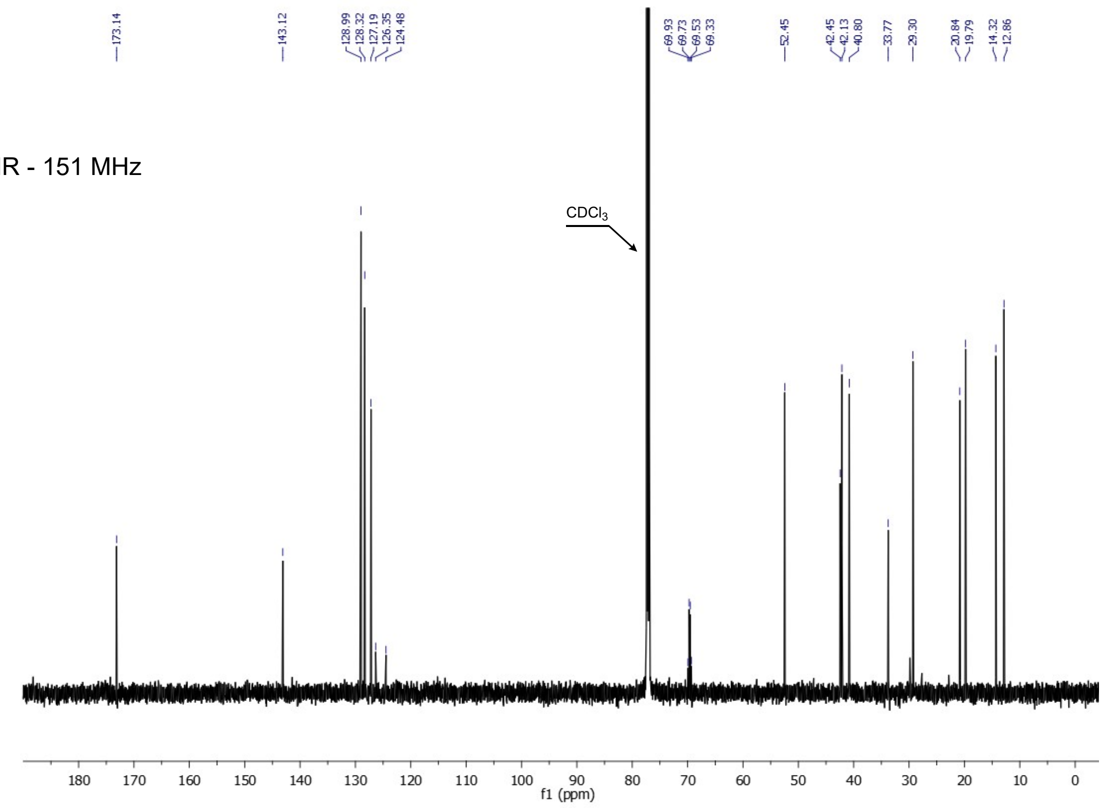

^{19}F NMR - 565 MHz

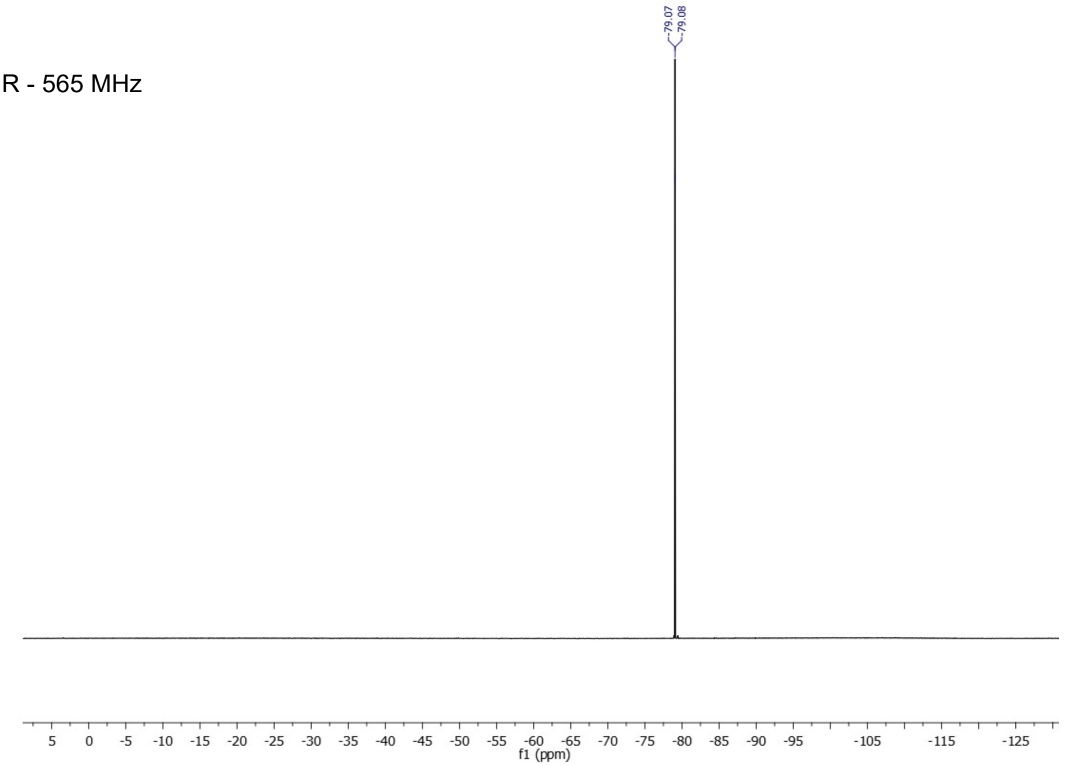

(2R,3S,5R)-6,6,6-trifluoro-5-hydroxy-2-isopropyl-3-phenyl-1-(1,4-dioxa-8-azaspiro[4.5]decan-8-yl)hexan-1-one (3q)

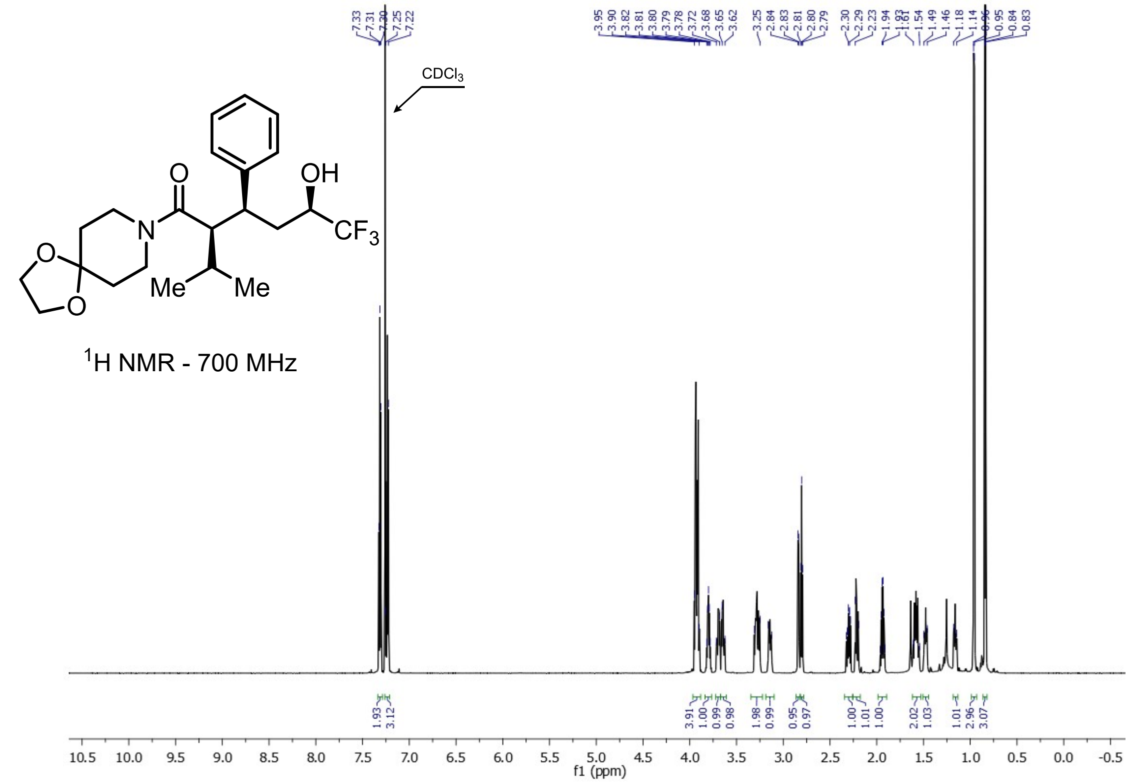

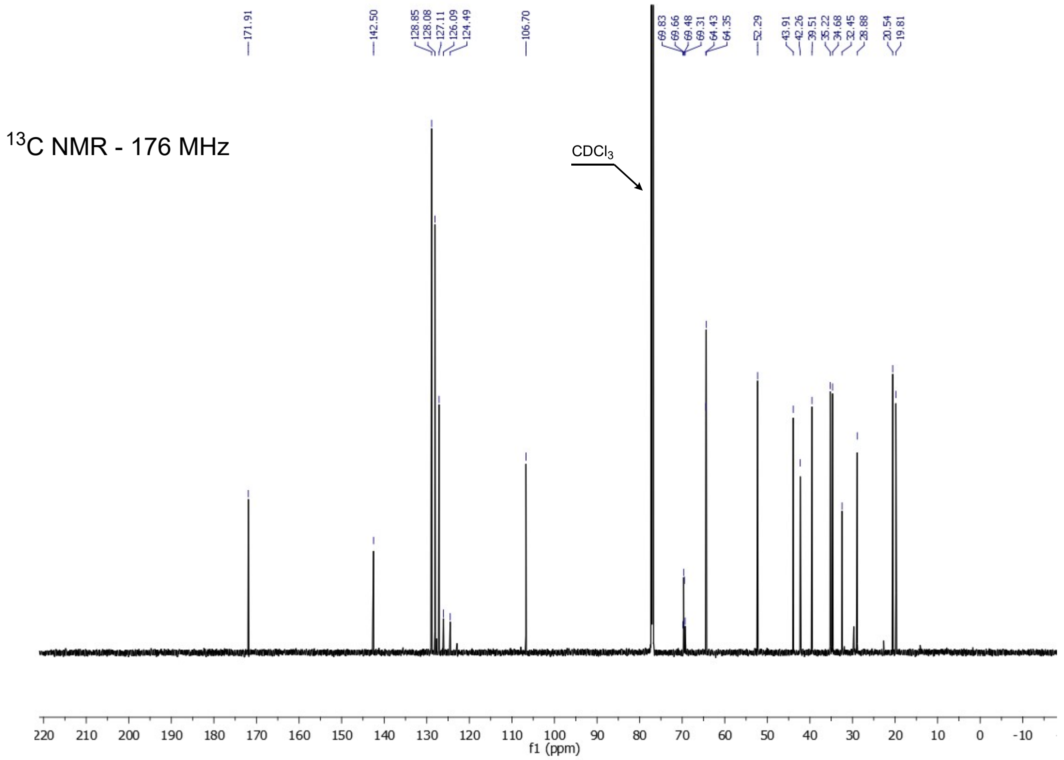

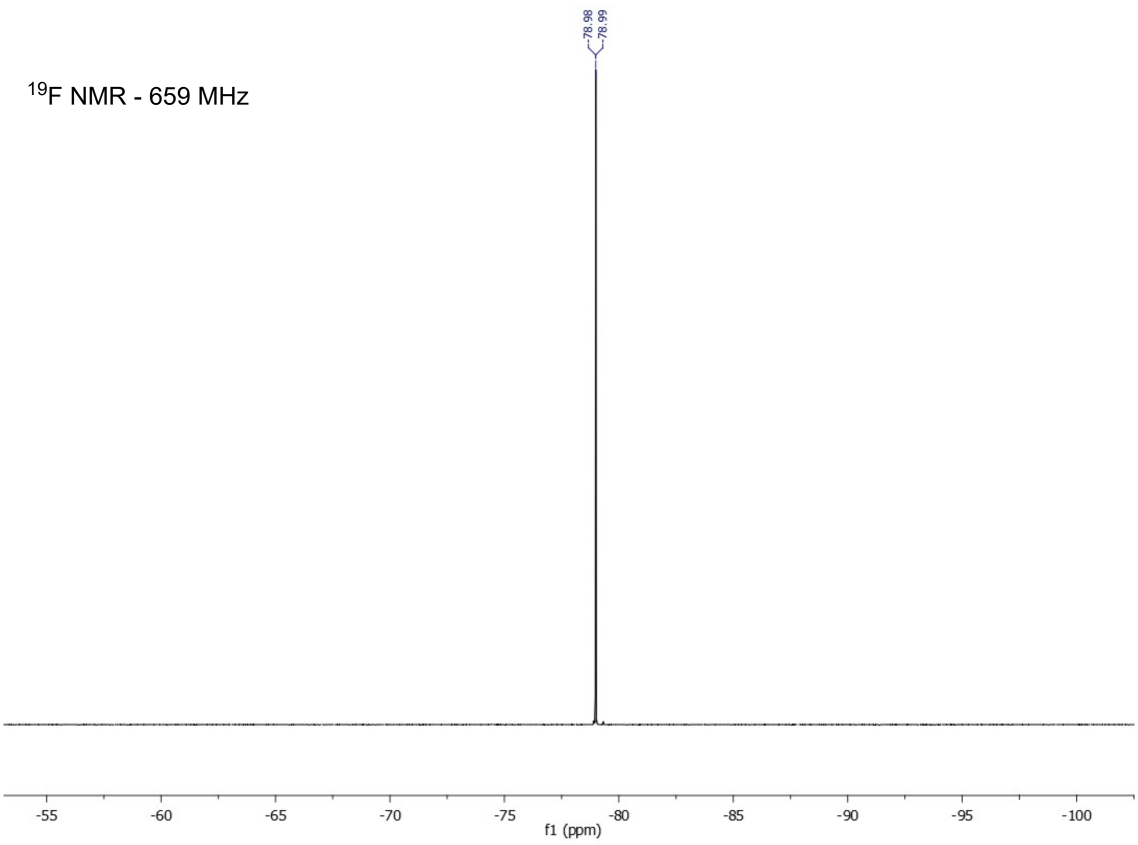
19F NMR - 659 MHz
-78.98
-78.99
-55
-60
-65
-70
-75
-80
-85
-90
-95
-100
f1 (ppm)

9.3 Post functionalization Compounds

(2*R*,4*S*,5*R*)-1,1,1-trifluoro-5-methyl-4-phenyl-6-(pyrrolidin-1-yl)hexan-2-ol (6a)

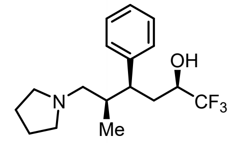

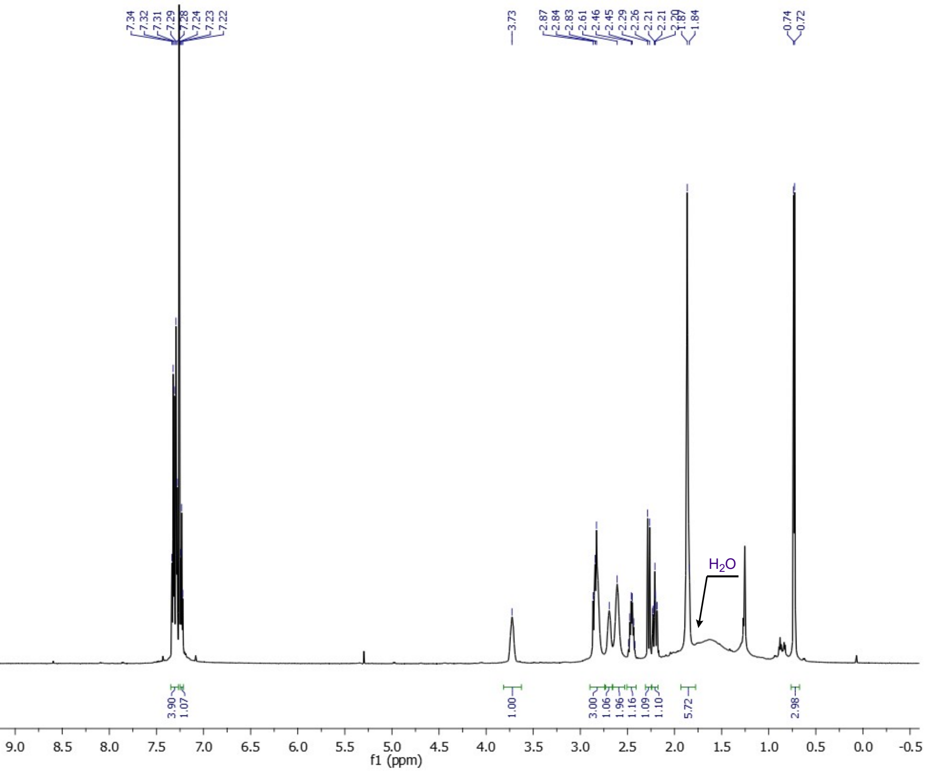

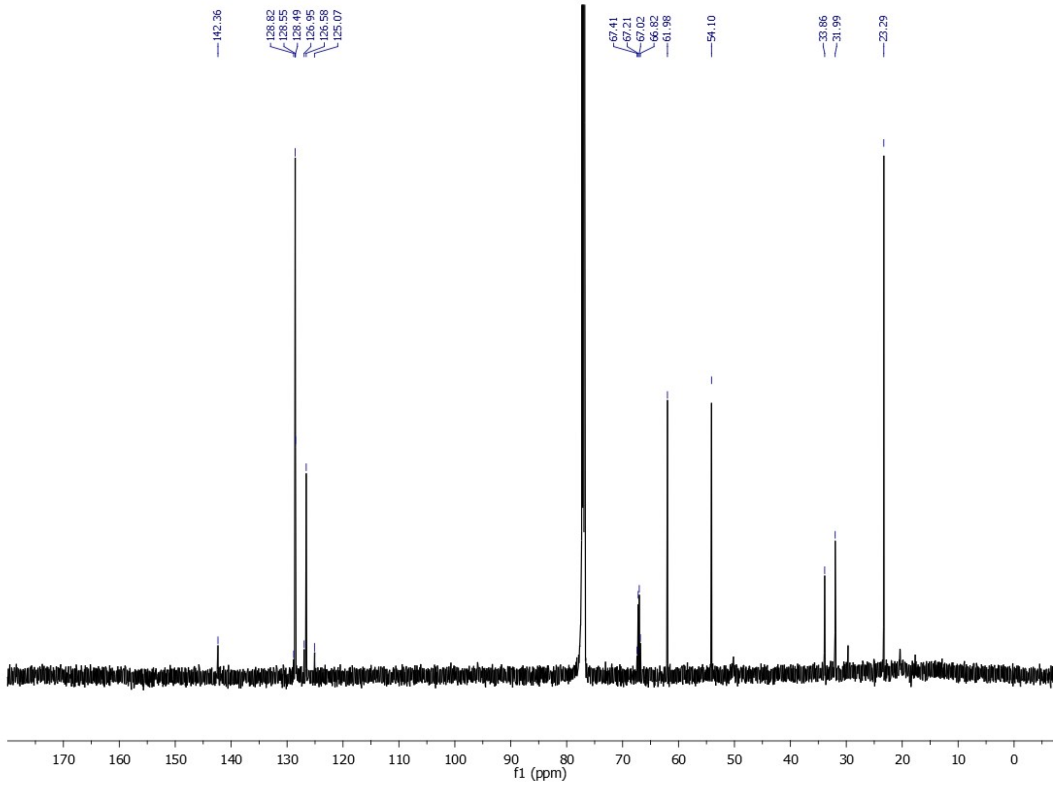

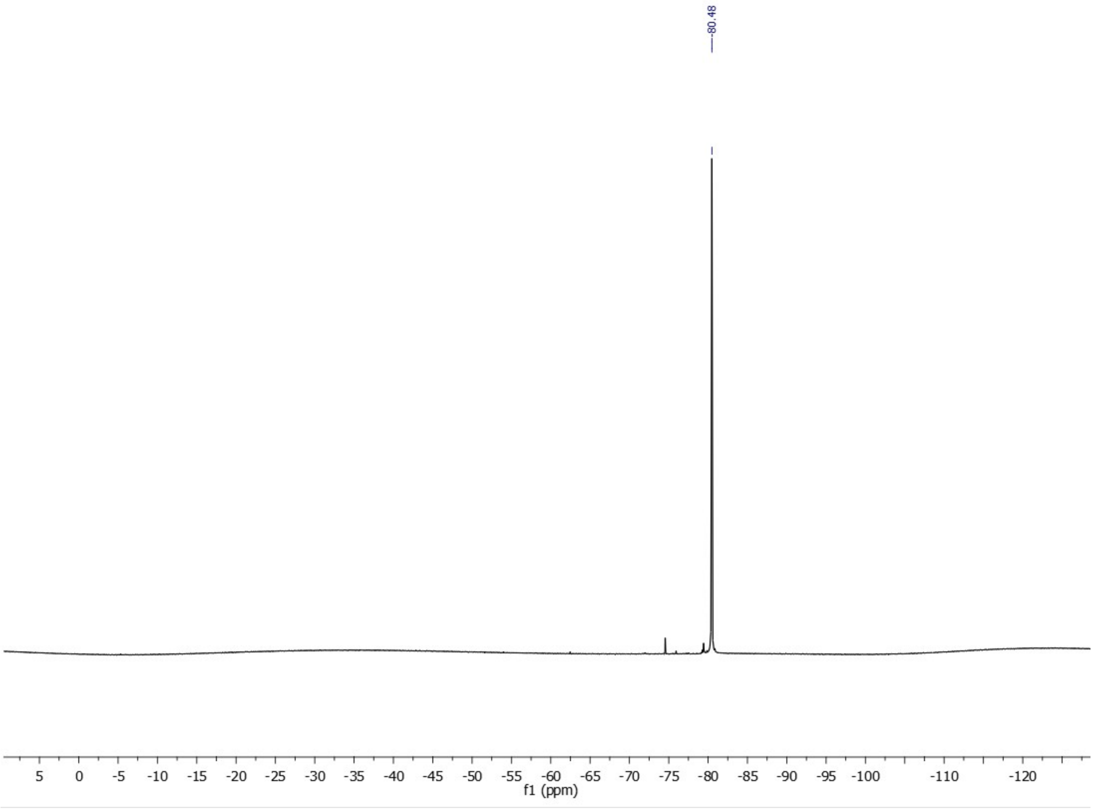
-80.48
5 0 -5 -10 -15 -20 -25 -30 -35 -40 -45 -50 -55 -60 -65 -70 -75 -80 -85 -90 -95 -100 -110 -120
f1 (ppm)

9.4 Mechanistically relevant Compounds

(2*S*,4*R*,5*S*,6*R*)-5-methyl-4-phenyl-6-(pyrrolidin-1-yl)-2-(trifluoromethyl)tetrahydro-2H-pyran-2-ol (4a)

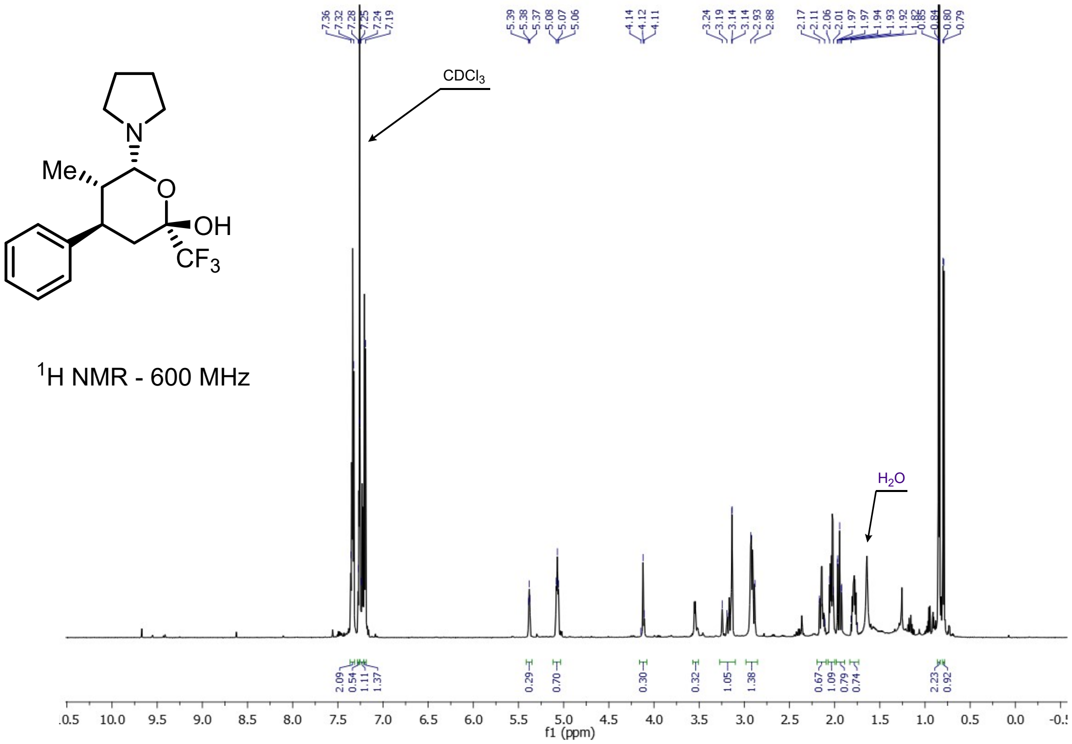

^{1}H NMR - 600 MHz

^{13}C NMR - 151 MHz

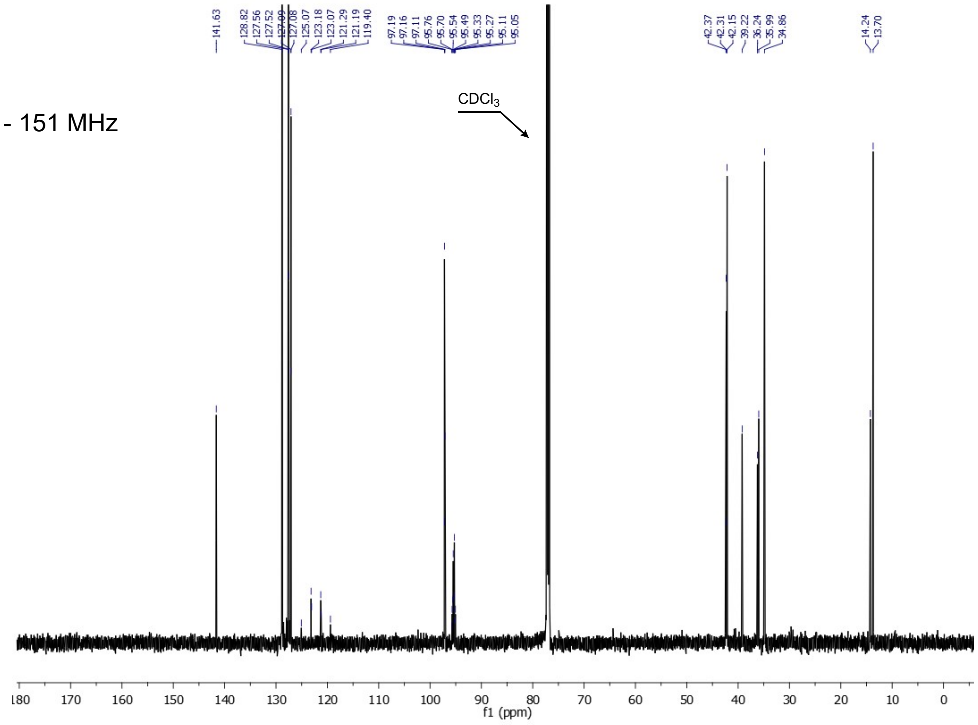

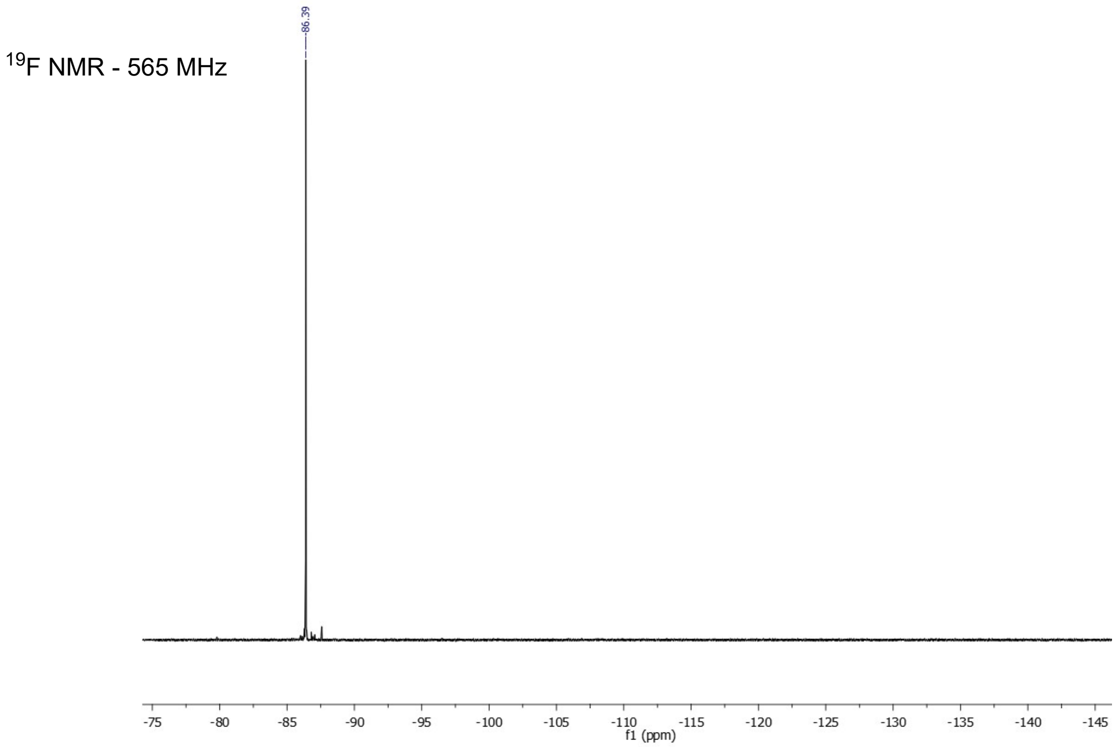

(2S,4S,5R)-6-(1H-indazol-1-yl)-5-methyl-4-phenyl-2-(trifluoromethyl)tetrahydro-2H-pyran-2-ol (4r)

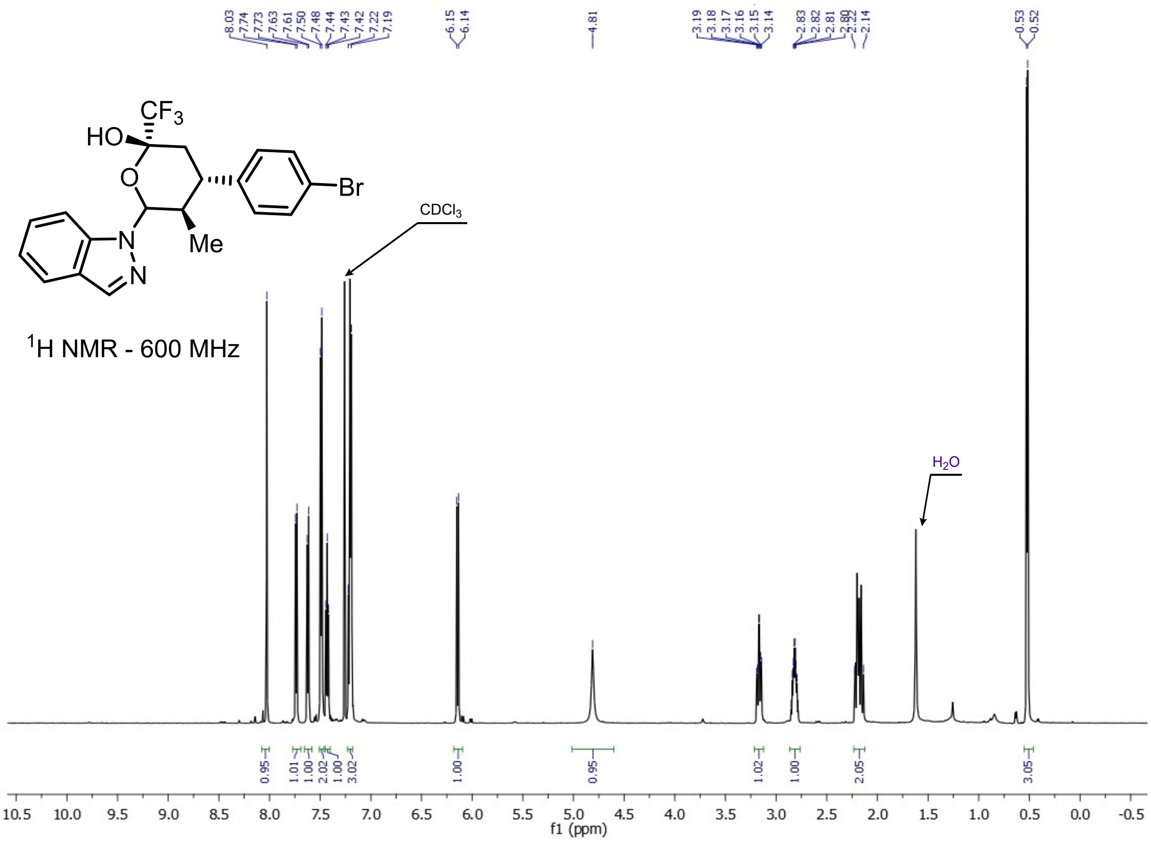

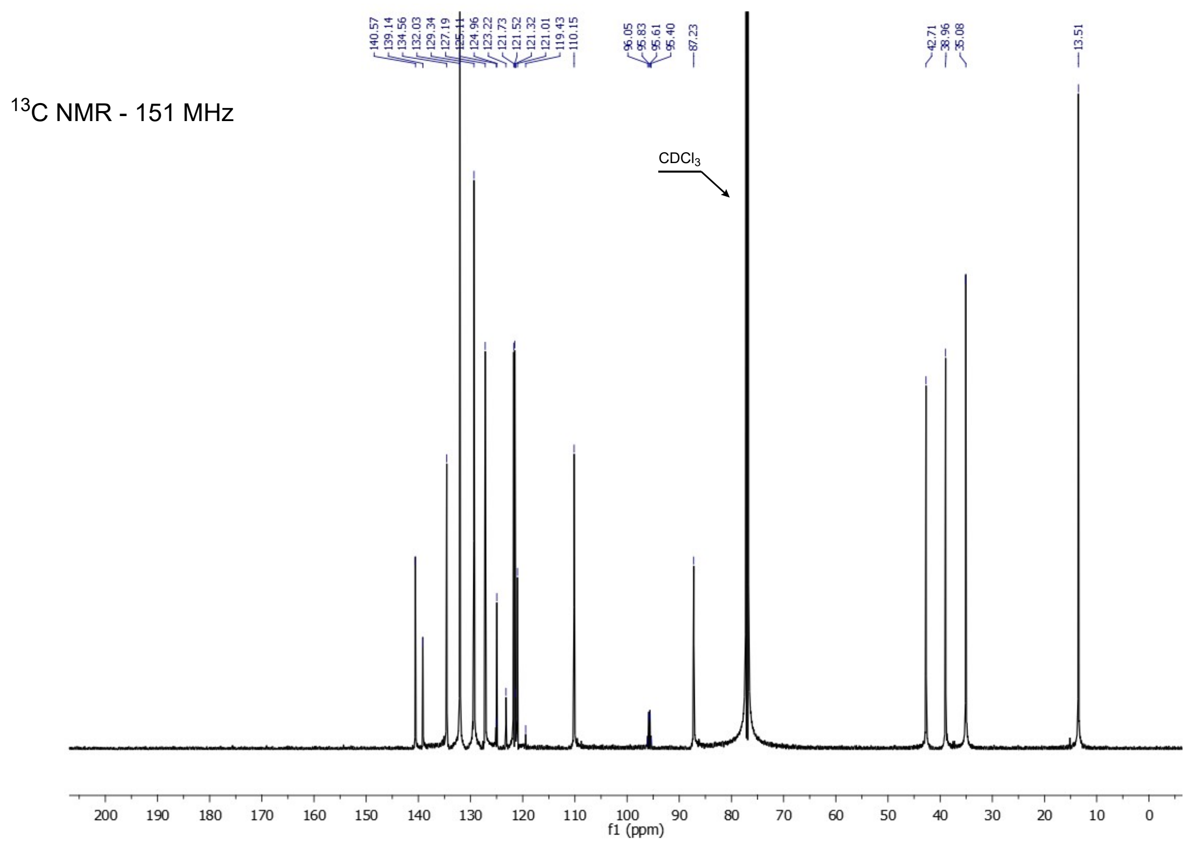

13C NMR - 151 MHz
140.57
139.14
134.56
132.03
129.34
127.19
125.11
124.96
123.22
121.73
121.52
121.32
121.01
119.43
110.15
96.05
95.83
95.61
95.40
87.23
42.71
38.96
35.08
13.51
CDCl3
f1 (ppm)

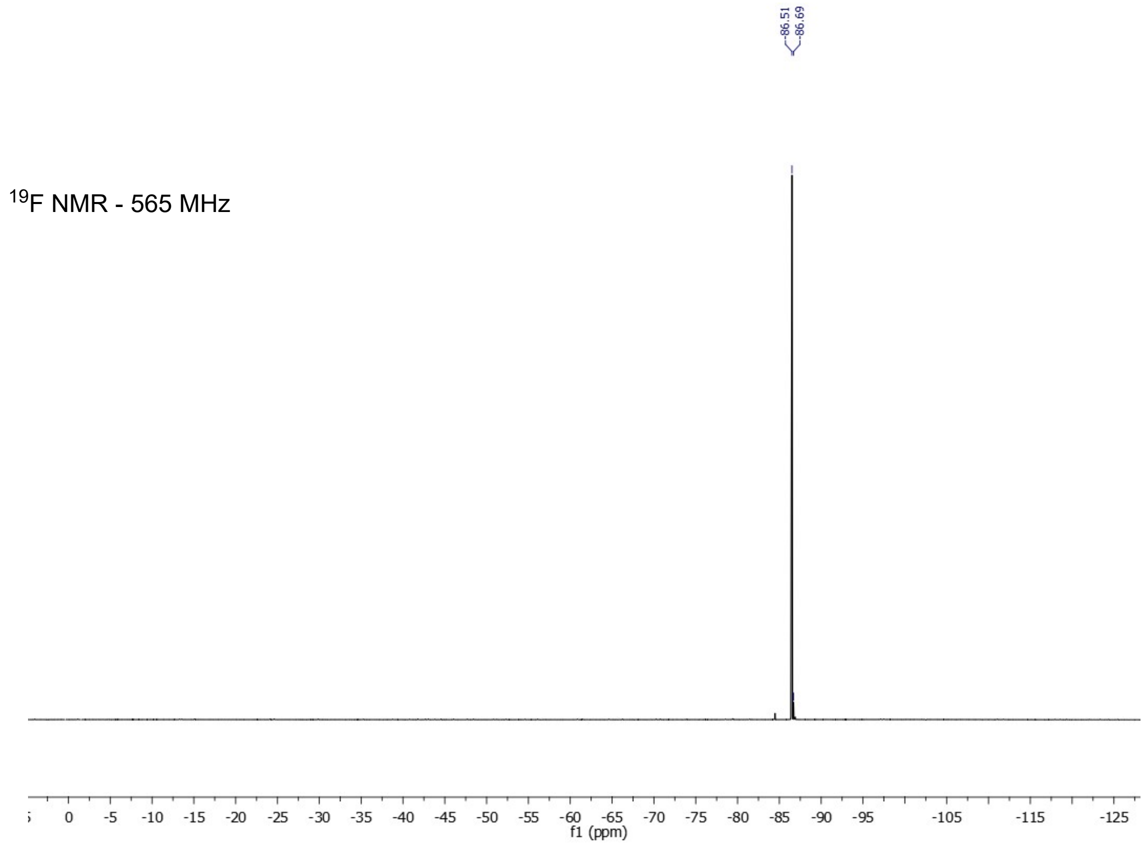

19F NMR - 565 MHz
-86.51
-86.69
f1 (ppm)

(2*R*,3*S*,5*S*)-6,6,6-trifluoro-5-hydroxy-2-methyl-3-phenyl-1-(piperidin-1-yl-d10)hexan-1-one-4-d (8)

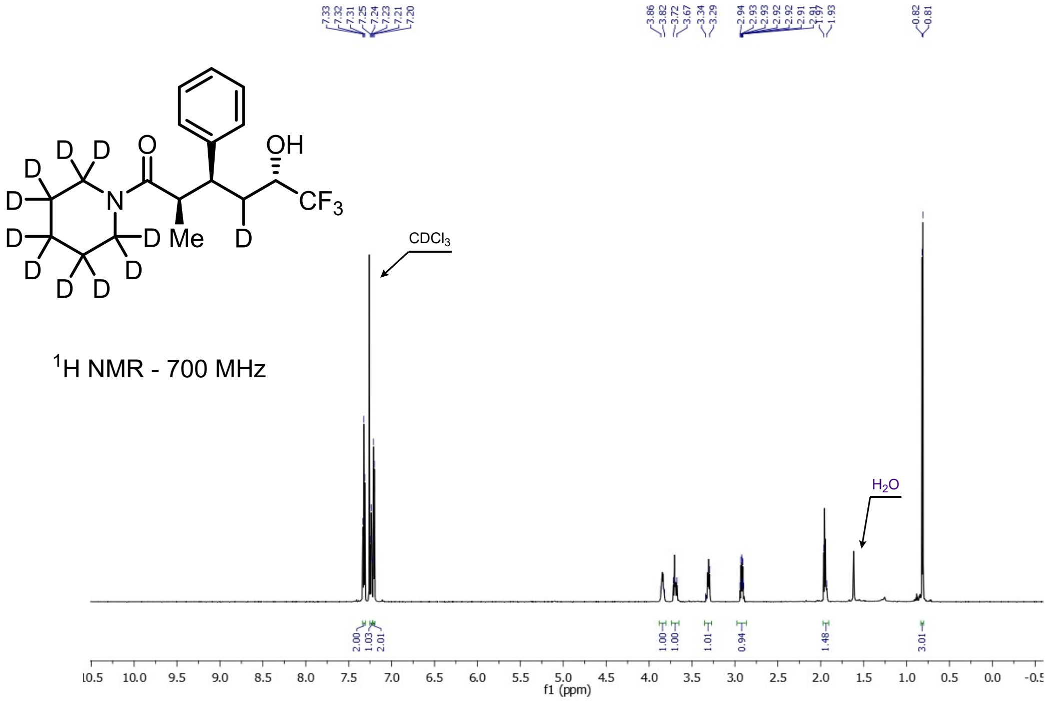

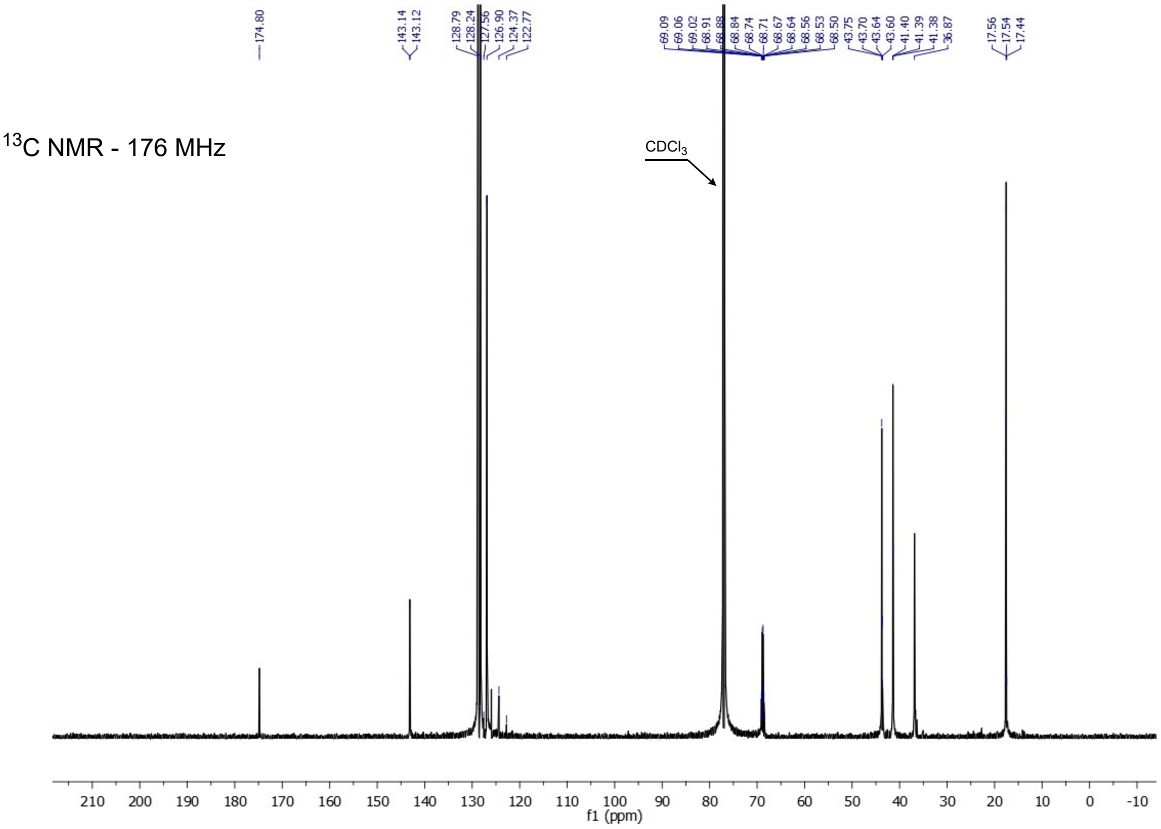

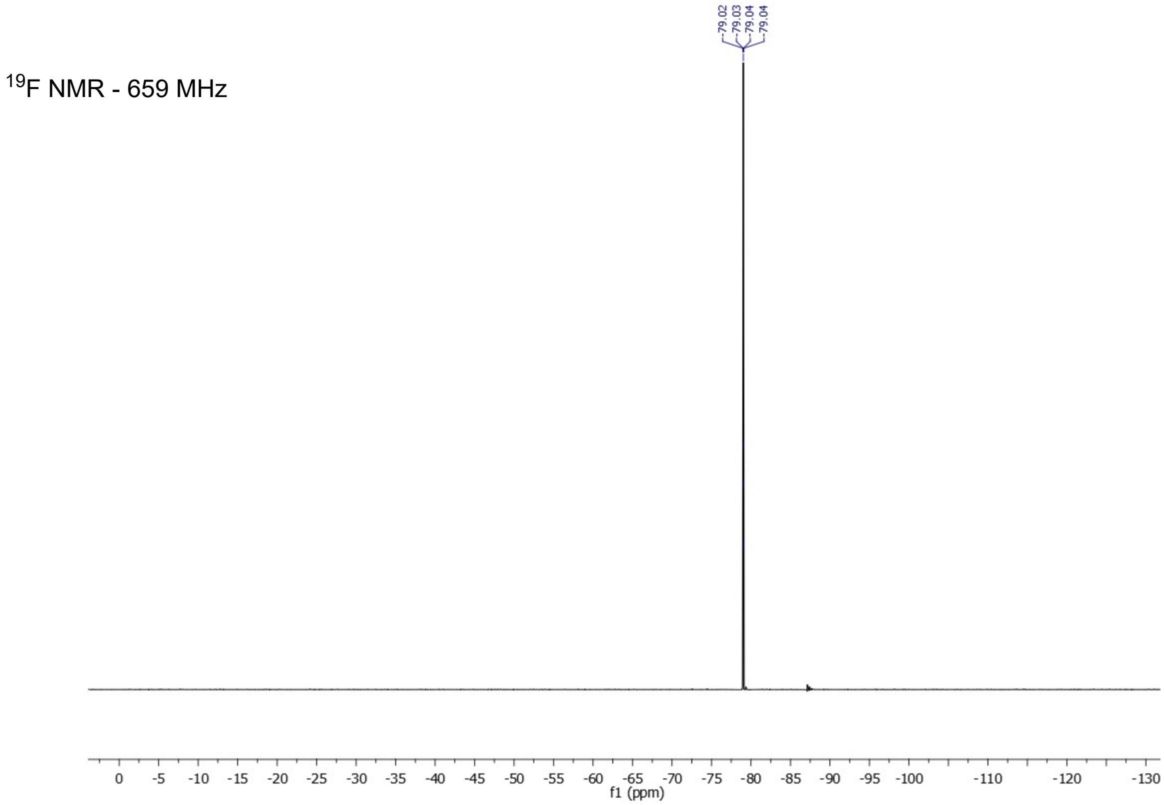

(2*R*,3*S*,5*S*)-6,6,6-trifluoro-5-hydroxy-2-methyl-3-phenyl-1-(pyrrolidin-1-yl)hexan-1-one-5-d (9)

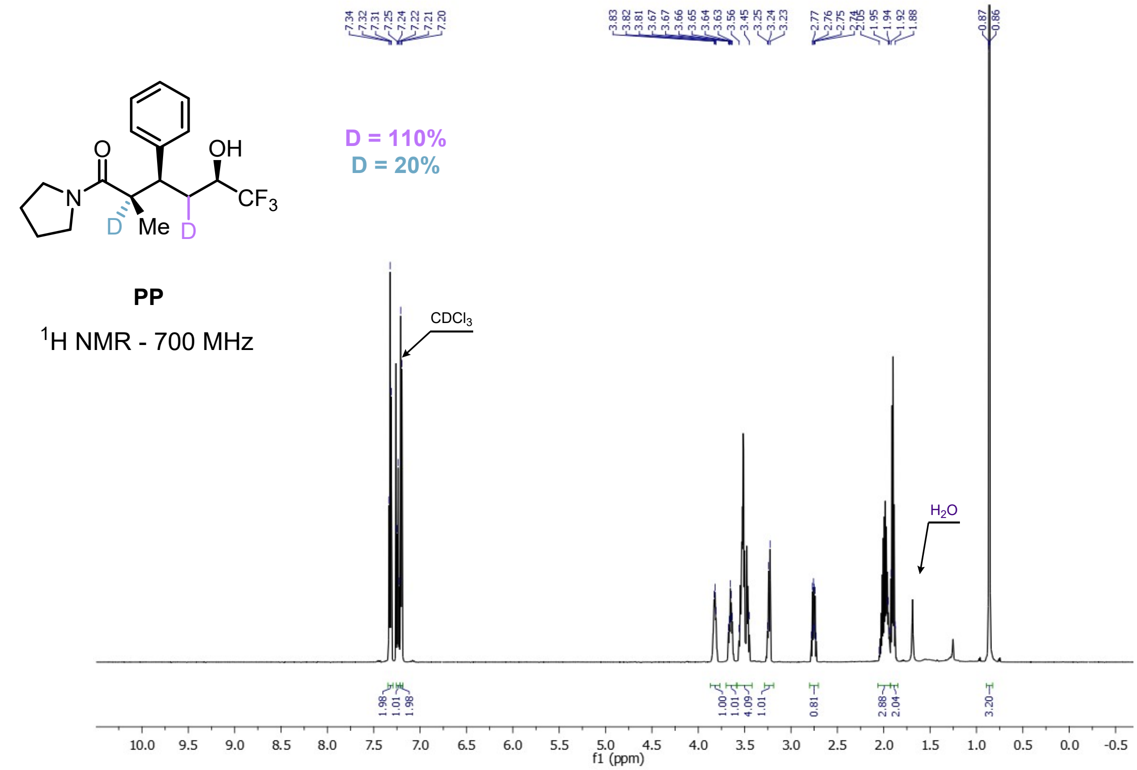

(2*R*,3*S*,5*S*)-6,6,6-trifluoro-5-hydroxy-2-methyl-3-phenyl-1-(pyrrolidin-1-yl)hexan-1-one-5-d (10)

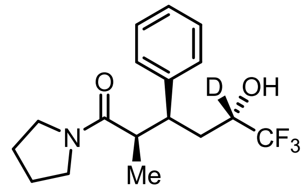

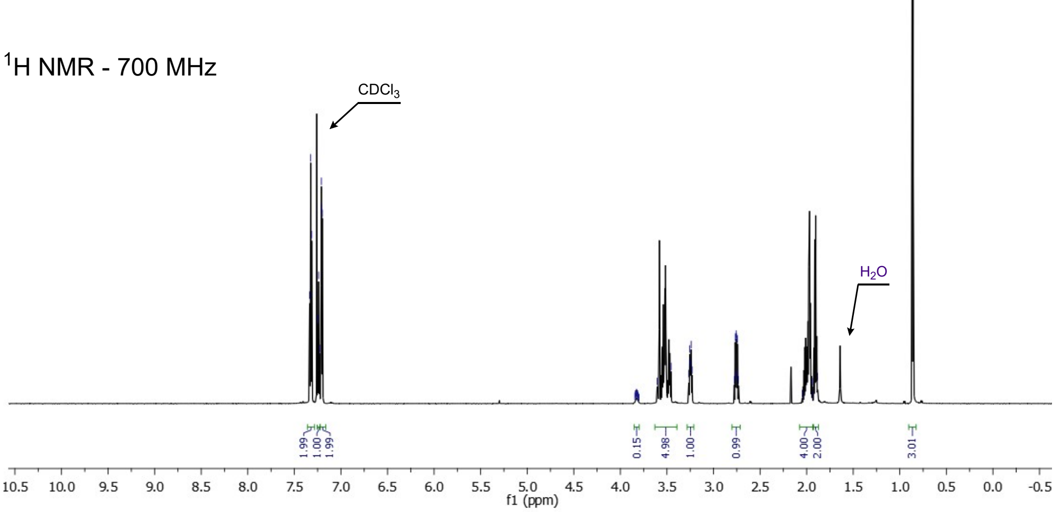

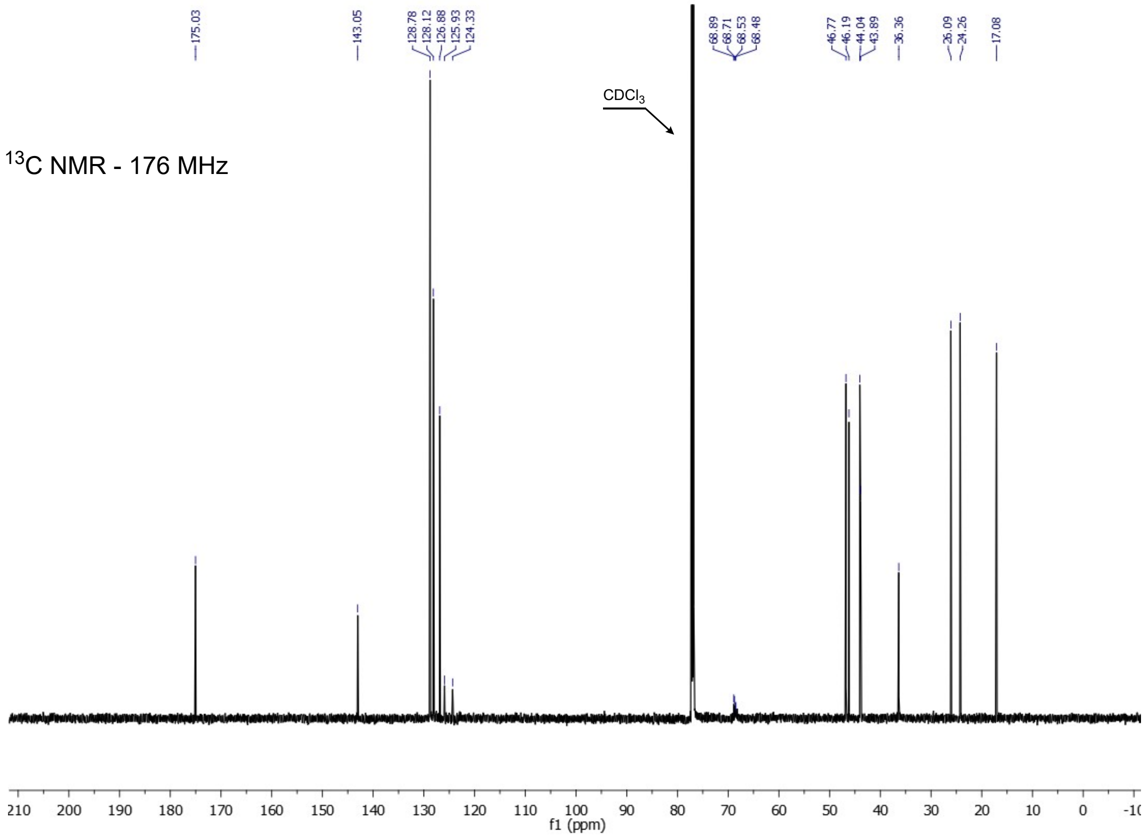

^{19}F NMR - 659 MHz

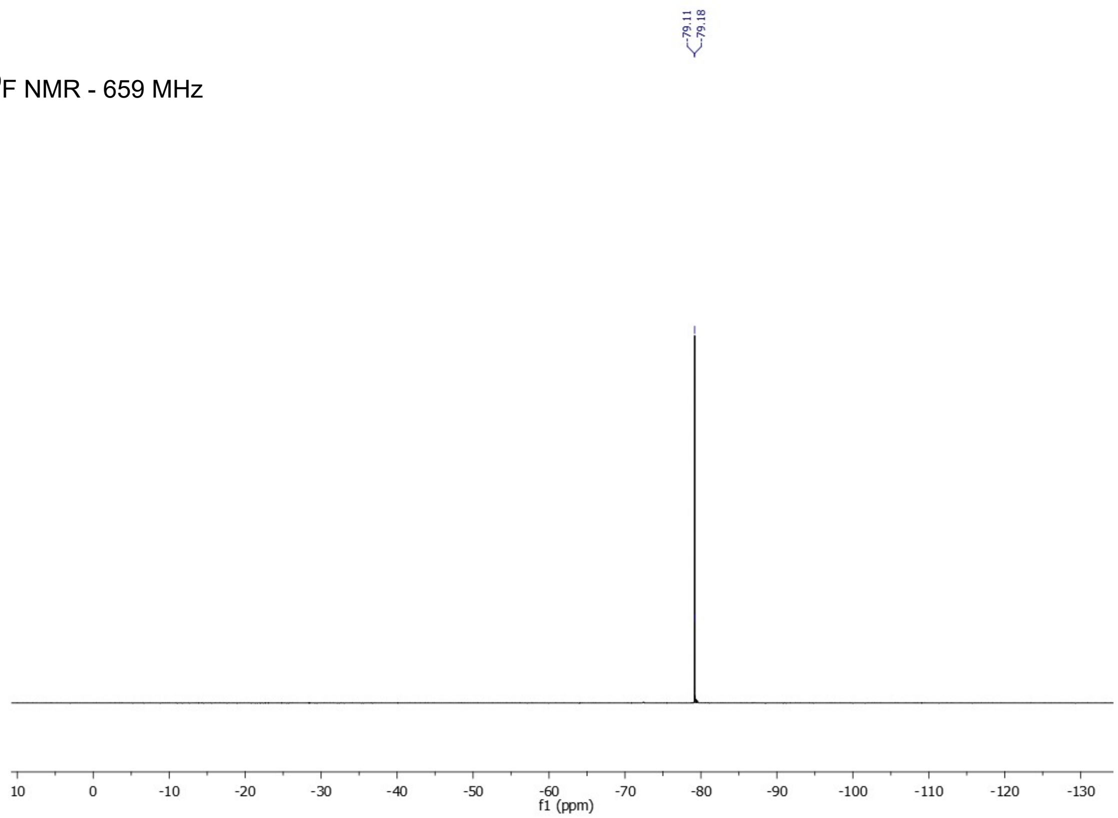

10.References

[1] X. Liu, J. Liu, J. Wu, C.-C. Li, *J. Org. Chem* **2021**, *86*, 11125–11139.

[2] T. A. Hamlin, C. B. Kelly, N. E. Leadbeater, *Eur. J. Org. Chem.* **2013**, *2013*, 3658–3661.

[3] L. C. Morrill, J. Douglas, T. Lebl, A. M. Z. Slawin, D. J. Fox, A. D. Smith, *Chem Sci* **2013**, *4*, 4146.

[4] K. Zhang, D. Rombach, N. Y. Nötel, G. Jeschke, D. Katayev, *Angew. Chem. Int. Ed.* **2021**, *60*, 22487–22495.

[5] A. G. Griesbeck, S. Bondock, P. Cygon, *J Am Chem Soc* **2003**, *125*, 9016–9017.

[6] Y. Zhao, X. Wang, J. Liu, *Synlett* **2008**, *7*, 1017-1020.

[7] L.-G. Xie, D. J. Dixon, *Chem. Sci.* **2017**, *8*, 7492–7497.

[8] P. Pracht, F. Bohle, S. Grimme, *Phys. Chem. Chem. Phys.* **2020**, *22*, 7169–7192.

[9] S. Grimme, *J. Chem. Theory Comput.* **2019**, *15*, 2847–2862.

[10] A. D. Becke, *J. Chem. Phys.* **1993**, *98*, 5648–5652.

[11] C. Lee, W. Yang, R. G. Parr, *Phys. Rev. B.* **1988**, *37*, 785–789.

[12] S. H. Vosko, L. Wilk, M. Nusair, *Can. J. Phys.* **1980**, *58*, 1200–1211.

[13] P. J. Stephens, F. J. Devlin, C. F. Chabalowski, M. J. Frisch, *J. Phys. Chem.* **1994**, *98*, 11623–11627.

[14] S. Grimme, J. Antony, S. Ehrlich, H. Krieg, *J. Chem. Phys.* **2010**, *132*, 154104.

[15] F. Weigend, R. Ahlrichs, *Phys. Chem. Chem. Phys.* **2005**, *7*, 3297.

[16] S. Grimme, S. Ehrlich, L. Goerigk, *J. Comput. Chem.* **2011**, *32*, 1456–1465.

[17] E. Cancès, B. Mennucci, J. Tomasi, *J. Chem. Phys.* **1997**, *107*, 3032–3041.

[18] A. V. Marenich, C. J. Cramer, D. G. Truhlar, *J. Phys. Chem. B* **2009**, *113*, 6378–6396.

[19] Frisch, M. J.; Trucks, G. W.; Schlegel, H. B.; Scuseria, G. E.; Robb, M. A.; Cheeseman, J. R.; Scalmani, G.; Barone, V.; Mennucci, B.; Petersson, G. A.; Nakatsuji, H.; Caricato, M.; Li, X.; Hratchian, H.; Izmaylov, A.; Bloino, J.; Zheng, G.; Sonnenberg, J.; Hada, M.; Ehara, M.; Toyota, K.; Fukuda, R.; Hasegawa, J.; Ishida, M.; Nakajima, T.; Honda, Y.; Kitao, O.; Nakai, H.; Vreven, T.; Montgomery, J.; Peralta, J.; Ogliaro, F.; Bearpark, M.; Heyd, J.; Brothers, E.; Kudin, K.; Staroverov, V.; Kobayashi, R.; Normand, J.; Raghavachari, K.; Rendell, A.; Burant, J.; Iyengar, S.; Tomasi, J.; Cossi, M.; Rega, N.; Millam, J.; Klene, M.; Knox, J.; Cross, J.; Bakken, V.; Adamo, C.; Jaramillo, J.; Gomperts, R.; Stratmann, R.; Yazyev, O.; Austin, A.; Cammi, R.; Pomelli, C.; Ochterski, J.; Martin,

R.; Morokuma, K.; Zakrzewski, V.; Voth, G.; Salvador, P.; Dannenberg, J.; Dapprich, S.; Daniels, A.; Farkas; Foresman, J.; Ortiz, J.; Cioslowski, J.; Fox, D. Gaussian 16, Revision C.01; Gaussian, Inc., Wallingford CT, 2016..

[20] G. M. Sheldrick, *Acta. Crystallogr. A. Found. Adv.* **2015**, *71*, 3–8.

[21] G. M. Sheldrick, *Acta. Crystallogr. C. Struct. Chem.* **2015**, *71*, 3–8.

[22] O. V. Dolomanov, L. J. Bourhis, R. J. Gildea, J. A. K. Howard, H. Puschmann, *J. Appl. Crystallogr.* **2009**, *42*, 339–341.

[23] C. B. Hübschle, G. M. Sheldrick, B. Dittrich, *J. Appl. Crystallogr.* **2011**, *44*, 1281–1284.
